# SOI: robust identification of orthologous synteny with the *Orthology Index* and broad applications in evolutionary genomics

**DOI:** 10.1093/nar/gkaf320

**Published:** 2025-04-18

**Authors:** Ren-Gang Zhang, Hong-Yun Shang, Richard Ian Milne, Fabricio Almeida-Silva, Hengchi Chen, Min-Jie Zhou, Heng Shu, Kai-Hua Jia, Yves Van de Peer, Yong-Peng Ma

**Affiliations:** State Key Laboratory of Plant Diversity and Specialty Crops/Yunnan Key Laboratory for Integrative Conservation of Plant Species with Extremely Small Populations, Kunming Institute of Botany, Chinese Academy of Sciences, Kunming 650201, China; University of the Chinese Academy of Sciences, Beijing 101408, China; State Key Laboratory of Plant Diversity and Specialty Crops/Yunnan Key Laboratory for Integrative Conservation of Plant Species with Extremely Small Populations, Kunming Institute of Botany, Chinese Academy of Sciences, Kunming 650201, China; University of the Chinese Academy of Sciences, Beijing 101408, China; Institute of Molecular Plant Sciences, School of Biological Sciences, University of Edinburgh, Edinburgh EH9 3JH, UK; Department of Plant Biotechnology and Bioinformatics, Ghent University, 9052 Ghent, Belgium; VIB Center for Plant Systems Biology, VIB 9052 Ghent, Belgium; Department of Plant Biotechnology and Bioinformatics, Ghent University, 9052 Ghent, Belgium; VIB Center for Plant Systems Biology, VIB 9052 Ghent, Belgium; State Key Laboratory of Plant Diversity and Specialty Crops/Yunnan Key Laboratory for Integrative Conservation of Plant Species with Extremely Small Populations, Kunming Institute of Botany, Chinese Academy of Sciences, Kunming 650201, China; University of the Chinese Academy of Sciences, Beijing 101408, China; State Key Laboratory of Plant Diversity and Specialty Crops/Yunnan Key Laboratory for Integrative Conservation of Plant Species with Extremely Small Populations, Kunming Institute of Botany, Chinese Academy of Sciences, Kunming 650201, China; University of the Chinese Academy of Sciences, Beijing 101408, China; Institute of Crop Germplasm Resources, Shandong Academy of Agricultural Sciences, Jinan 250100, China; Department of Plant Biotechnology and Bioinformatics, Ghent University, 9052 Ghent, Belgium; VIB Center for Plant Systems Biology, VIB 9052 Ghent, Belgium; Department of Biochemistry, Genetics and Microbiology, Centre for Microbial Ecology and Genomics, University of Pretoria, Pretoria 0028, South Africa; College of Horticulture, Academy for Advanced Interdisciplinary Studies, Nanjing Agricultural University, Nanjing 210095, China; State Key Laboratory of Plant Diversity and Specialty Crops/Yunnan Key Laboratory for Integrative Conservation of Plant Species with Extremely Small Populations, Kunming Institute of Botany, Chinese Academy of Sciences, Kunming 650201, China

## Abstract

With the explosive growth of whole-genome datasets, accurate detection of orthologous synteny has become crucial for reconstructing evolutionary history. However, current methods for identifying orthologous synteny face great limitations, particularly in scaling with varied polyploidy histories and accurately removing out-paralogous synteny. In this study, we developed a scalable and robust approach, based on the *Orthology Index* (*OI*), to effectively identify orthologous synteny. Our evaluation across a large-scale empirical dataset with diverse polyploidization events demonstrated the high reliability and robustness of the *OI* method. Simulation-based benchmarks further validated the accuracy of our method, showing its superior performance against existing methods across a wide range of scenarios. Additionally, we explored its broad applications in reconstructing the evolutionary histories of plant genomes, including the inference of polyploidy, identification of reticulation, and phylogenomics. In conclusion, *OI* offers a robust, interpretable, and scalable approach for identifying orthologous synteny, facilitating more accurate and efficient analyses in plant evolutionary genomics.

## Graphical Abstract

**Graphical Abstract:**
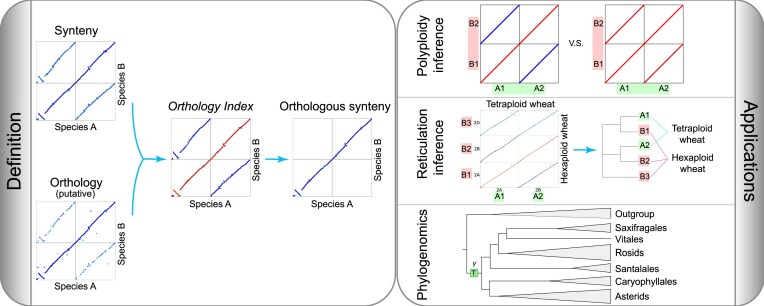

## Introduction

Synteny is commonly used to describe the conservation of the order of genes on chromosomes or genomic regions inherited from a common ancestor [1, 2], although the original definition of synteny simply refers to the co-localization of gene loci on the same chromosome within a genome [3]. Based on the common usage, synteny may arise from speciation events or originate via whole or segmental genome duplications. Synteny can be orthologous or paralogous, which we define respectively as arising from speciation, or arising from genome duplication, following the definitions of orthology and paralogy [4]. Paralogous synteny can be further subdivided into in-paralogous and out-paralogous categories, respectively resulting from a genome duplication event that occurred after, or before, a given speciation event [4, 5].

Similar to orthology, orthologous synteny is highly useful for reconstructing evolutionary histories. This is especially true for closely related lineages with conserved synteny, as it helps establish reliable orthologous relationships [6]. The reconstruction of evolutionary histories of organisms, including inference of the tree/network of life, identification of polyploidy events, and placement of these events on the tree/network, typically relies on the orthologous relationships at gene, chromosomal block, chromosome, subgenome, and/or whole-genome scales. Confidence in the reconstruction generally increases with both the number and reliability of orthologous genes or loci involved. Indeed, phylogenomic reconstructions using genome-scale data have become the gold standard for understanding the evolution of lineages across the tree of life [7]. Orthologous synteny has been established as a proxy to generate a maximum number of reliable orthologs, from the chromosomal to the subgenomic/genomic scales, for example, in the reconstruction of evolutionary histories for the octoploid strawberry [8] and the major angiosperm clades [9]. Identifying orthologous synteny is also vital for other synteny-based analyses, such as pan-genome construction, ancestral genome reconstruction, polyploidy inference, and subgenome phasing [8, 10–13]. However, accurate identification of orthologous synteny remains challenging, especially in plants, where pervasive and recurrent whole-genome duplication (WGD, also known as polyploidization) events have produced abundant out-paralogous synteny. This can significantly complicate the inference of orthologous relationships [7, 14] and may mislead the reconstruction of evolutionary history. For example, the overlooked orthologous relationships in the synteny between two *Papaver* species resulted in an incorrect interpretation of the polyploidy history in these species [15–17].

To date, two main strategies have been employed to identify orthologous synteny. The first strategy, termed the “criterion strategy”, is to filter the detected synteny using certain criteria, which are usually case-specific and thus challenging to scale for large-scale datasets. One widely-used criterion is the number of synonymous substitutions per synonymous site (*K*_S_). Since the WGD event (which produces paralogy) and speciation event (which produces orthology) occurred at different times in the past, *K*_S_ values between syntenic gene pairs can be used to differentiate the orthologous and paralogous synteny [18, 19]. However, such events occur at different times in different lineages, and substitution rates also vary between lineages [20, 21], both of which cause *K*_S_ values to vary case by case. Consequently, *K*_S_-based methods are not always effective for distinguishing syntenic blocks (SBs) from different evolutionary events [22], and cannot be universally applied to the large-scale automated identification of orthologous synteny. Another criterion, *homo*, has been proposed for use with WGDI to extract the best homology (orthology) of SBs [12]. Unfortunately, this criterion relies on a parameter, *multiple*, to define the top number of hits as best hits [12], but *multiple* cannot be universally applied to different cases with different syntenic depth ratios. An additional tool, QUOTA-ALIGN [22], was developed to screen orthologous SBs under given constraints on syntenic depths (i.e. *QUOTA*). However, setting the *QUOTA* criterion requires prior knowledge of lineage-specific WGD histories, which varies across different species. For example, *QUOTA* needs to be set to 1:1 for *Arabidopsis thaliana* and *Arabidopsis lyrata*, but adjusted to 4:2 for *A. thaliana* and poplar [22]. This variability makes it challenging to apply QUOTA-ALIGN uniformly across large-scale datasets with multiple species pairs and diverse WGD scenarios. Similar issues arise when setting the *multiple* parameter in WGDI -c option.

An alternative strategy, the “pre-inferred strategy”, uses pre-inferred orthologs to call synteny, with tools such as MCScanX_h [23]. This strategy is scalable for large-scale datasets. However, hidden out-paralogs (i.e. false positives in ortholog inference) in the pre-inferred orthologs may result in out-paralogous synteny that need to be further removed using the aforementioned “criterion” strategy. For example, undesirable out-paralogous synteny was commonly observed in the synteny results from our previous work in *Populus* [18] and *Salix* [24] using this strategy. Moreover, the presence of hidden orthologs (i.e. false negatives in ortholog inference) can reduce the efficiency of the subsequent detection of orthologous synteny in practice. In addition, pre-inferred orthology and paralogy relationships can be corrected by the SCORPiOs tool, using synteny information and greater similarity of local gene retention patterns in orthologs relative to paralogs [25]. However, SCORPiOs relies on pre-inferred orthogroups, which may be constrained by the accuracy of orthogroup clustering. Like QUOTA-ALIGN and WGDI -c, SCORPiOs also requires prior knowledge of WGD histories.

To address these challenges and to identify orthologous synteny robustly for large-scale genomic datasets, we developed a scalable approach called the *Orthology Index* (*OI*), which describes the proportion of syntenic gene pairs that are pre-inferred as orthologs (see the ‘Materials and methods’ section). We evaluated the efficacy of *OI* using a large-scale dataset comprising 91 well-documented cases with diverse WGD and speciation events, as well as a simulated benchmarking dataset. Our results demonstrated the high robustness and accuracy of *OI*, which generally outperformed existing methods. We further explored its broad applicability in evolutionary inference, including analyses of polyploidy, reticulation, and phylogenomics. We finally integrated the index into a toolkit (freely available from https://github.com/zhangrengang/SOI) to facilitate its use.

## Materials and methods

### Empirical data collection and pre-processing

Genomic data were sourced from public databases or from the corresponding authors, as detailed in Supplementary Table S2. For each species pair, an all-versus-all sequence comparison of proteins was performed using DIAMOND v0.9.24 [26]. Orthologous relationships were inferred using OrthoFinder v2.3.1 (parameters: -M msa) [27]. SBs were identified using the “-icl” option of WGDI [12] v0.6.2 (default parameters). The *K*_S_ for homologous gene pairs was calculated using the “-ks” option of WGDI (default parameters).

### Definition of the *OI*

We introduce an index, named the *OI*, to distinguish orthologous SBs from out-paralogous ones. The index is defined as:

$OI = \frac{n}{m},$ where *m* is the total number of syntenic gene pairs in a block, and *n* is the number of pairs pre-inferred as orthologs. For instance, in a SB with 80 gene pairs, if 72 of these pairs are pre-inferred as orthologs, then *m*= 80 and *n*= 72, resulting in an *OI* value of 72/80 = 0.9. The *OI*, ranging from 0 to 1, thus represents the proportion of orthologous pairs within a SB. Orthologous synteny is expected to result in high *OI* values (approaching 1 with perfect recall of pre-inferred orthologs), whereas out-paralogous synteny is expected to result in relatively low *OI* values (approaching 0 with perfect precision of pre-inferred orthologs). Based on our analysis of many varied cases (Fig. 1 and Supplementary Figs S1–S90), the major peak with the highest *OI* typically corresponds to orthologous synteny.

**Figure 1.**
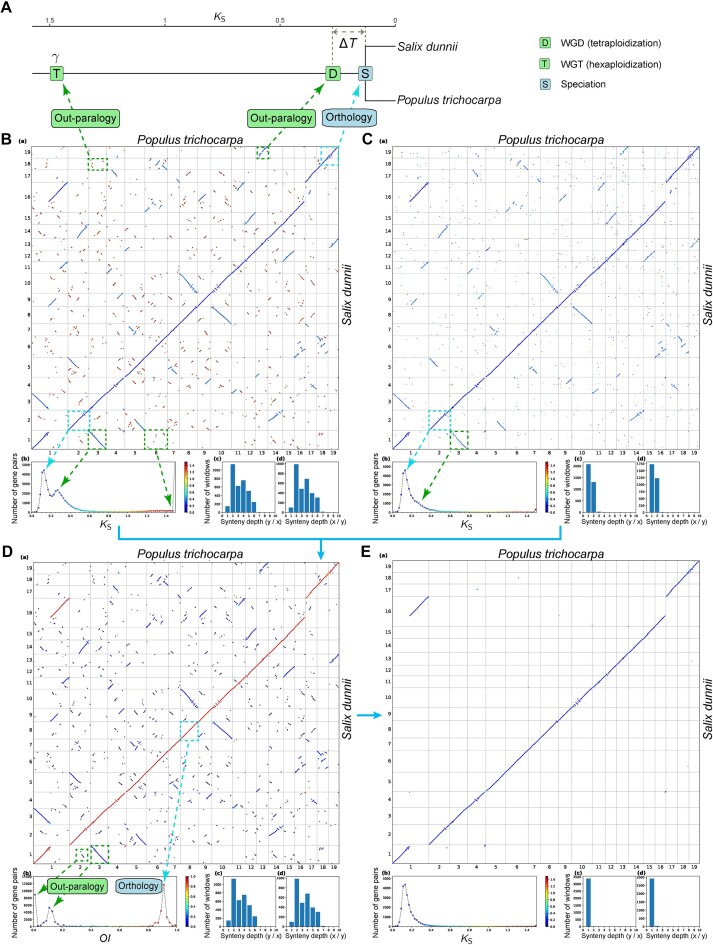
Results from using the *OI* to identify orthologous synteny for the typical Salicaceae case. (**A**) Schematic representation of the evolutionary history of the poplar and willow genomes, adapted from the literature [45–48]. (**B**) *K*_S_-colored dot plots showing synteny detected by WGDI (-icl), with an observable distinction between the three categories of SBs derived from three evolutionary events (three peaks: *K*_S_≈ 1.5, *K*_S_≈ 0.27, and *K*_S_≈ 0.13). (**C**) *K*_S_-colored dot plots illustrating the orthology as inferred by OrthoFinder2, with an observable high proportion (∼15%) of hidden out-paralogs (*K*_S_≈ 0.27). (**D**) *OI*-colored dot plots: integrating synteny (B) and orthology (C), showing polarized distinction of the three categories of SBs (three peaks: *OI*≈ 0, *OI*≈ 0.1, and *OI*≈ 0.9). (**E**) *K*_S_-colored dot plots of synteny after applying an *OI* cutoff of 0.6, showing clean one-to-one orthology as expected from the evolutionary history. Panels (B)–(E) are plotted using the ‘dotplot’ subcommand with four subplots: (a) dot plots colored by *K*_S_ or *OI* (x-axis and y-axis, chromosomes of the two genomes; a dot indicates a homologous gene pair between the two genomes); (b) histogram of *K*_S_ or *OI* (x-axis, *K*_S_ or *OI*; y-axis, number of homologous gene pairs), using the same color map as the dot plots; and (c) and (d) synteny depth (indicative of relative ploidy) across 50-gene windows (x-axis, synteny depth; y-axis, number of windows), relative to the genomes on the x-axis (c) or y-axis (d) of subplot a. Examples of the SBs from three evolutionary events [referred to as WGT-SBs (*K*_S_≈ 1.5, *OI*≈ 0), WGD-SBs (*K*_S_≈ 0.27, *OI*≈ 0.1), and S-SBs (*K*_S_≈ 0.13, *OI*≈ 0.9)] are highlighted with dashed squares. These are associated with the evolutionary events and peaks of *K*_S_ or *OI*, indicated by arrows, and labeled as ‘Out-paralogy’ or ‘Orthology’. Additional cases illustrating other lineages can be found in Supplementary Figs S1–S90 (summarized in Supplementary Table S1).

### Implementation of the *OI*

Using this index as a foundation, we developed a user-friendly, all-in-one toolkit (called SOI) for visualization and downstream analyses. The subcommand ‘dotplot’ enables visualization and evaluation of synteny, with the dots colored by the *OI* or *K*_S_ values. The subcommand ‘filter’ retrieves orthologous synteny by discarding all SBs with an *OI* value below the default threshold of 0.6. However, users can also apply more stringent criteria (higher *OI* and block length cutoffs) to obtain longer, highly credible blocks. The subcommand ‘cluster’ groups orthologous syntenic genes into syntenic orthogroups (SOGs) by constructing an orthologous syntenic graph and applying the Markov Cluster (MCL) algorithm [28] to perform graph clustering. This process breaks weak links and bridges unexpectedly disrupted links. The clustering algorithm is widely employed by popular tools of orthogroup clustering, such as OrthoFinder2 [27], SonicParanoid2 [29], and OrthoMCL [30]. The subcommand ‘outgroup’ retrieves syntenic orthologs from outgroups that lack WGDs shared with ingroups. The subcommand ‘phylo’ reconstructs multi-copy or single-copy gene trees, by aligning protein sequences with MAFFT v7.481 [31], converting protein alignments to codon alignments with PAL2NAL v14 [32], trimming alignments with trimAl v1.2 [33] (parameter: -automated1), and reconstructing maximum-likelihood trees with IQ-TREE v2.2.0.3 [34]. These gene trees serve as input to infer a species tree with the coalescence-based method ASTRAL-Pro v1.10.1.3 [35]. The default threshold for missing taxa is set to 40%, according to an evaluation for this parameter [36].

This tool is implemented in Python3 and supports synteny outputs from state-of-the-art synteny detectors, including MCscan/JCVI [37], MCscanX [23] and WGDI [12], as well as orthology outputs from OrthoFinder2 [27], Broccoli [38], SonicParanoid2 [29], Proteinortho6 [39], InParanoid [40], OrthoMCL [30], and other tools upon request. These orthology inference methods have competitive accuracy [29, 38, 41] and thereby should produce similar *OI* values. The tool can be easily installed using the conda environment or the Apptainer/Singularity container system [42]. Since both synteny and orthology analyses are generally standard procedures in genomics projects, the *OI* tool can be seamlessly integrated into these and their downstream pipelines.

### Simulation-based benchmarks

To validate the feasibility and robustness of the *OI*, we conducted simulation-based benchmarks. We simulated a shared-WGD scenario similar to the Salicaceae case (Fig. 1A) using Zombi [43]. The input species tree was “(((A1:0.2, B1:0.2):Δ*T*, (A2:0.2, B2:0.2):Δ*T*):0.2, O:Δ*T*+ 0.4):0.1”, where the subgenomes of taxon A (A1 and A2) and taxon B (B1 and B2) were each treated as pseudo-species, with O as an outgroup species. The variable Δ*T* represents the time lag between the shared WGD event and the subsequent speciation event of taxa A and B, ranging from 0.01 to 1 substitution per site. This time lag is measured in terms of nucleotide substitutions per site and reflects the evolutionary distance between these two events. For each simulation, 5000 coding sequences were generated with other parameters (e.g. those regarding substitutions and chromosomal rearrangements) kept at default values (see https://github.com/AADavin/Zombi/tree/master/Parameters/). The simulated three-taxon genomic data were then input into the aforementioned OrthoFinder2–WGDI–SOI pipeline to calculate the *K*_S_ and *OI* values and to identify syntenic orthologs between taxa A and B. True positives (TP), false positives (FP), and false negatives (FN) were counted from the results, and precision [*P* = TP/(TP + FP)], recall [*R* = TP/(TP + FN)], and F1-score [2 × *P* × *R*/(*P* + *R*)] were calculated. The simulation was independently replicated 50 times for each Δ*T* condition.

To assess the influence of chromosome evolution on the identification of syntenic orthologs, we adjusted the parameters for chromosomal rearrangements in Zombi (i.e. genome-wide rates of DUPLICATION, TRANSFER, LOSS, INVERSION, TRANSPOSITION, and ORIGINATION) to 10, 100, and 1000 times their default values. We also benchmarked our method against existing tools for identifying orthologous synteny: QUOTA-ALIGN, WGDI v0.6.2 -c option, MCScanX_h, and SCORPiOs v2.0.0. For QUOTA-ALIGN, parameters were set to ‘- -Dm=20 --min_size=5 - -quota=1:1’ with DIAMOND results pre-filtered by ‘blast_to_raw.py - -tandem_Nmax=10 - -cscore=.5’ [22]. For WGDI -c, parameters were configured as ‘multiple=1 homo=0.5,1 pvalue=0.2 block_length=5’ [11, 12]. MCScanX_h used OrthoFinder2-inferred orthologs with default parameters. For SCORPiOs, codon alignments were constructed using the MAFFT–PAL2NAL pipeline based on orthogroups inferred by OrthoFinder2, and parameters for the WGD event were set accordingly, with other parameters kept at default. The precision and recall on identifying orthologs were used to assess the performance of these methods as described above.

### Evaluation based on empirical data

To evaluate the robustness of the *OI* method, we compiled an empirical dataset comprising 91 well-documented plant species pairs, each with at least one shared polyploidy event (Supplementary Table S1, Fig. 1, and Supplementary Figs S1–S90). Given the known WGD history and consistent differences in patterns of *K*_S_ values, *OI* values, and fragmentation extent, it is straightforward to visually distinguish orthologous synteny from out-paralogous synteny with colored dot plots. Typically, orthologous synteny features the lowest *K*_S_ peaks, the least fragmentation, and the highest *OI* peaks, while out-paralogous synteny shows higher *K*_S_ values, greater fragmentation, and lower *OI* values (e.g. Fig. 1). In particular, the *OI* value distribution shows a polarized pattern as expected, facilitating easy identification of orthologous synteny. Setting the *OI* cutoff between 0.5 and 0.6 allows us to retrieve orthologous SBs for most cases. A higher cutoff leads to a higher false negative rate, filtering out true orthologous synteny, while a lower cutoff results in a higher false positive rate, retaining out-paralogous synteny. To balance these trade-offs in a few difficult cases, we selected a cutoff of 0.6 as the default. Under this criterion, the retrieved orthologous synteny aligns with the expected synteny depth based on the known WGD history and consistently matches the patterns of *OI*, *K*_S_, and fragmentation extent. However, because the true evolutionary history of each gene is typically unknown, establishing a gene-level ground truth for the empirical evaluation remains challenging [44].

To evaluate how genome assembly and annotation quality affect the robustness of the *OI* method, we used the Salicaceae syntenic ortholog data set (Fig. 1) as a benchmark. We simulated varying levels of annotation completeness by randomly subsampling 10%–90% of the genes from the *Salix* genome, creating relative completeness values ranging from 0.1 to 0.9. To prevent recall underestimation, the removed genes were also excluded from the benchmark dataset for each simulation. We also simulated variations in assembly continuity by fragmenting the *Salix* genome’s chromosomes, producing relative N50 values ranging from 0.001 to 0.9. The relative N50 is the ratio of the post-fragmentation N50 to the pre-fragmentation N50.

## Results and discussion

### Identification of orthologous synteny using the *OI*

We aimed to develop a robust and accurate method to distinguish orthologous synteny from out-paralogous synteny in genomes of any two species that shared a WGD event (producing out-paralogous synteny) prior to their divergence (speciation event, producing orthologous synteny). This method should allow users to generally avoid adjusting parameters for varied scenarios, despite the presence of species-specific WGDs. To evaluate our approach, we compiled ninety well-documented plant species pairs, each descended from a common ancestor that had undergone at least one polyploidy event (Supplementary Table S1 and Supplementary Figs S1–S90), along with the typical poplar–willow (Salicaceae) case (Fig. 1).

Fig. 1 shows an analysis for the Salicaceae case, represented by *Populus trichocarpa* and *Salix dunnii*. The two genera speciated ∼50 million years ago (Mya) [45, 46], after a WGD event in their common ancestor ∼60 Mya [45–48] (Fig. 1A). The time lag between the shared WGD event and the subsequent speciation event is denoted as Δ*T* (Fig. 1A), which we considered a key factor that may influence the ability to distinguish between these two sources of synteny. Additionally, both genera also share a much older paleohexaploidization event (the γ event, whole-genome triplication or WGT) in the common ancestor of core eudicots ∼120 Mya [49], making three detectable evolutionary events in total (Fig. 1A).

With the “criterion” strategy using the synteny detector WGDI [12], SBs derived from these three evolutionary events could be identified and distinguished visually using their distinct *K*_S_ values and fragmentation extent (the more ancient the event, the more fragmented the blocks or greater the chromosomal rearrangements, and the higher the *K*_S_ value) (Fig. 1B). These three categories of SBs were denoted WGT-SBs (*K*_S_≈ 1.5, most fragmented, out-paralogous), WGD-SBs (*K*_S_≈ 0.27, moderately fragmented, out-paralogous), and S-SBs (*K*_S_≈ 0.13, least fragmented, orthologous) (Fig. 1B) according to the evolutionary events (i.e. WGT, WGD, and speciation) (Fig. 1A). However, the recent two *K*_S_ peaks derived from WGD-SBs (out-paralogy) and S-SBs (orthology) largely overlapped (Fig. 1B) and were difficult to neatly split with a cutoff. This overlap is likely due to varied evolutionary rates among genes, resulting in similar accumulated substitutions for genes from different events. Indeed, of the other empirical cases examined here, there were only a few (e.g. Supplementary Figs S4 and S30) where a *K*_S_ cutoff could explicitly distinguish orthology and out-paralogy for synteny. Moreover, for some extreme cases where Δ*T* was quite small, *K*_S_ peaks from two events (shared WGD and speciation) overlapped completely, showing only one peak (e.g. Supplementary Figs S1, S8, S16, and S34) with no detectable distinction between orthology and out-paralogy.

With the “pre-inferred” strategy using the orthology inference method OrthoFinder2 [27], the peak (*K*_S_≈ 0.27) of out-paralogs from WGD-SBs was significantly reduced, while out-paralogs from the older WGT event (WGT-SBs, *K*_S_≈ 1.5) were almost entirely eliminated (Fig. 1C). This suggests that OrthoFinder2 has relatively high accuracy in orthology inference (∼85% orthologs located in the orthologous chromosomal segments for the Salicaceae case). However, a substantial number of the inferred “orthologous” genes (∼15%) exhibited synteny in out-paralogous blocks (*K*_S_≈ 0.27; Fig. 1C), suggesting that they are hidden paralogs and would introduce out-paralogous synteny if they were imported into synteny detectors such as MCScanX_h [23]. This problem was also observed in numerous cases where the Δ*T* was not substantial (e.g. Supplementary Figs S1, S5, S6, S8, and S10), which could be attributed to systematic errors or biased gene loss, and could have a confounding effect on downstream analyses, such as phylogenomics [7].

Consequently, we introduced a straightforward index, named the *OI* to determine the orthology of a SB, combining the algorithmic advances of the two methods described above. *OI* represents the proportion of gene pairs pre-inferred to be orthologs within a SB. Thus, orthologous and out-paralogous synteny predict *OI* values of 1 and 0 respectively, i.e. with all syntenic gene pairs are inferred to be orthologs, or none, respectively. However, for real datasets, due to the limited recall and precision of orthology inference methods, the *OI* distribution could deviate from the expectations. For the Salicaceae case, the WGT-SBs and WGD-SBs (both out-paralogous but of different ages) exhibited an *OI* of ∼0 and 0.1, respectively, whereas the S-SBs (orthologous) produced an *OI* peak of ∼0.9 (Fig. 1D), close to the expectations. In this case the *OI* distribution reflected the same orthologous and out-paralogous relationships as *K*_S,_ but was better able to distinguish between them (Fig. 1B and D). Remarkably, the *OI* peaks derived from orthology and out-paralogy did not overlap, but showed a polarized pattern with a very clear, wide dividing range (*OI* = ∼0.3–0.7) between the orthology and out-paralogy peaks (Fig. 1D). Therefore, in this case, the *OI* was easily able to distinguish orthology from out-paralogy, with a cutoff between 0.3 and 0.7 (Fig. 1D).

**Figure 2.**
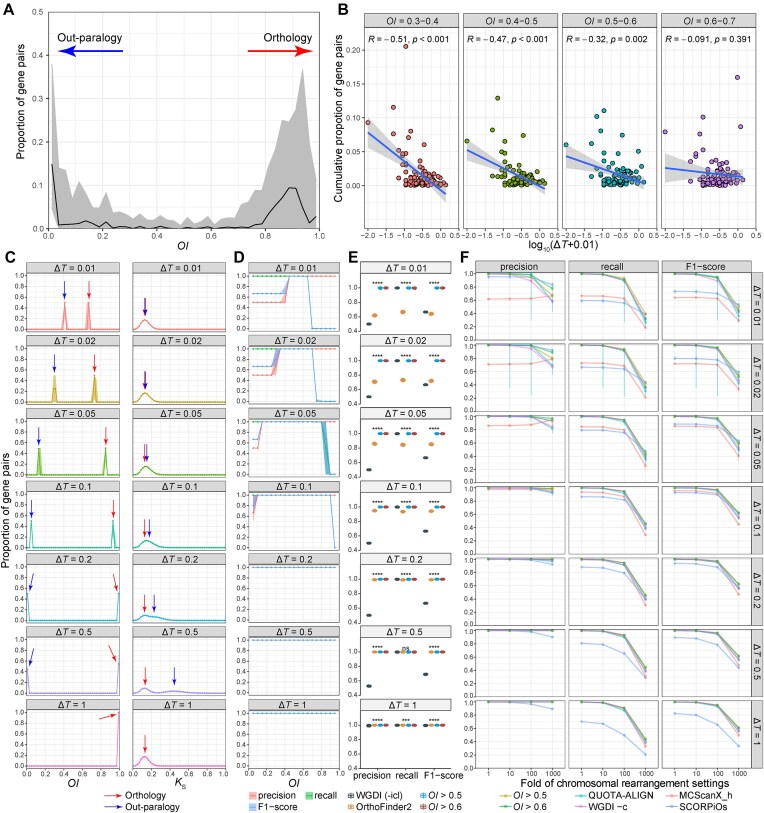
The performance of the *OI* in identifying orthologous synteny in empirical and simulated datasets. (**A**) Summary of *OI* distributions in the 91 empirical test cases. The black line and gray shadow represent the median and percentile-based 95% confidence interval (CI) values, respectively. (**B**) The correlation between Δ*T* and the noise around *OI*= 0.5 in the empirical datasets. The Δ*T* values of empirical datasets were estimated from *K*_S_ peak values of shared WGD and speciation events. The noise is defined as the cumulative proportion of syntenic gene pairs falling within the *OI* intervals of 0.3–0.4, 0.4–0.5, 0.5–0.6, and 0.6–0.7. These putative noises are generally unexpected and likely arise from false orthology inference. *R* is the Pearson’s correlation coefficient. (**C**) Comparisons of inter-genomic *K*_S_ and *OI* distributions in the simulated datasets at different Δ*T* settings (Δ*T* ε {0.01, 0.02, 0.05, 0.1, 0.2, 0.5, 1}, measured in substitutions per site). The line/point and shadow represent the median and 95% CI values from 50 repeated simulations, respectively. (**D**) Comparisons of precision, recall and F1 scores of orthology identification using different *OI* cutoffs (0.05–0.95) in simulated benchmarks. The line/point and shadow represent the median and 95% CI values from 50 repeated simulations, respectively. (**E**) Comparisons of precision, recall and F1 score of orthology identification using WGDI (-icl option), OrthoFinder2 and *OI* with cutoffs of 0.5 and 0.6, based on the simulated benchmarks. The boxplot represents the values from 50 repeated simulations. ns, *P* > .05; ***P ≤ .001; ****P ≤ .0001; Kruskal-Wallis test. (**F**) Comparisons of *OI* and other tools for identifying orthologous synteny, based on the simulated benchmarks on varied levels of Δ*T* (0.01–1) and chromosome evolution parameters (*fold* ε {1, 10, 100, 1000}). The line and point represent the median values of precision, recall or F1 score, and the error bar indicates the 95% CI from 50 repeated simulations. See also Supplementary Fig. S92 for additional information.

We next applied the index analysis to ninety study cases with diverse polyploidy histories and varying Δ*T* (Supplementary Table S1 and Supplementary Figs S1–S90). In nearly all instances (85 out of 90), there were also clear divides around *OI* = ∼0.5–0.6 (although minor noise around *OI* = ∼0.5–0.6 can be observed in some cases) (Fig. 2A and B, and Supplementary Figs S1–S90D), suggesting a potentially unified *OI* criterion for identifying orthologous synteny. We empirically set the *OI* cutoff to 0.6 according to the *OI* distributions in these cases (Figs 1D and 2A, and Supplementary Figs S1–S90D), in order to balance the visible false positives (out-paralogy mis-identified as orthology) and false negatives (excessive removal of true orthologous synteny). With this cutoff, the *OI* method performed exceptionally and consistently well in identifying orthologous synteny across all these instances (Fig. 1E and Supplementary Figs S1–S90E). For example, in the Salicaceae case, an *OI* cutoff of 0.6 resulted in a clean 1:1 orthology relationship without visible false positives or false negatives (Fig. 1E), as expected. In this case, 12.2% of the SBs and 47.6% of the syntenic pairs were retained. Of the retained syntenic gene pairs, 90.4% were pre-inferred as orthologs by OrthoFinder2, while the remaining 9.6% were presumed to be orthologs but not identified by OrthoFinder2. Moreover, although some visible false positives or false negatives were observed in a few study cases, there were far fewer false positives than when using the above two strategies (Supplementary Figs S50, S68, S74, and S86). The method also showed robustness against variations in genome assembly continuity and gene annotation completeness (Supplementary Fig. S91). These findings highlight the efficiency and robustness of *OI* as a unified criterion for identifying orthologous synteny.

To validate the feasibility and robustness of *OI*, we further performed benchmarking by simulating a shared-WGD scenario similar to that of the Salicaceae case (Fig. 1A). Δ*T* was correlated to the unexpected noises around *OI*= 0.5 in the empirical datasets (Fig. 2B), and was thus considered a key parameter affecting the accuracy of identifying orthologous synteny. With varied Δ*T* settings (Δ*T* ε {0.01, 0.02, 0.05, 0.1, 0.2, 0.5, 1}, measured in unit of nucleotide substitutions per site), *OI* consistently outperformed *K*_S_ in distinguishing orthology from out-paralogy (Fig. 2C). Specifically, when Δ*T* was between 0.01 and 0.1, *K*_S_ values from both orthologous and out-paralogous synteny showed a single peak without any observable distinction. Even when Δ*T* reached 0.2, the two *K*_S_ peaks still overlapped significantly and could not be efficiently distinguished. In striking contrast, *OI* showed clear dividing ranges around *OI*= 0.5–0.6 for all tested Δ*T* values (Fig. 2C). By applying an *OI* cutoff of either 0.5 or 0.6, both precision and recall consistently approached 1.0 across various Δ*T* values (Fig. 2D). Moreover, when Δ*T* was between 0.01 and 0.05, where OrthoFinder2 showed a relatively low accuracy in ortholog inference (precision = 0.62–0.85, recall = 0.66–0.83, and F1 score = 0.64–0.84), the *OI* method with a cutoff of 0.5 or 0.6 significantly improved both the precision (0.992–1.0) and recall (0.992–1.0) of ortholog detection (Fig. 2E). This suggests that *OI* could significantly improve ortholog inference under these conditions and could therefore facilitate ortholog-based analyses such as phylogenomics and pangenomics.

Chromosome evolution can disrupt synteny and thus negatively impact synteny-based methods. To assess this impact, we increased the levels of chromosomal rearrangements to 10, 100, and 1000 times the default values (denoted as *fold*= 10, 100, and 1000) in our simulations. We also compared our method with existing tools, including QUOTA-ALIGN [22], WGDI -c option [12], MCScanX_h [23], and SCORPiOs [25]. When chromosomal rearrangements were extensive (*fold*= 1000) with a mean size of SBs around 10 genes, the recall of these synteny-based methods decreased sharply, while the precision of *OI* method remained high (precision > 0.95 when Δ*T*≥ 0.05; Fig. 2F). This suggests that *OI* can reliably identify orthologous synteny even under extensive chromosomal rearrangements. Furthermore, our method outperformed or matched the existing tools across nearly all tested conditions (Fig. 2F and Supplementary Fig. S92). SCORPiOs showed an obvious advantage in F1 score only when Δ*T* was small (Δ*T*≤ 0.05) and *fold* was very large (*fold*= 1000), but typically performed worse under other conditions (Fig. 2F and Supplementary Fig. S92). QUOTA-ALIGN and WGDI -c were competitive with the *OI* method in most conditions (Fig. 2F and Supplementary Fig. S92), yet require parameter adjustments based on lineage-specific WGD histories to achieve optimal performance, diminishing their scalability and robustness. The *OI* method and MCScanX_h are both scalable, but MCScanX_h performed poorly when Δ*T* < 0.1 (Fig. 2F and Supplementary Fig. S92). These results also support the robustness of the *OI* method, which maintained consistent performance across varied Δ*T* conditions (Fig. 2F).

Overall, the simulation-based benchmarking results were highly consistent with those above based on empirical datasets, demonstrating that the *OI* method generally outperforms the existing methods and is able to produce accurate results for identifying orthologous synteny. *OI* can therefore be applied in automated pipelines for large-scale datasets (ranging from dozens to hundreds of genomes at present) with a unified threshold of *OI*. We integrated the *OI* method into a toolkit (https://github.com/zhangrengang/SOI) to identify orthologous synteny and to facilitate downstream applications. The toolkit can output orthologous synteny (SBs that are identified as orthologous), syntenic orthologs (gene pairs within the orthologous SBs), and SOGs (gene families grouped from syntenic orthologs), providing substantial flexibility for downstream synteny-based analyses and ortholog/orthogroup-based analyses.

### Applications in polyploidy inference

**Figure 3.**
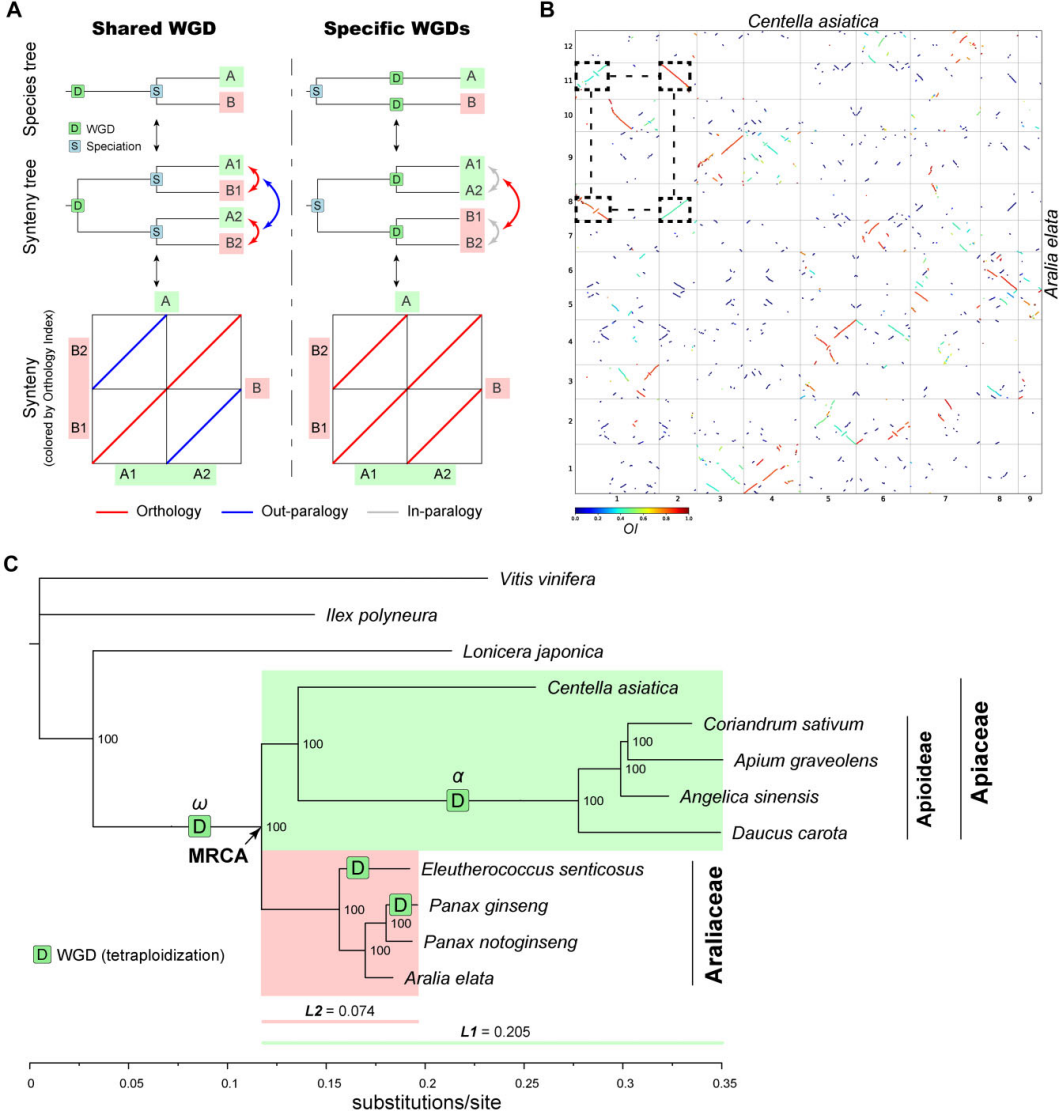
Inference of polyploidy in Apiales (Apiaceae + Araliaceae) genomes using the *OI*. (**A**) Schematic illustration of determining shared or lineage-specific WGD(s) hypotheses using the orthologous synteny patterns identified by the *OI*. Despite a similar 2:2 ratio of synteny depth, the two scenarios have distinct patterns of orthologous synteny (1:1 orthology vs. 2:2 orthology). Labels A and B indicate two species, and A1, A2 and B1, B2 indicate duplicated chromosomes or blocks from WGD event(s). (**B**) *OI*-colored dot plots indicating orthologous and out-paralogous synteny between the genomes of *C. asiatica* (Apiaceae) and *A. elata* (Araliaceae). A typical 1:1 orthology + 1:1 out-paralogy synteny pattern is highlighted by dashed squares. (**C**) Phylogeny reconstructed from the genomes of certain species in the Apiaceae and the Araliaceae (Apiales), with labels indicating polyploidization events. *L1* and *L2* represent the average branch length (substitution rate) of the Apiaceae and the Araliaceae, respectively, from their MRCA. Numbers at the nodes denote bootstrap values. The maximum-likelihood phylogenetic tree was reconstructed using IQ-TREE2, based on concatenated codon alignments of 2363 single-copy genes (with at most 20% taxa missing). Additional evidence supporting the inferred polyploidization events can be found in Supplementary Figs S93–S99.

Polyploidization (or WGD) has been identified as a critical mechanism in eukaryotic evolution and is pervasive in both ancient and recent plant lineages [50]. Both synteny and *K*_S_*-*based methods have been widely used for inferring polyploidy, including identifying the occurrence of WGDs, their resulting ploidy levels, and the phylogenetic placement of these events [12, 51]. However, inter-species orthologous synteny patterns have been previously overlooked in polyploidy inference, potentially leading to misunderstandings regarding the evolutionary history of WGD events (see discussions in [52]). We argue that distinguishing orthologous synteny from out-paralogous synteny is not only straightforward but also vital for accurately inferring the evolutionary history of polyploidy. We illustrate a straightforward model to explain the process of polyploidy inference using the patterns of orthologous synteny (Fig. 3A). When two genomes exhibit a 2:2 ratio of synteny depth, there are two main hypotheses to consider: either the genomes share a tetraploidization event that occurred in their common ancestor (the ‘shared-WGD’ hypothesis), or they each underwent a lineage-specific tetraploidization event independently after they diverged (the ‘specific-WGDs’ hypothesis) (Fig. 3A). The *OI* offers a straightforward and visual method to test these two hypotheses by separating orthologous and out-paralogous synteny. If the genomes display a clear 1:1 orthology + 1:1 out-paralogy, similar to the Salicaceae case (Fig. 1D; ignoring the very ancient out-paralogy from the γ event), the shared-WGD hypothesis is supported (Fig. 3A, left panel). In contrast, if they exhibit a 2:2 orthology, then the specific-WGDs hypothesis is supported (Fig. 3A, right panel). Applying this method to 90 known cases (Supplementary Figs S1–S90) suggests that these inferences of polyploidy history based on orthologous synteny patterns are reasonable and accurate.

Apiaceae and Araliaceae (both within Apiales) provide an ideal test case for this. Previous synteny and *K*_S_*-*based analysis suggested either a shared WGD event in their common ancestor [53], or independent WGD events in the two families [54–56]. Therefore, we re-examined the polyploidy of available genomes from these two families using the newly developed *OI* method.

We first determined the occurrence of polyploidization and the resulting ploidy using orthologous synteny patterns and a summarized term, relative ploidy (*p*, i.e. the overall orthologous synteny depth relative to a given reference genome), for the Apiales genomes, referring to the *Vitis vinifera* (Vitaceae) genome, which has not undergone lineage-specific polyploidy since the paleohexaploidy γ event shared by core eudicots [49]. Specifically, *p*> 1 indicates one or more independent polyploidization events since the divergence from the reference genome, which resulted in a ploidy of 2*p* for a haploid genome assembly (e.g. *p =* 2 suggests tetraploidy and *p =* 3 suggests hexaploidy). Using *Centella asiatica* (Apiaceae) and *Aralia elata* (Araliaceae) as representatives, we observed a clear 2:1 orthologous synteny depth (*p =* 2) with the *V. vinifera* genome (Supplementary Fig. S93), suggesting that both species underwent a tetraploidization event following their divergence from Vitaceae, consistent with previous research [55, 56]. However, upon comparison of the two genomes, a clear 1:1 orthology (*p =* 1) + 1:1 out-paralogy pattern was highlighted by the *OI* (Fig. 3B), aligning with the first model (Fig. 3A, left panel) and thus supporting the hypothesis of shared WGD (ω, Fig. 3C) in their common ancestor (see Song *et al.* [53]). This inference was corroborated by macro-synteny phylogenies (Supplementary Fig. S94), demonstrating the reliability of the *OI* method. Similar patterns were also observed in other combinations of genomes from the two families (e.g. *C. asiatica*: *Eleutherococcus senticosus* = 1:2 orthology) (Supplementary Figs S95 and S96), further supporting the inference. Notably, we observed a considerable disparity in branch length (or substitution rate) between the two families from their most recent common ancestor (MRCA), with the mean branch length of Apiaceae (*L1* = 0.205) nearly three times that of Araliaceae (*L2*= 0.074) (Fig. 3C). This discrepancy could explain the unreasonable phylogenetic placements drawn from the traditional *K*_S_-based methods [54–56], which assumed equal substitution rates for different lineages when estimating the relative timing of polyploidization and speciation events. However, the assumption of equal substitution rates is often false or uncertain in practice. Even though the substitution rate could be properly corrected [21, 51], the results are likely to remain ambiguous, when Δ*T* is small and thus orthology and paralogy-derived *K*_S_ peaks overlap significantly (e.g. Fig. 2C).

Furthermore, using the *OI*-based approach, we deduced additional lineage-specific polyploidy events within the two families **(**Fig. 3C**)**. We observed that all studied genomes from species in the Apioideae subfamily (including *Daucus carota*, *Angelica sinensis*, *Apium graveolens*, and *Coriandrum sativum*) exhibit a 2:1 orthologous synteny depth (*p =* 2) when compared to the *C. asiatica* genome (Supplementary Fig. S97), suggesting that they each experienced a tetraploidization event subsequent to their divergence from *C. asiatica*. Moreover, any two genomes within subfamily Apioideae display distinct 1:1 orthology (*p =* 1) + 1:1 out-paralogy patterns (Supplementary Figs S56 and S98), suggesting a shared tetraploidization event (α). This finding is in line with previous research [53, 56] and places the α event between the stem and crown nodes of the Apioideae subfamily (Fig. 3C). In addition, employing the *OI*, we also inferred one species-specific tetraploidization event in each of the *Panax ginseng* and *E. senticosus* genomes (Fig. 3C and Supplementary Figs S57–S59 and S99). This is consistent with some previous research [55, 56] but conflicts with the inference of two independent WGD events in *P. ginseng* in Song *et al.* [53], which may have been misled by other types of gene duplications (non-WGD).

In summary, each previous inference for polyploidy in the Apiales genomes [53–56] was partly correct but did not capture the whole picture. The *OI* can now accurately unravel the history of polyploidy with strong evidence of orthologous synteny patterns. These findings suggest that misinterpretation is likely if inter-genomic orthologous synteny patterns are not considered, potentially leading to confusion with out-paralogs and other types of gene duplications. Traditional *K*_S_-based methods can be ambiguous and ineffective in identifying the occurrence and phylogenetic placement of WGDs [57], while traditional synteny-based methods likely fail to correctly place WGDs in a phylogenetic context when confounded by out-paralogous synteny. In contrast, orthologous synteny inferred using the *OI*, which integrates both synteny and orthology information, provides a straightforward, reliable and versatile method for identifying the occurrence of WGD events, determining the resulting ploidy, and accurately placing WGDs in a phylogenetic context. Nonetheless, when orthologous synteny patterns are not very clear, the phylogenetic placement should be confirmed using phylogeny-based methods. An automated function for macro-synteny phylogeny (Supplementary Fig. S94) is also provided in the SOI toolkit, which requires prior knowledge of relative ploidy inferred from the orthologous synteny patterns.

### Applications in identification of reticulation

**Figure 4.**
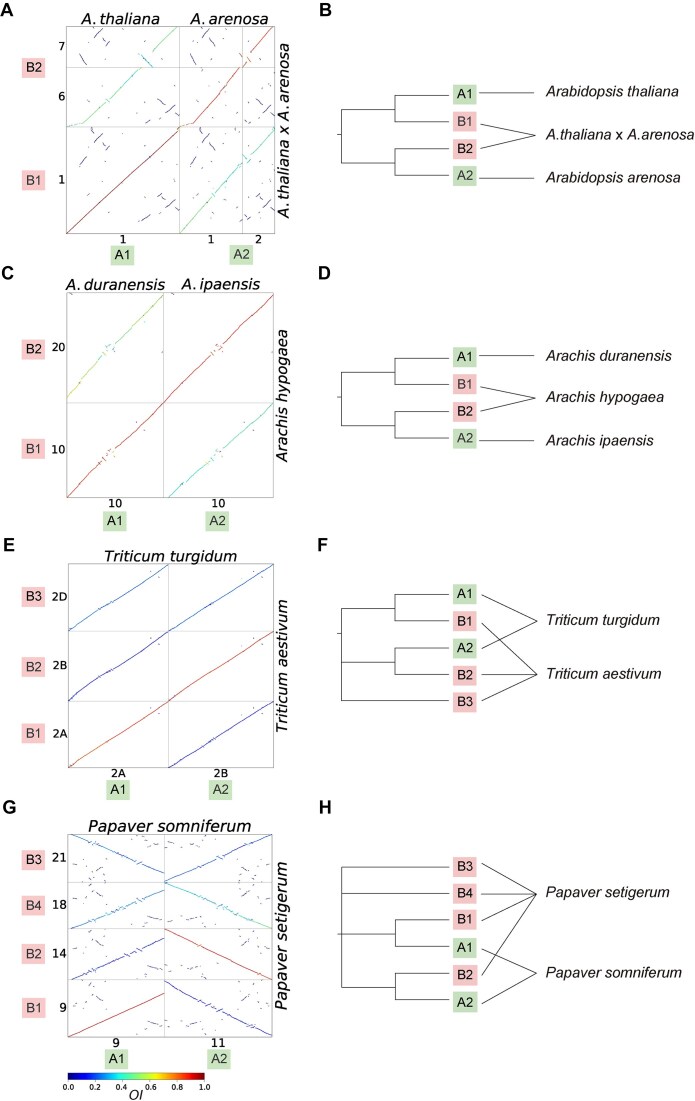
Examples of reticulation inferences based on the *OI*. (**A**, **B**) *OI*-colored dot plots (A) of the genomes of *A. thaliana* + *A. arenosa* and their hybrid, with the inference (B; in dendrogram form) based on the orthologous relationships. (**C**, **D**) *OI*-colored dot plots (C) of the genomes of the tretaploid *A. hypogaea* and its diploid progenitors, and the inference (D; in dendrogram form) based on the orthologous relationships. (**E**, **F**) *OI*-colored dot plots (E) of the genomes of the hexaploid *T. aestivum* and its intermediate tretaploid *T. turgidum*, with the inference (F; in dendrogram form) based on the orthologous relationships. (**G**, **H**) *OI*-colored dot plots (G) of the genomes of the neo-octoploid *P. setigerum* and its intermediate tretaploid *P. somniferum*, with the inference (H; in dendrogram form) based on the orthologous relationships. Only one set of representative homoeologous chromosomes is shown in the dot plots; dot plots with the full set of chromosomes can be found in Supplementary Figs S100–S103.

Reticulation, driven by hybridization and/or allopolyploidization, is a significant factor in eukaryotic evolution, yielding novel phenotypes that facilitate ecological diversification and the occupation of new niches [58]. Numerous genomes originating from recent reticulation events have been documented (summarized in Jia *et al.* [59]), encompassing critical cereals [60], fruits [61], vegetables [62], trees [18], and fish [63]. We evaluated the application of the *OI* in several representative, well-documented cases, ranging from simple to complex evolutionary scenarios (Fig. 4).

The cases of *Arabidopsis* [64] and *Arachis* [65] represent simple hybridization or allopolyploidization scenarios, respectively. From the *OI*-colored dot plots, the two subgenomes of both the hybrid (*A. thaliana* × *A. renosa*) and tetraploid (*Arachis hypogaea*) showed clear and separate orthologous relationships with their diploid progenitors (Fig. 4A and C, and Supplementary Figs S100 and S101). Based on the orthologous relationships revealed by the *OI*, the hybridization events can be easily inferred with the straightforward visualization (Fig. 4B and D).

The orthologous relationships between the genomes in the complicated polyploid species complexes *Triticum* and *Papaver* were also clear from the *OI*-colored dot plots (Fig. 4E and G, and Supplementary Figs S102 and S103). The two subgenomes of the tetraploid *T. turgidum* were orthologous to the two subgenomes of the hexaploid *T. aestivum* (Fig. 4E and Supplementary Fig. S102), supporting the inference that *T. turgidum* is the intermediate tetraploid progenitor of the allohexaploid *T. aestivum* (Fig. 4F), in line with previous results [60]. Similarly, a reticulate allopolyploidization origin in two *Papaver* genomes (Fig. 4H) can be inferred from the *OI*-colored dot plots (Fig. 4G and Supplementary Fig. S103). This inference agrees with our previous work [16]; however, the phylogenetic relationships between homoeologous subgenomes cannot be resolved directly from the *OI*-colored dot plots and require further evidence, such as chromosome or subgenome-scale phylogenies [16].

Therefore, we conclude that reticulate speciation with intact subgenomic structure can be simply and directly inferred from the *OI*-colored dot plots. Although phylogeny-based methods could provide a deeper insight into the reticulation [8], the *OI* method is more straightforward and allows for visualization that at least suggests a hypothesis to test further. Indeed, the straightforward visualization of the patterns of orthologous synteny can make it easier to recognize the signals of reticulation and thus reasonably elucidate the evolutionary history (e.g. [15–17]).

### Applications in phylogenomics

Accurate inference of orthology plays a crucial role in the estimation of species trees, and pseudo-orthologs (i.e. hidden paralogs) derived from WGD and gene loss can greatly mislead species tree inference under some circumstances [66]. As demonstrated above (Figs 1 and 2, and Supplementary Figs S1–S90), the *OI* exhibited a high level of accuracy in identifying syntenic orthologs and can therefore minimize the confounding influence of pseudo-orthologs. Consequently, the resulting syntenic orthologs can be directly applied to species tree reconstruction. We used the core eudicots as an example to showcase this application. It is accepted that all core eudicots share a paleohexaploidization event (γ event, WGT) around 120 Mya, whereas no two orders within the core eudicots share an additional polyploidy event [50]. Therefore, it is appropriate to use the *OI* to remove the out-paralogy produced from the γ event, and to better resolve the phylogenetic relationships among the orders of the core eudicots, many of which remain poorly resolved, such as the Celastrales–Oxalidales–Malpighiales (COM) clade [67]. Here we utilized the genome-scale syntenic orthologs inferred by the *OI* to reconstruct a backbone phylogeny of the core eudicots, aiming to minimize the confounding influence of the γ event.

**Figure 5.**
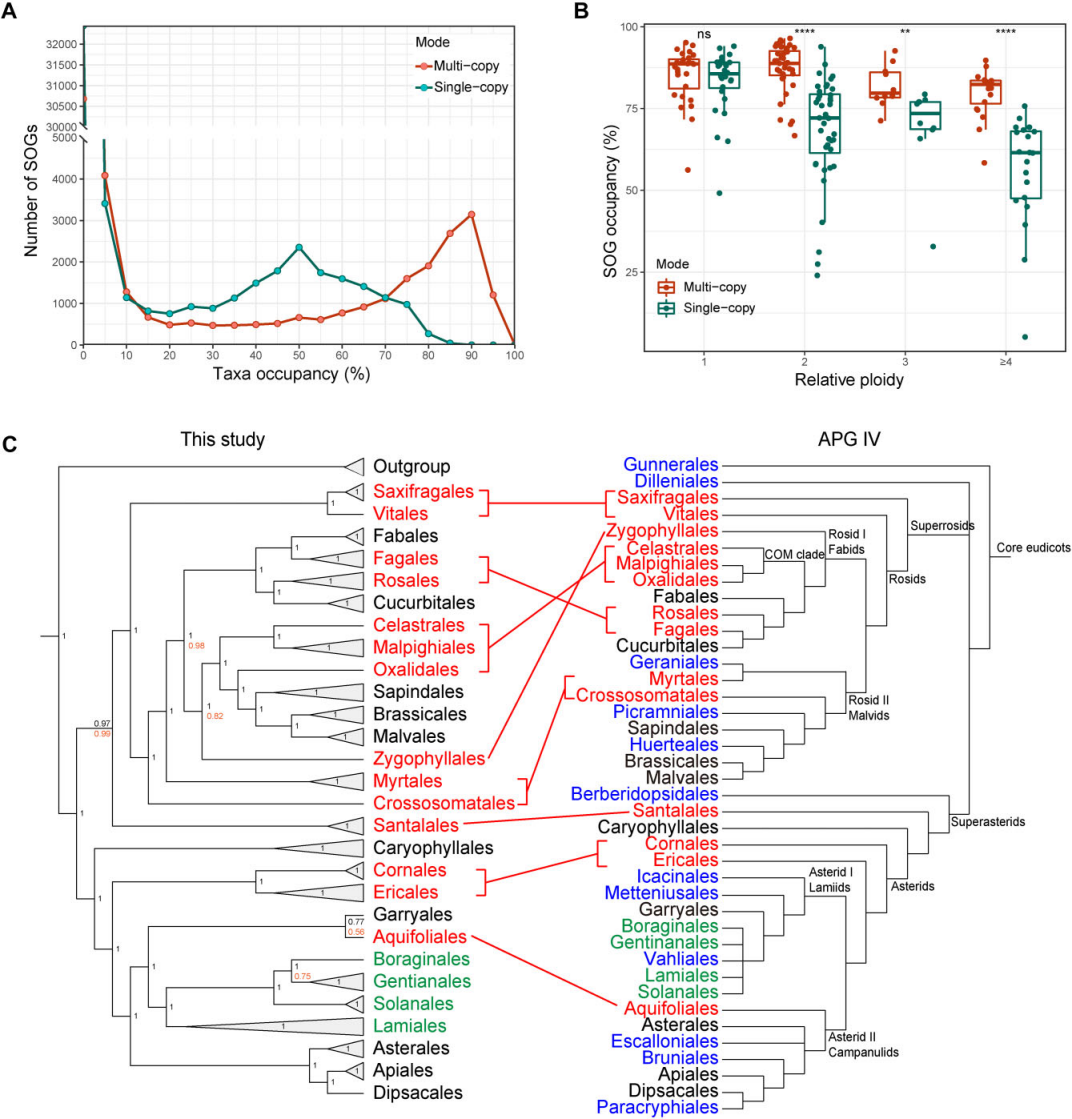
An example (core eudicots) of phylogenomics based on the *OI*. (**A**) The number of multi-copy and single-copy SOGs with different taxon occupancy. (**B**) The occupancy of SOGs in species with different relative ploidy (i.e. orthologous syntenic depth relative to the *V. vinifera* genome), allowing up to 40% taxa missing. Each point represents one species. ns, *P* > .05; **, *P* ≤ .01; ****, *P* ≤ .0001; Wilcoxon test. (**C**) Comparison of phylogenetic relationships within the core eudicots reconstructed in this study versus those in APG IV. Conflicting positions are marked in red; unresolved relationships in APG IV are marked in green, and orders not covered in this study are marked in blue. The numbers at the nodes are posterior probabilities from ASTRAL, with the black representing values from the multi-copy SOGs and orange representing values from the single-copy SOGs (omitted for equal values). Further details of the two trees reconstructed in this study can be found in Supplementary Figs S104 and S105.

We compiled a high-quality genomic dataset that covers 28 (70%) of the 40 orders and 98 (33%) of the 298 families of core eudicots treated in APG IV [67]. We then applied the *OI* to this dataset to identify syntenic orthologs, resulting in the identification of 54 322 SOGs. After filtering, 12 277 multi-copy and 5 154 single-copy SOGs were retrieved, allowing for up to 40% missing taxa (Fig. 5A). This imbalance between the numbers of multi-copy and single-copy SOGs is attributable to lineage-specific polyploidy events within the core eudicots. As a result of these polyploidy events, the occupancy of single-copy SOGs showed significant decreases in species with high relative ploidy (i.e. orthologous syntenic depth relative to the *V. vinifera* genome) (Fig 5B). Nevertheless, the order-level species tree topologies based on the two gene sets were identical and both were strongly supported with high posterior probabilities (Fig. 5C**)**, although the tree based on multi-copy SOGs was more robust with equal or higher posterior probabilities at nearly all nodes (Fig. 5C), and there were slight differences in the positions of a few species (Supplementary Figs S104 and S105).

We observed large incongruences between our phylogeny and the APG IV [67], as nearly half of the orders covered in this study had inconsistent phylogenetic positions (Fig. 5C). For example, in our analysis, the COM clade was not monophyletic and was placed with the malvids, in contrast to that from APG IV (Fig. 5C). The incongruences are likely due to the fact that the APG IV analysis was conducted using a very limited number of phylogenetic markers [67]. In contrast, recent phylogenomics/phylotranscriptomic studies are consistent with most of our findings [68–75]. For example, the phylogenetic positions of the Fagales, Rosales, Celastrales–Oxalidales–Malpighiales, Myrtales, Cornales, and Ericales in our study (Fig. 5C) are consistent with the genome-scale phylogenomics based on coalescent-based analysis of 482 single-copy nuclear orthologous sequences [72]. In addition, the phylogenetic positions of the Santalales (sister to the superrosids), Aquifoliales (sister to the Garryales), and Crossosomatales (sister to the fabids + remaining malvids) in our study (Fig. 5C) are consistent with the phylogenetic inference based on coalescent tree analysis of 410 single-copy gene families extracted from transcriptome and genome data [69].

Our results further provide interesting phylogenomic insights into the core eudicots. In our study, the Vitales was found to be sister to the Saxifragales with high support, and this clade was sister to the remaining rosids (Fig. 5C). The finding is inconsistent with the hypothesis that the Vitales are sister to the Saxifragales plus all other taxa in the superrosids clade [68–70, 72, 73, 75]. We also found that the Zygophyllales were sister to the malvids clade with high support (Fig. 5C), inconsistent with its placement as sister of the Myrtales [69, 73, 75]. These discrepancies can be mainly attributed to incomplete lineage sorting and ancient hybridization [76]. Given that our analyses involved ten thousand syntenic orthologous gene families, many more than the hundreds or thousands of loci used in previous studies [68–75], and considering that we minimized the confounding effects of the shared γ event, our results likely represent a more accurate depiction of the real tree of life of the core eudicots than those from other studies. Previous studies have used ortholog-based methods, which are prone to misidentify out-paralogs as orthologs, and we argue that our orthologous synteny-based method should be emphasized in plant phylogenomic studies. Nevertheless, our analysis is limited by taxon sampling (lack of high-quality genomes for some orders and families). However, this is expected to be resolved in the near future with ongoing developments and efforts in genome sequencing [77].

### Limitations

The *OI* may not perform well in extremely complex cases. For instance, when Δ*T* is notably small (e.g. speciation following polyploidization within a few generations), similar to other methods (e.g. *K*_S_*-*based), *OI* may also find it difficult to distinguish between out-paralogy and orthology (for a difficult case also see Supplementary Fig. S68). Therefore, although we set a unified *OI* cutoff (0.6) in our pipeline, users should manually inspect the results (mainly dot plots) for confirmation, and the extremely complex cases showing unexpected patterns should be investigated on a case-by-case basis. This method is also limited in some scenarios where orthology inference and/or synteny detection is limited. For example, synteny is known to be non-conserved in distantly-related or fast-evolving lineages [4], suggesting that this method should not be applied in such cases (e.g. angiosperms–ferns). Additionally, fragmented assemblies and mis-assemblies, as well as chromosome evolution, can disrupt synteny and subsequently reduce the efficiency of the method. However, the assembly issues are likely to cease to be a concern with the fast development of sequencing and assembly techniques. To address the reduced sensitivity of orthology inference due to disrupted synteny by factors such as chromosome rearrangements (e.g. Fig. 2F), we can use the highly reliable syntenic orthologs to train machine learning models to retrieve non-syntenic orthologs. This approach might significantly improve the recall of *OI* method in orthology inference. Finally, while our method currently supports the clustering of orthogroups across multiple species, it does not yet support the reconstruction of hierarchical orthologous groups [78]. This is also an area for future improvement.

## Conclusions

In summary, we present a human-interpretable and machine-actionable approach to distinguish orthologous synteny from out-paralogous synteny. This approach can identify orthologous synteny robustly, as validated with nearly 100 representative empirical cases and simulation-based benchmarks. Another major advantage is that this approach generally does not require pre-set parameters specific to the species pair in question, making it highly scalable and adaptable to diverse datasets. We have demonstrated the broad and valuable applications of this approach to the reconstruction of evolutionary history in plant genomes, including the reconstruction of the tree/network of life, and the identification and placement of polyploidy events on the tree/network. This approach will facilitate accurate and efficient analyses in evolutionary genomics and may reduce the misleading data generated using some traditional methods.

## Supplementary Material

gkaf320_Supplemental_File

## Data Availability

The codes of the SOI toolkit and pipeline, and typical examples can be found on GitHub (https://github.com/zhangrengang/SOI and https://github.com/zhangrengang/evolution_example). The source code has also been deposited to Zenodo (https://doi.org/10.5281/zenodo.14835296). The codon alignments and gene trees of the core eudicots are available from Figshare (https://doi.org/10.6084/m9.figshare.24174930).
