# Supplementary material for: SOI: robust identification of orthologous synteny with the Orthology Index and broad applications in evolutionary genomics
Source: Nucleic Acids Res. 2025 Apr 18;53(7):gkaf320. doi: 10.1093/nar/gkaf320 (PMC12006799; doi:10.1093/nar/gkaf320)
Supplement: gkaf320_Supplemental_File [file gkaf320_supplemental_file.pdf]

Supplementary Materials for

SOI: Robust identification of orthologous synteny with the *Orthology Index* and broad applications in evolutionary genomics

Ren-Gang Zhang *et al.*

Corresponding emails: mayongpeng@mail.kib.ac.cn; yves.vandepeer@psb.vib-ugent.be; kaihuajia_saas@163.com

This PDF file includes:

Figures S1 to S105
Tables S1 to S2
References (S1 to S204)

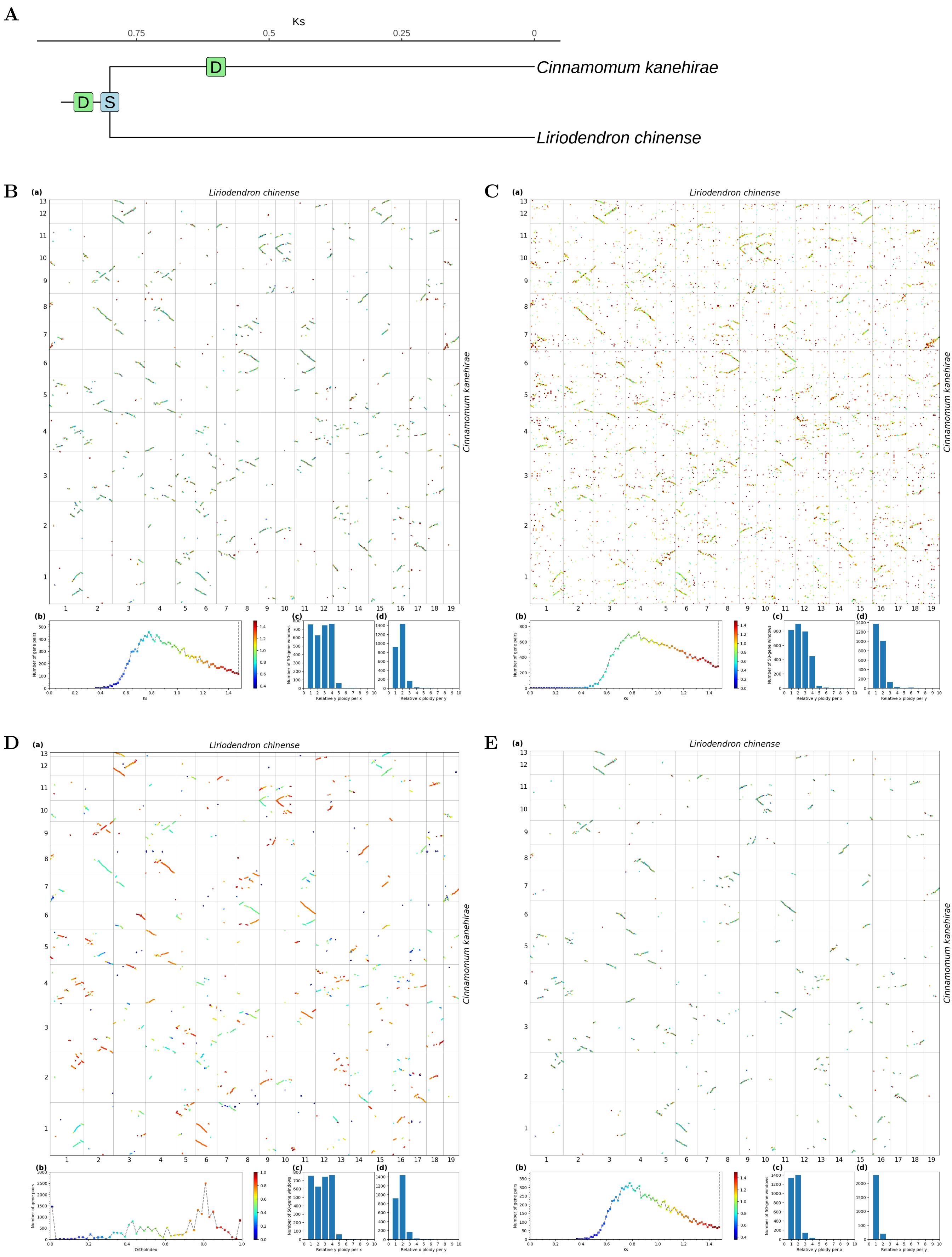

Figure S1. *Orthology Index* in the identification of orthologous synteny in *Liriodendron chinense* and *Cinnamomum kanehirae*. Refer to **Fig.1** for detailed descriptions.

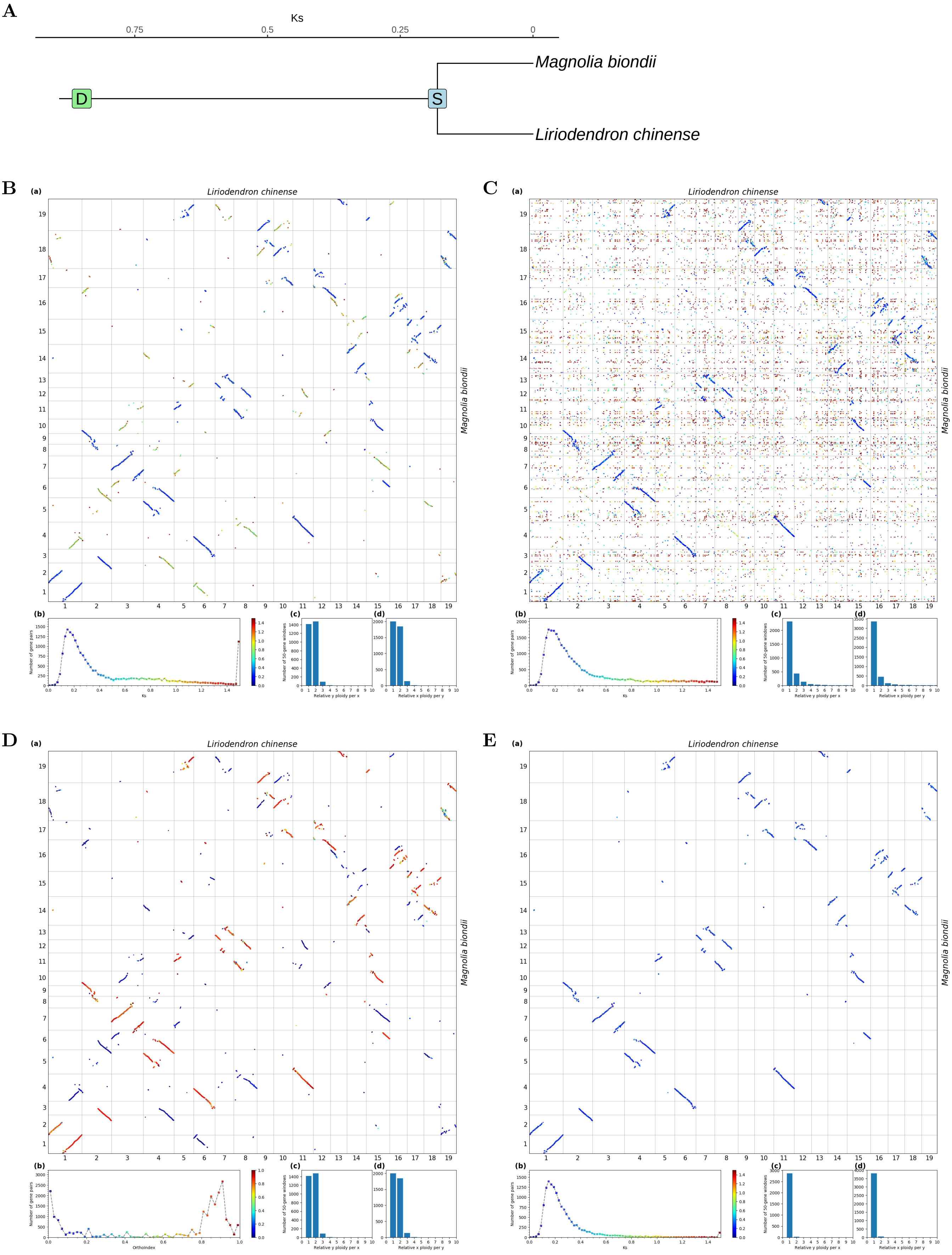

Figure S2. *Orthology Index* in the identification of orthologous synteny in *Liriodendron chinense* and *Magnolia biondii*. Refer to **Fig.1** for detailed descriptions.

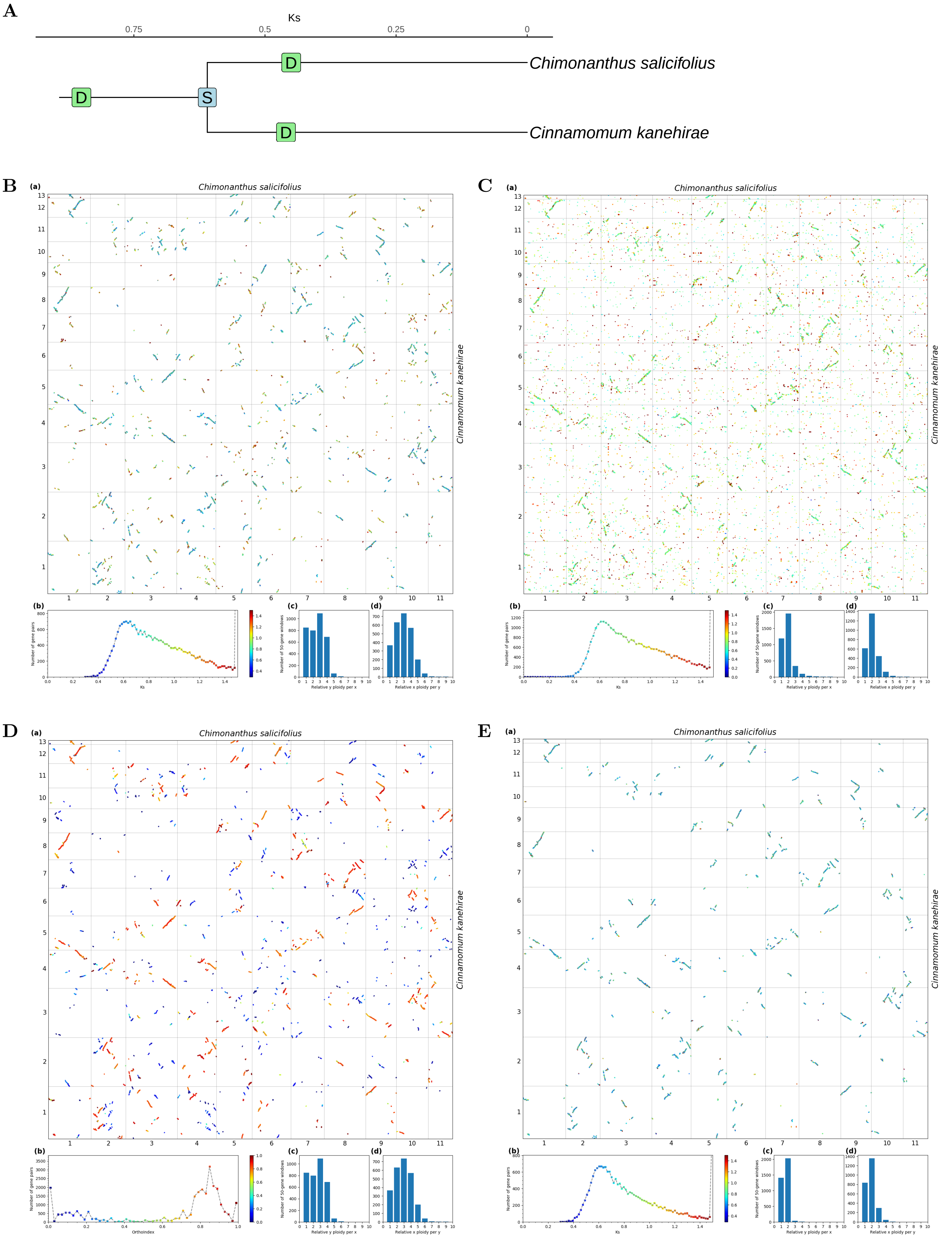

Figure S3. *Orthology Index* in the identification of orthologous synteny in *Cinnamomum kanehirae* and *Chimonanthus salicifolius*. Refer to **Fig.1** for detailed descriptions.

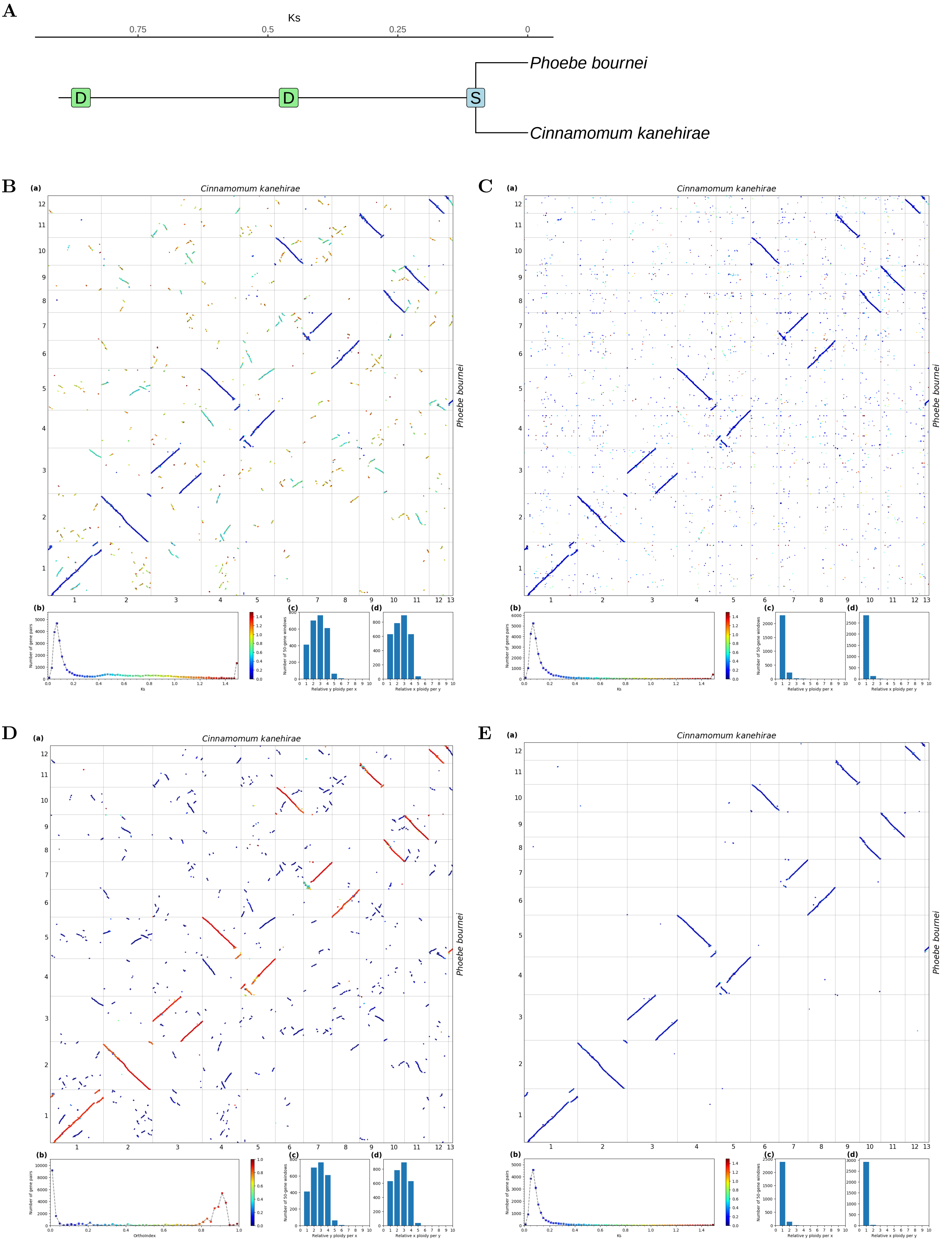

Figure S4. *Orthology Index* in the identification of orthologous synteny in *Cinnamomum kanehirae* and *Phoebe bournei*. Refer to **Fig.1** for detailed descriptions.

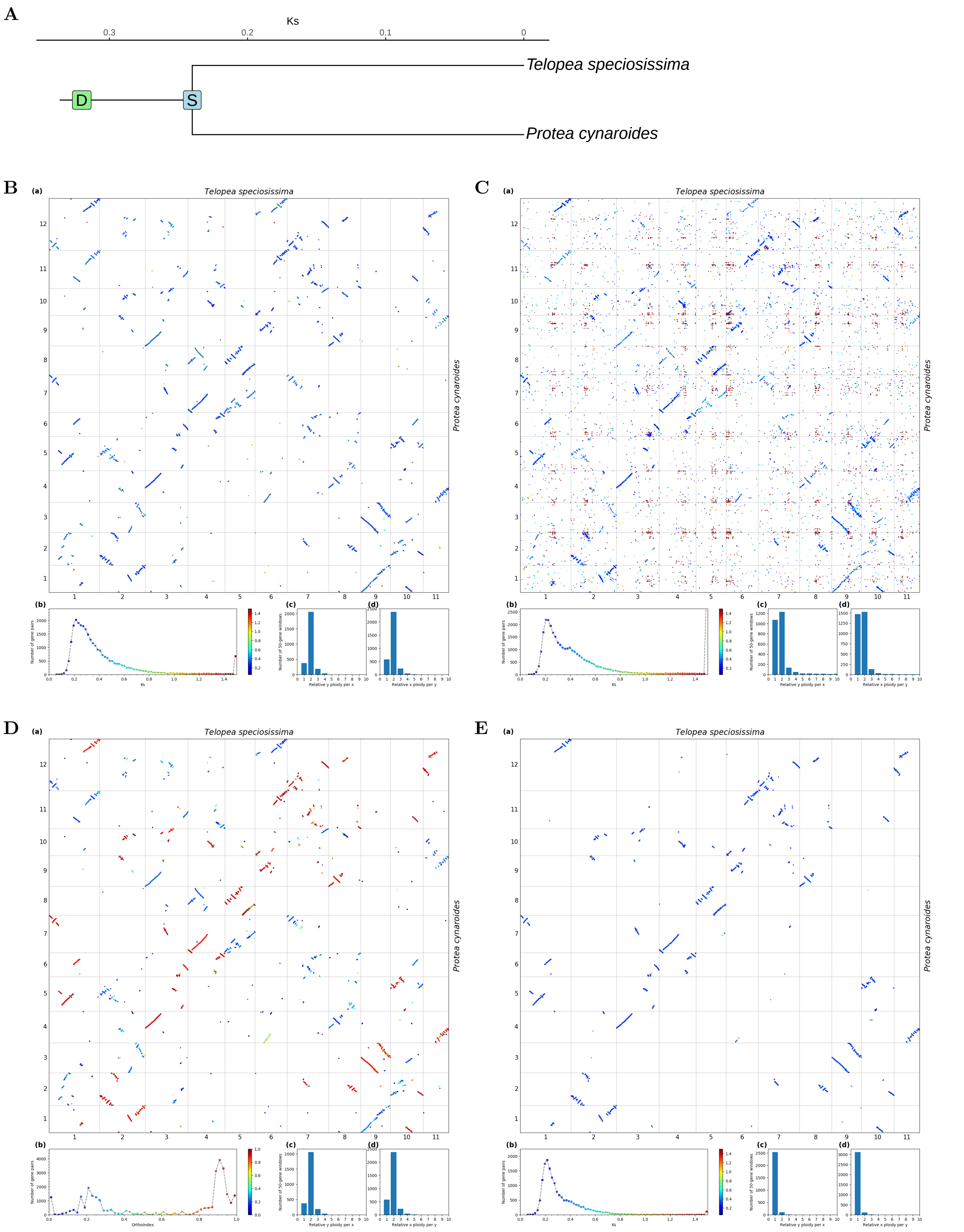

Figure S5. *Orthology Index* in the identification of orthologous synteny in *Protea cynaroides* and *Telopea speciosissima*. Refer to **Fig.1** for detailed descriptions.

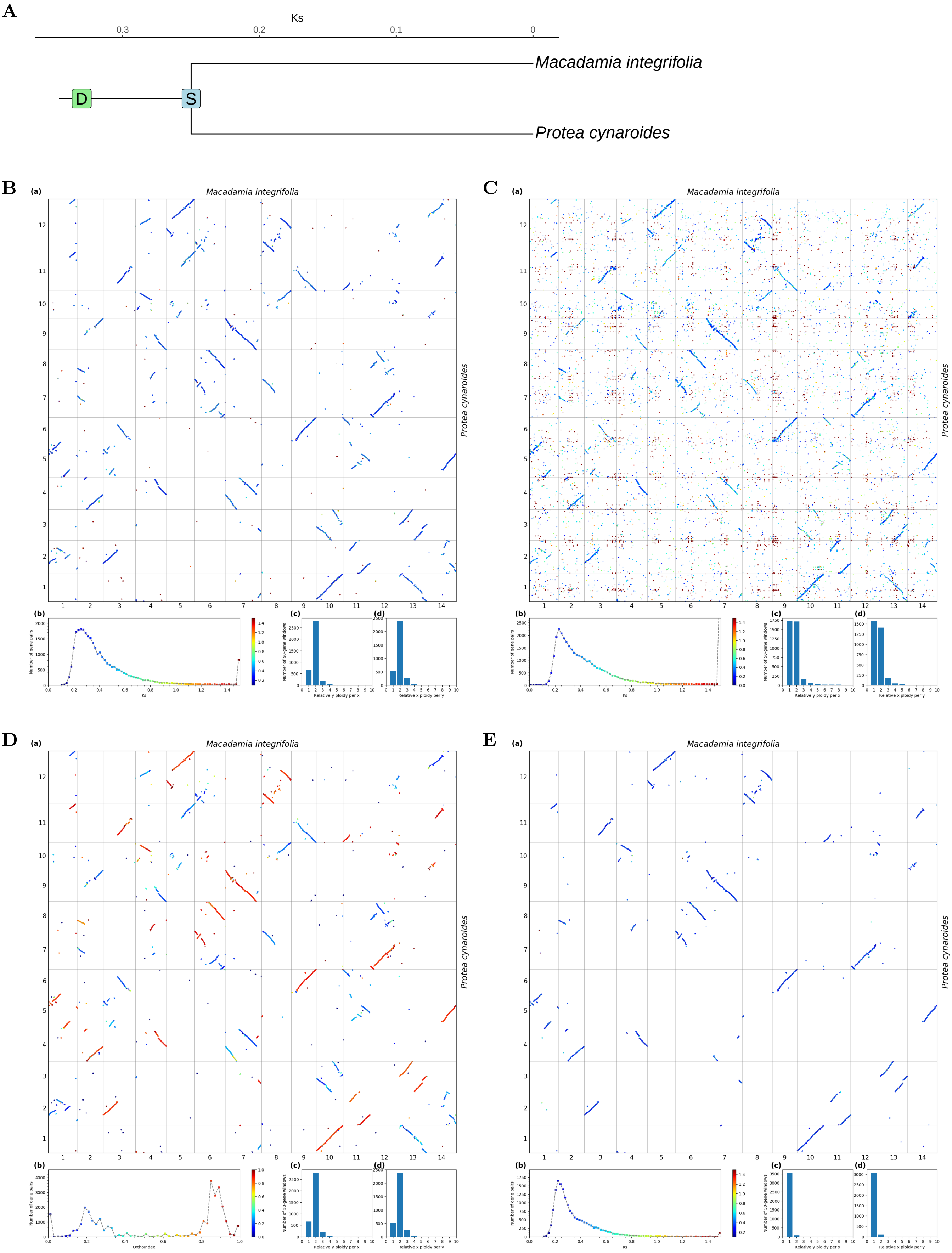

Figure S6. *Orthology Index* in the identification of orthologous synteny in *Protea cynaroides* and *Macadamia integrifolia*. Refer to **Fig.1** for detailed descriptions.

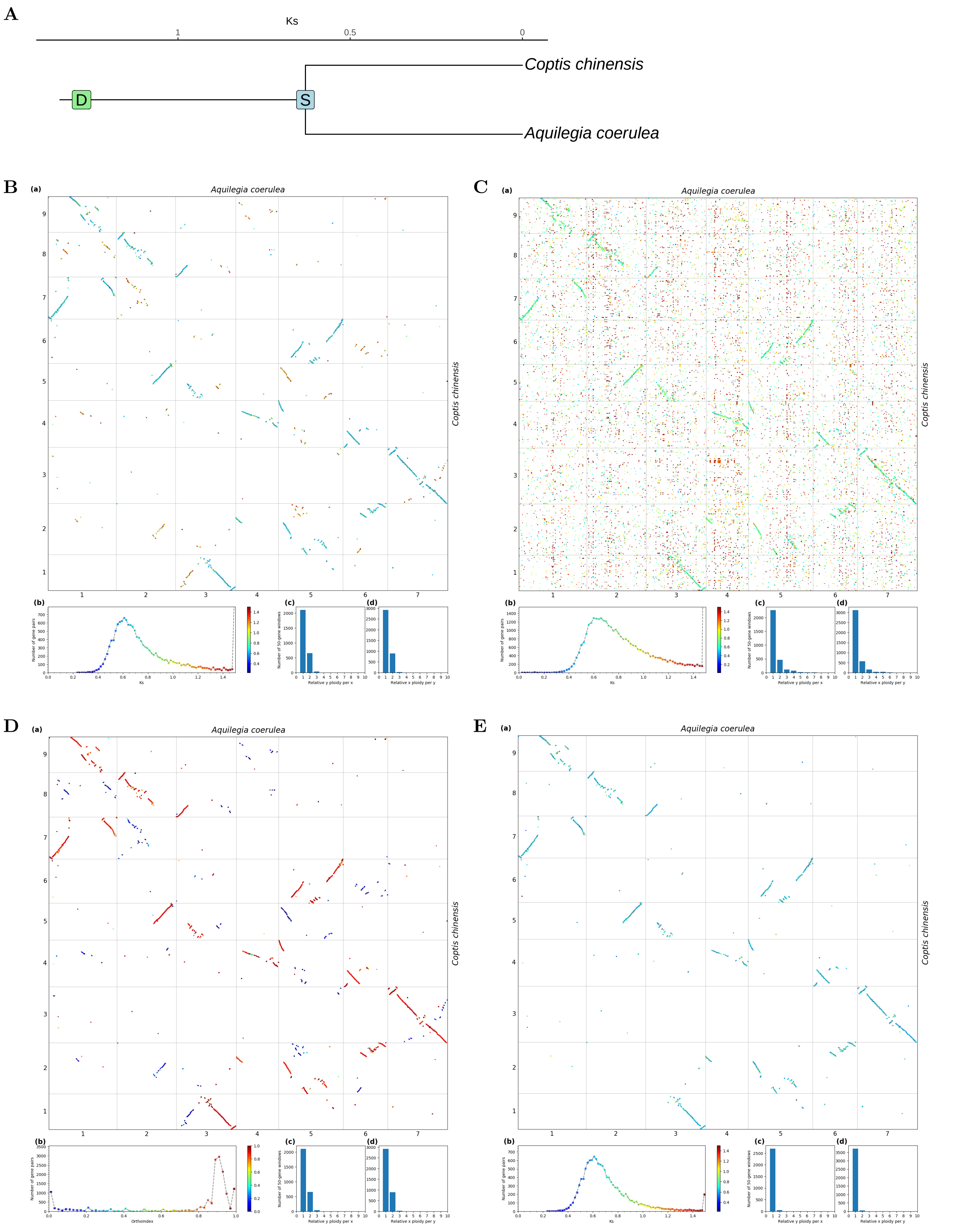

Figure S7. *Orthology Index* in the identification of orthologous synteny in *Aquilegia coerulea* and *Coptis chinensis*. Refer to **Fig.1** for detailed descriptions.

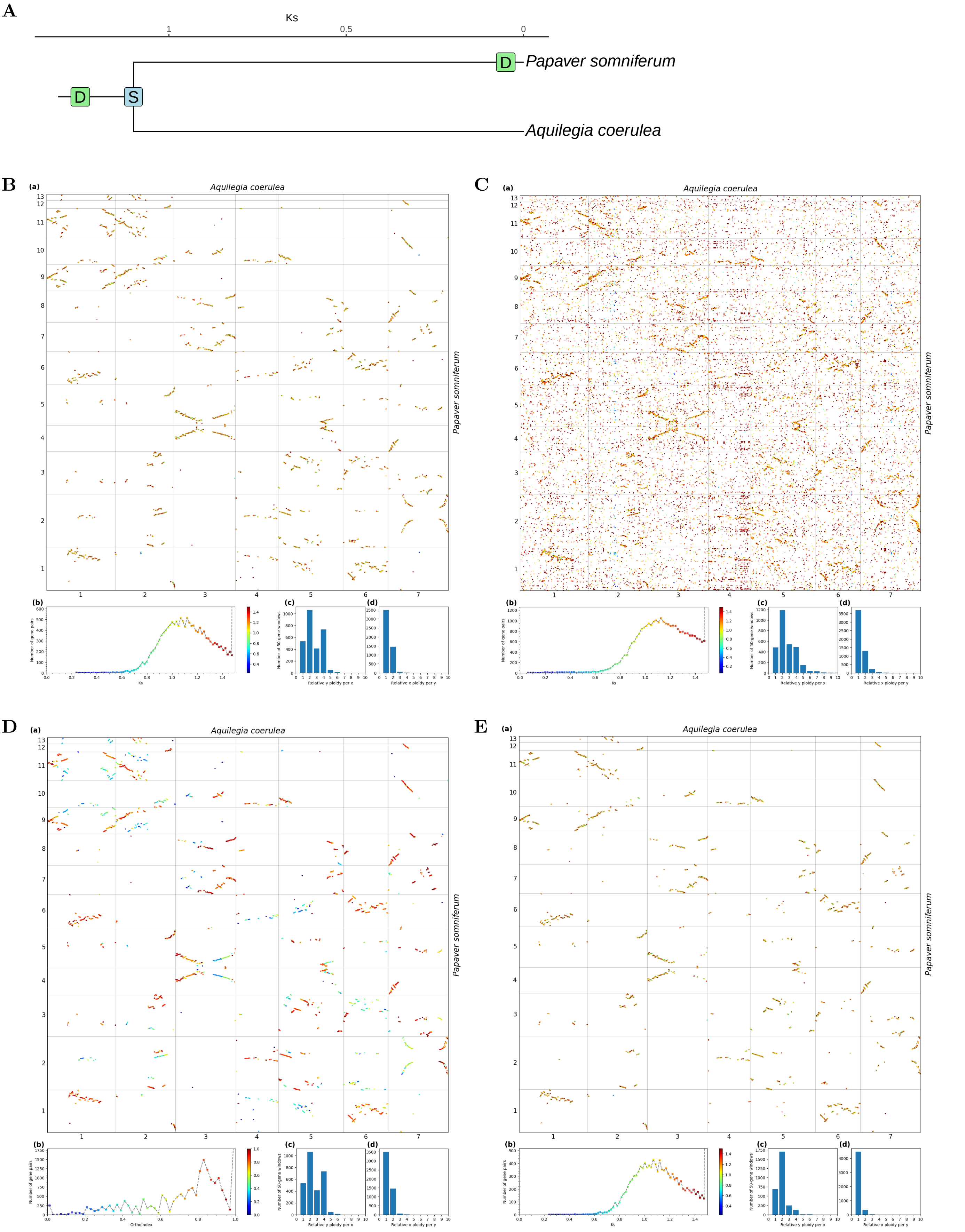

Figure S8. *Orthology Index* in the identification of orthologous synteny in *Aquilegia coerulea* and *Papaver somniferum*. Refer to **Fig.1** for detailed descriptions.

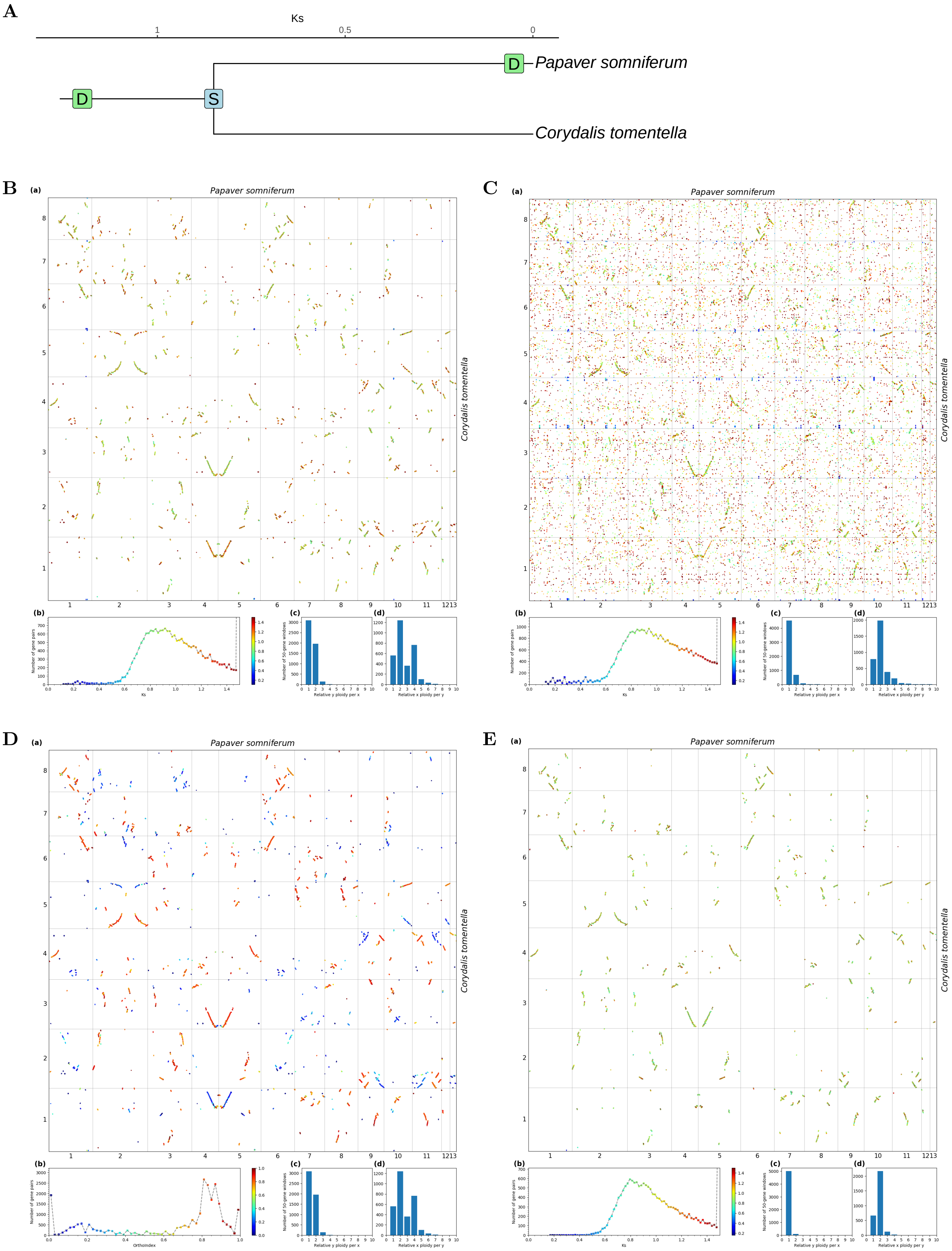

Figure S9. *Orthology Index* in the identification of orthologous synteny in *Corydalis tomentella* and *Papaver somniferum.* Refer to **Fig.1** for detailed descriptions.

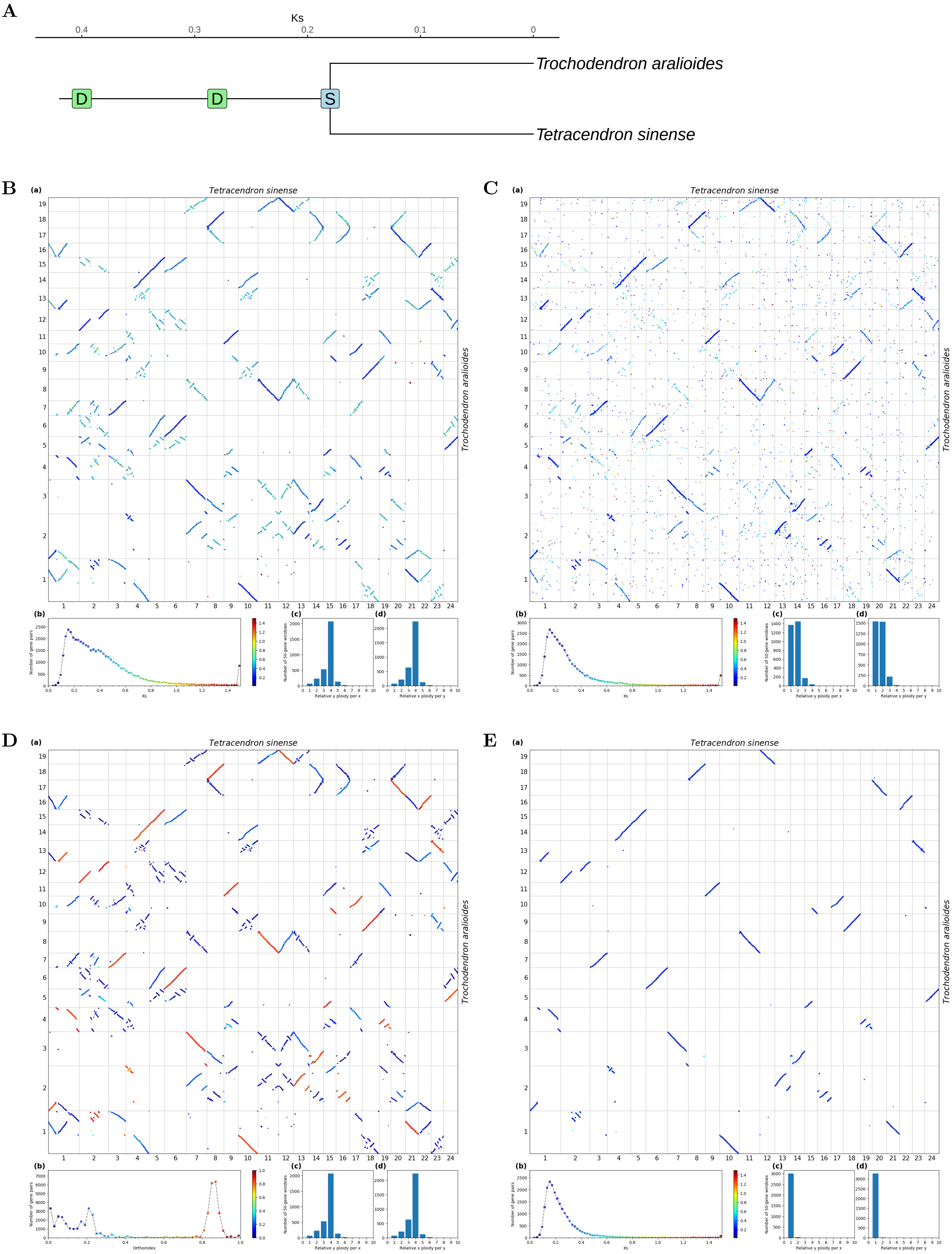

Figure S10. *Orthology Index* in the identification of orthologous synteny in *Tetracendron sinense* and *Trochodendron aralioides*. Refer to **Fig.1** for detailed descriptions.

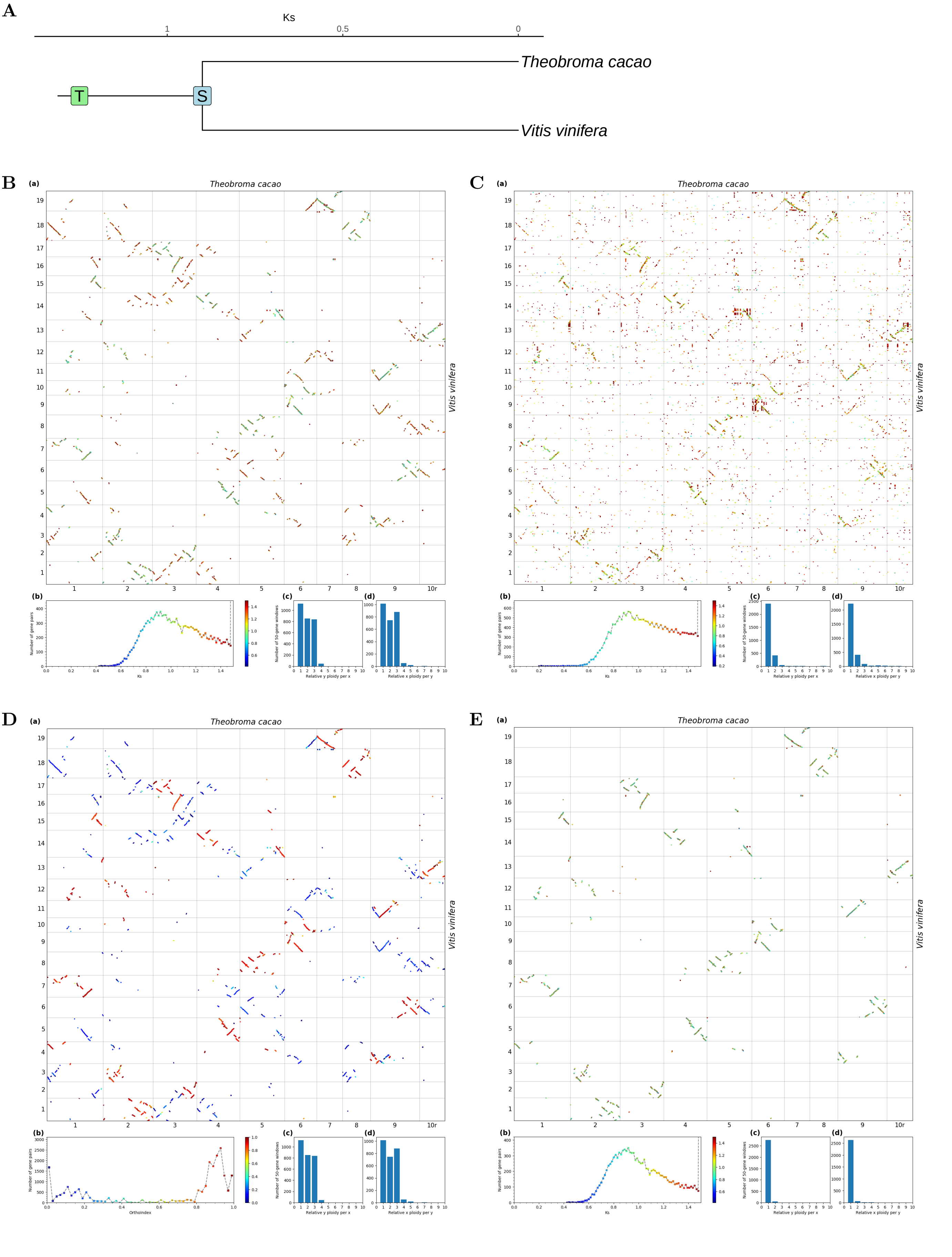

Figure S11. *Orthology Index* in the identification of orthologous synteny in *Vitis vinifera* and *Theobroma cacao*. Refer to **Fig.1** for detailed descriptions.

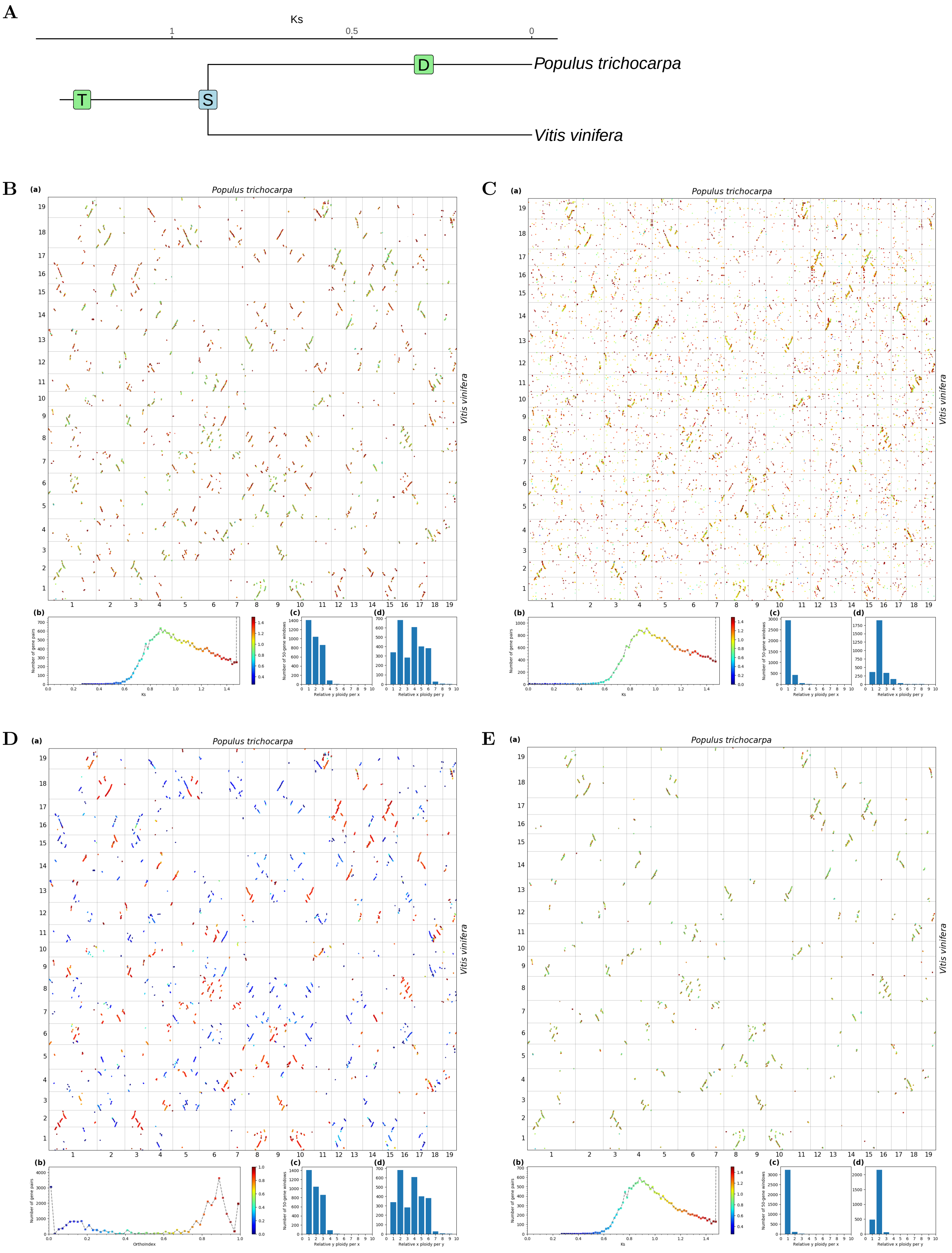

Figure S12. *Orthology Index* in the identification of orthologous synteny in *Vitis vinifera* and *Populus trichocarpa*. Refer to **Fig.1** for detailed descriptions.

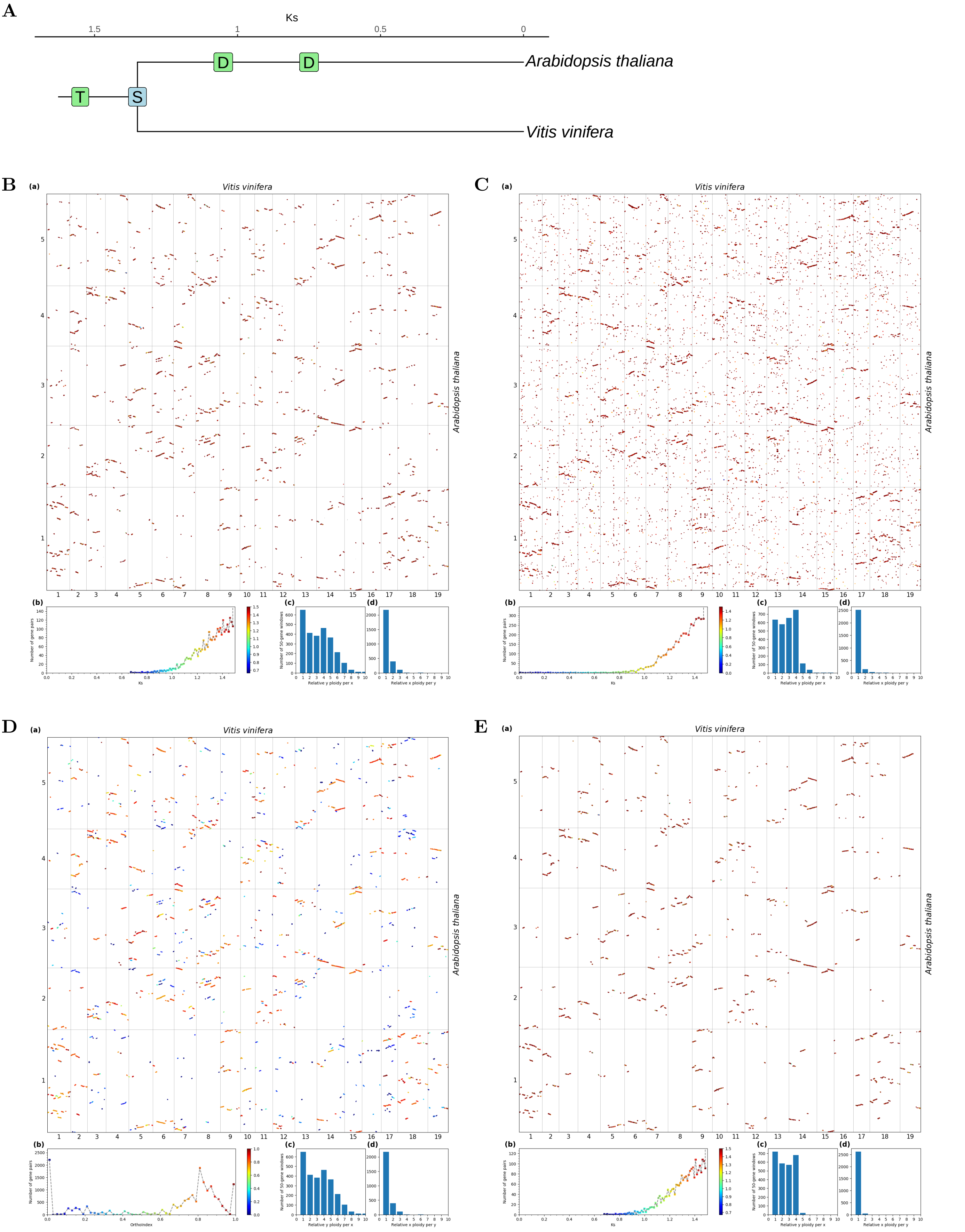

Figure S13. *Orthology Index* in the identification of orthologous synteny in *Vitis vinifera* and *Arabidopsis thaliana*. Refer to **Fig.1** for detailed descriptions.

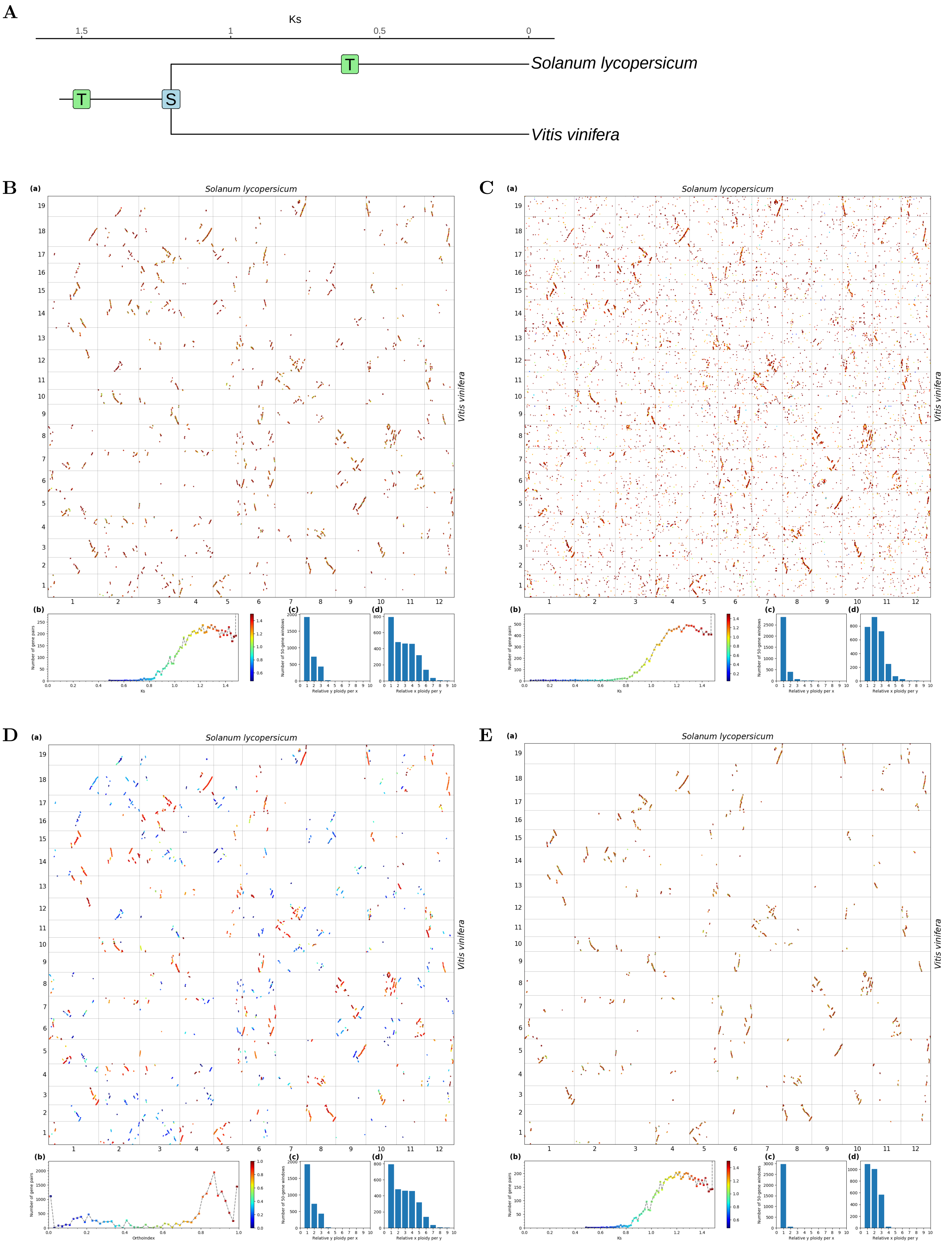

Figure S14. *Orthology Index* in the identification of orthologous synteny in *Vitis vinifera* and *Solanum lycopersicum*. Refer to **Fig.1** for detailed descriptions.

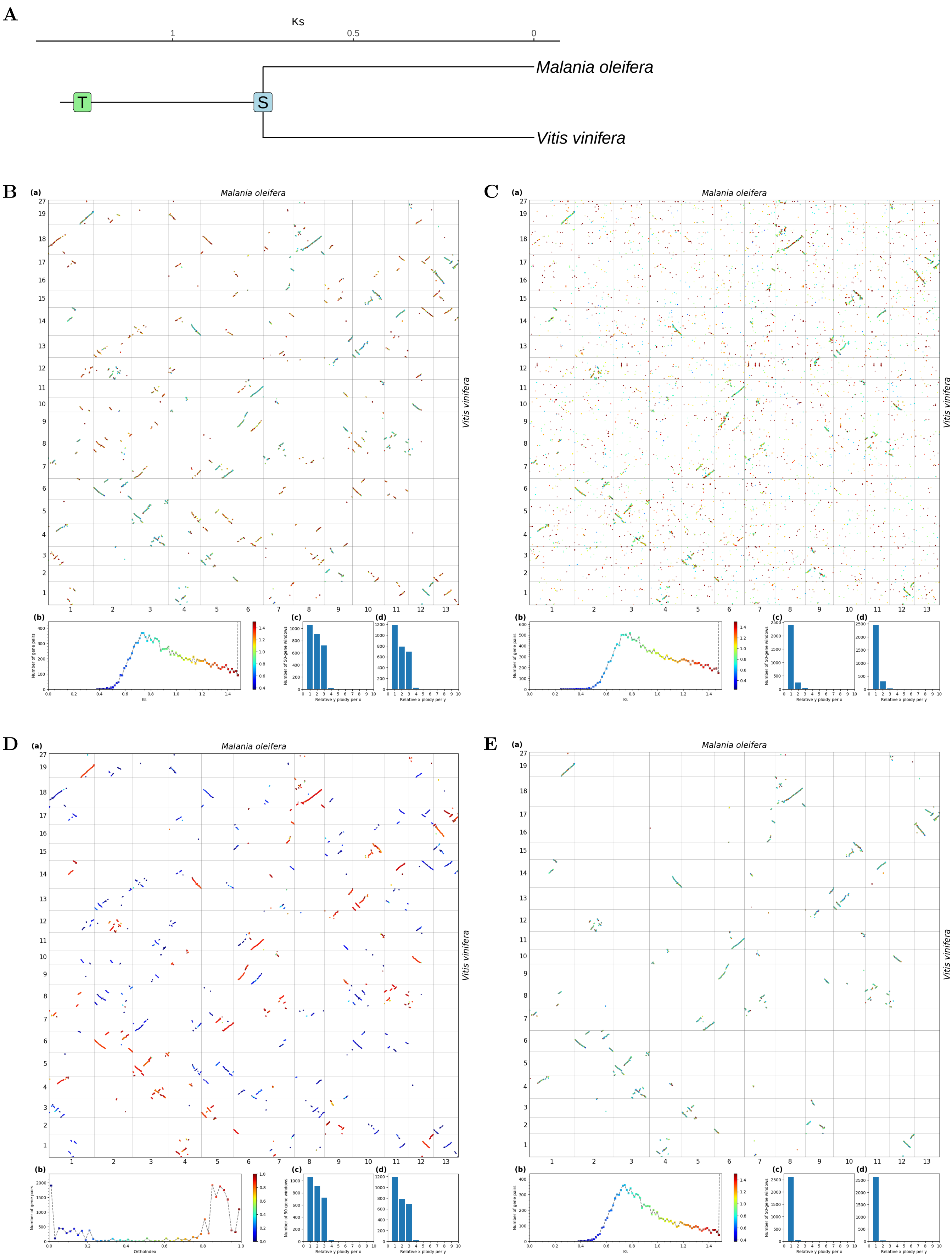

Figure S15. *Orthology Index* in the identification of orthologous synteny in *Vitis vinifera* and *Malania oleifera*. Refer to **Fig.1** for detailed descriptions.

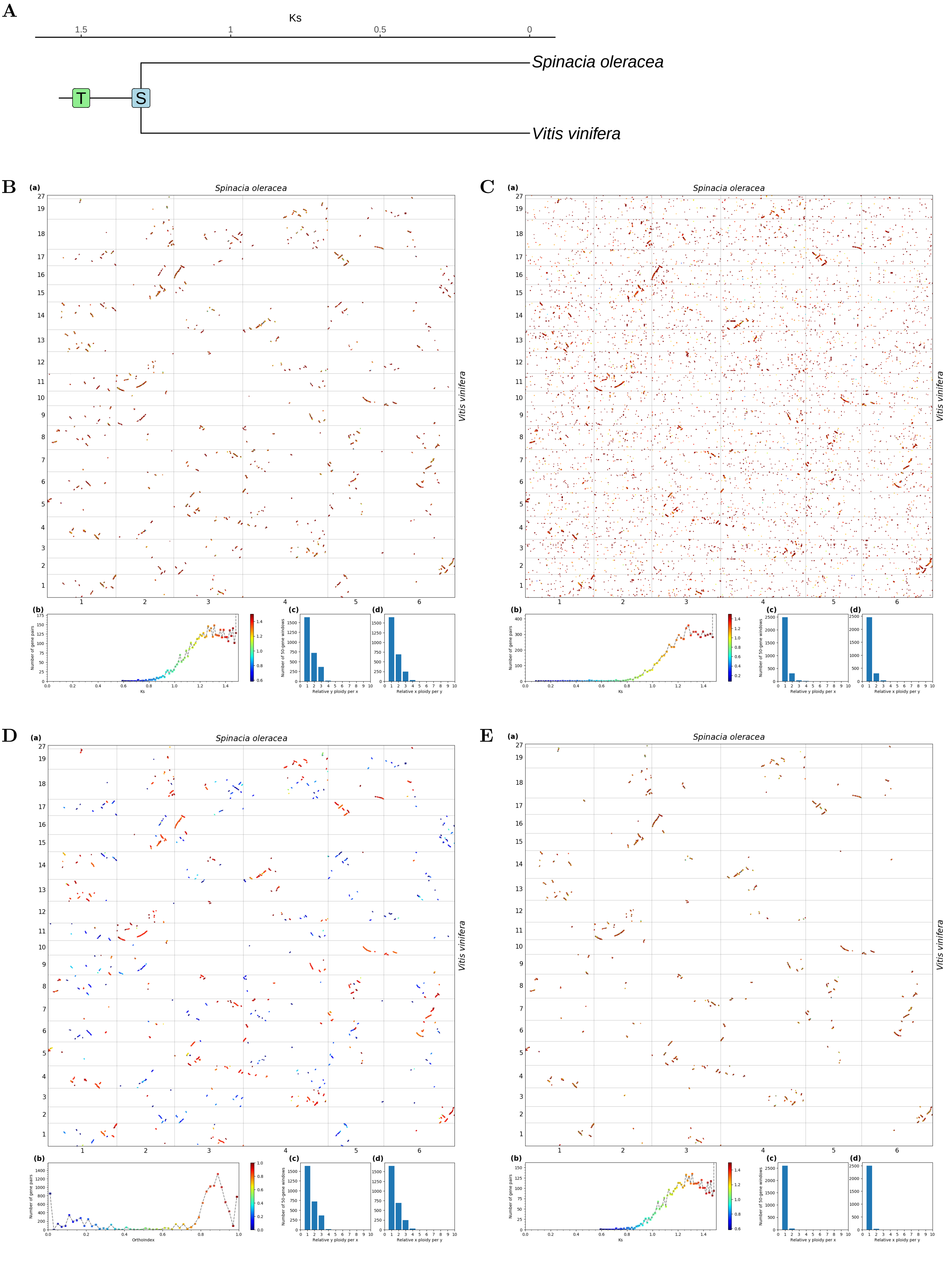

Figure S16. *Orthology Index* in the identification of orthologous synteny in *Vitis vinifera* and *Spinacia oleracea*. Refer to **Fig.1** for detailed descriptions.

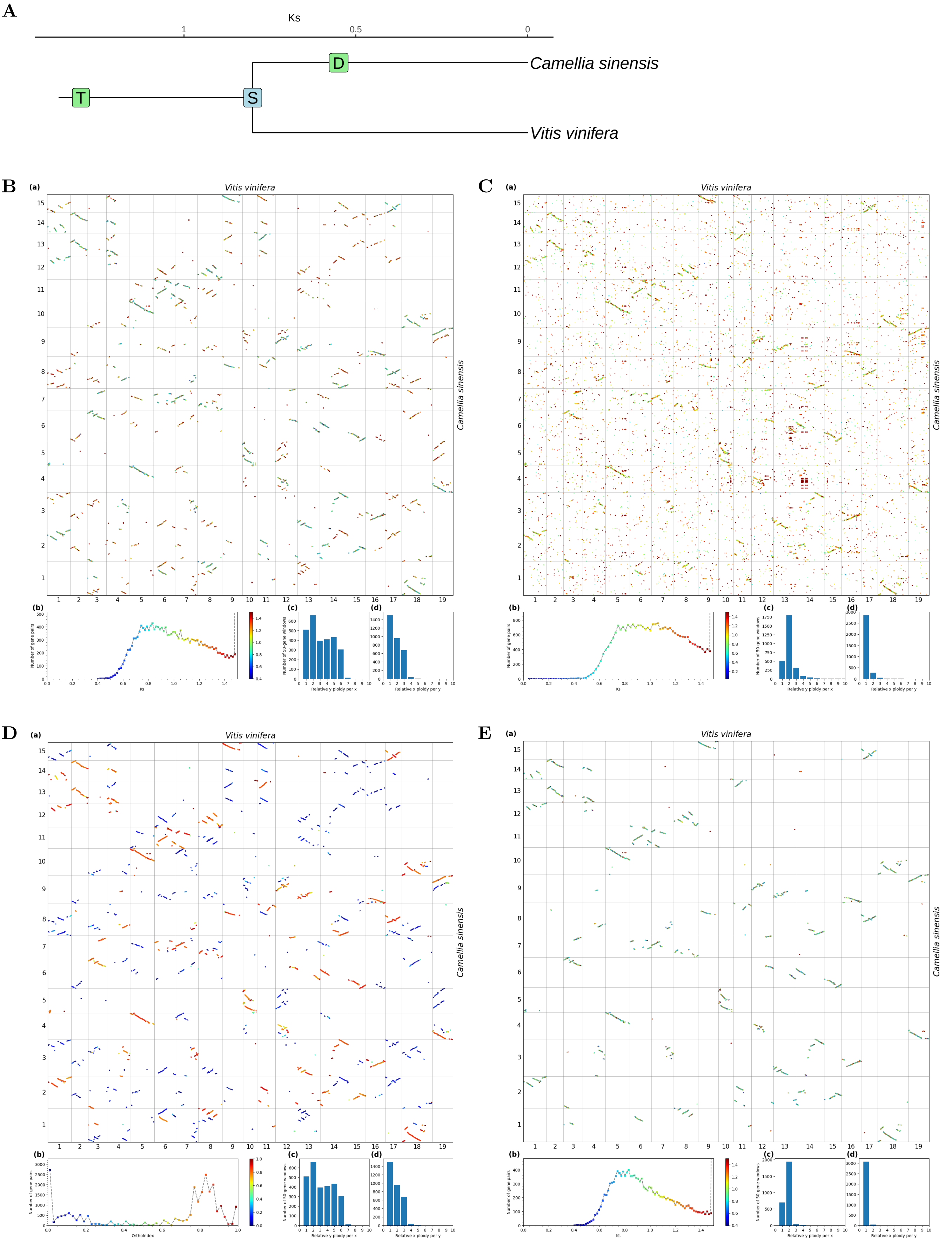

Figure S17. *Orthology Index* in the identification of orthologous synteny in *Vitis vinifera* and *Camellia sinensis*. Refer to **Fig.1** for detailed descriptions.

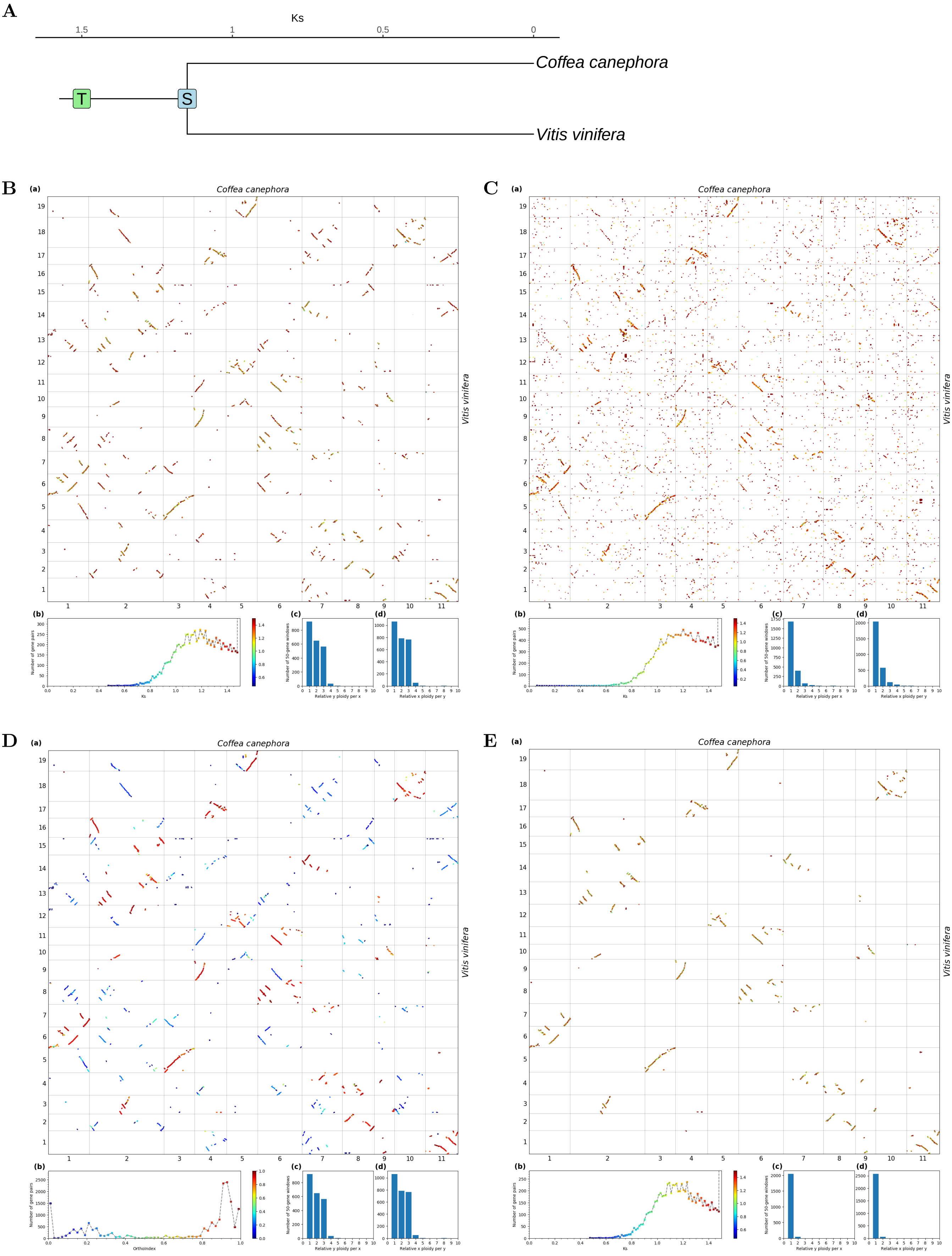

Figure S18. *Orthology Index* in the identification of orthologous synteny in *Vitis vinifera* and *Coffea canephora*. Refer to **Fig.1** for detailed descriptions.

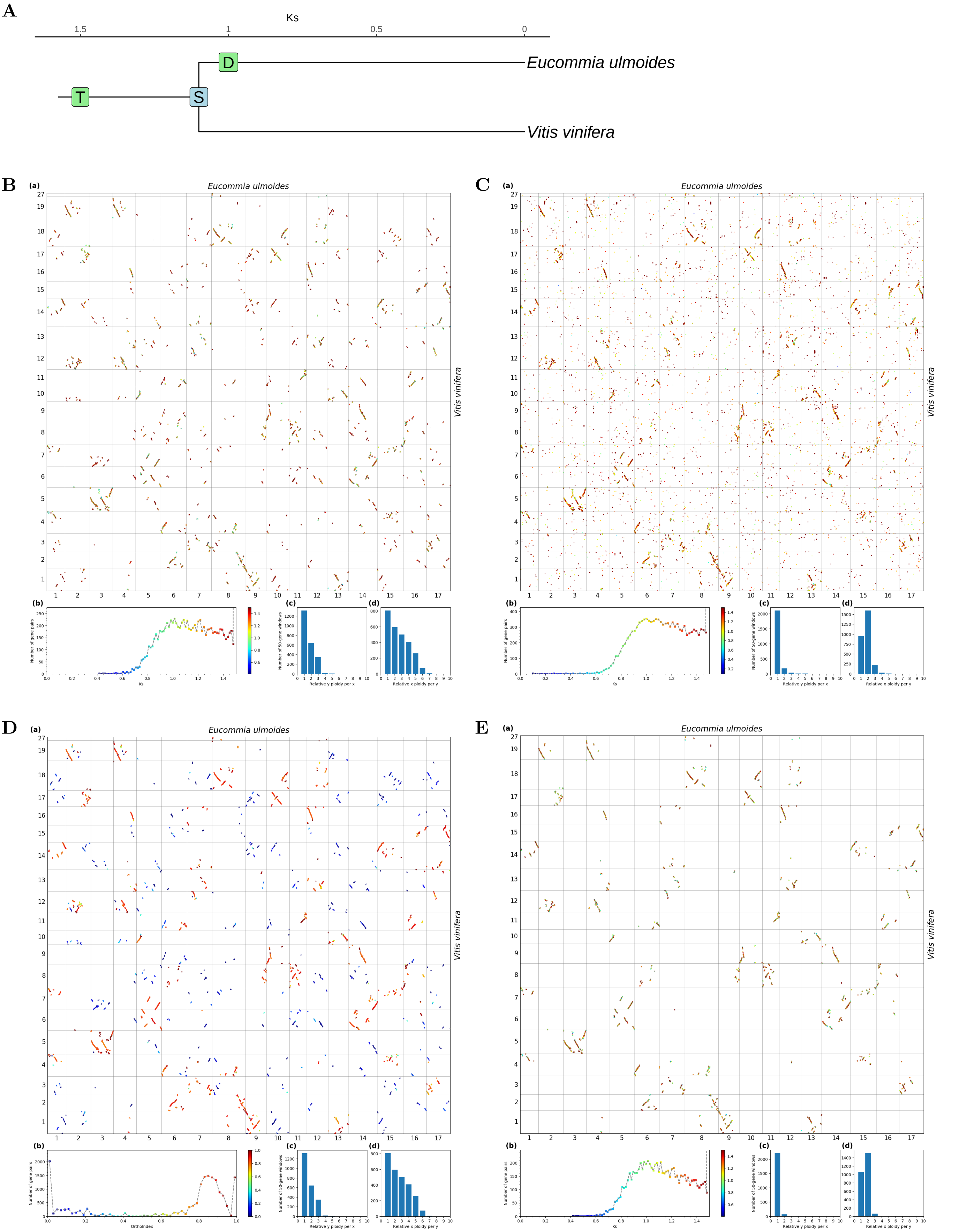

Figure S19. *Orthology Index* in the identification of orthologous synteny in *Vitis vinifera* and *Eucommia ulmoides*. Refer to **Fig.1** for detailed descriptions.

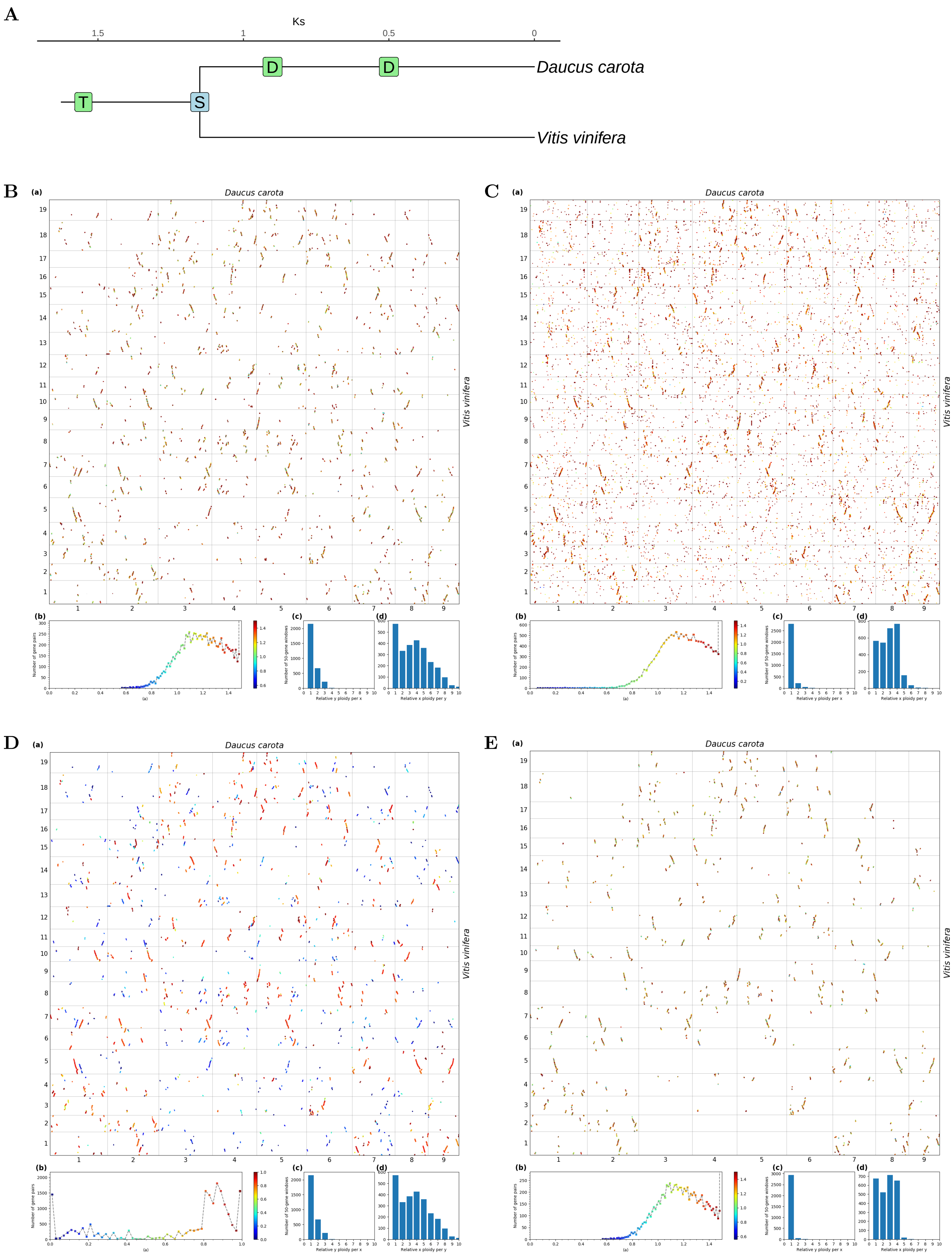

Figure S20. *Orthology Index* in the identification of orthologous synteny in *Vitis vinifera* and *Daucus carota*. Refer to **Fig.1** for detailed descriptions.

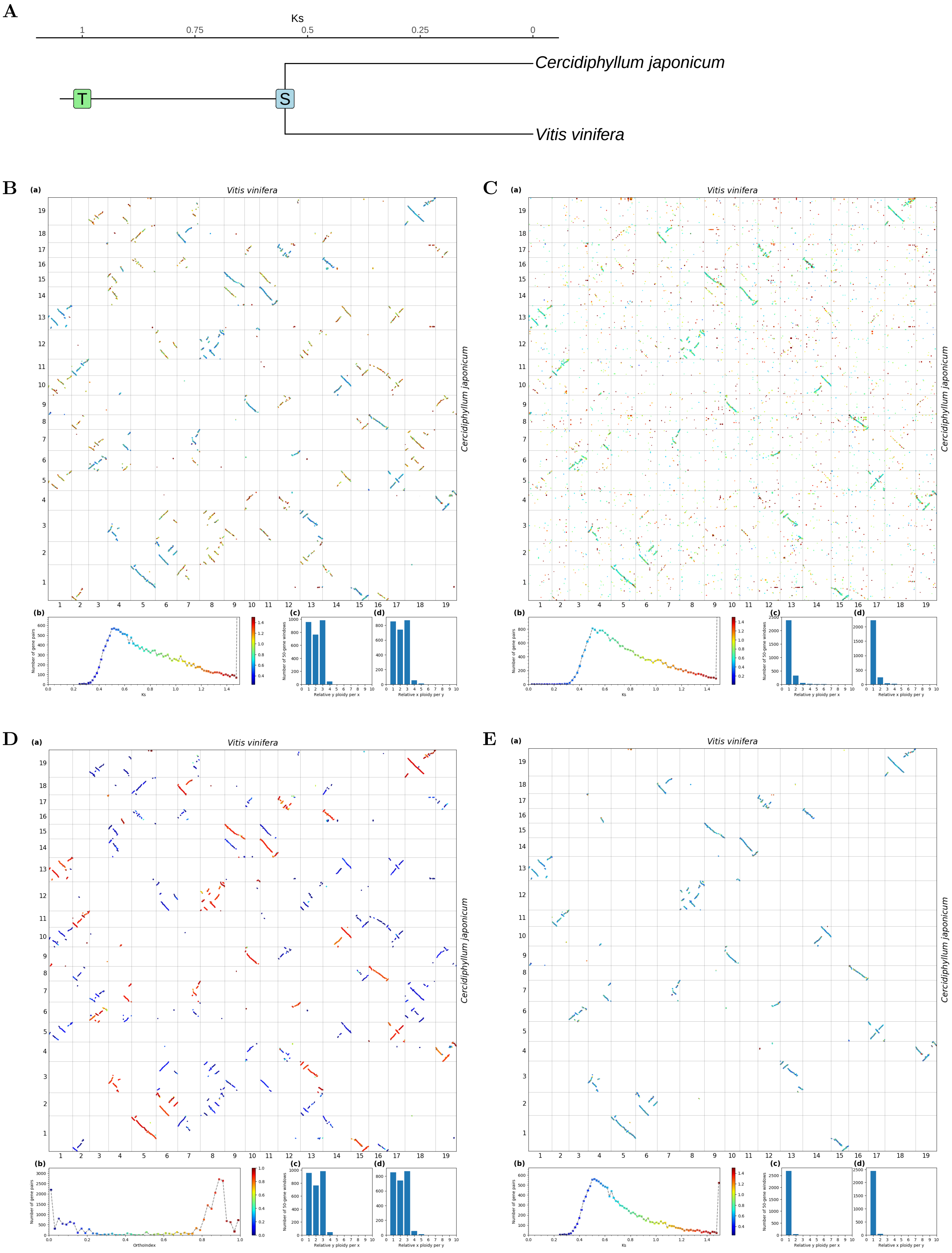

Figure S21. *Orthology Index* in the identification of orthologous synteny in *Vitis vinifera* and *Cercidiphyllum japonicum*. Refer to **Fig.1** for detailed descriptions.

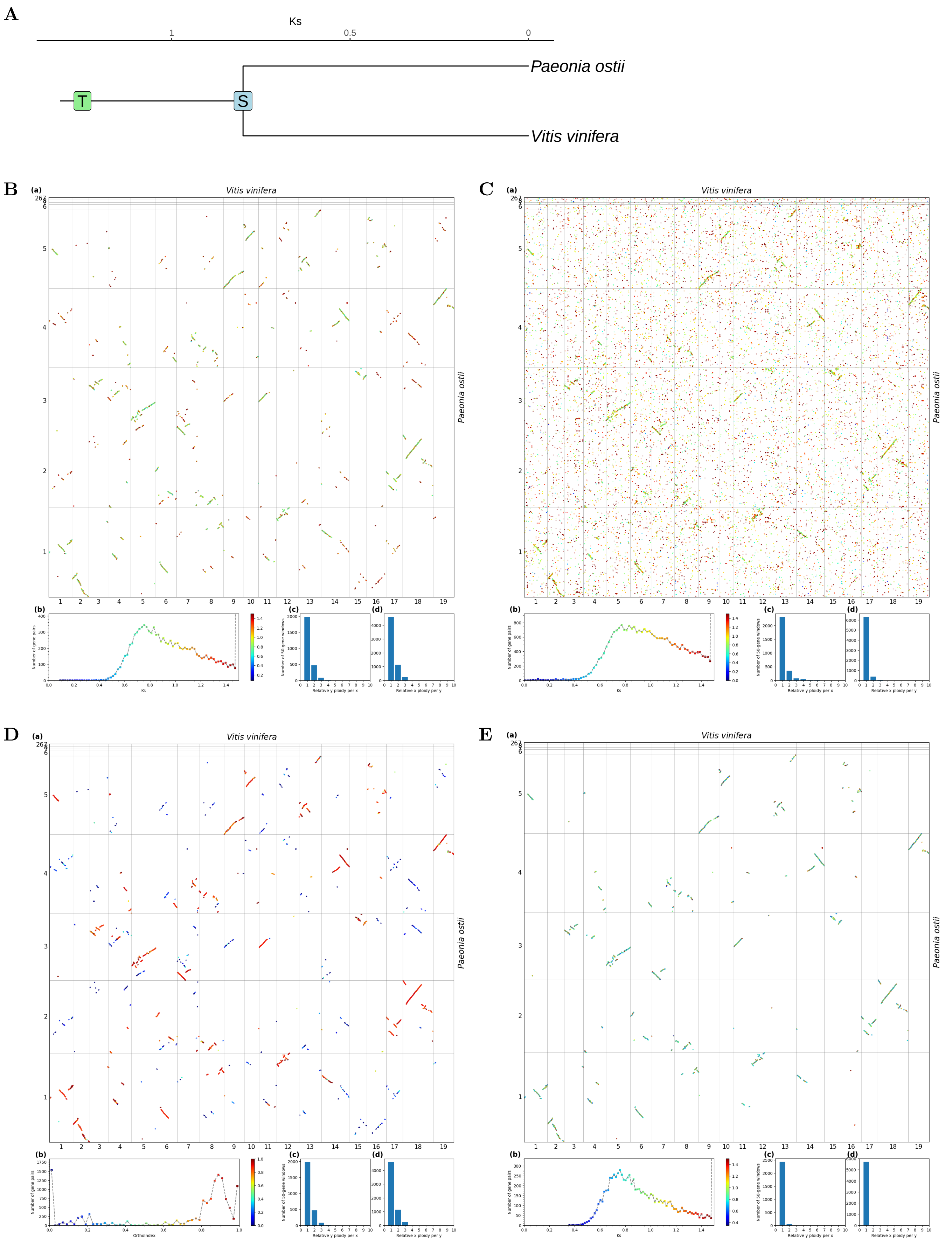

Figure S22. *Orthology Index* in the identification of orthologous synteny in *Vitis vinifera* and *Paeonia ostii*. Refer to **Fig.1** for detailed descriptions.

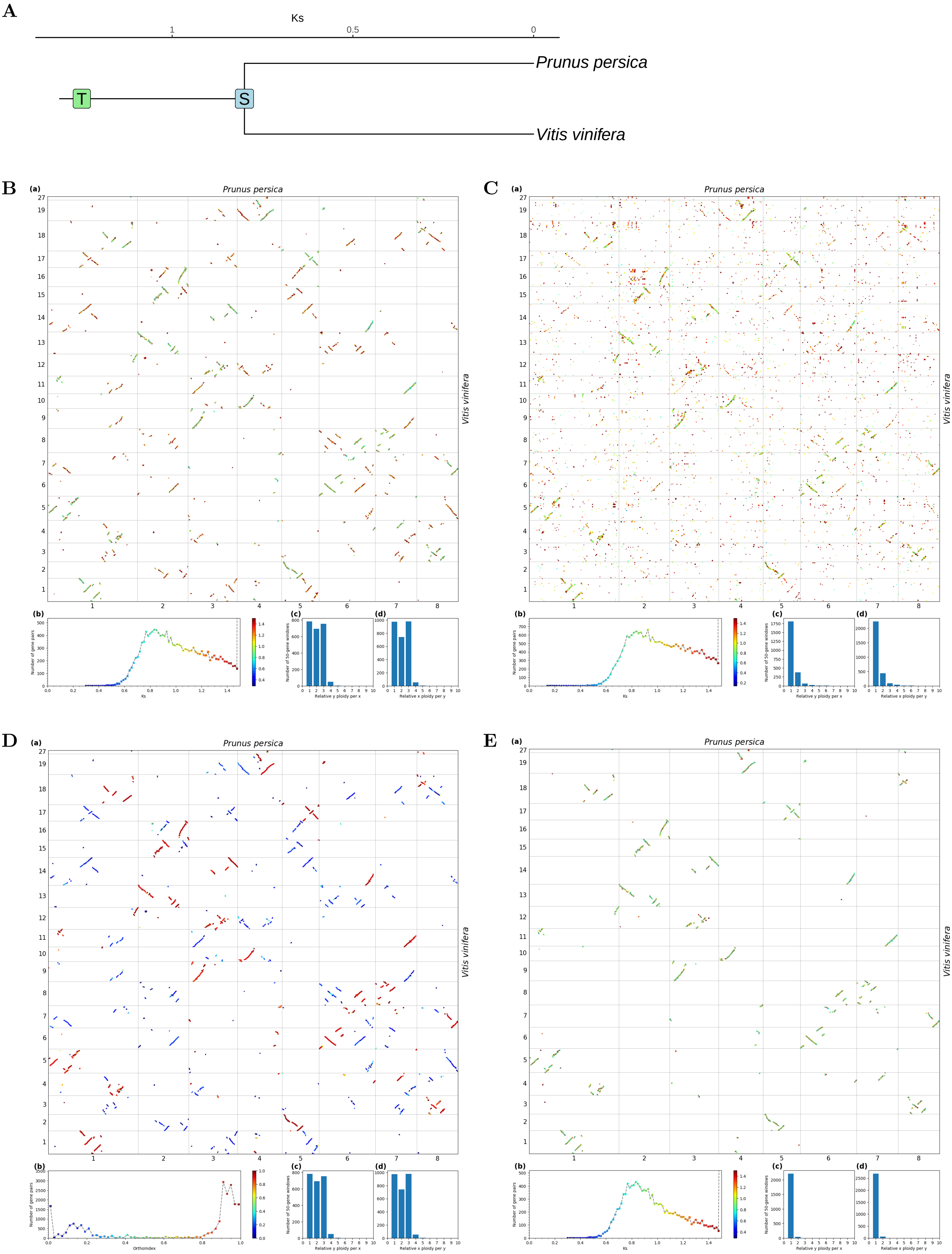

Figure S23. *Orthology Index* in the identification of orthologous synteny in *Vitis vinifera* and *Prunus persica*. Refer to **Fig.1** for detailed descriptions.

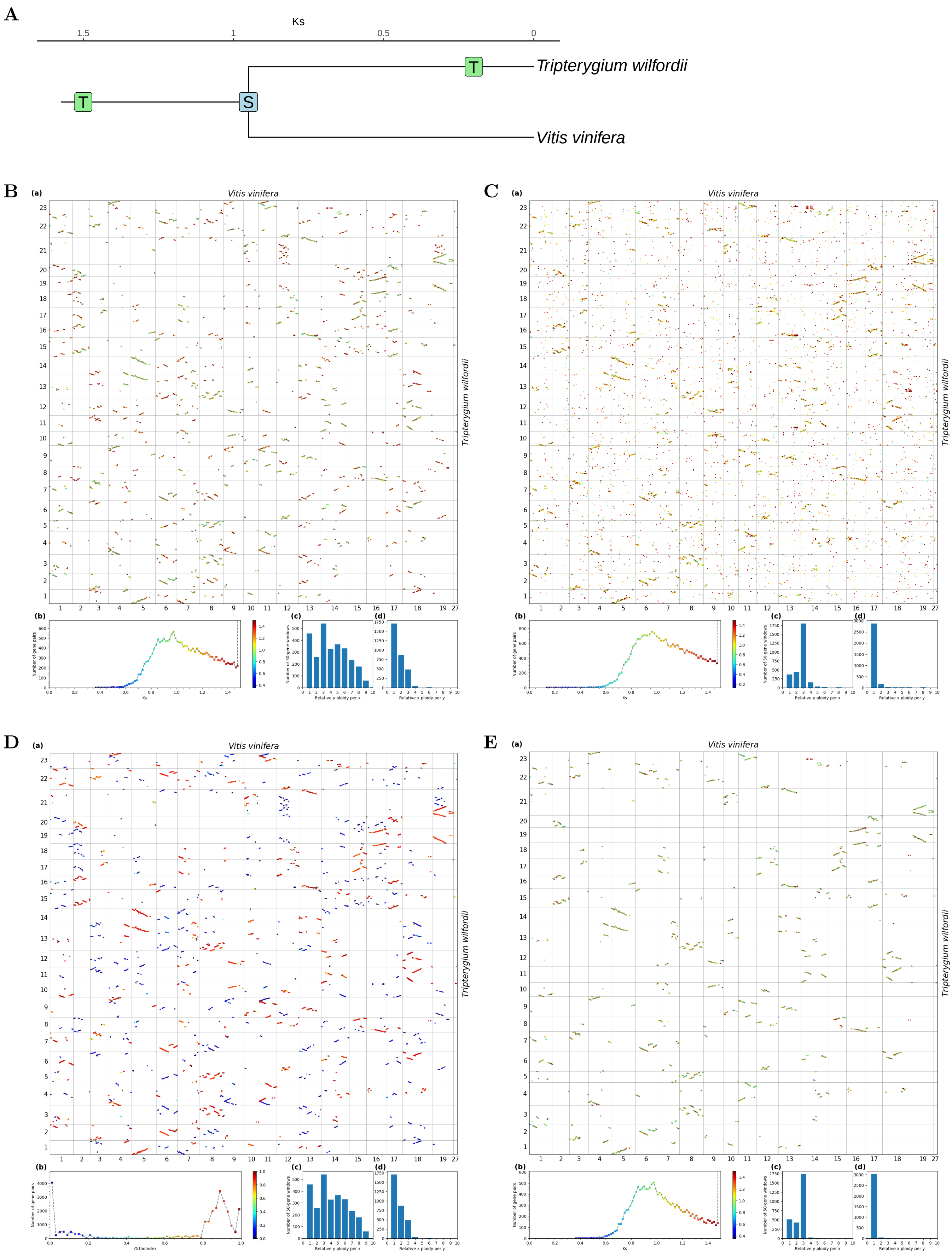

Figure S24. *Orthology Index* in the identification of orthologous synteny in *Vitis vinifera* and *Tripterygium wilfordii*. Refer to **Fig.1** for detailed descriptions.

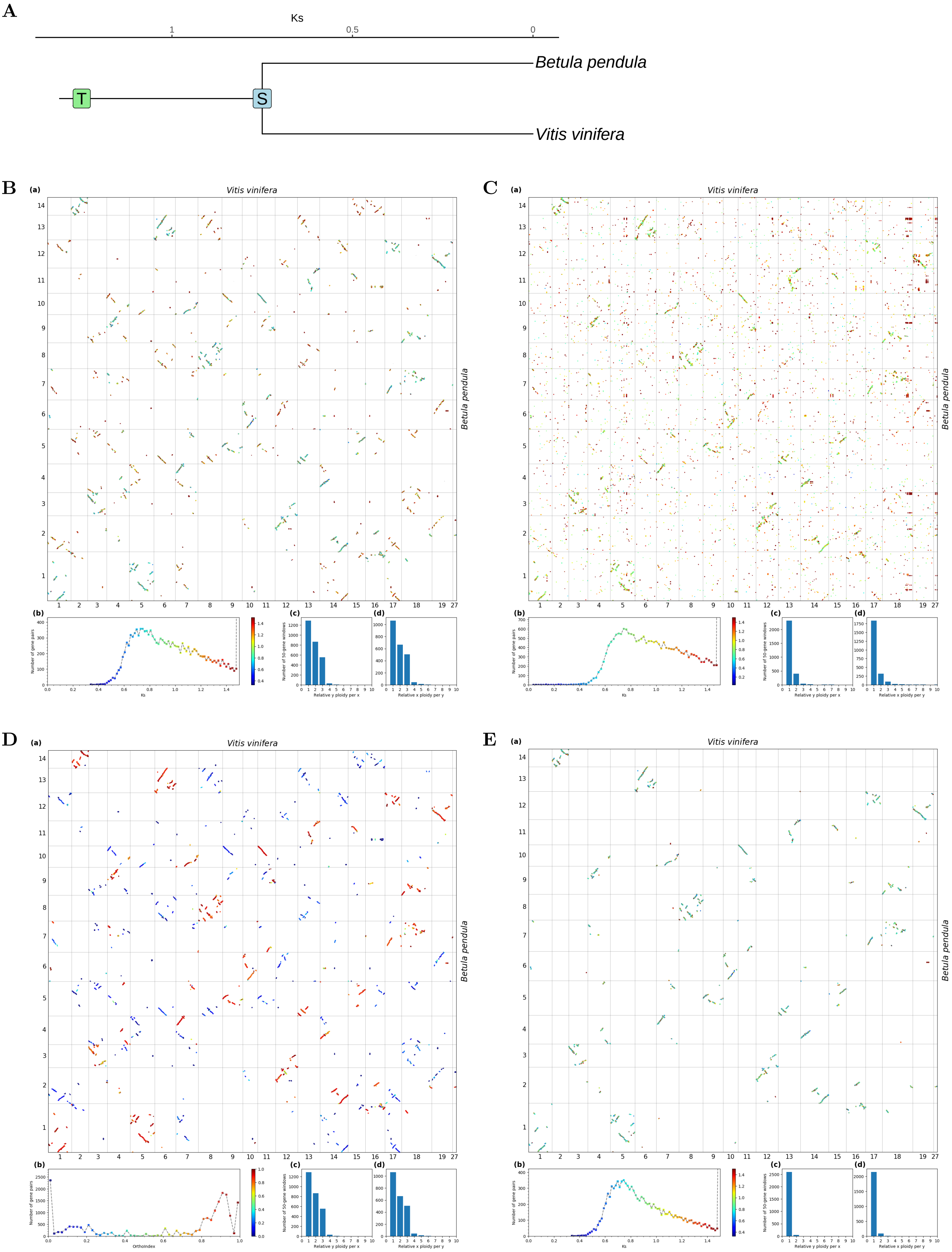

Figure S25. *Orthology Index* in the identification of orthologous synteny in *Vitis vinifera* and *Betula pendula*. Refer to **Fig.1** for detailed descriptions.

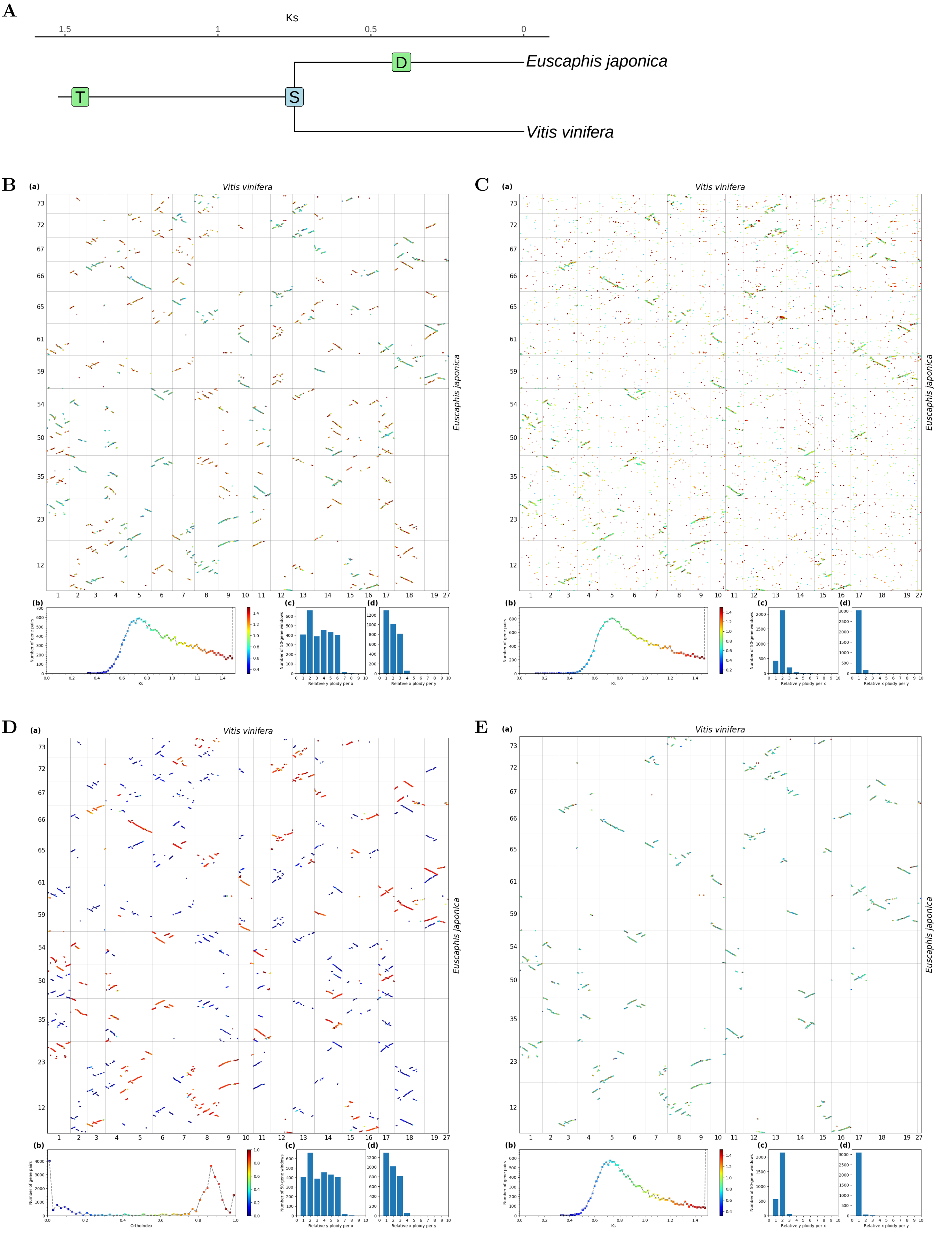

Figure S26. *Orthology Index* in the identification of orthologous synteny in *Vitis vinifera* and *Euscaphis japonica*. Refer to **Fig.1** for detailed descriptions.

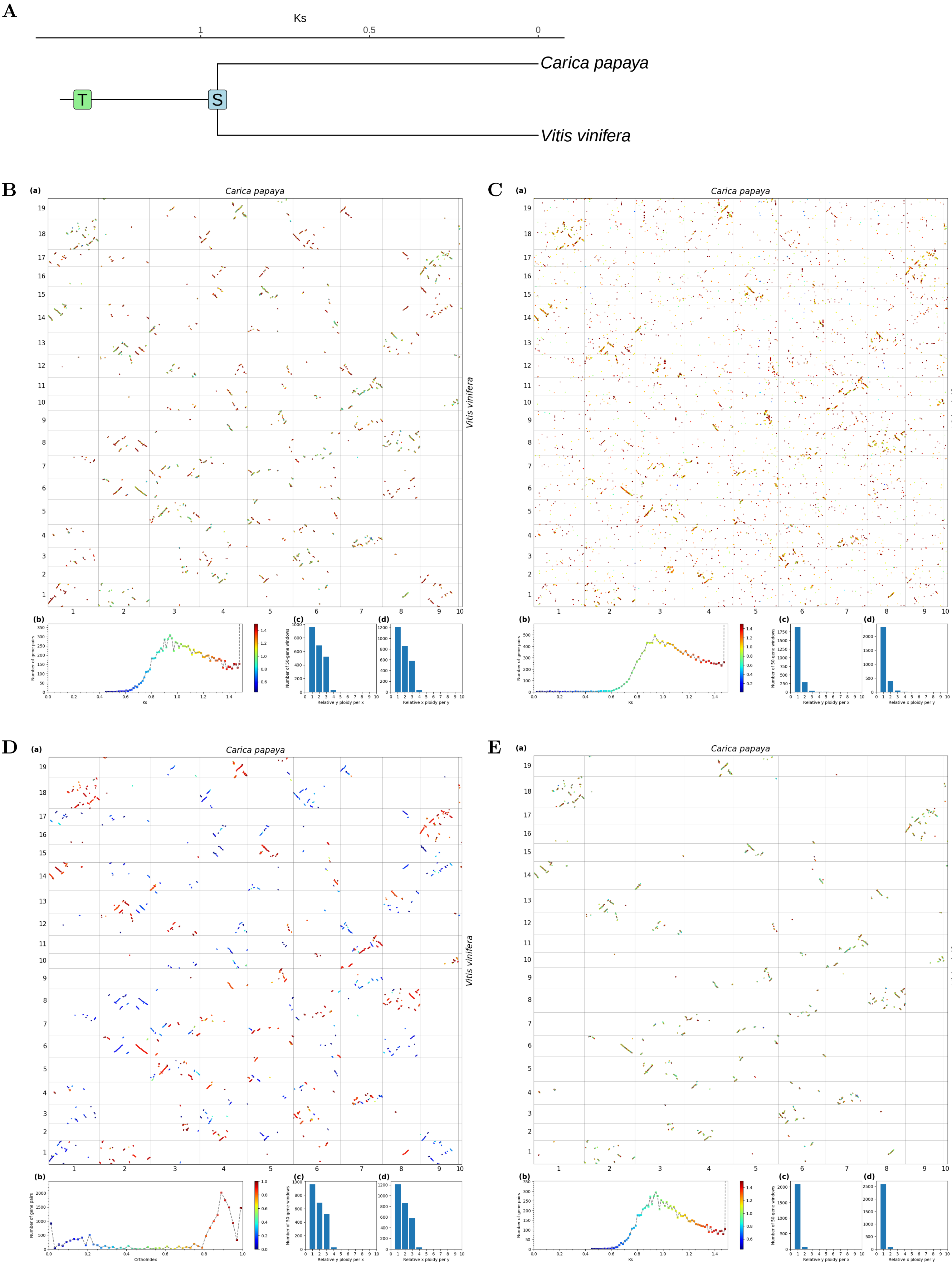

Figure S27. *Orthology Index* in the identification of orthologous synteny in *Vitis vinifera* and *Carica papaya*. Refer to **Fig.1** for detailed descriptions.

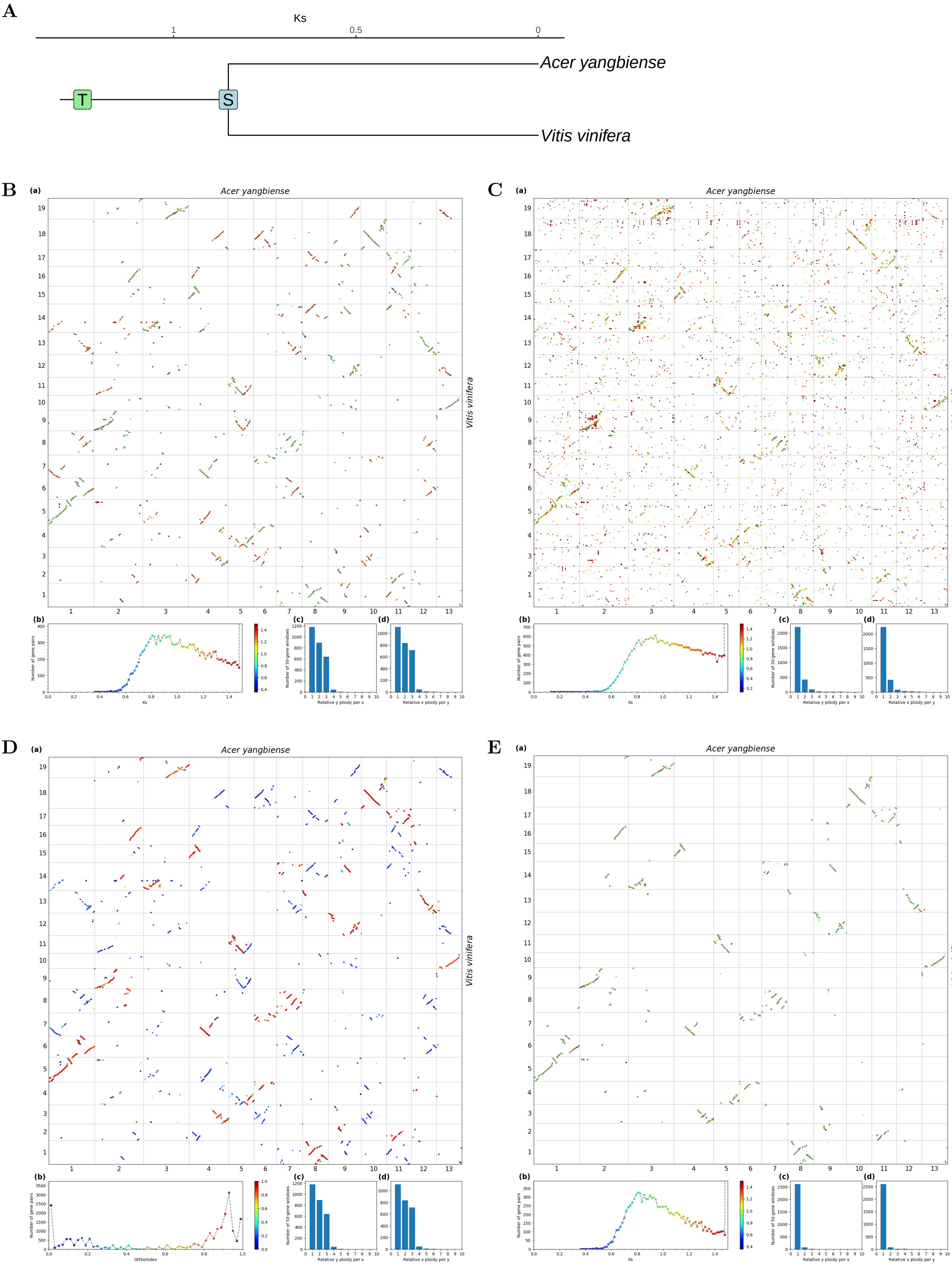

Figure S28. *Orthology Index* in the identification of orthologous synteny in *Vitis vinifera* and *Acer yangbiense*. Refer to **Fig.1** for detailed descriptions.

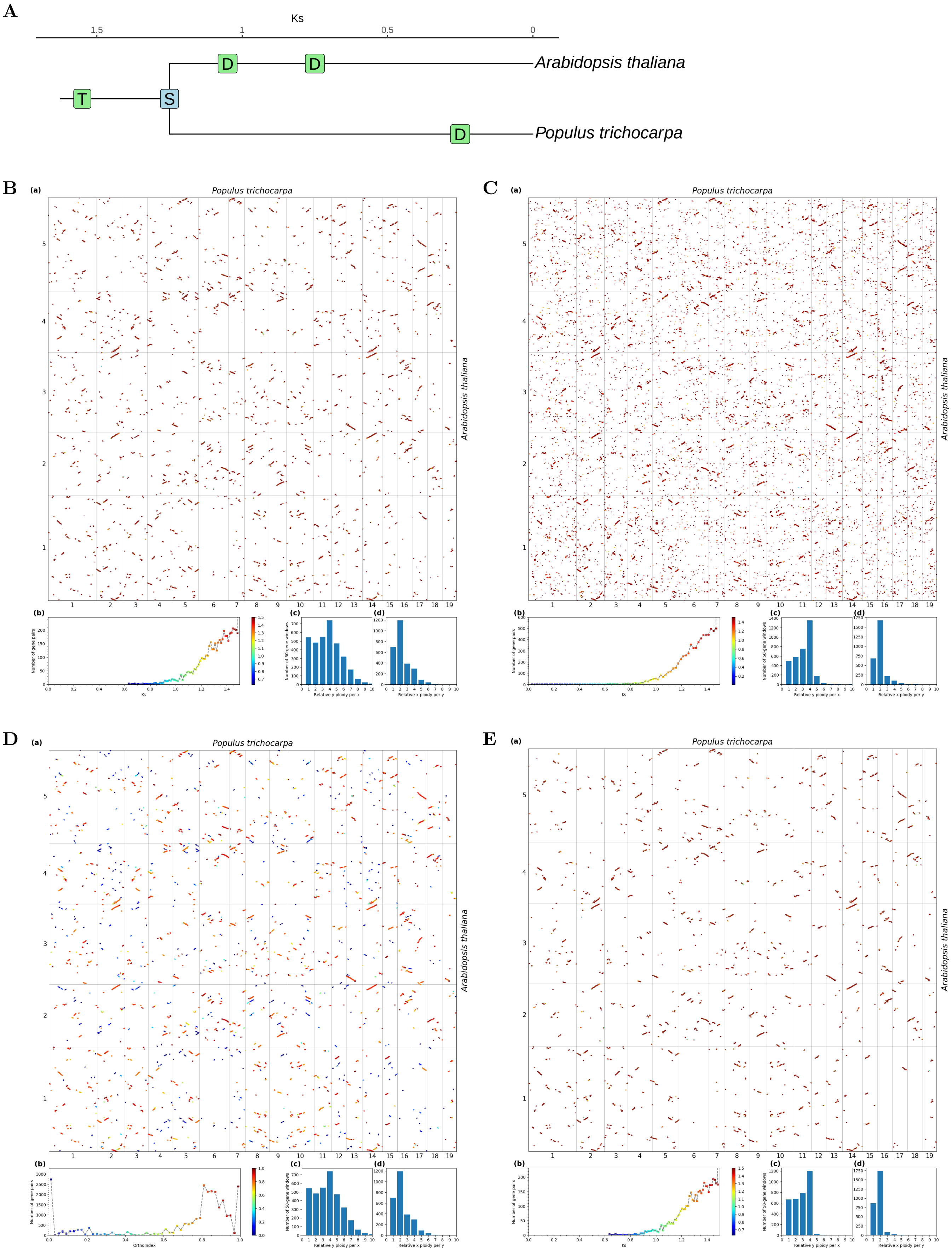

Figure S29. *Orthology Index* in the identification of orthologous synteny in *Populus trichocarpa* and *Arabidopsis thaliana*. Refer to **Fig.1** for detailed descriptions.

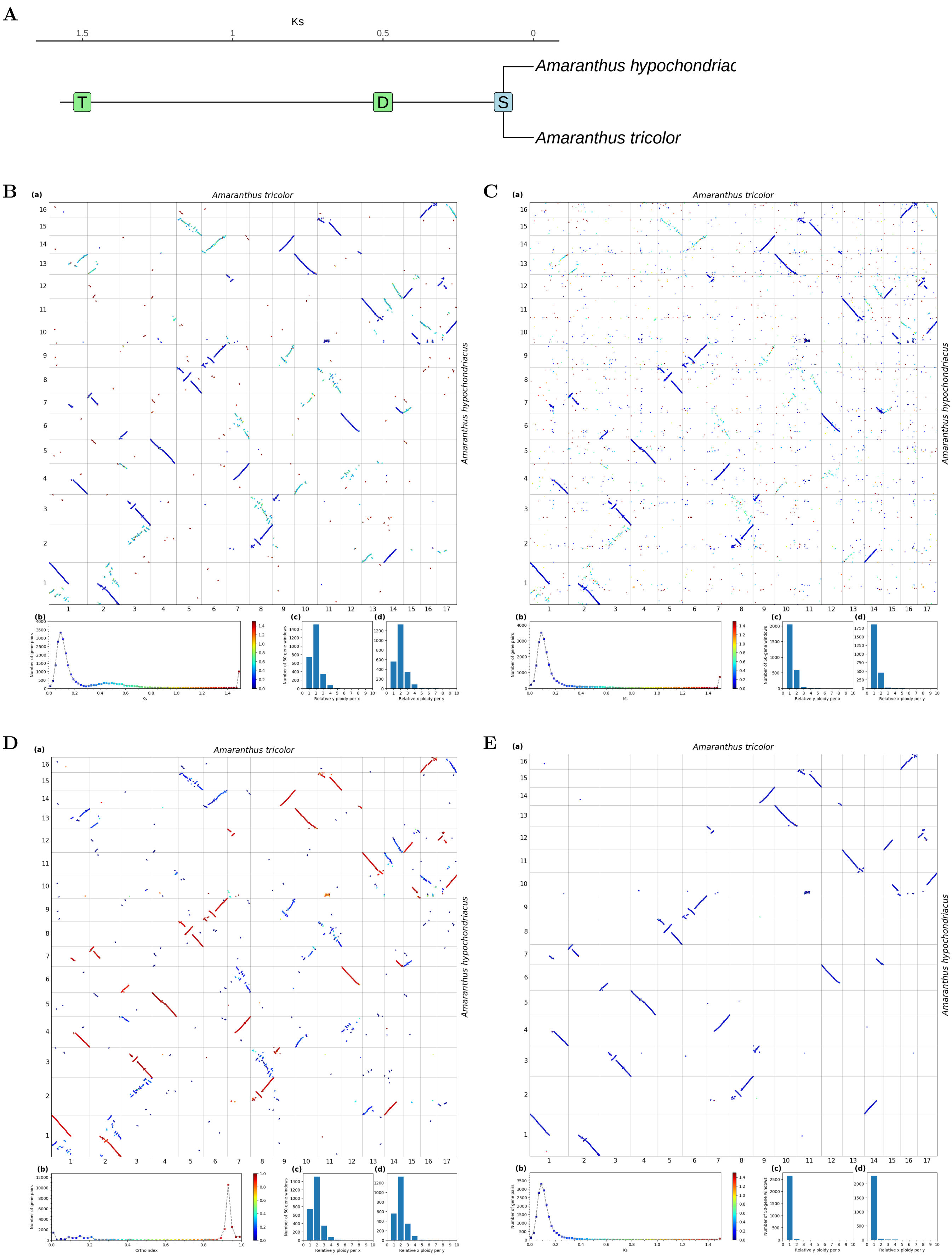

Figure S30. *Orthology Index* in the identification of orthologous synteny in *Amaranthus tricolor* and *Amaranthus hypochondriacus*. Refer to **Fig.1** for detailed descriptions.

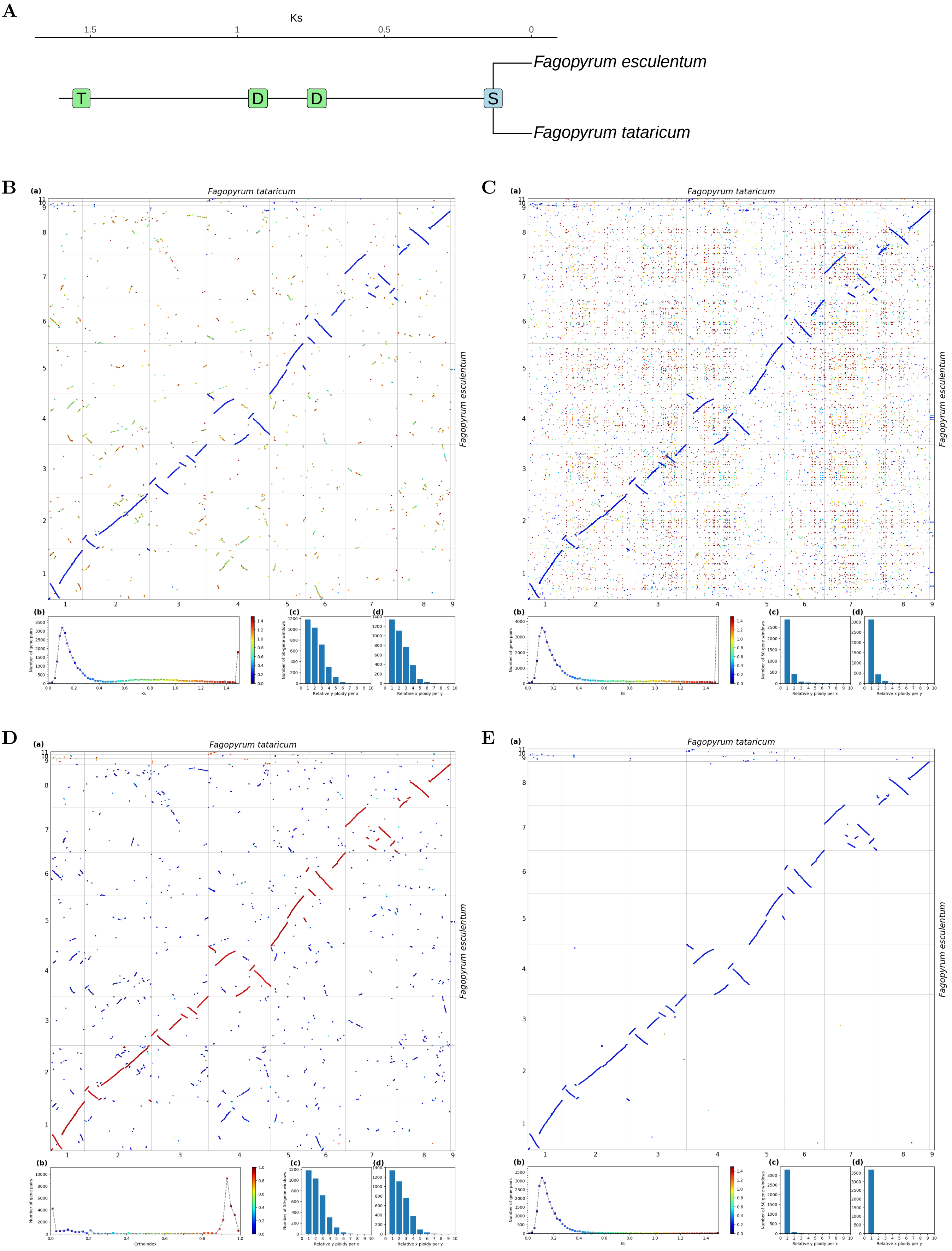

Figure S31. *Orthology Index* in the identification of orthologous synteny in *Fagopyrum tataricum* and *Fagopyrum esculentum*. Refer to **Fig.1** for detailed descriptions.

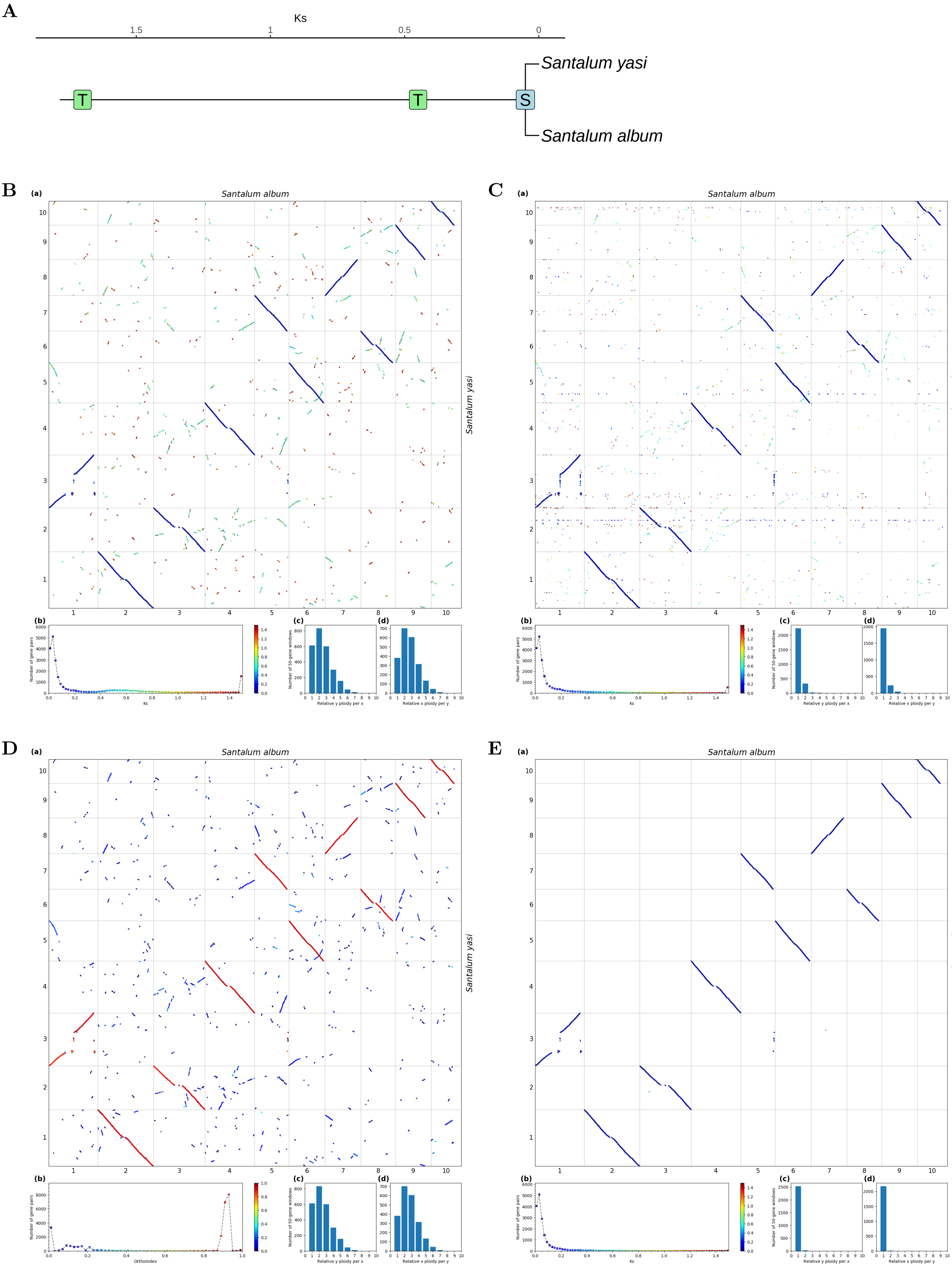

Figure S32. *Orthology Index* in the identification of orthologous synteny in *Santalum album* and *Santalum yasi*. Refer to **Fig.1** for detailed descriptions.

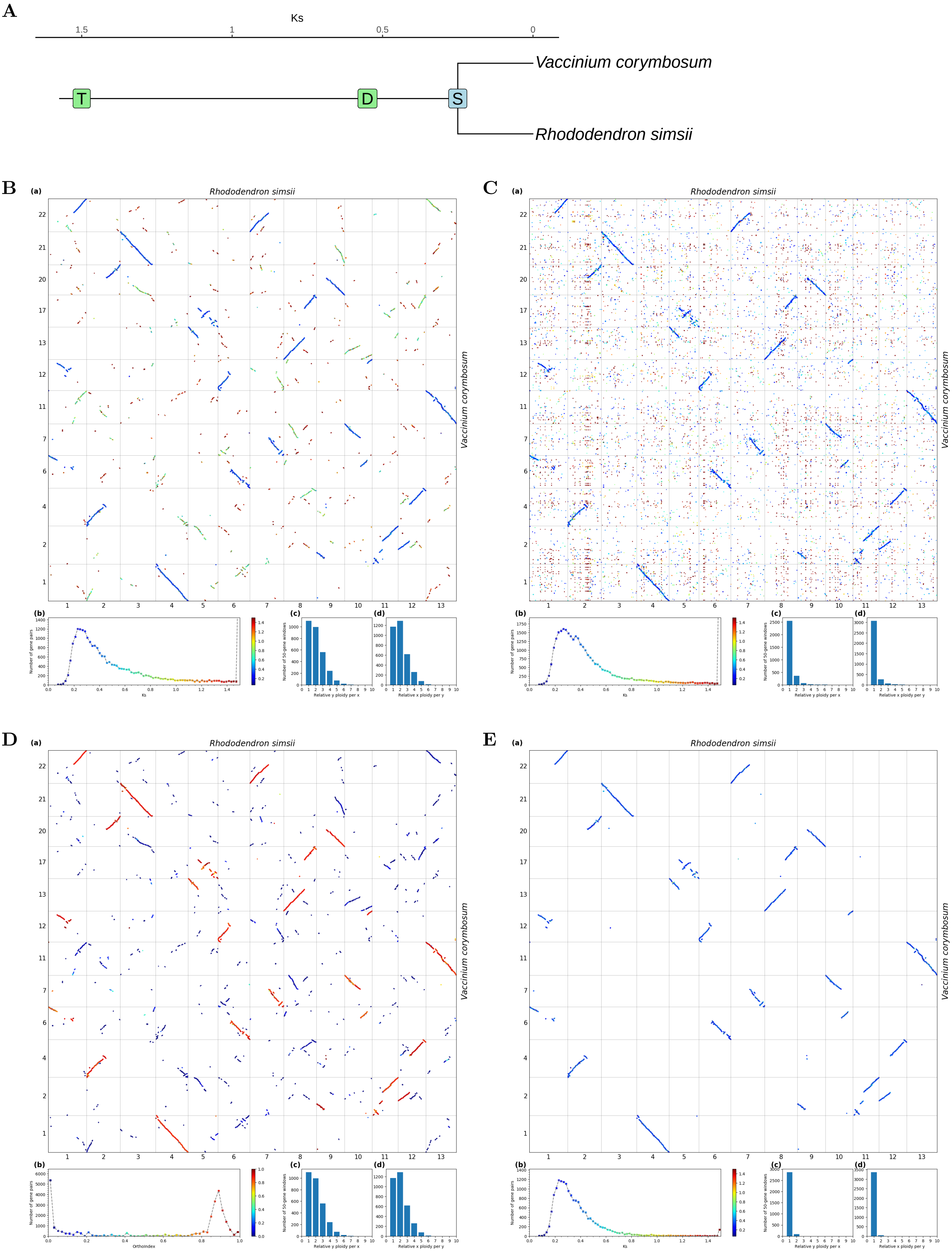

Figure S33. *Orthology Index* in the identification of orthologous synteny in *Rhododendron simsii* and *Vaccinium corymbosum*. Refer to **Fig.1** for detailed descriptions.

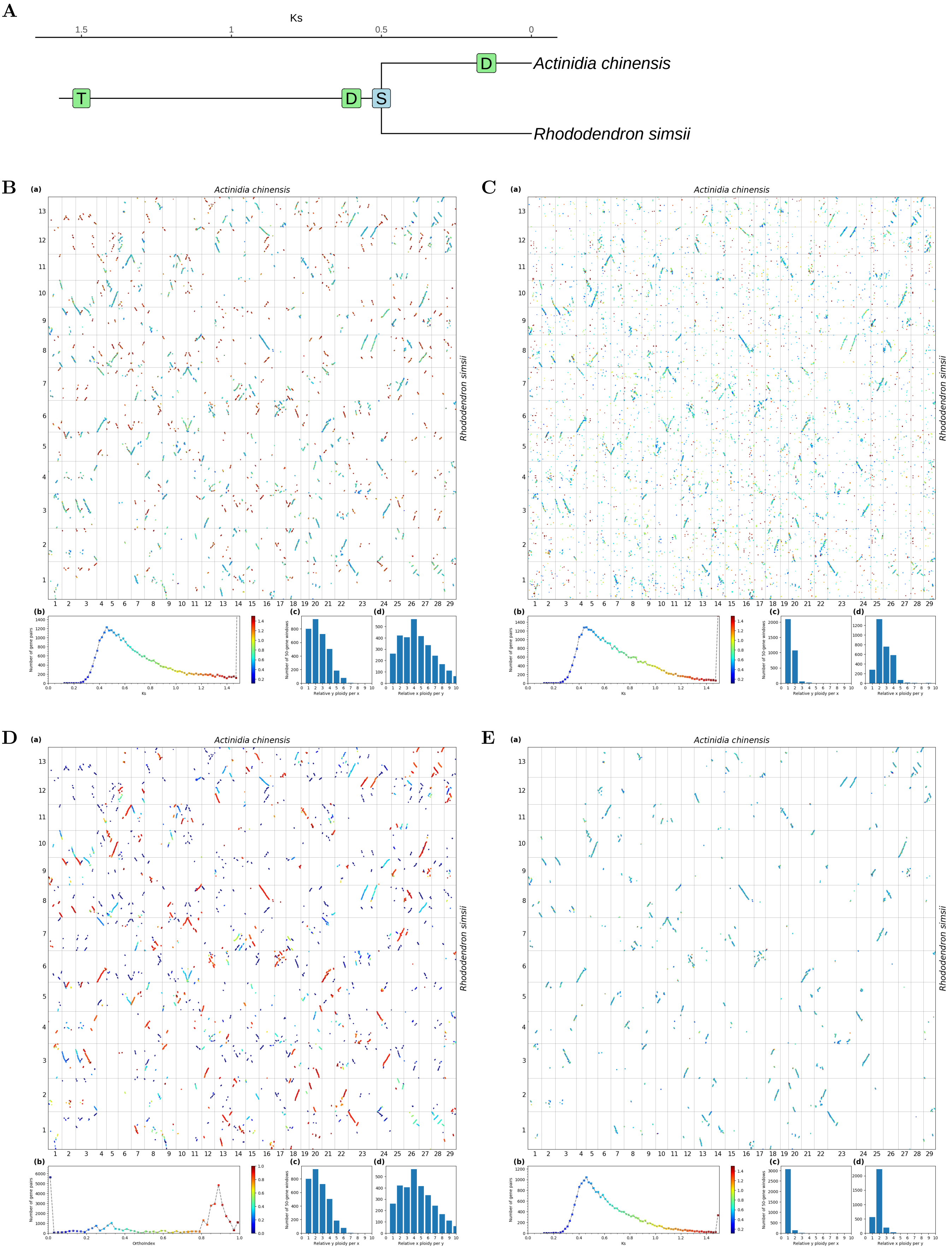

Figure S34. *Orthology Index* in the identification of orthologous synteny in *Rhododendron simsii* and *Actinidia chinensis*. Refer to **Fig.1** for detailed descriptions.

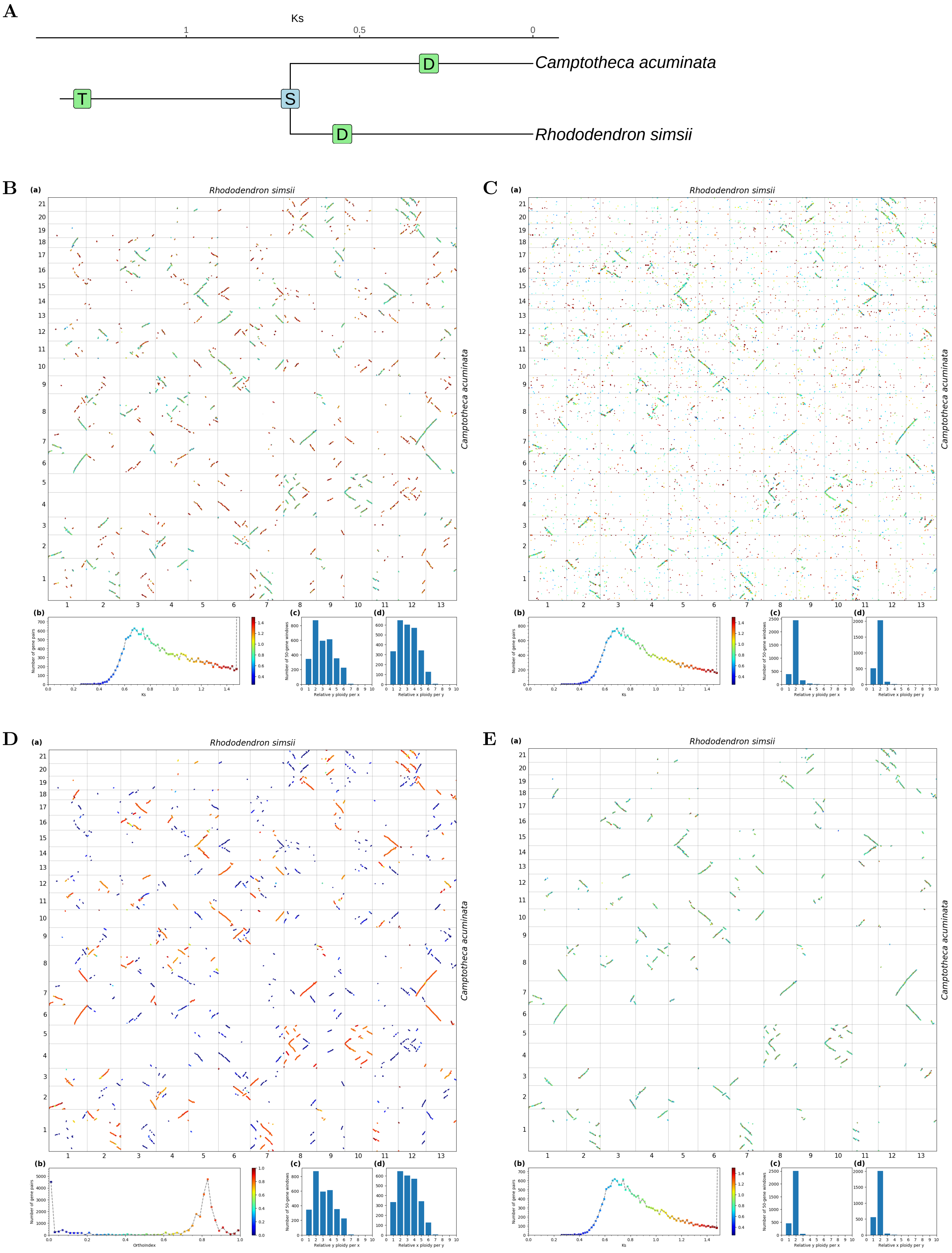

Figure S35. *Orthology Index* in the identification of orthologous synteny in *Rhododendron simsii* and *Camptotheca acuminata*. Refer to **Fig.1** for detailed descriptions.

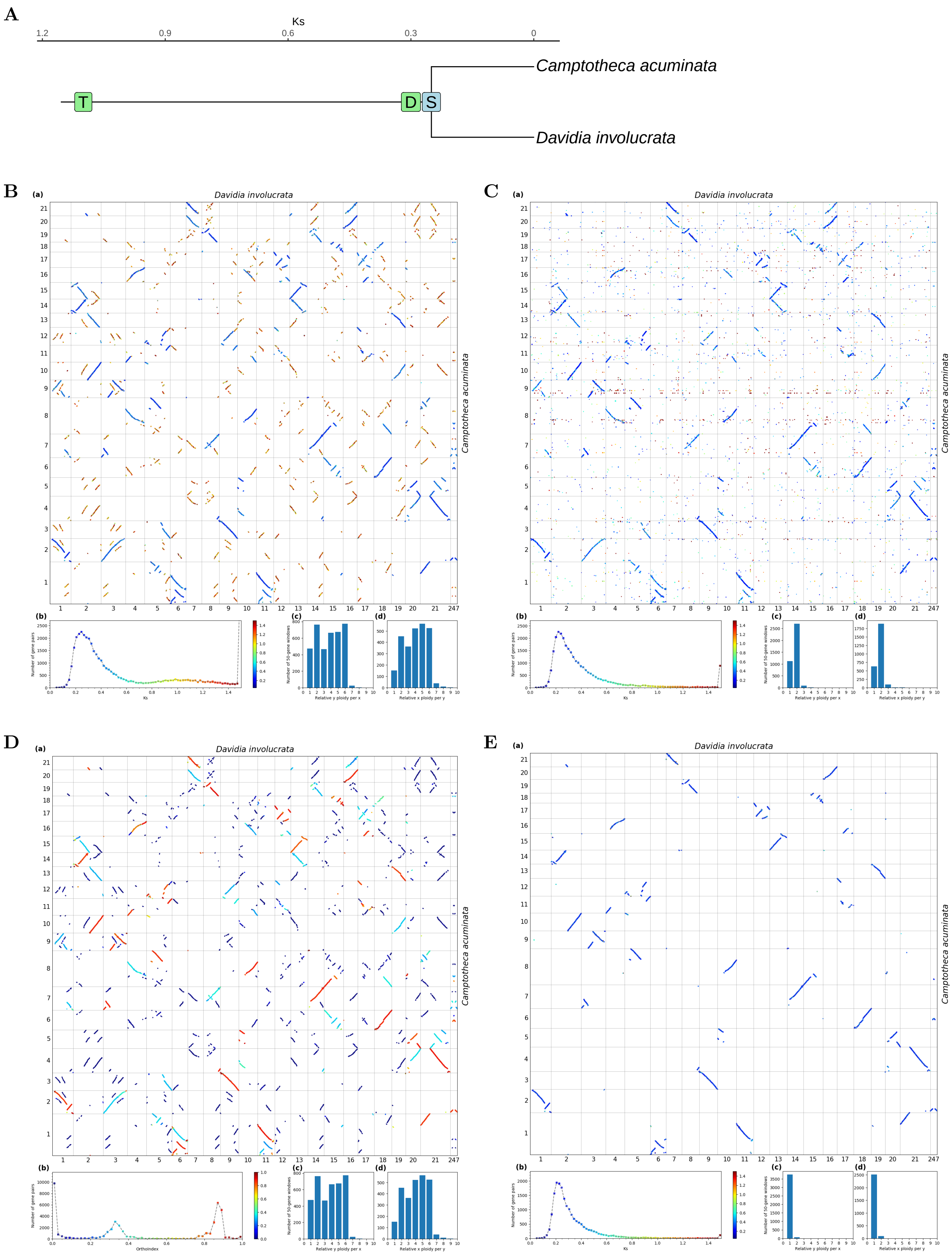

Figure S36. *Orthology Index* in the identification of orthologous synteny in *Davidia involucrata* and *Camptotheca acuminata*. Refer to **Fig.1** for detailed descriptions.

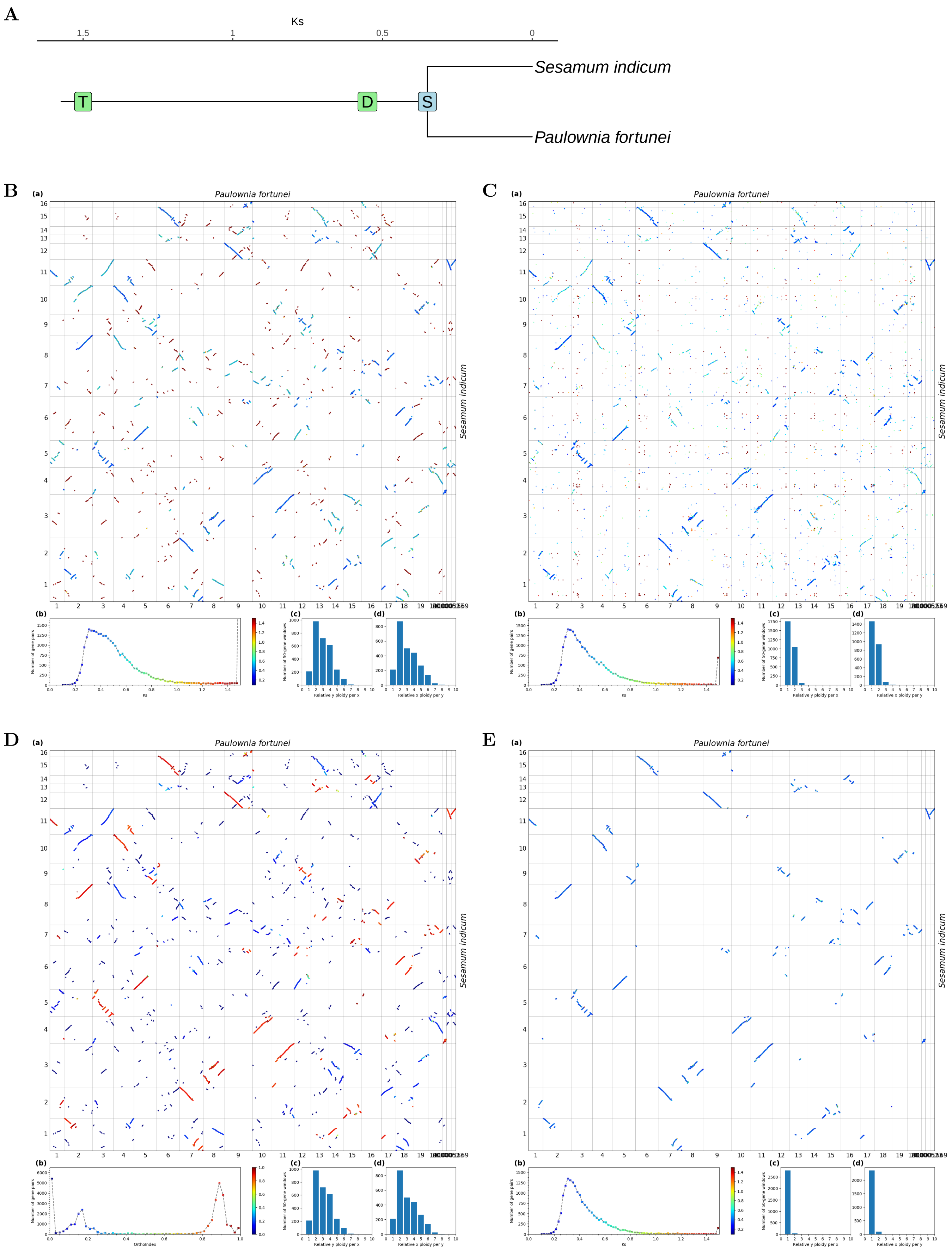

Figure S37. *Orthology Index* in the identification of orthologous synteny in *Paulownia fortunei* and *Sesamum indicum*. Refer to **Fig.1** for detailed descriptions.

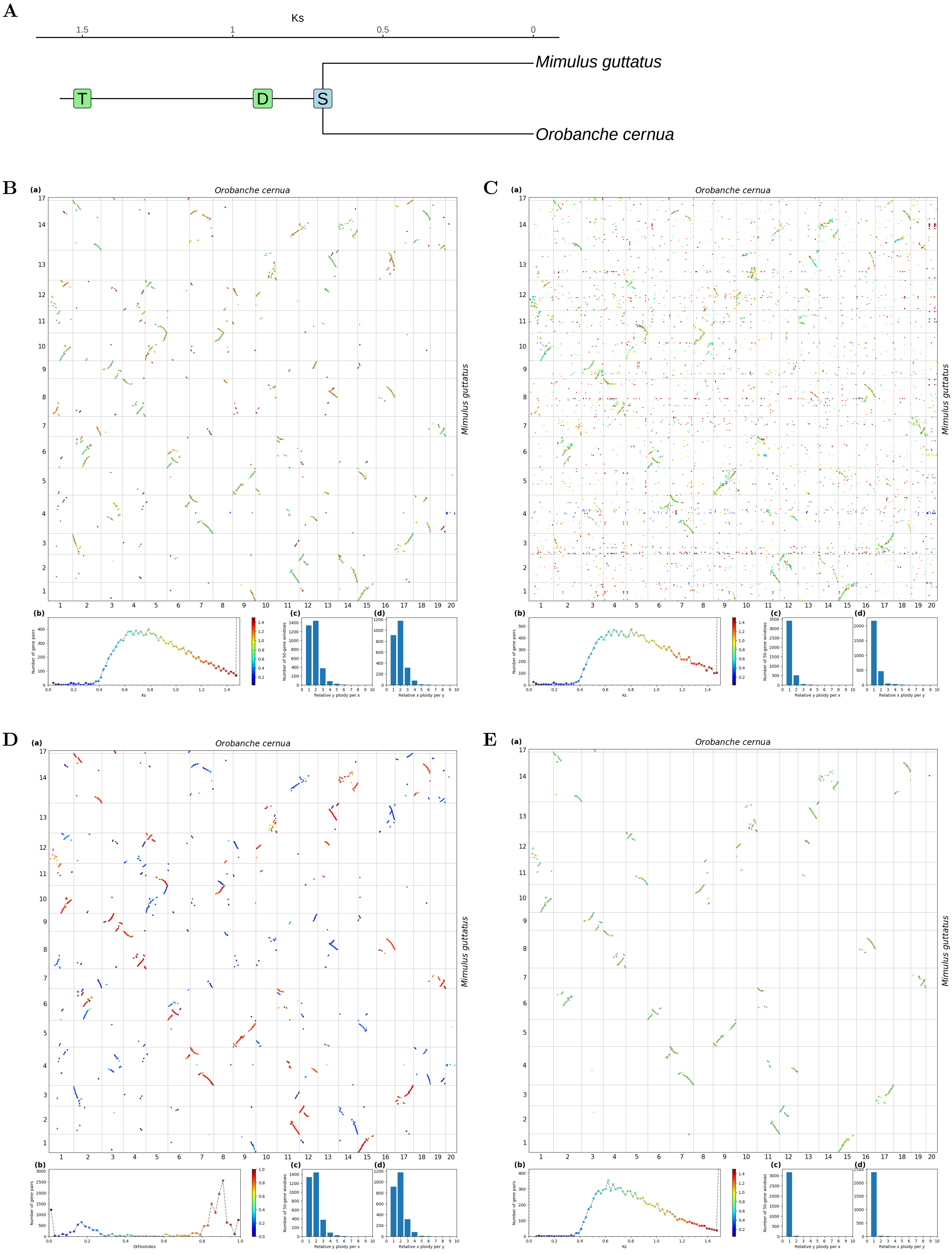

Figure S38. *Orthology Index* in the identification of orthologous synteny in *Orobanche cernua* and *Mimulus guttatus*. Refer to **Fig.1** for detailed descriptions.

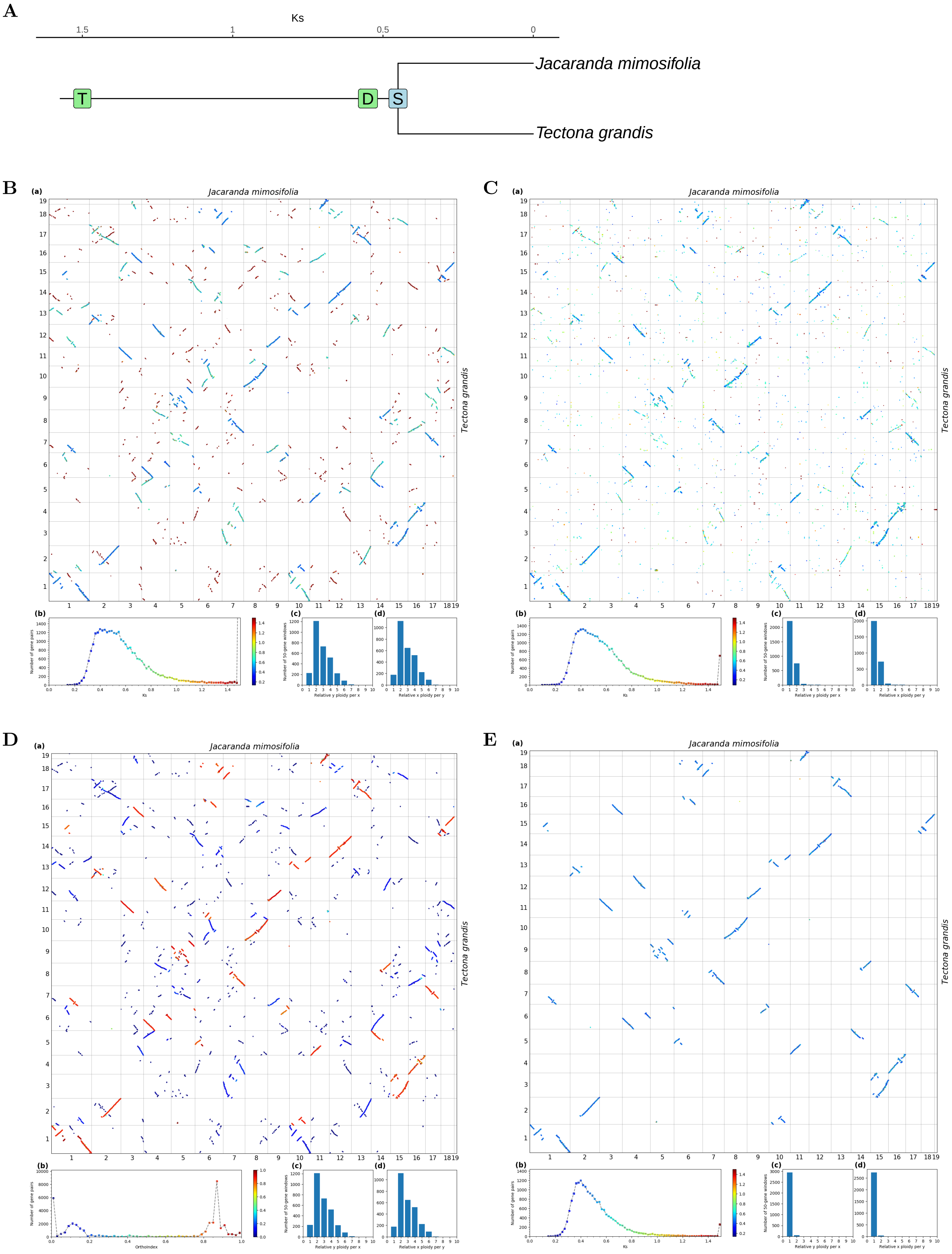

Figure S39. *Orthology Index* in the identification of orthologous synteny in *Tectona grandis* and *Jacaranda mimosifolia*. Refer to **Fig.1** for detailed descriptions.

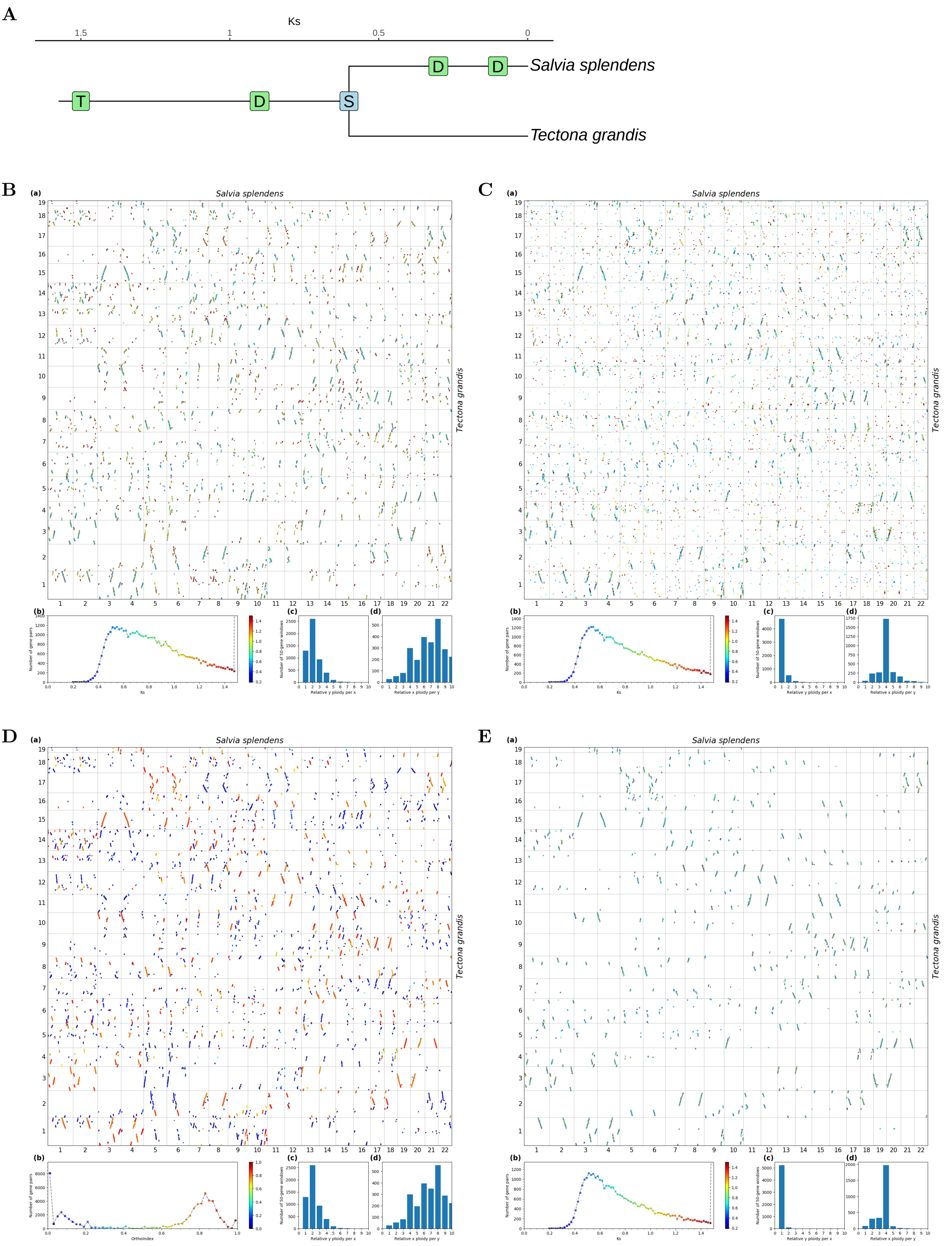

Figure S40. *Orthology Index* in the identification of orthologous synteny in *Tectona grandis* and *Salvia splendens*. Refer to **Fig.1** for detailed descriptions.

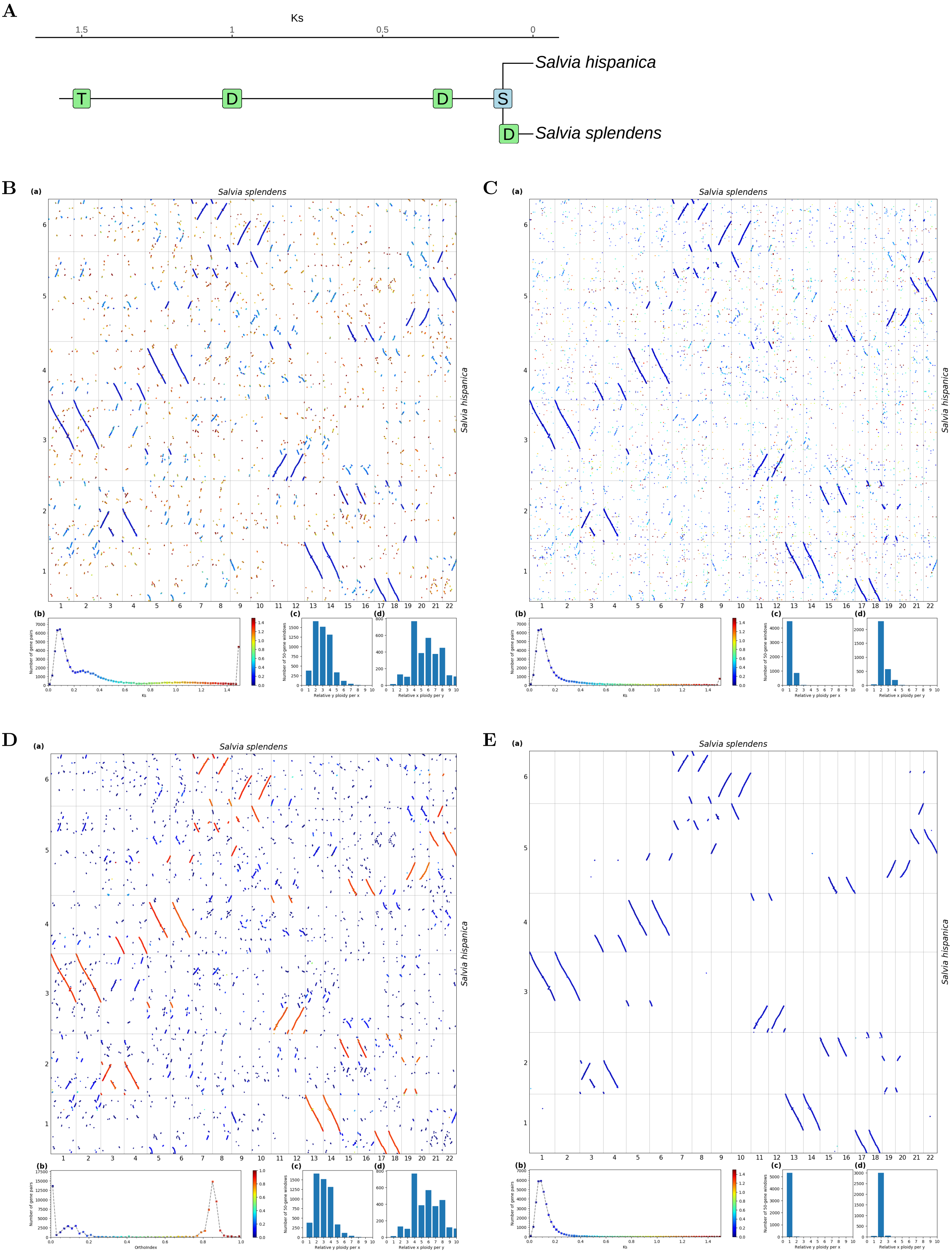

Figure S41. *Orthology Index* in the identification of orthologous synteny in *Salvia splendens* and *Salvia hispanica*. Refer to **Fig.1** for detailed descriptions.

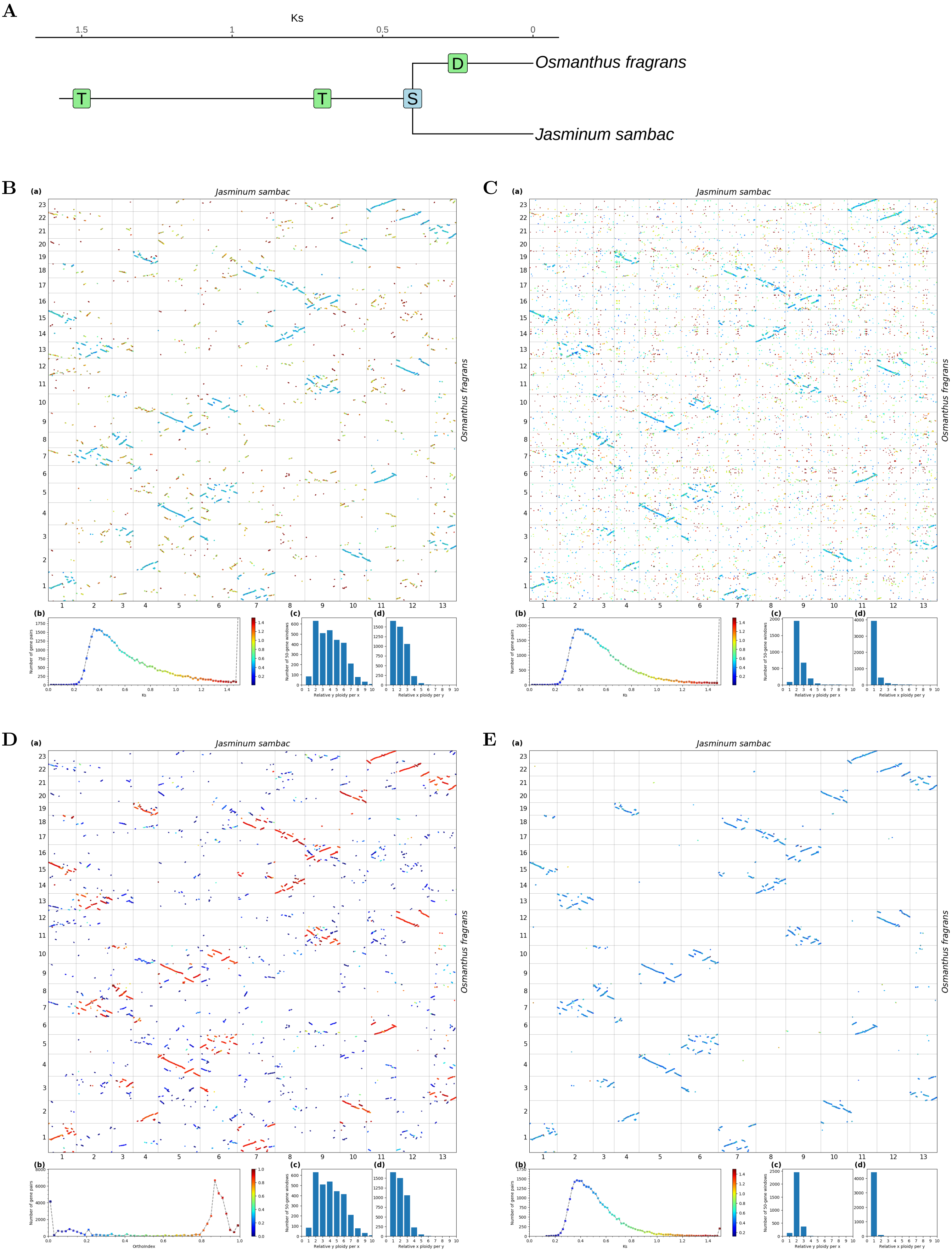

Figure S42. *Orthology Index* in the identification of orthologous synteny in *Jasminum sambac* and *Osmanthus fragrans*. Refer to **Fig.1** for detailed descriptions.

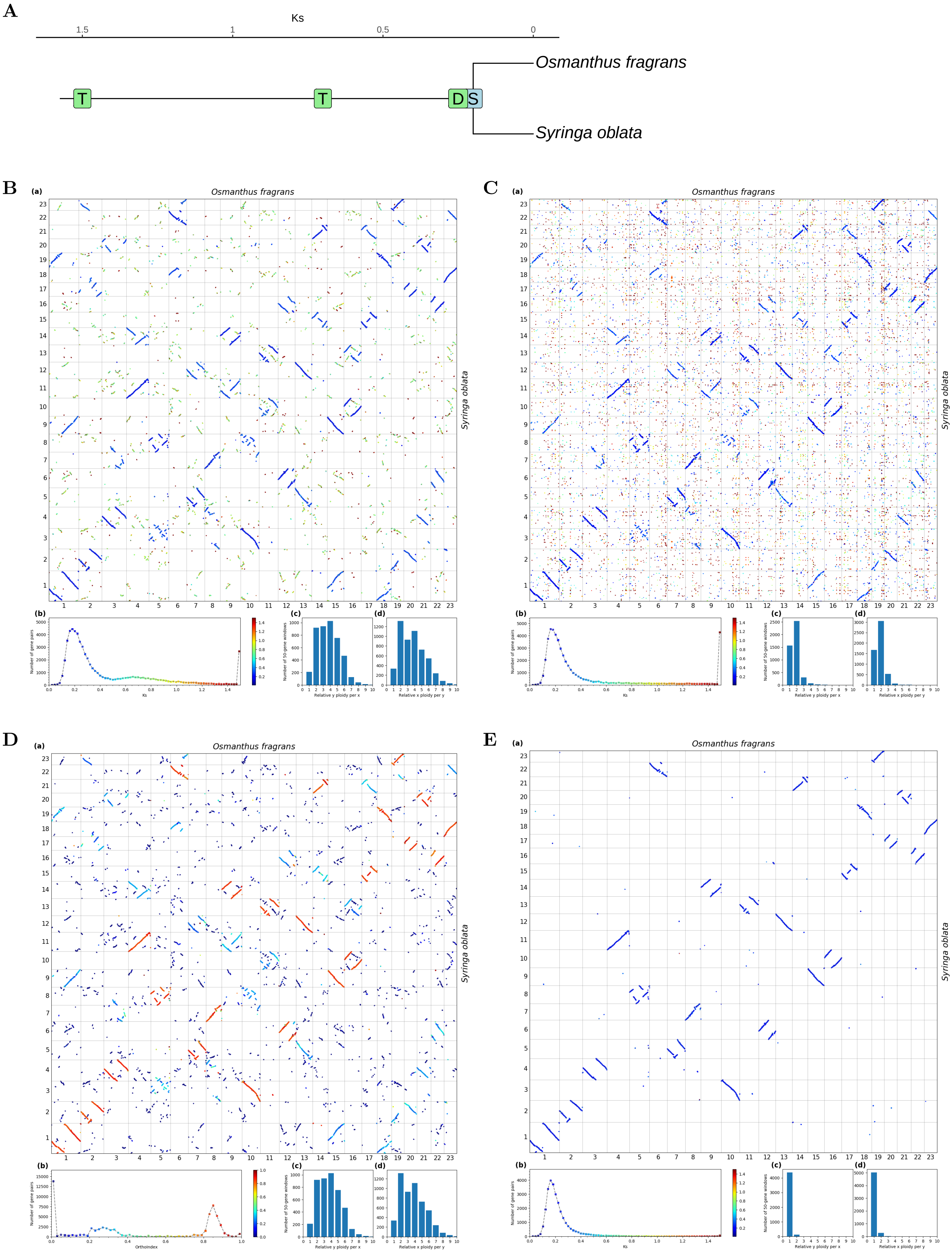

Figure S43. *Orthology Index* in the identification of orthologous synteny in *Syringa oblata* and *Osmanthus fragrans*. Refer to **Fig.1** for detailed descriptions.

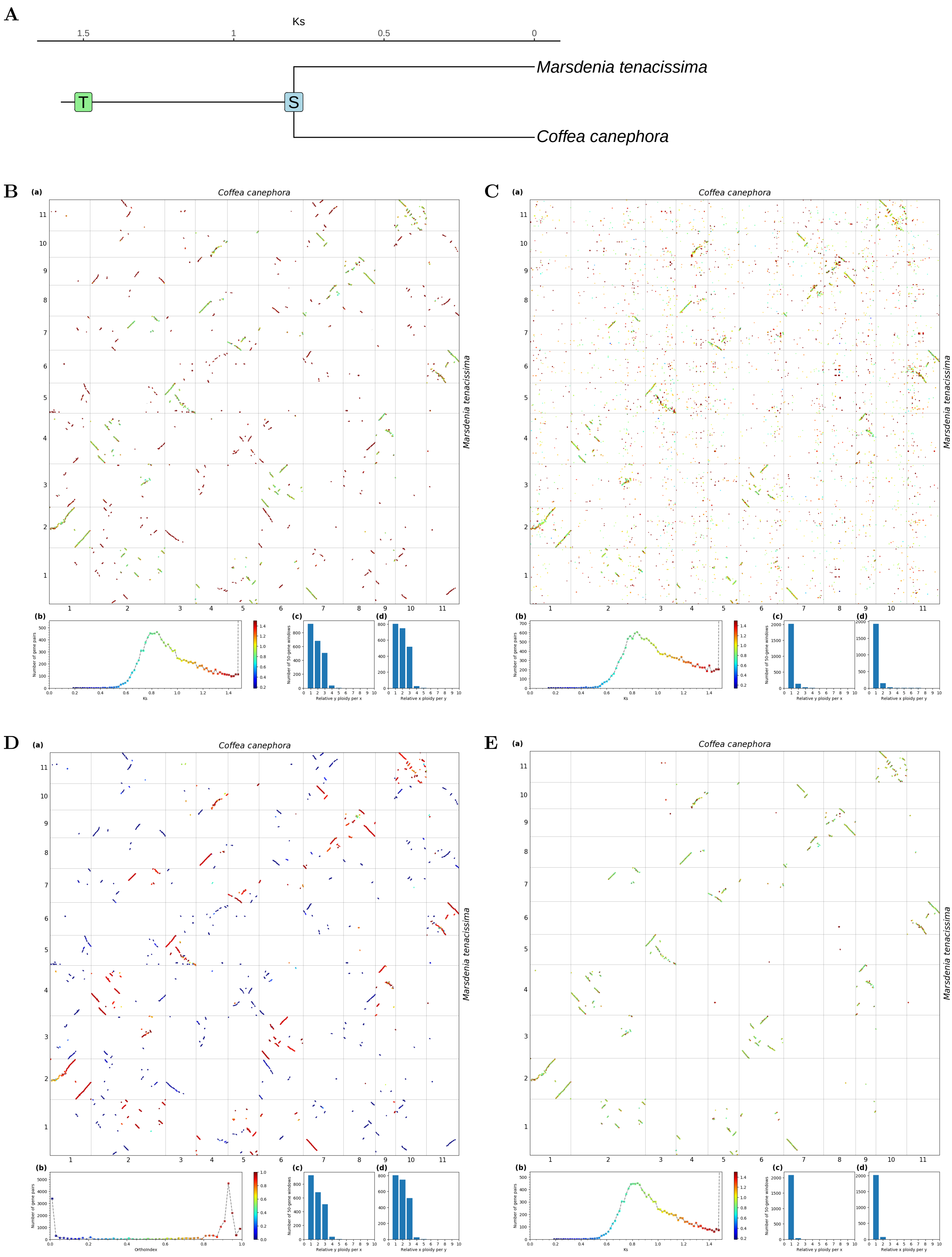

Figure S44. *Orthology Index* in the identification of orthologous synteny in *Coffea canephora* and *Marsdenia tenacissima*. Refer to **Fig.1** for detailed descriptions.

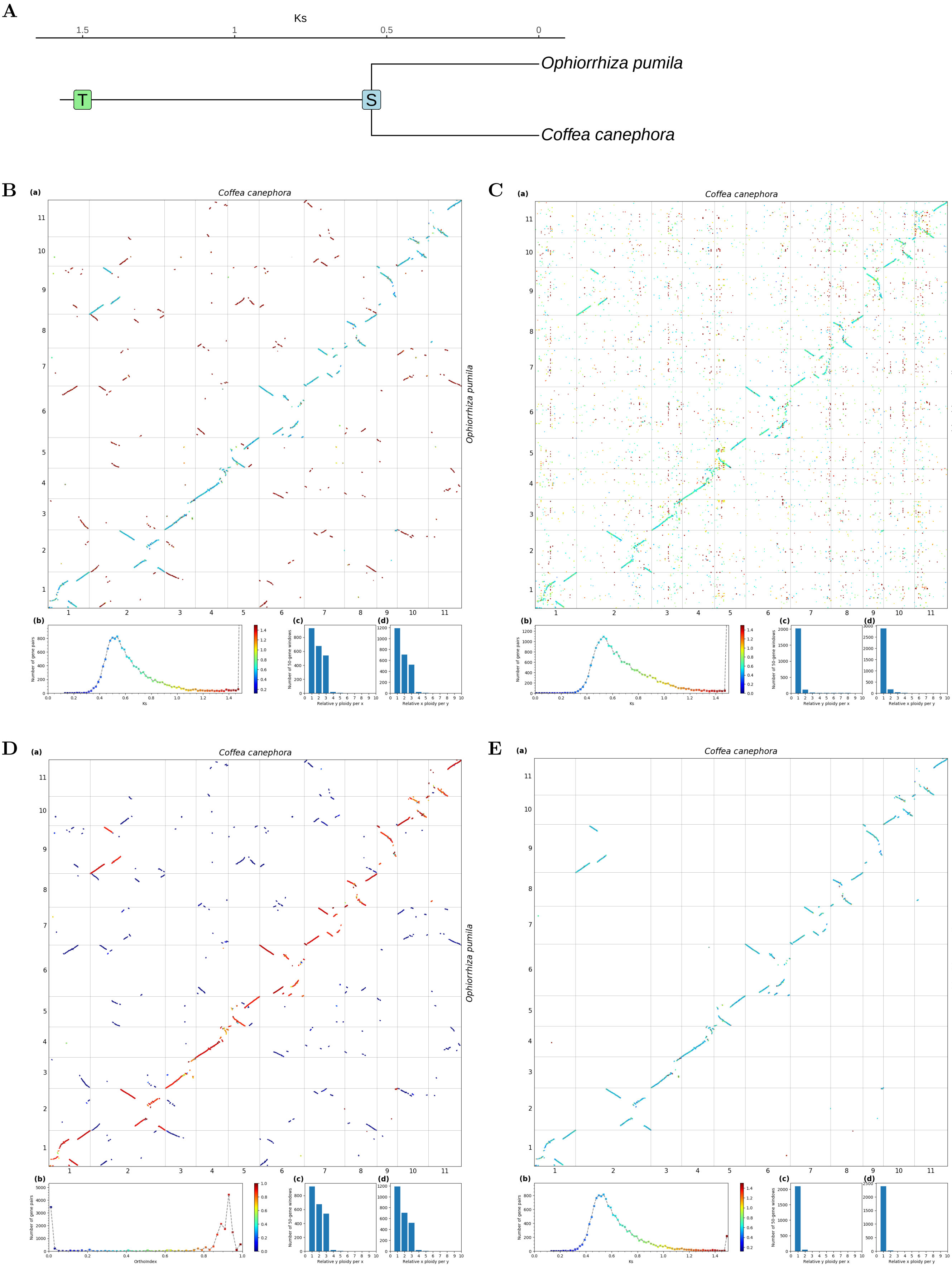

Figure S45. *Orthology Index* in the identification of orthologous synteny in *Coffea canephora* and *Ophiorrhiza pumila*. Refer to **Fig.1** for detailed descriptions.

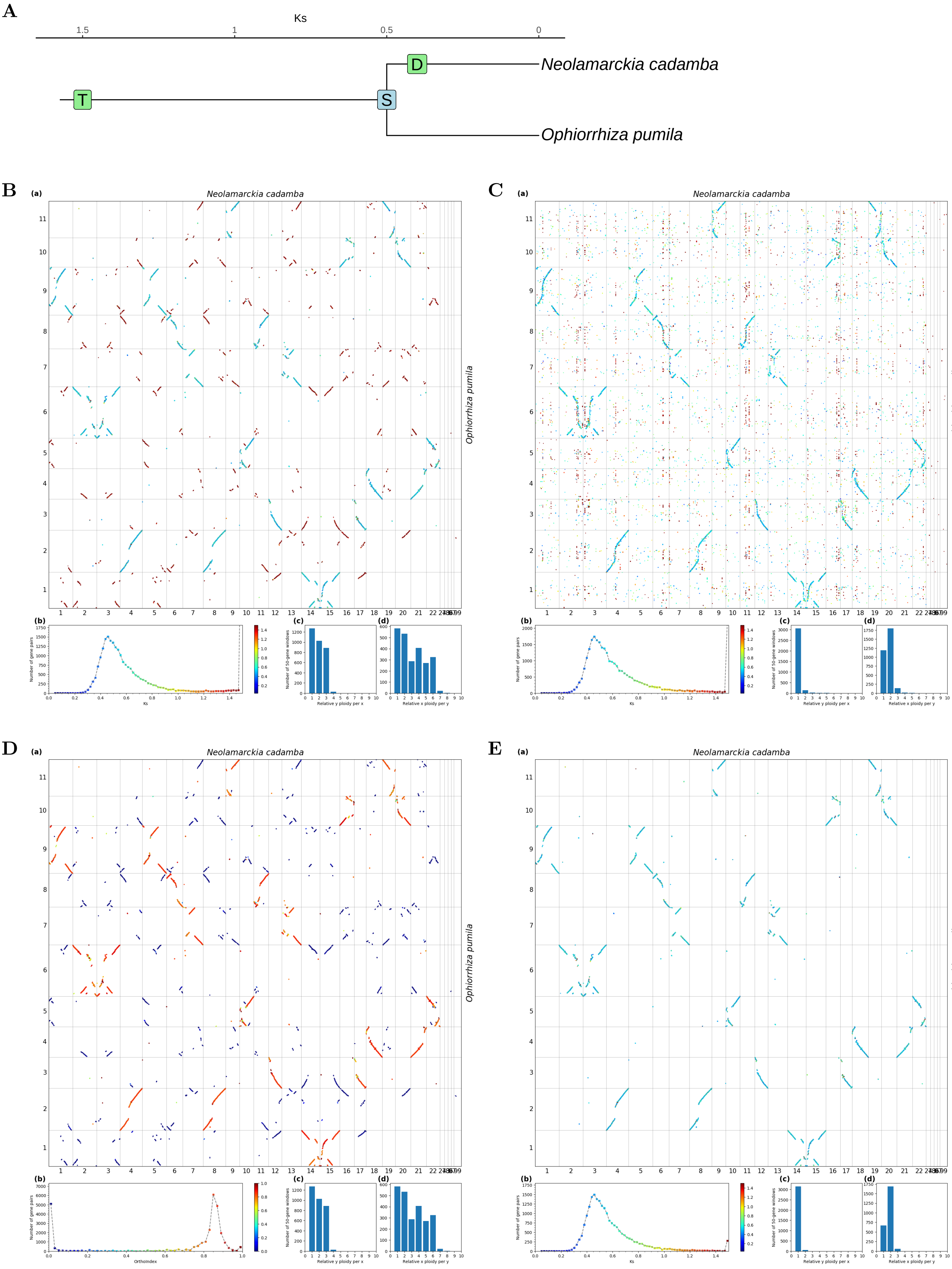

Figure S46. *Orthology Index* in the identification of orthologous synteny in *Ophiorrhiza pumila* and *Neolamarckia cadamba.* Refer to **Fig.1** for detailed descriptions.

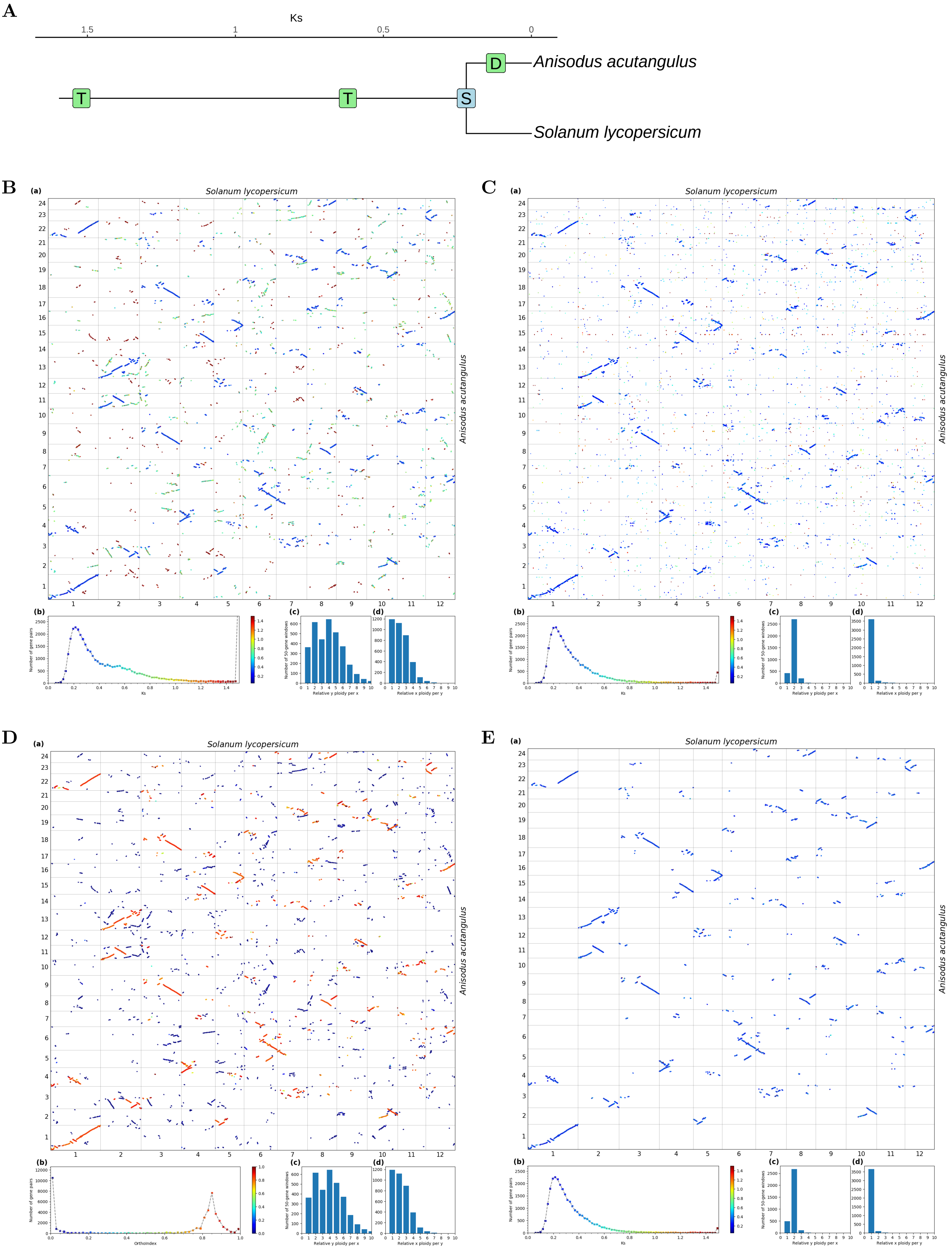

Figure S47. *Orthology Index* in the identification of orthologous synteny in *Solanum lycopersicum* and *Anisodus acutangulus*. Refer to **Fig.1** for detailed descriptions.

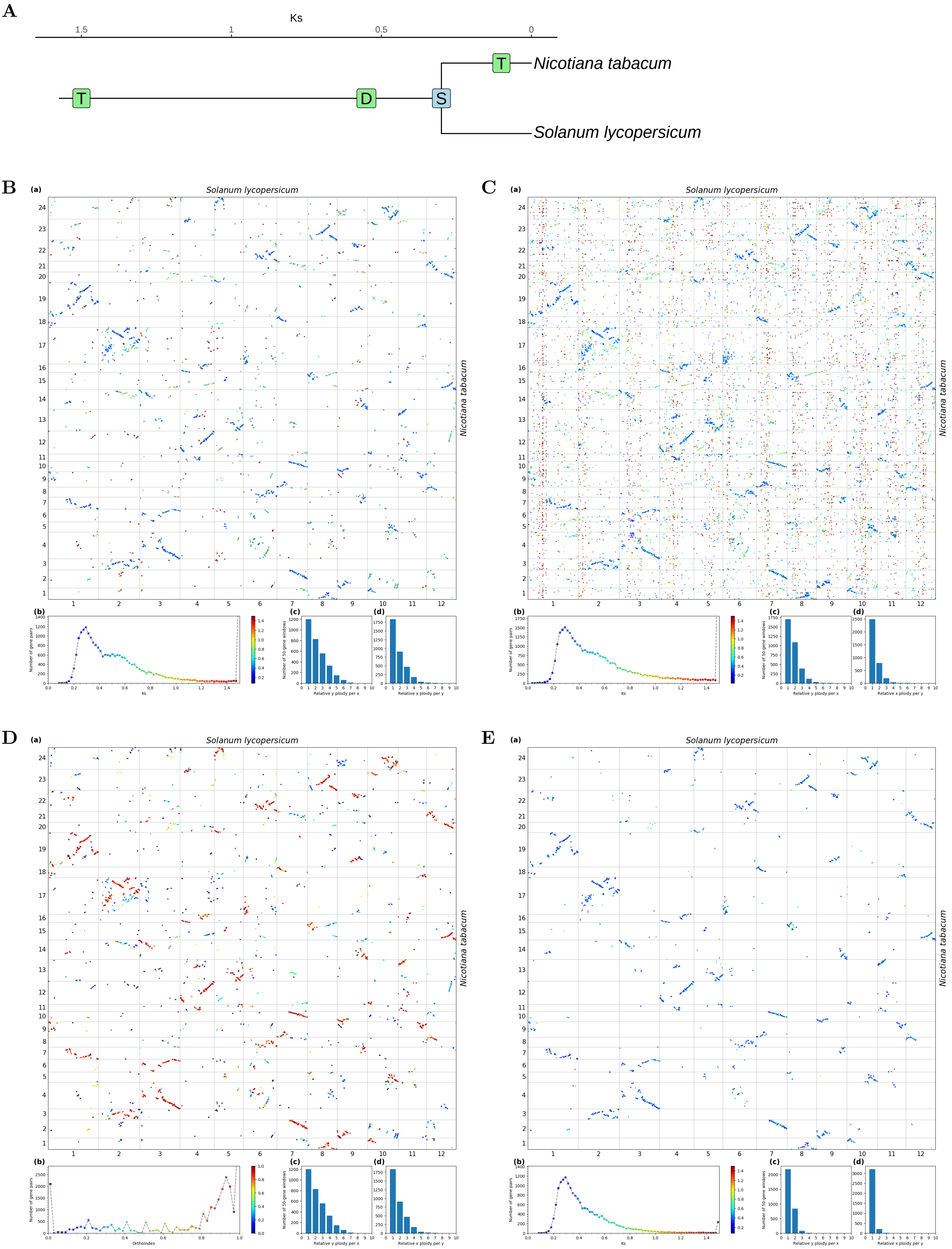

Figure S48. *Orthology Index* in the identification of orthologous synteny in *Solanum lycopersicum* and *Nicotiana tabacum*. Refer to **Fig.1** for detailed descriptions.

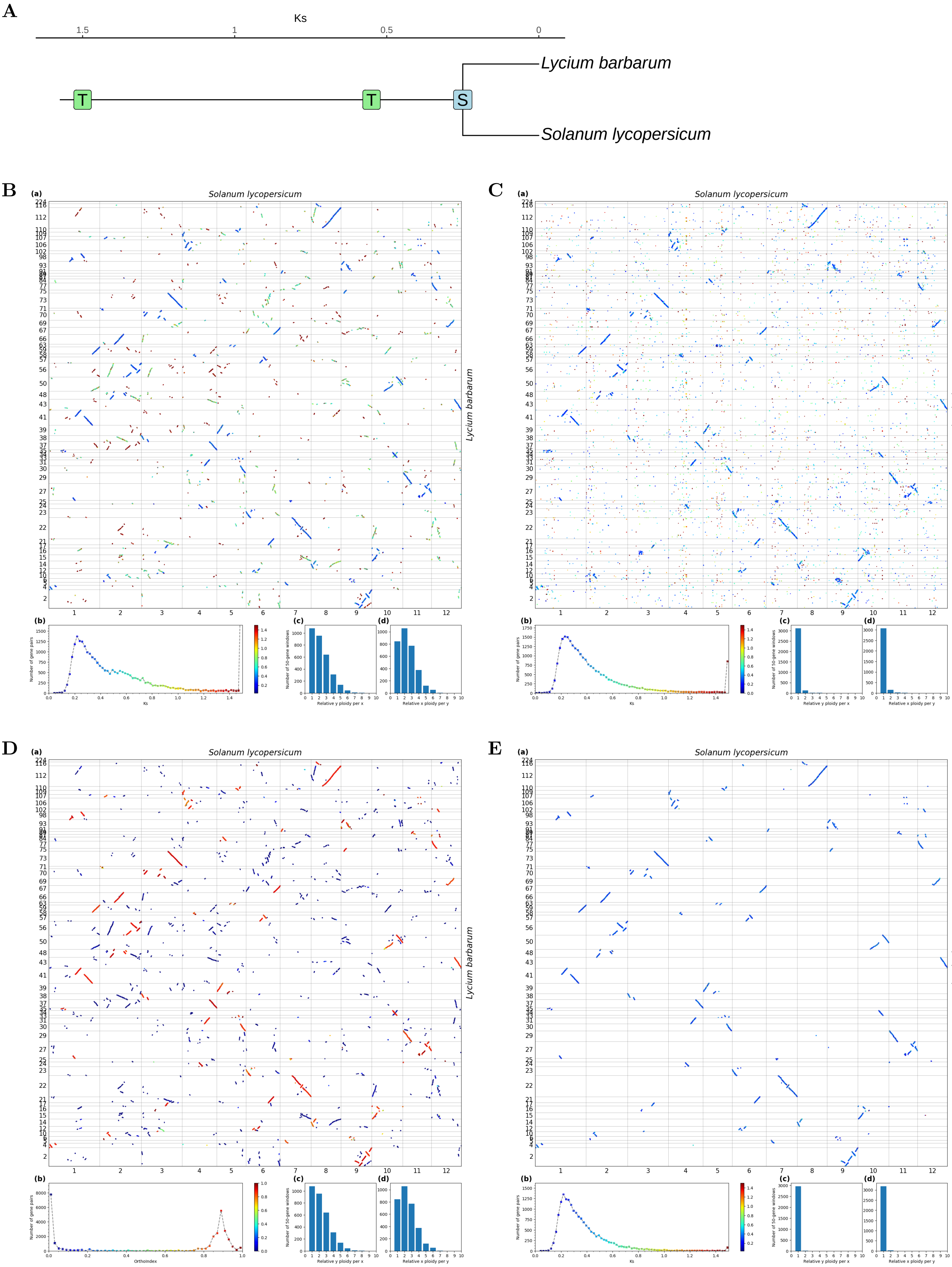

Figure S49. *Orthology Index* in the identification of orthologous synteny in *Solanum lycopersicum* and *Lycium barbarum*. Refer to **Fig.1** for detailed descriptions.

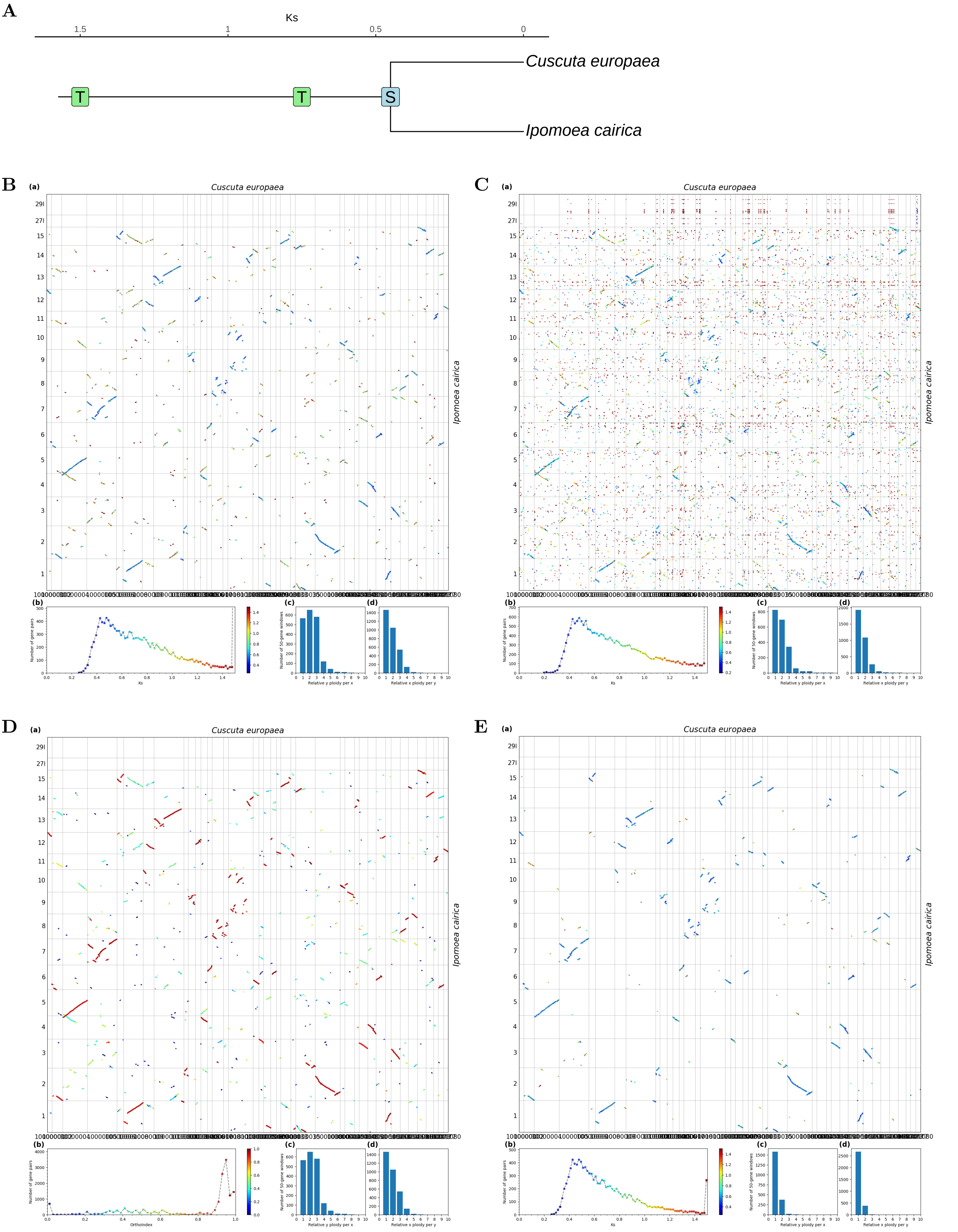

Figure S50. *Orthology Index* in the identification of orthologous synteny in *Ipomoea cairica* and *Cuscuta europaea*. Refer to **Fig.1** for detailed descriptions.

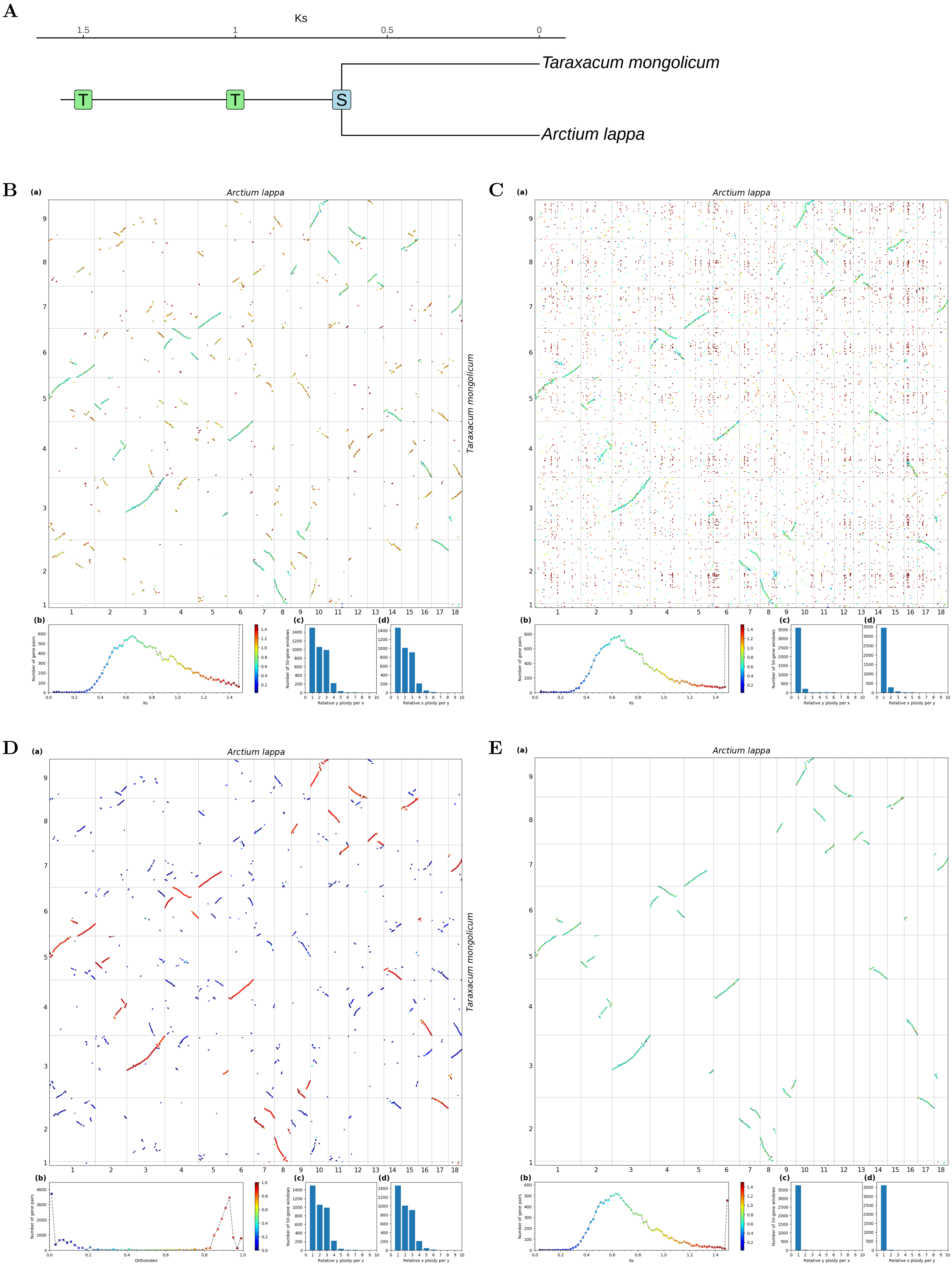

Figure S51. *Orthology Index* in the identification of orthologous synteny in *Arctium lappa* and *Taraxacum mongolicum*. Refer to **Fig.1** for detailed descriptions.

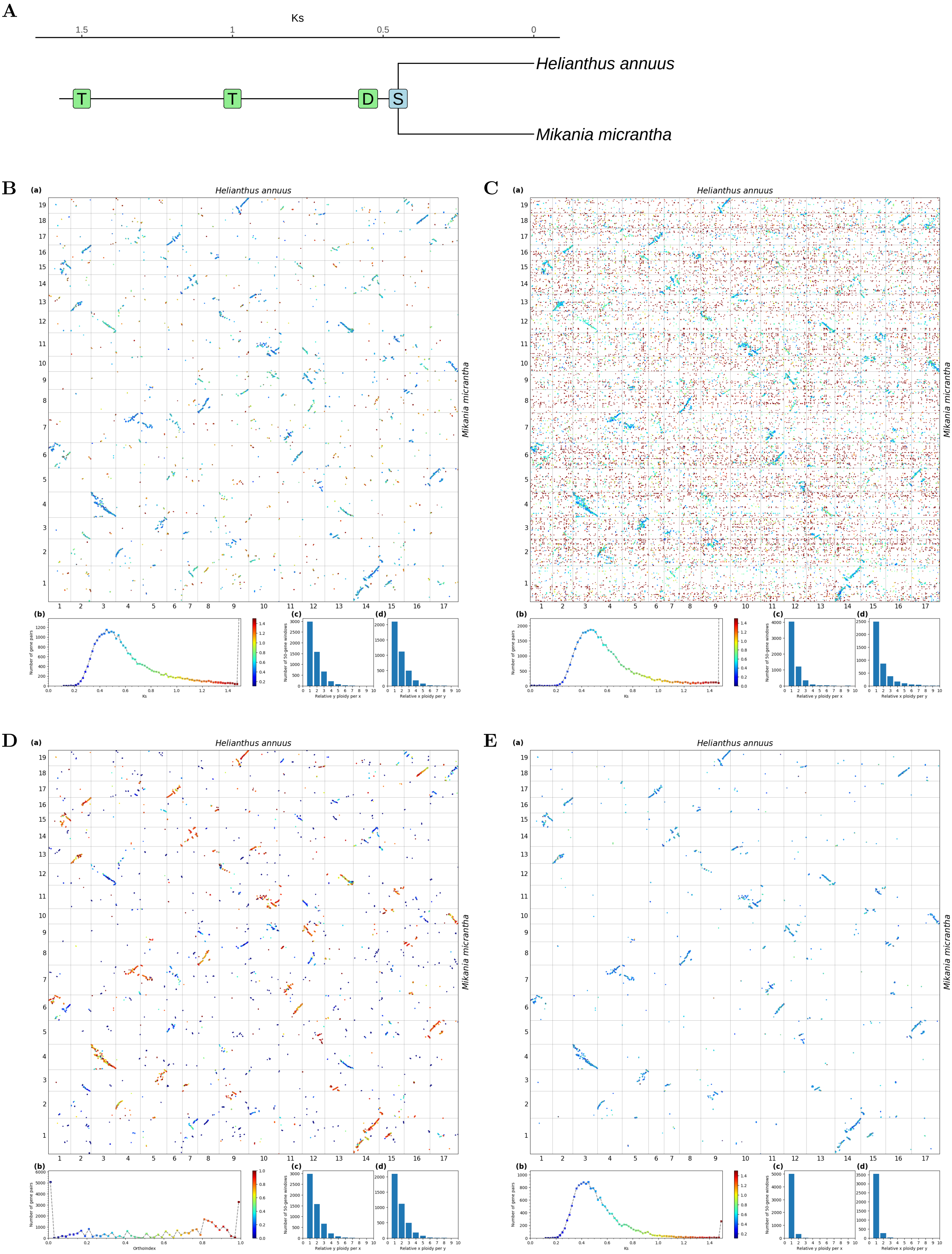

Figure S52. *Orthology Index* in the identification of orthologous synteny in *Mikania micrantha* and *Helianthus annuus*. Refer to **Fig.1** for detailed descriptions.

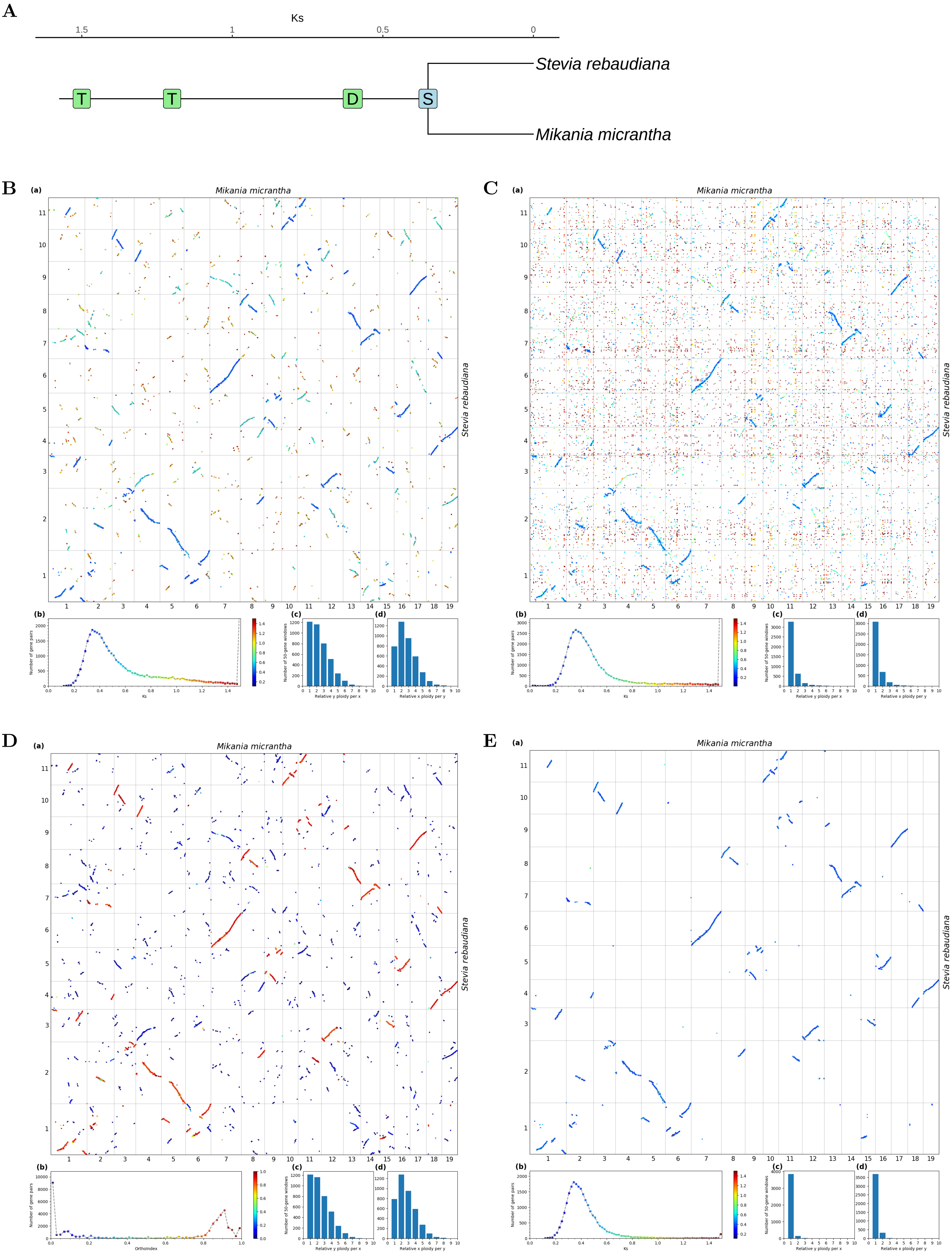

Figure S53. *Orthology Index* in the identification of orthologous synteny in *Mikania micrantha* and *Stevia rebaudiana*. Refer to **Fig.1** for detailed descriptions.

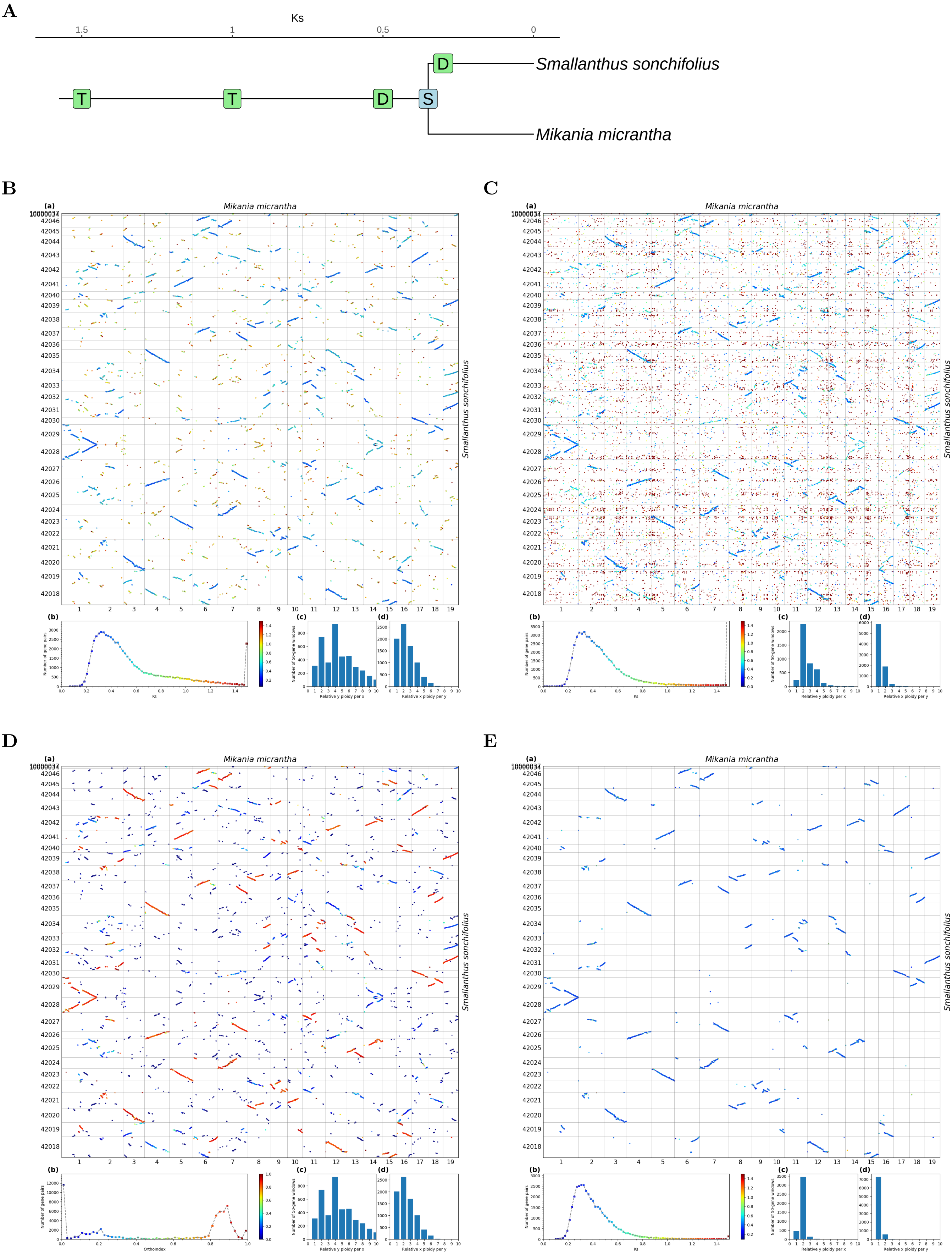

Figure S54. *Orthology Index* in the identification of orthologous synteny in *Mikania micrantha* and *Smallanthus sonchifolius*. Refer to **Fig.1** for detailed descriptions.

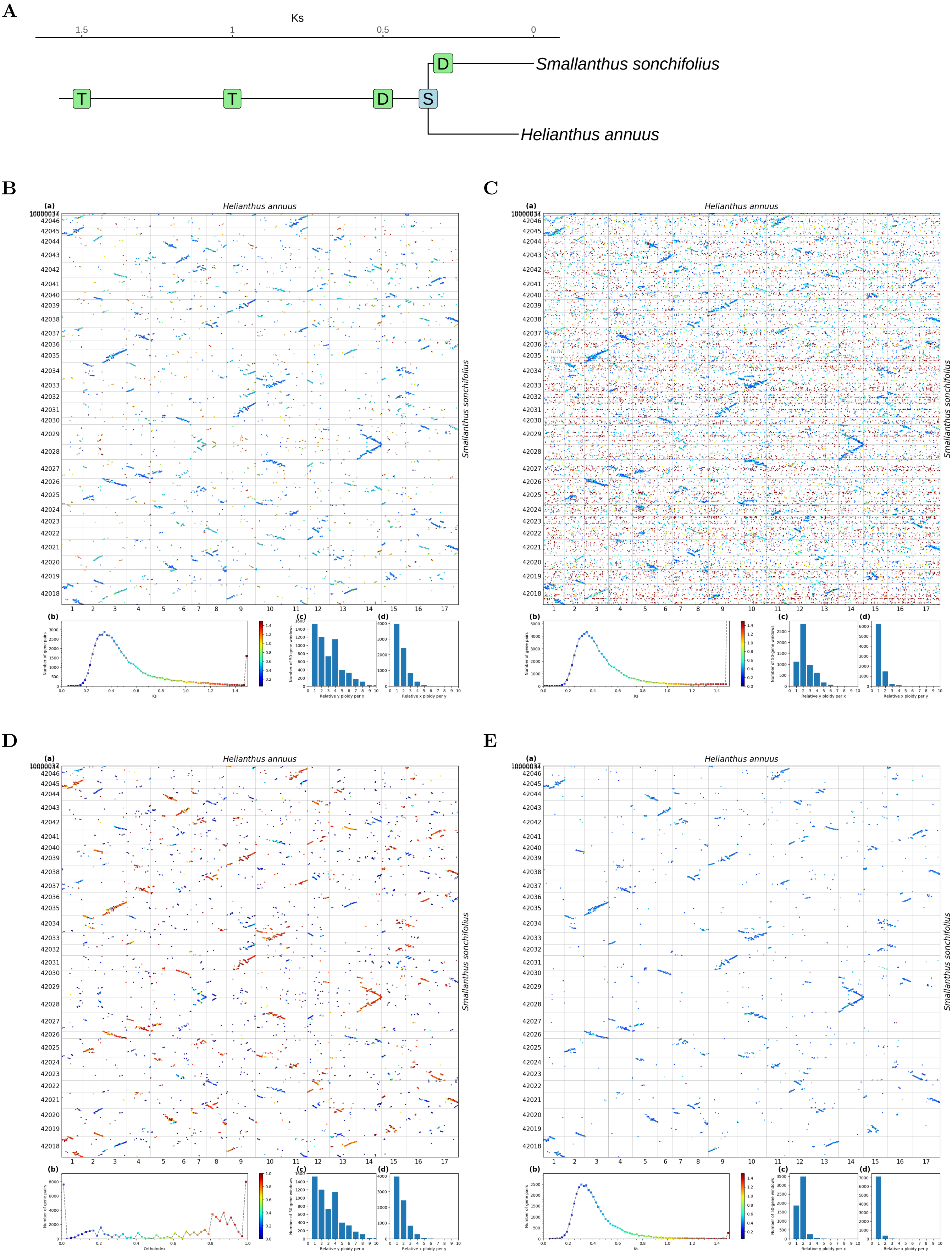

Figure S55. *Orthology Index* in the identification of orthologous synteny in *Helianthus annuus* and *Smallanthus sonchifolius*. Refer to **Fig.1** for detailed descriptions.

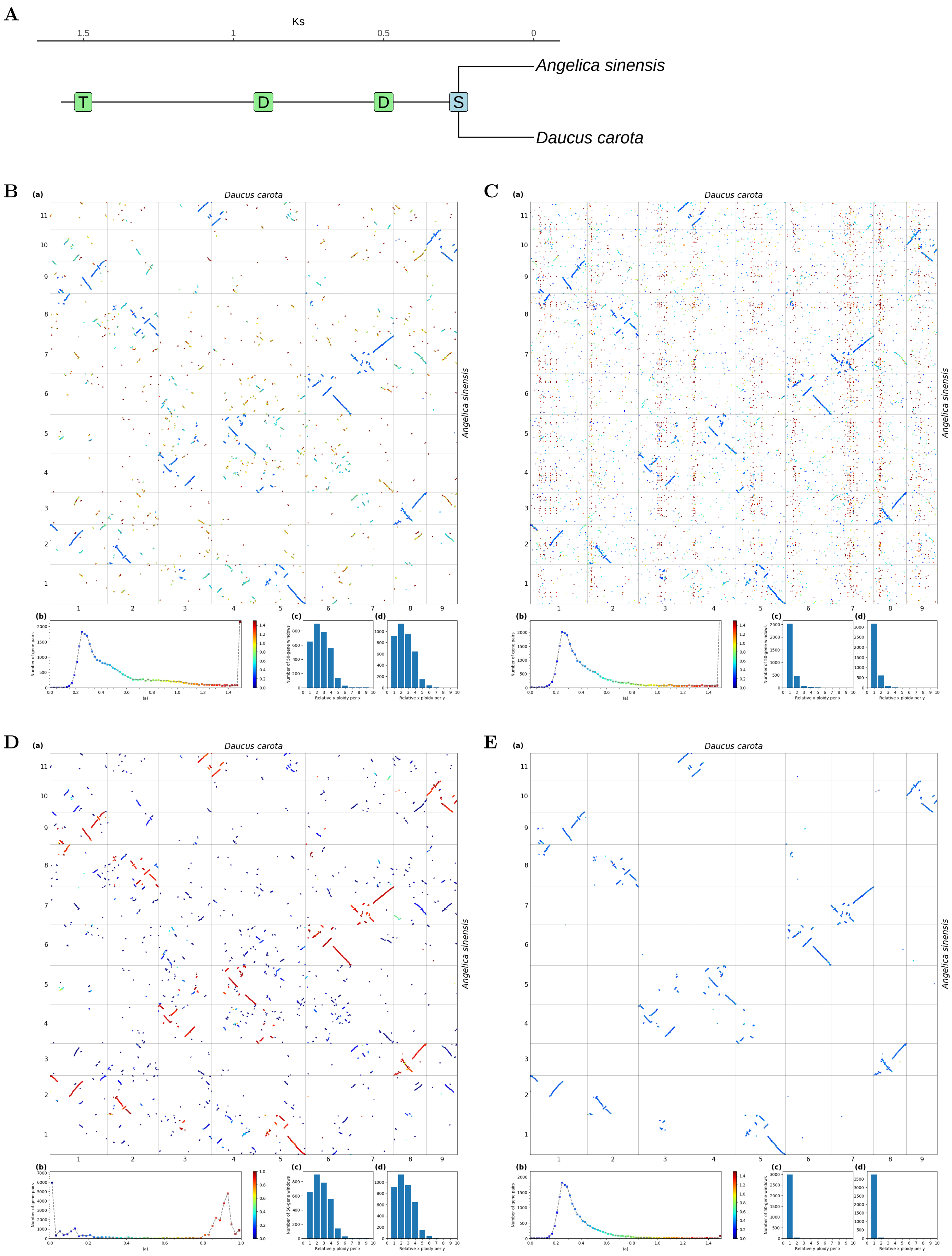

Figure S56. *Orthology Index* in the identification of orthologous synteny in *Daucus carota* and *Angelica sinensis*. Refer to **Fig.1** for detailed descriptions.

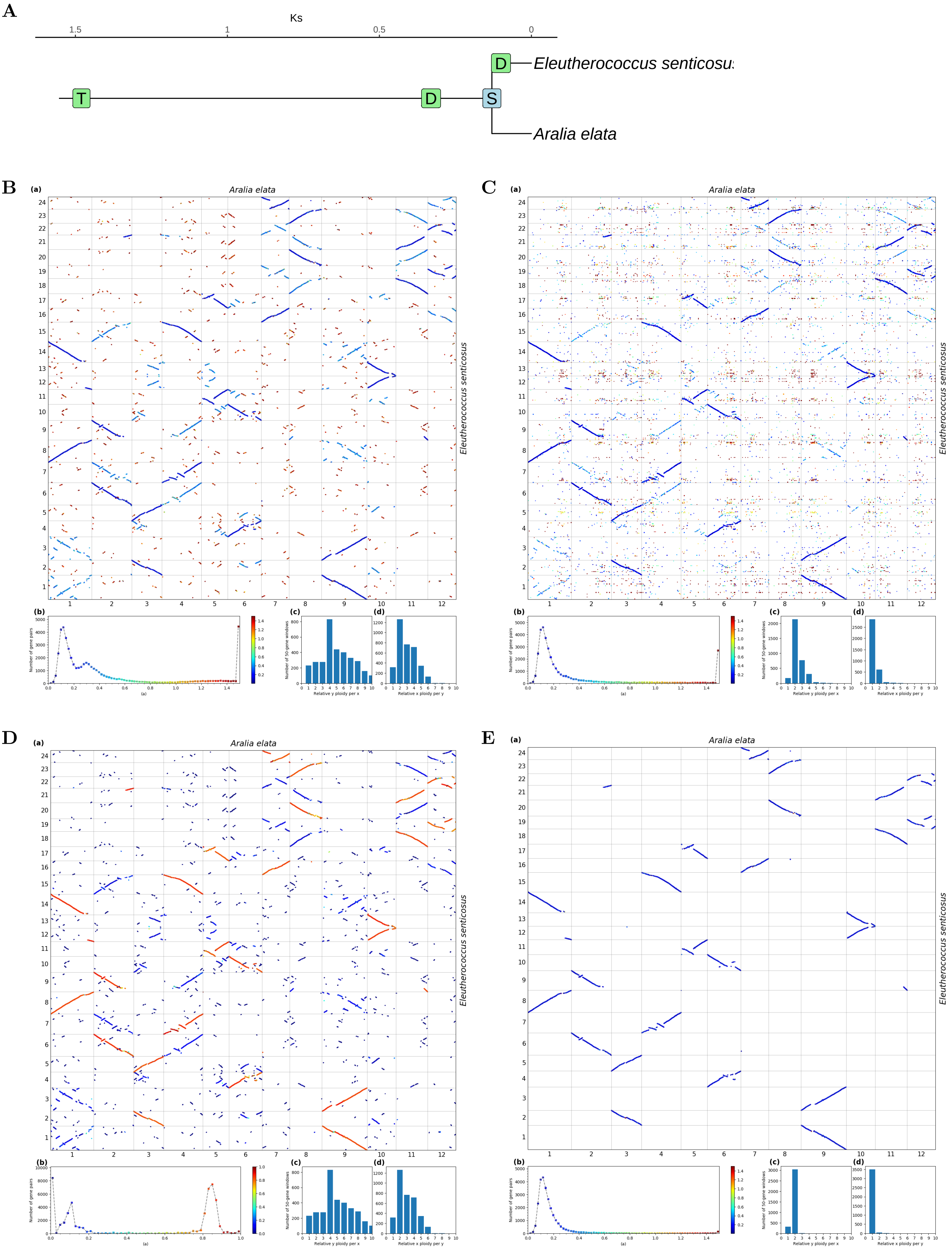

Figure S57. *Orthology Index* in the identification of orthologous synteny in *Aralia elata* and *Eleutherococcus senticosus*. Refer to **Fig.1** for detailed descriptions.

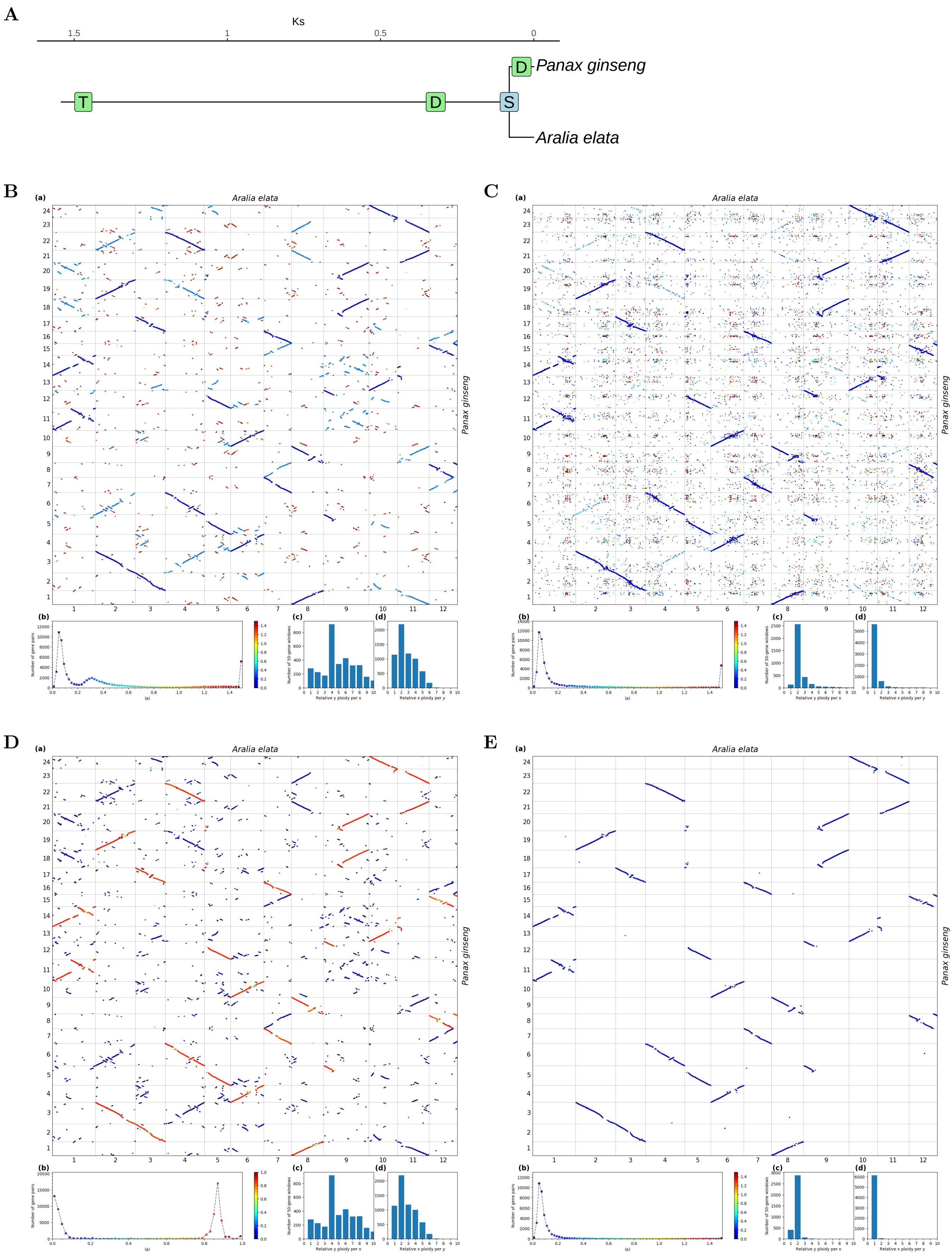

Figure S58. *Orthology Index* in the identification of orthologous synteny in *Aralia elata* and *Panax ginseng*. Refer to **Fig.1** for detailed descriptions.

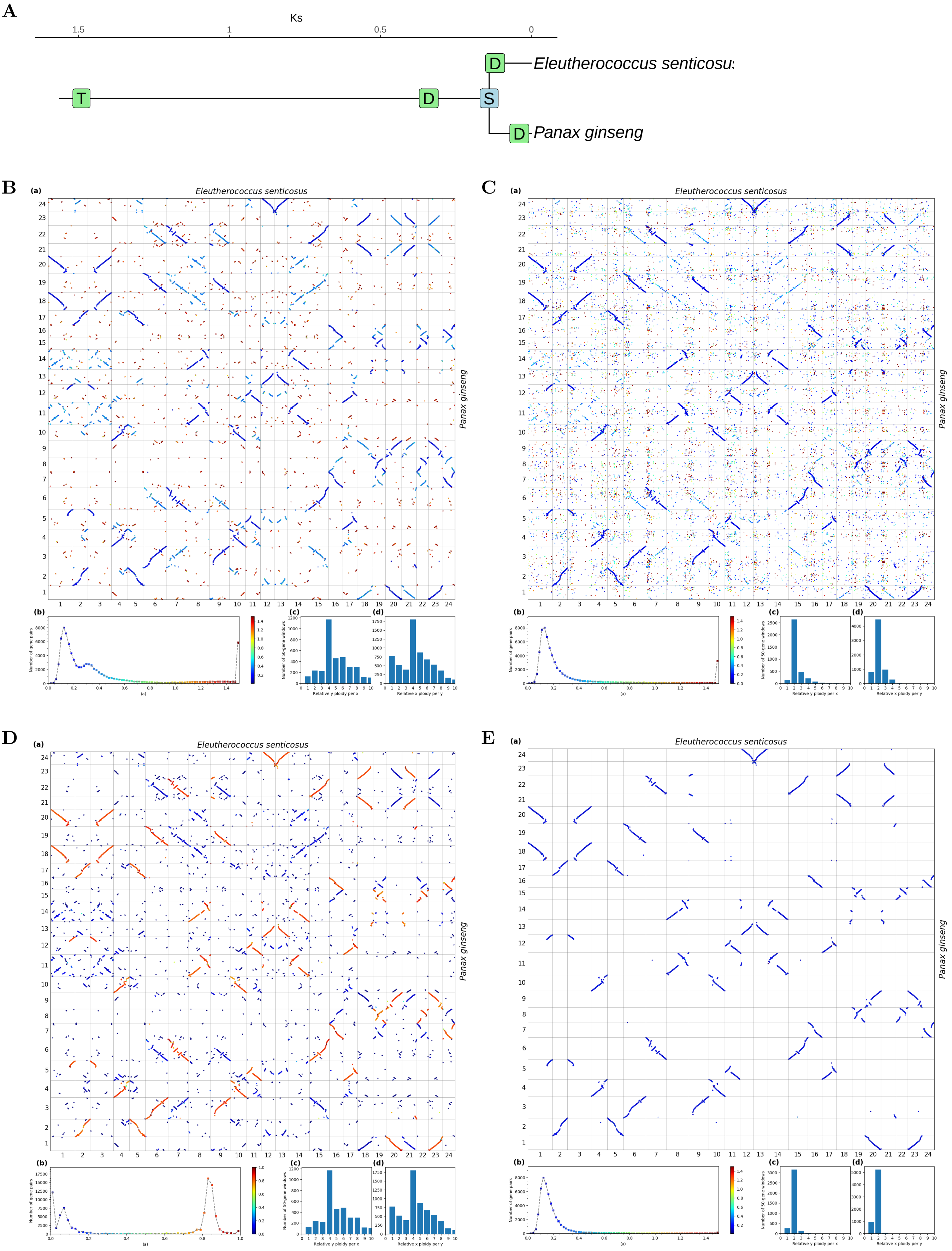

Figure S59. *Orthology Index* in the identification of orthologous synteny in *Panax ginseng* and *Eleutherococcus senticosus*. Refer to **Fig.1** for detailed descriptions.

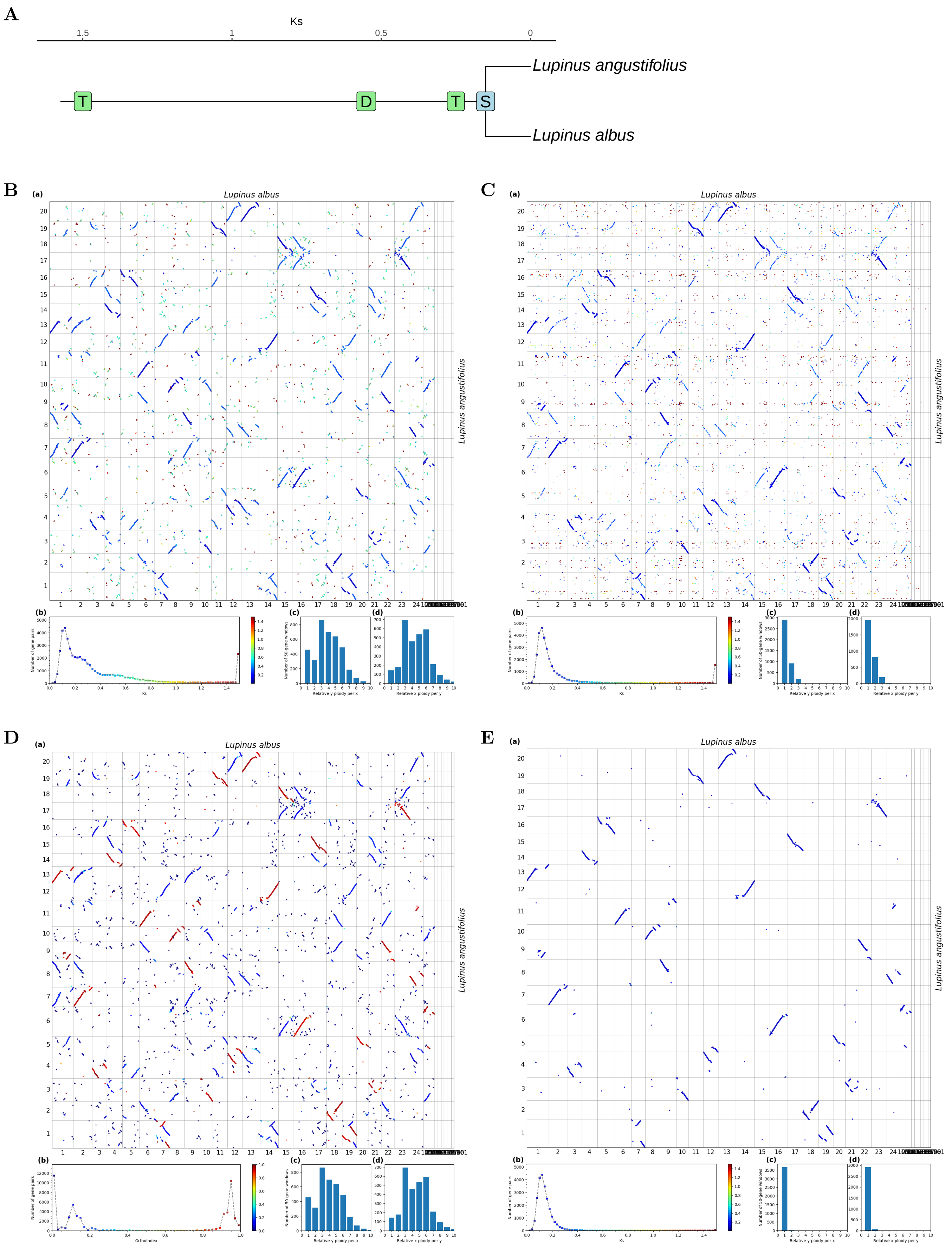

Figure S60. *Orthology Index* in the identification of orthologous synteny in *Lupinus albus* and *Lupinus angustifolius*. Refer to **Fig.1** for detailed descriptions.

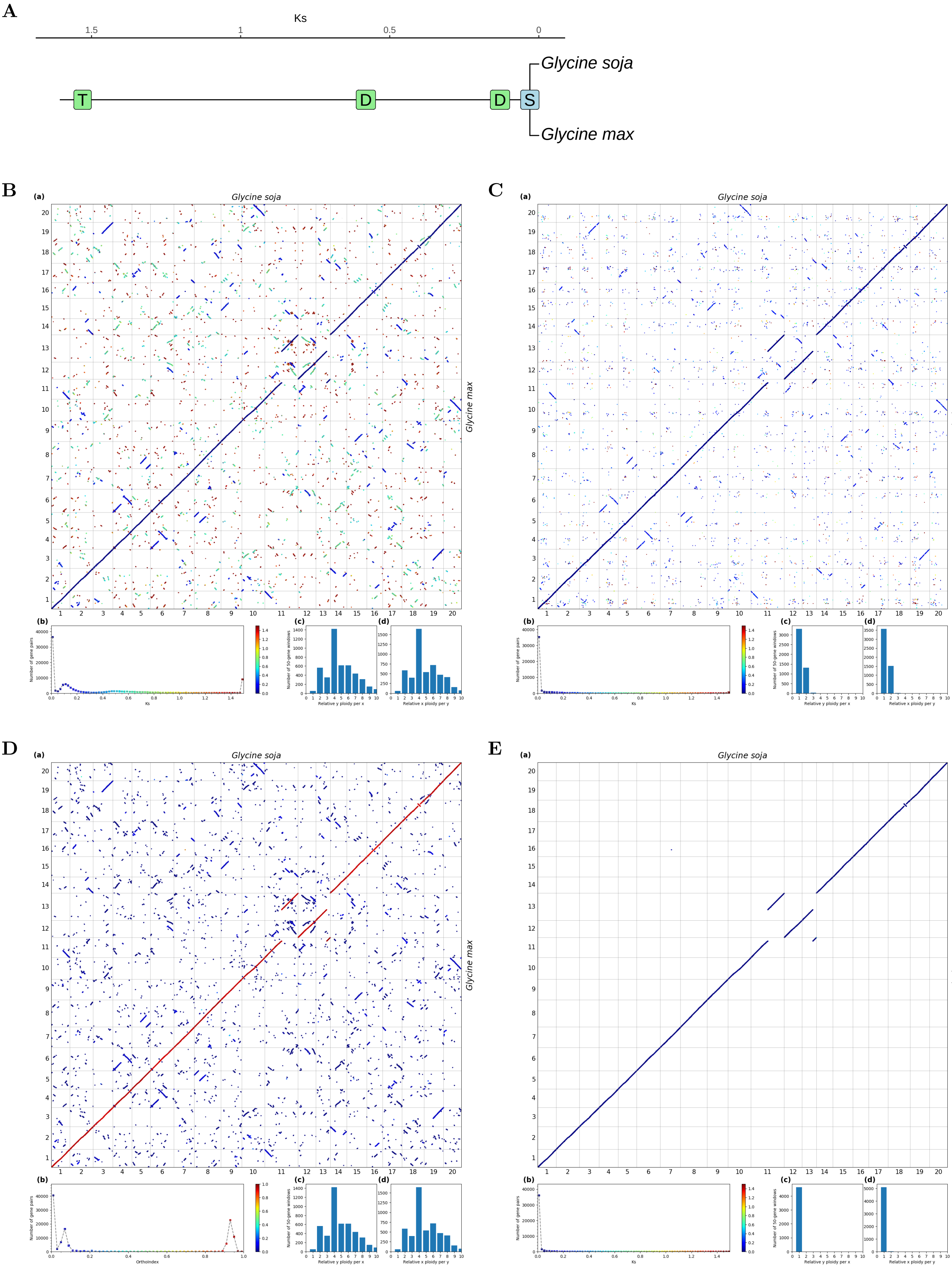

Figure S61. *Orthology Index* in the identification of orthologous synteny in *Glycine max* and *Glycine soja*. Refer to **Fig.1** for detailed descriptions.

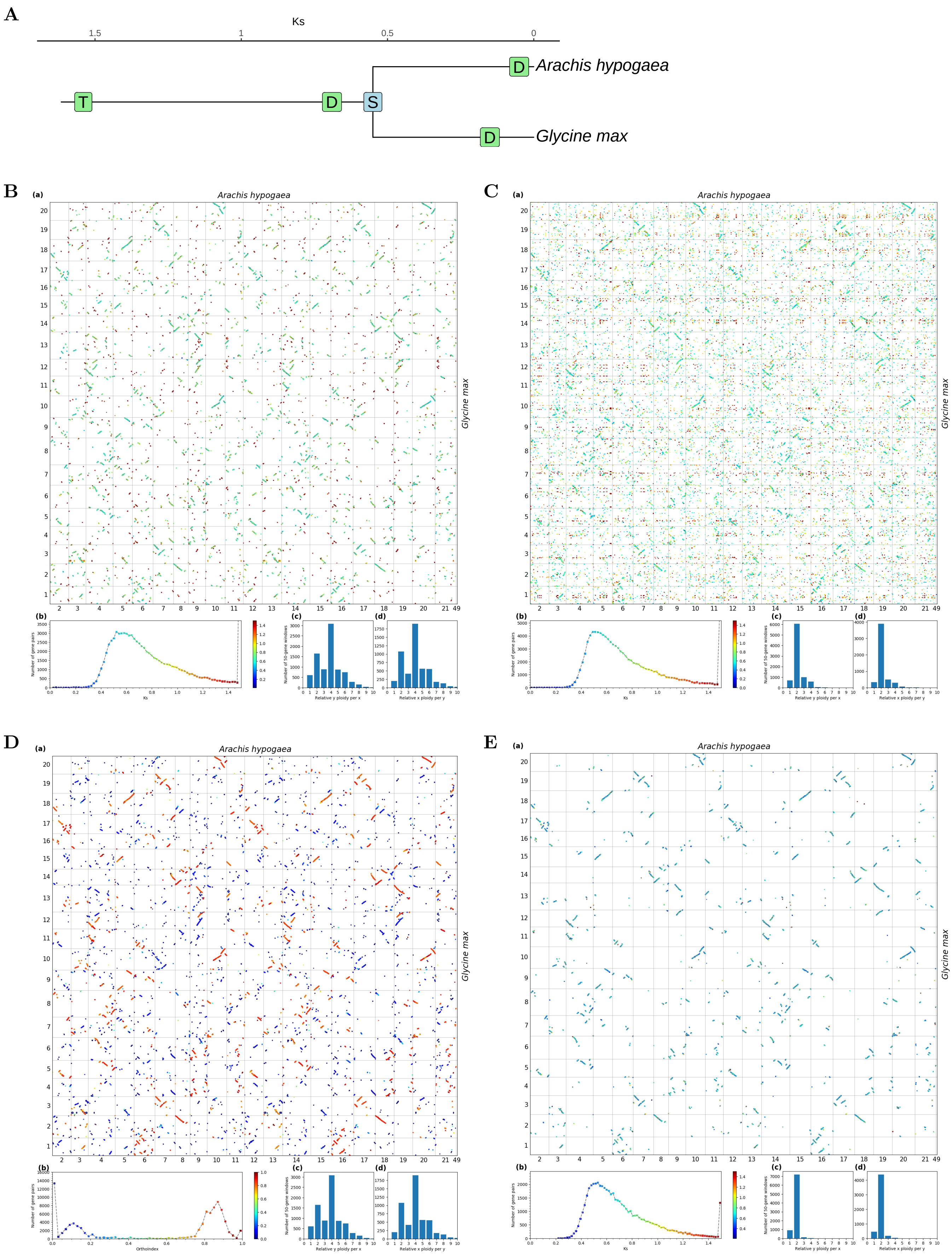

Figure S62. *Orthology Index* in the identification of orthologous synteny in *Glycine max* and *Arachis hypogaea*. Refer to **Fig.1** for detailed descriptions.

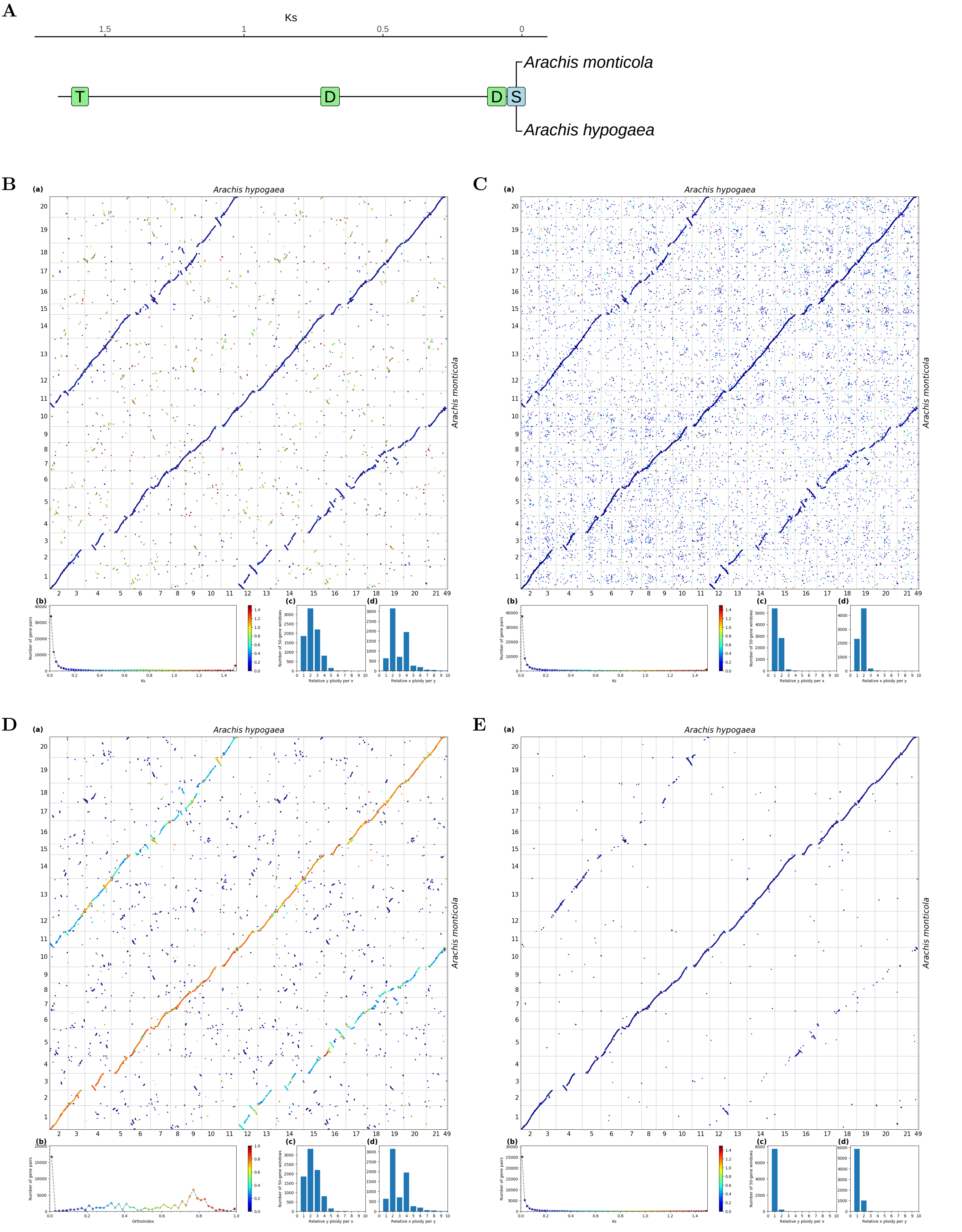

Figure S63. *Orthology Index* in the identification of orthologous synteny in *Arachis hypogaea* and *Arachis monticola*. Refer to **Fig.1** for detailed descriptions.

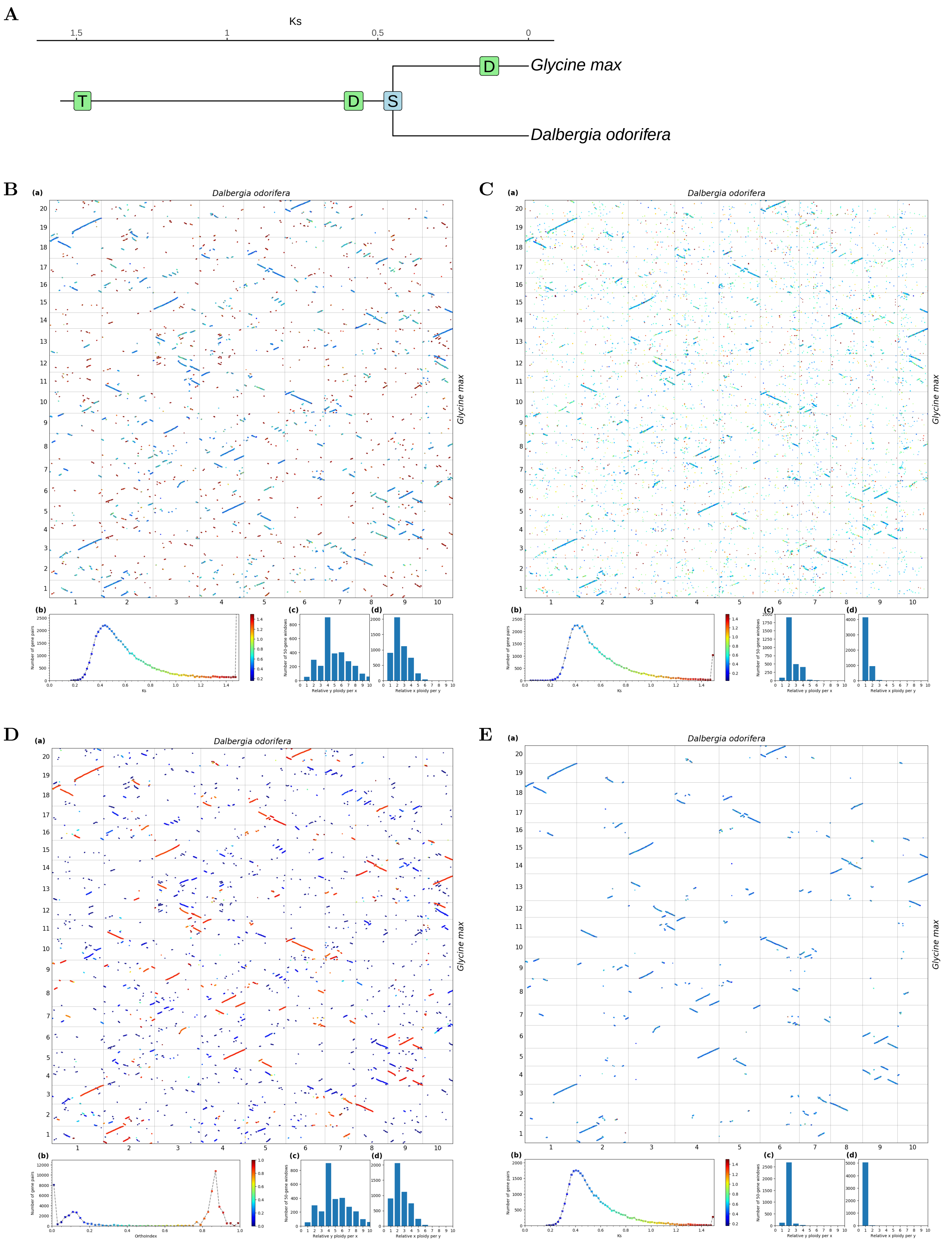

Figure S64. *Orthology Index* in the identification of orthologous synteny in *Dalbergia odorifera* and *Glycine max.* Refer to **Fig.1** for detailed descriptions.

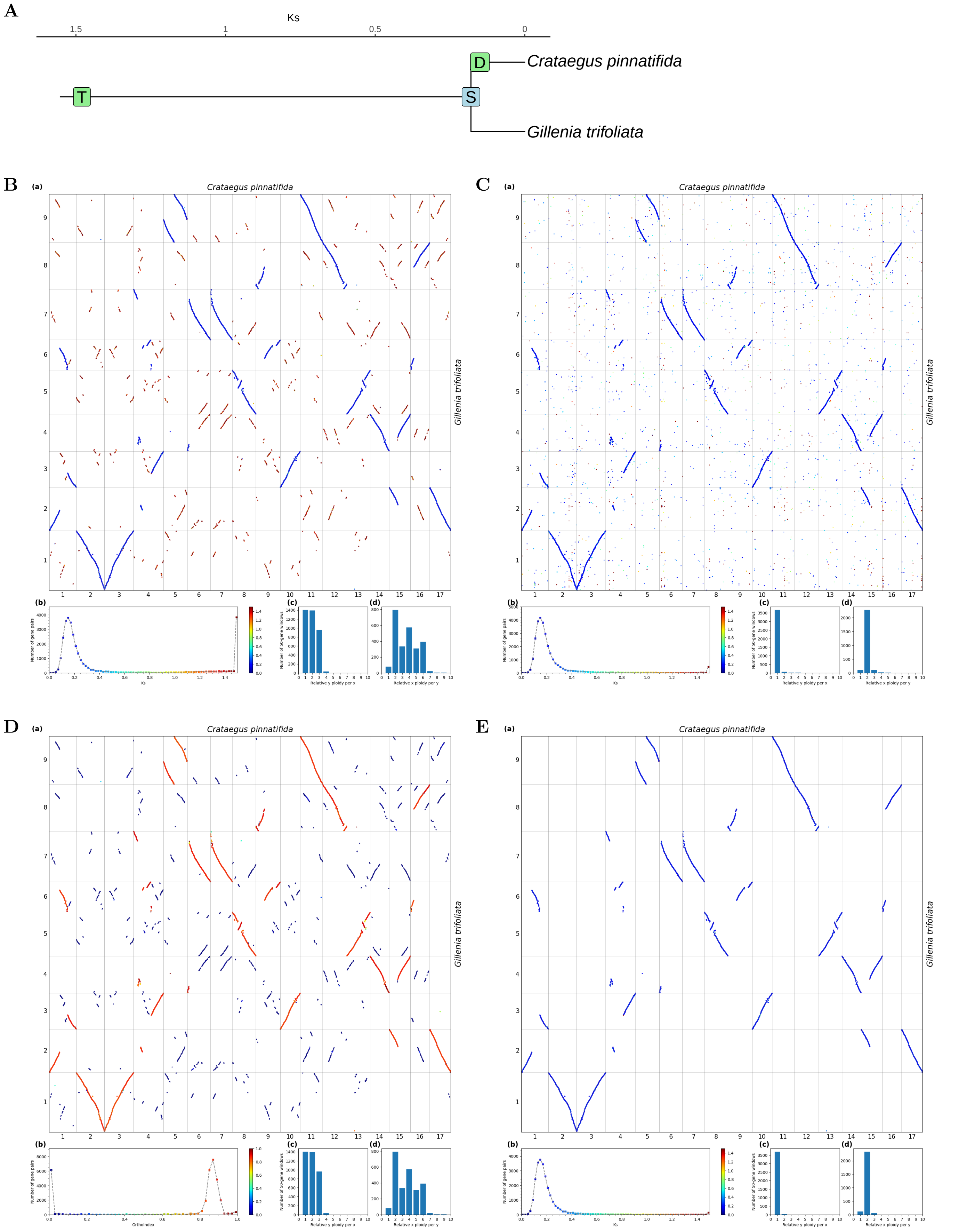

Figure S65. *Orthology Index* in the identification of orthologous synteny in *Gillenia trifoliata* and *Crataegus pinnatifida*. Refer to **Fig.1** for detailed descriptions.

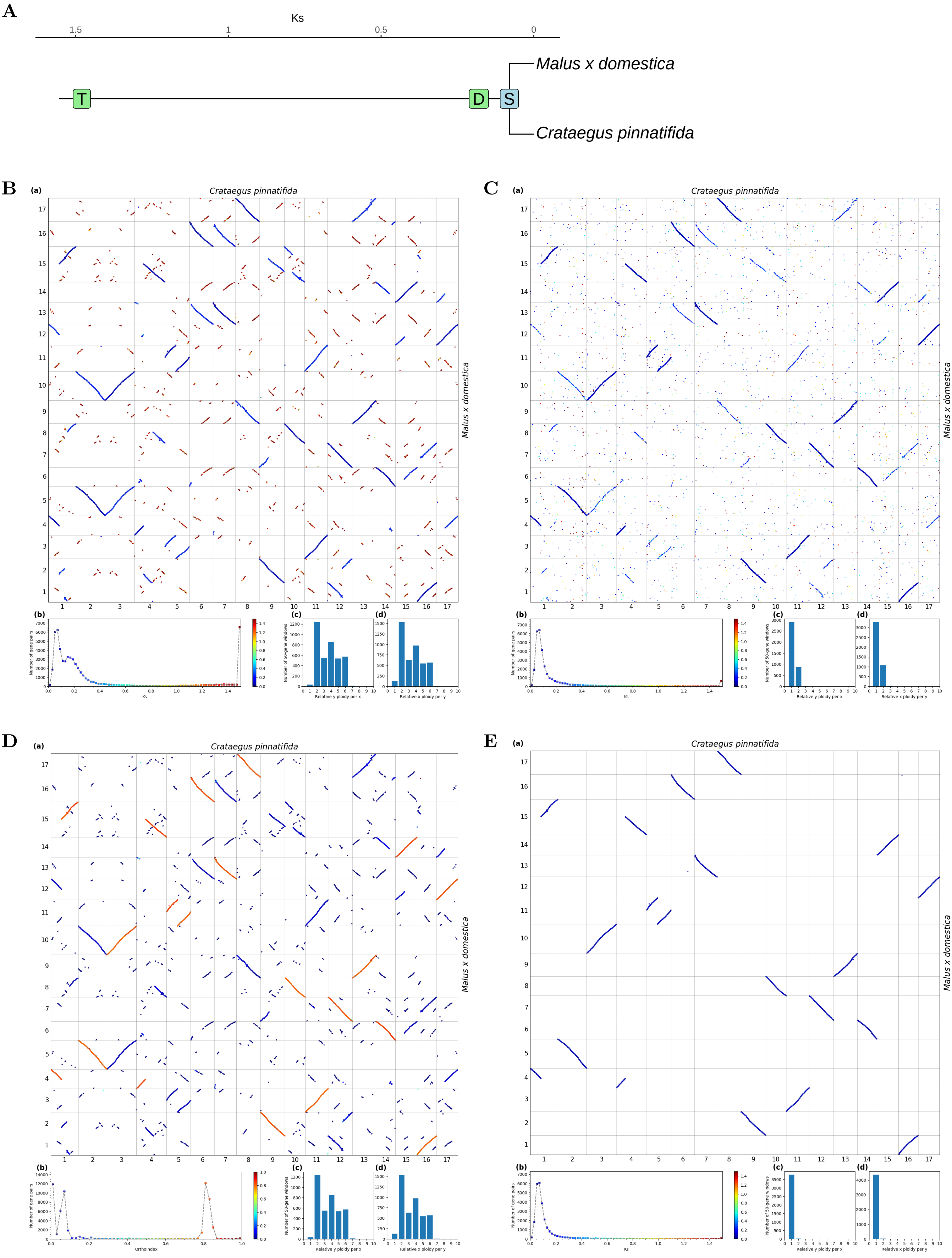

Figure S66. *Orthology Index* in the identification of orthologous synteny in *Crataegus pinnatifida* and *Malus x domestica*. Refer to **Fig.1** for detailed descriptions.

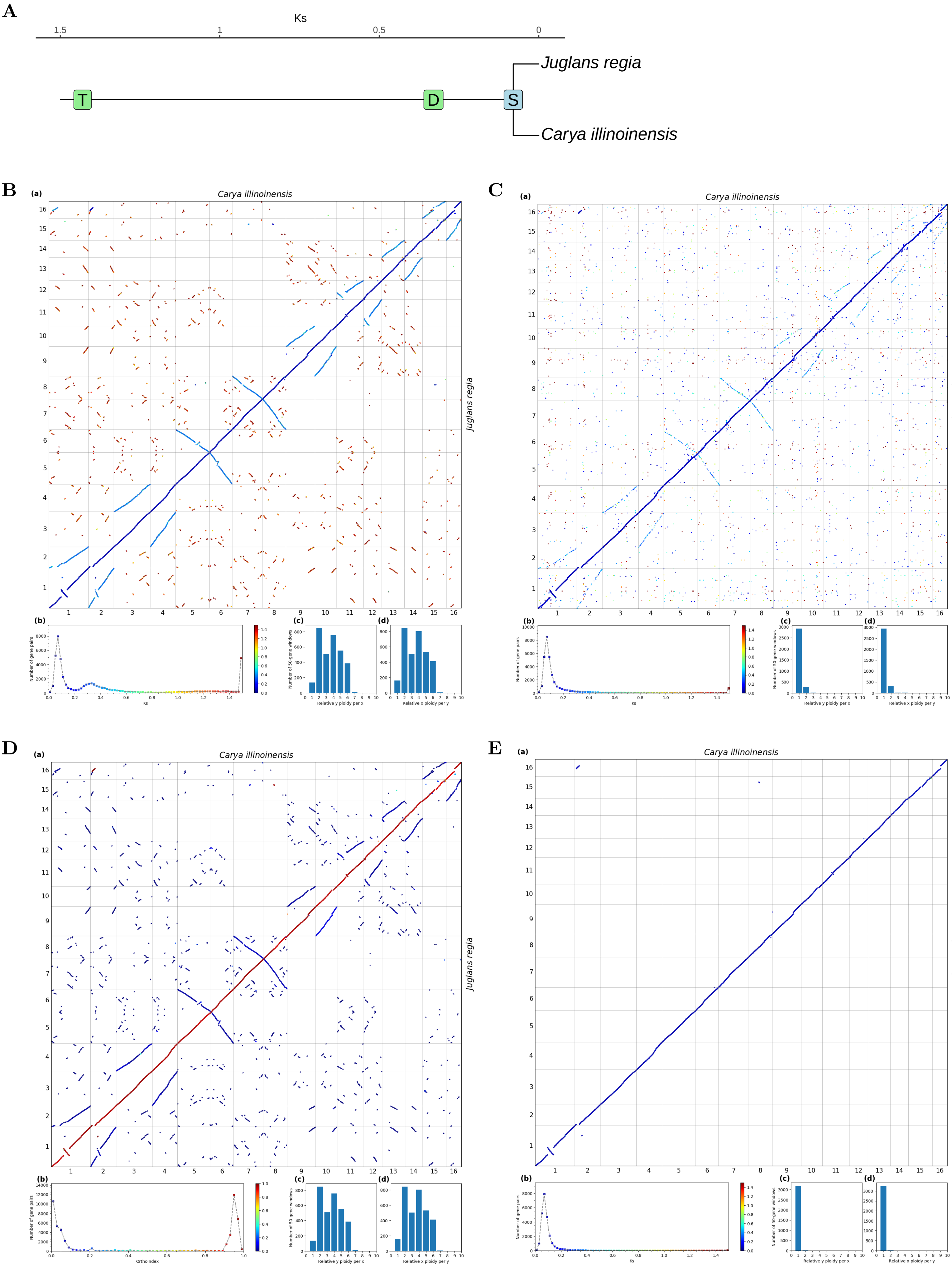

Figure S67. *Orthology Index* in the identification of orthologous synteny in *Carya illinoinensis* and *Juglans regia*. Refer to **Fig.1** for detailed descriptions.

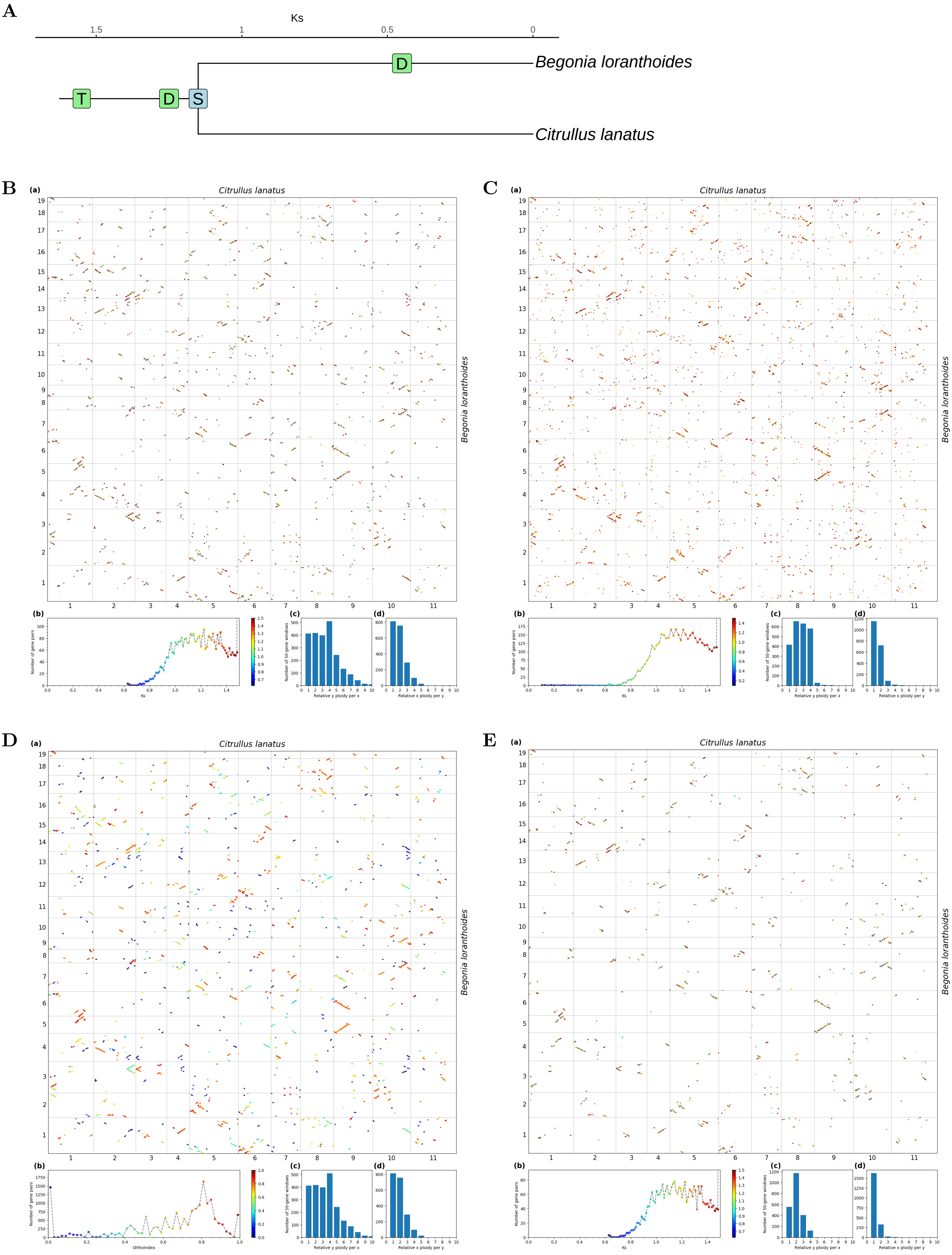

Figure S68. *Orthology Index* in the identification of orthologous synteny in *Citrullus lanatus* and *Begonia loranthoides*. Refer to **Fig.1** for detailed descriptions.

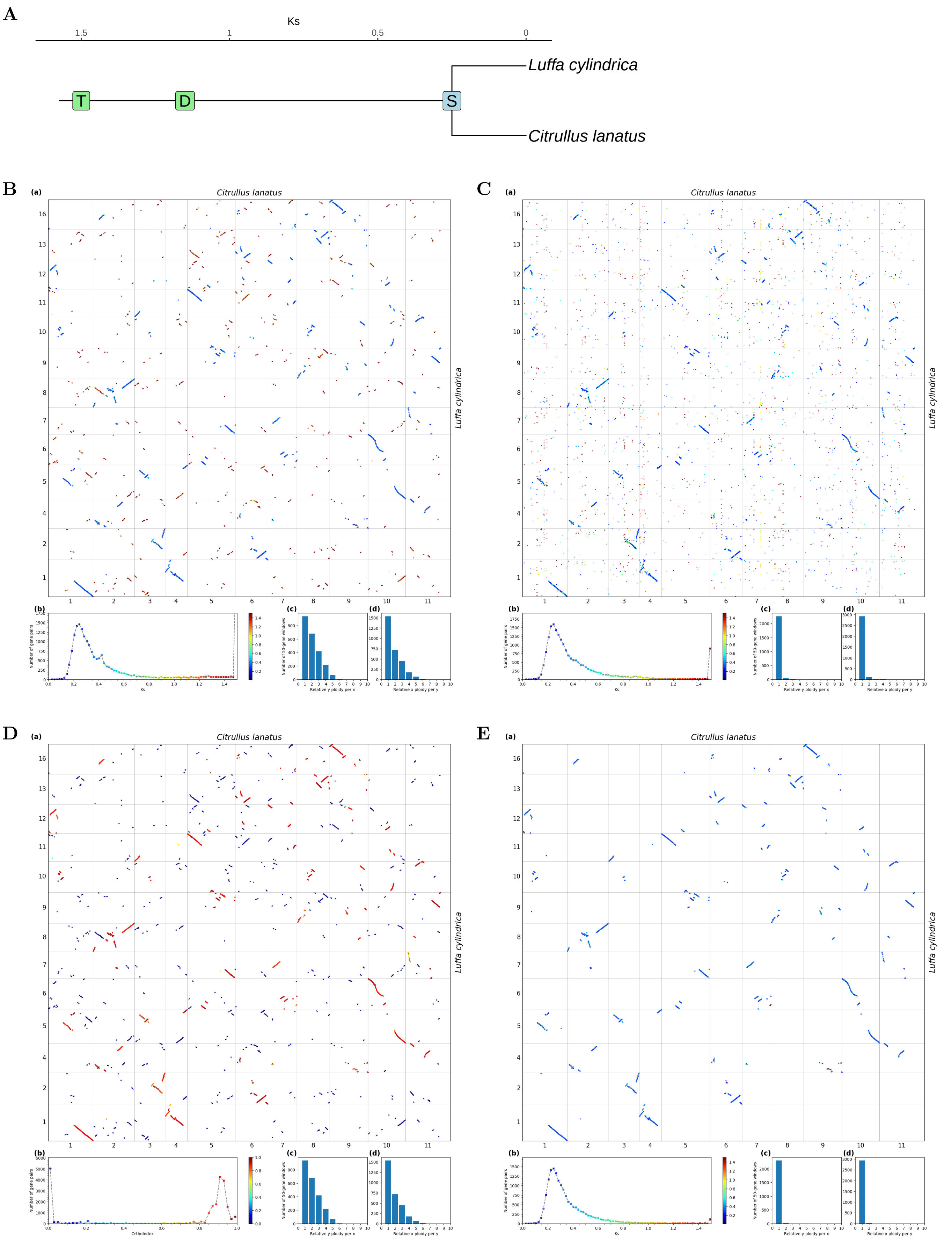

Figure S69. *Orthology Index* in the identification of orthologous synteny in *Citrullus lanatus* and *Luffa cylindrica*. Refer to **Fig.1** for detailed descriptions.

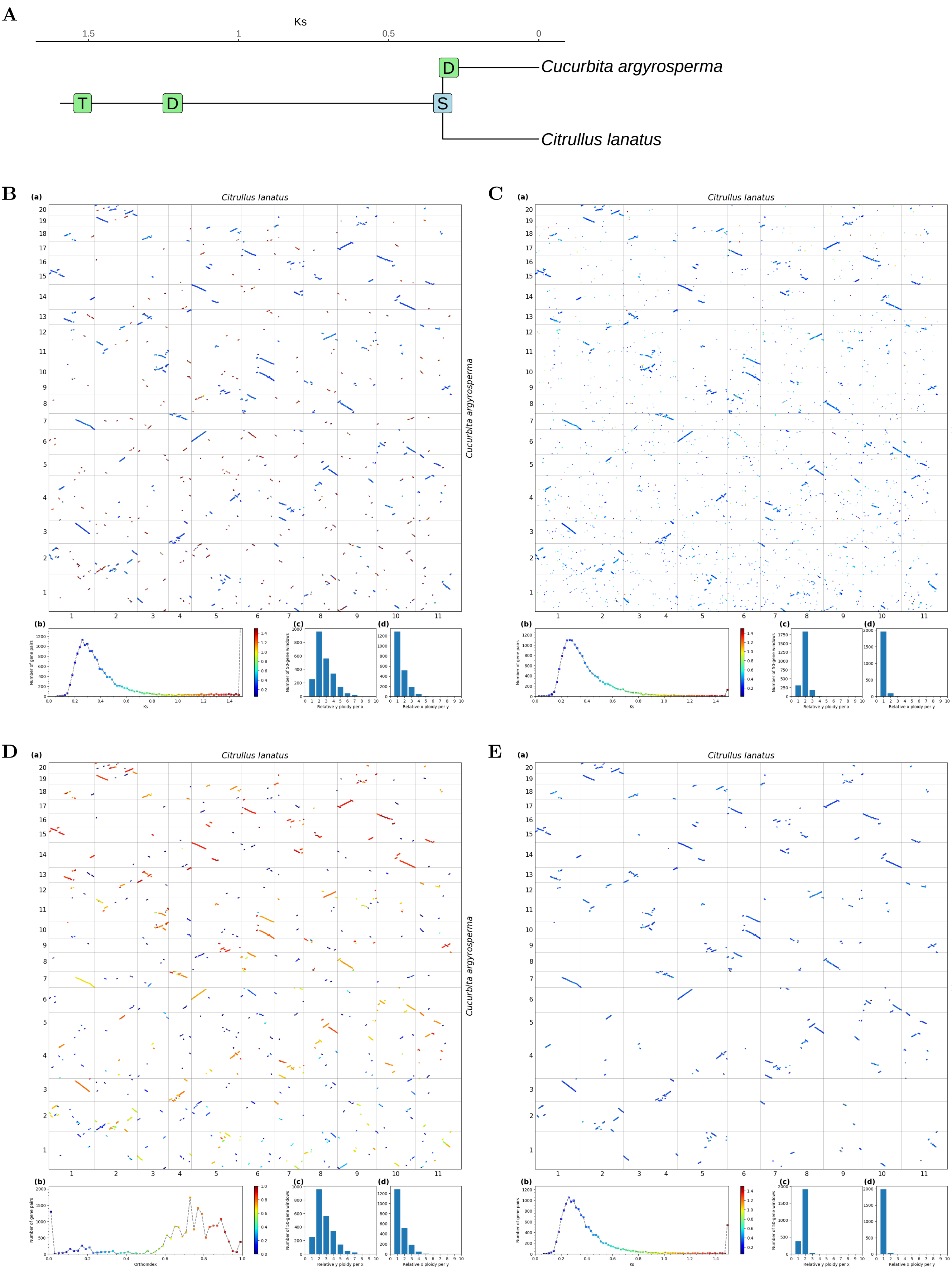

Figure S70. *Orthology Index* in the identification of orthologous synteny in *Citrullus lanatus* and *Cucurbita argyrosperma*. Refer to **Fig.1** for detailed descriptions.

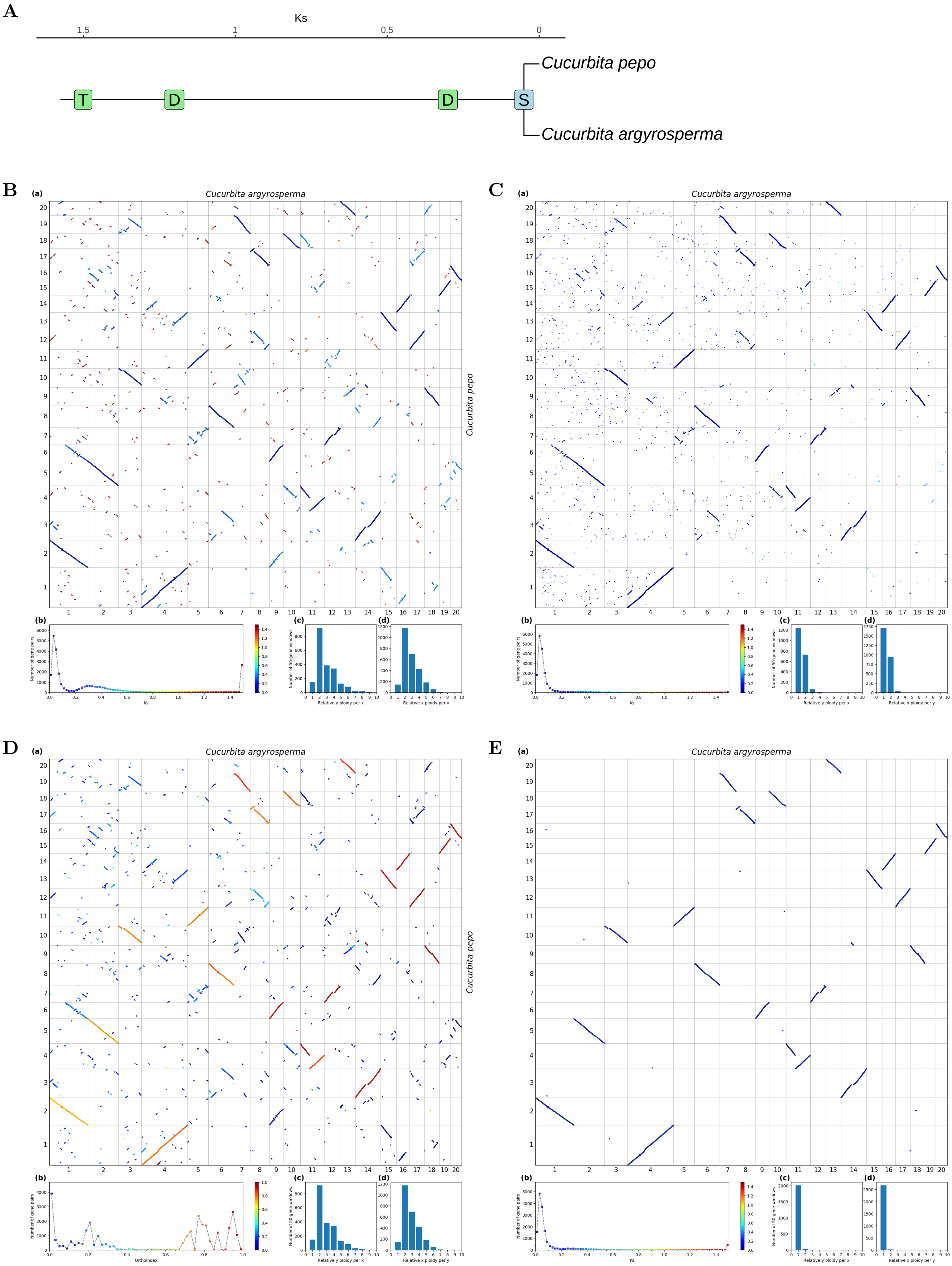

Figure S71. *Orthology Index* in the identification of orthologous synteny in *Cucurbita argyrosperma* and *Cucurbita pepo*. Refer to **Fig.1** for detailed descriptions.

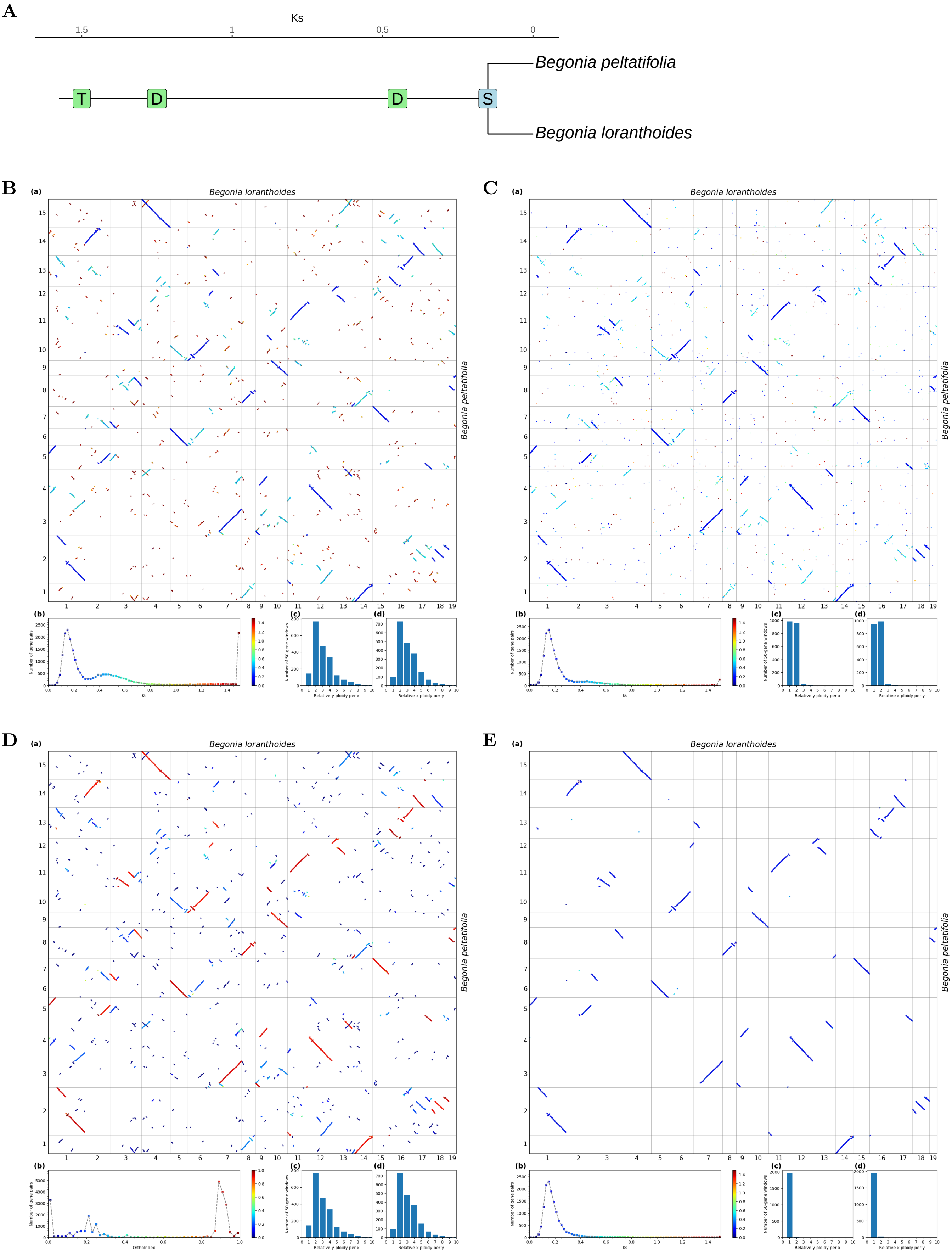

Figure S72. *Orthology Index* in the identification of orthologous synteny in *Begonia loranthoides* and *Begonia peltatifolia*. Refer to **Fig.1** for detailed descriptions.

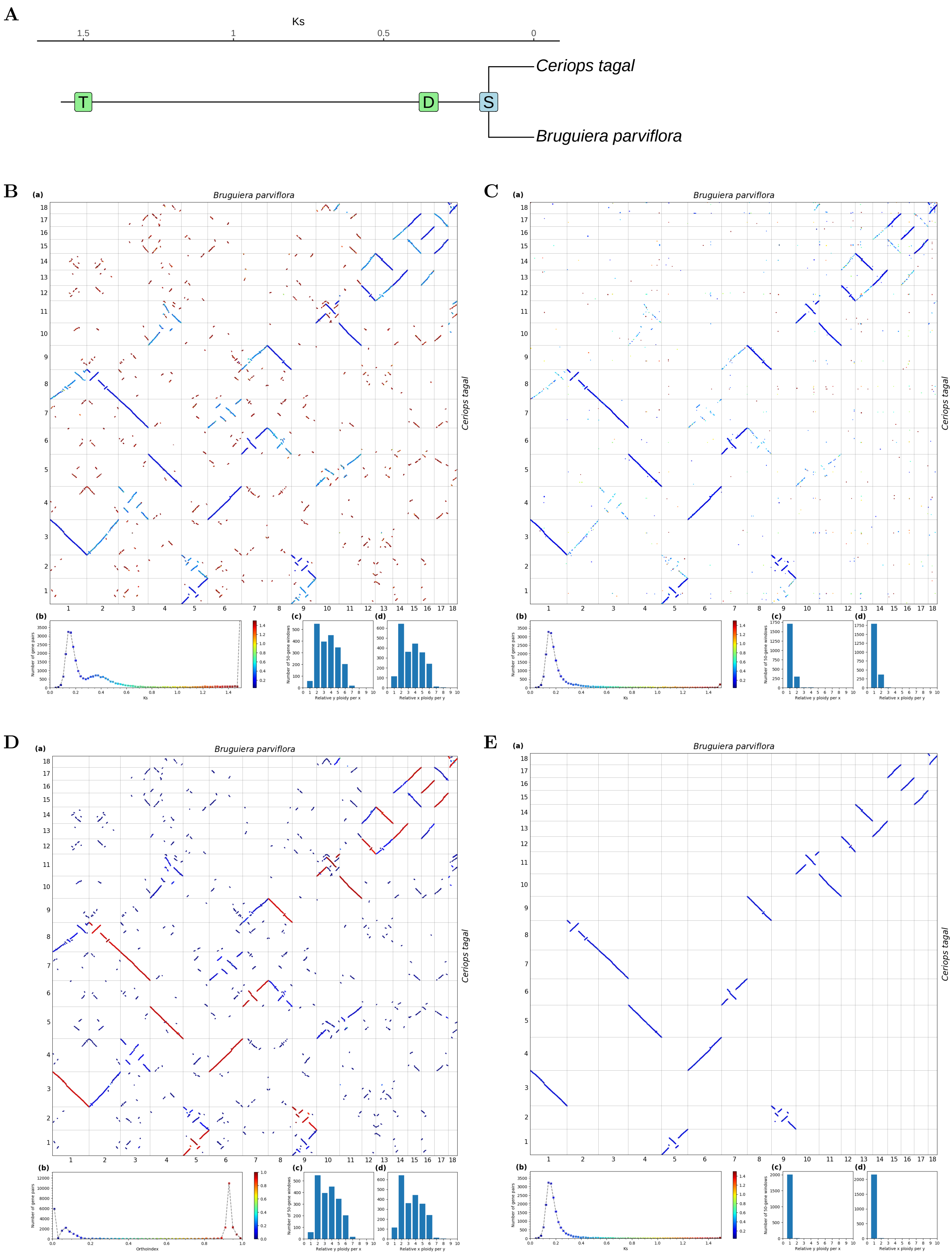

Figure S73. *Orthology Index* in the identification of orthologous synteny in *Bruguiera parviflora* and *Ceriops tagal*. Refer to **Fig.1** for detailed descriptions.

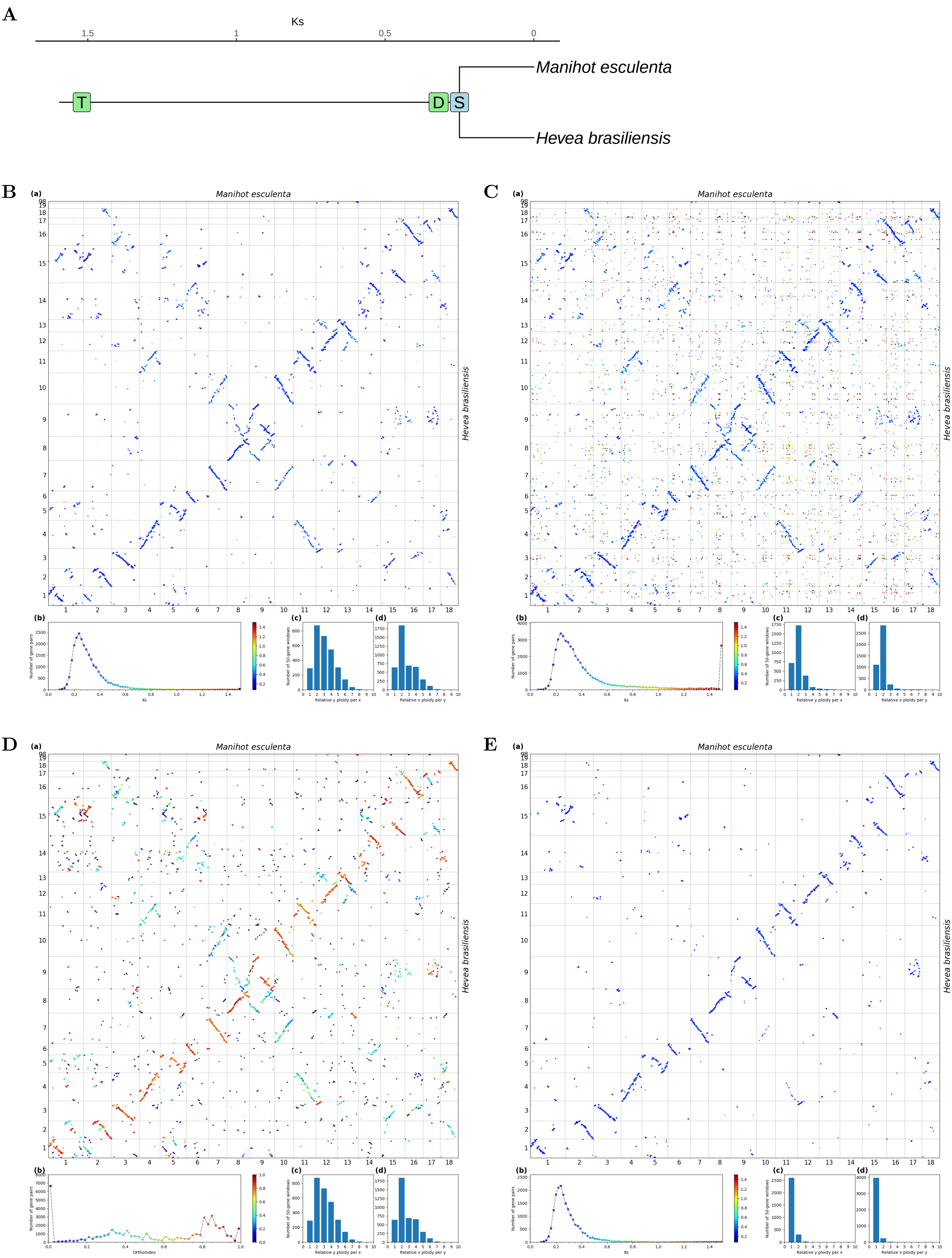

Figure S74. *Orthology Index* in the identification of orthologous synteny in *Hevea brasiliensis* and *Manihot esculenta.* Refer to **Fig.1** for detailed descriptions.

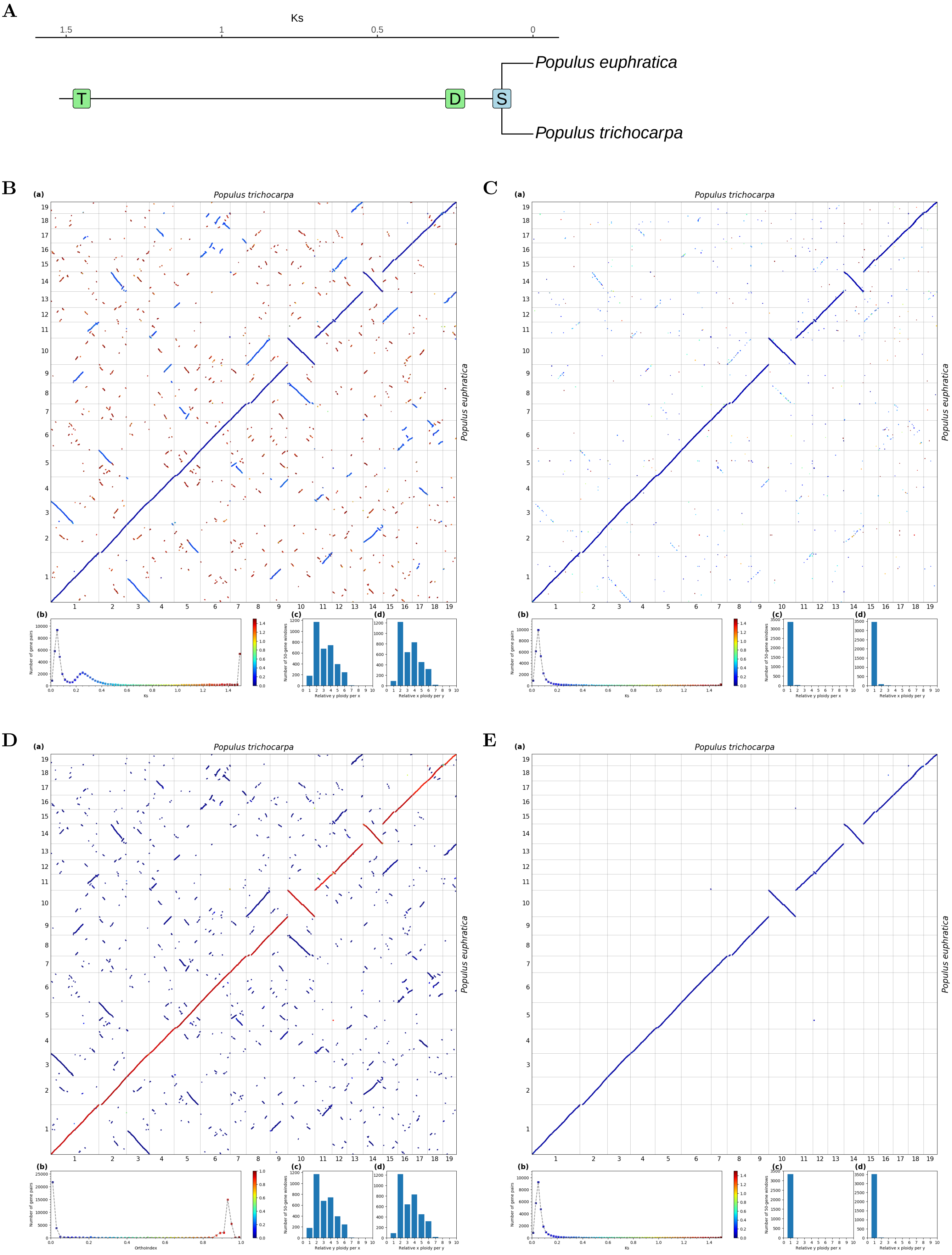

Figure S75. *Orthology Index* in the identification of orthologous synteny in *Populus trichocarpa* and *Populus euphratica*. Refer to **Fig.1** for detailed descriptions.

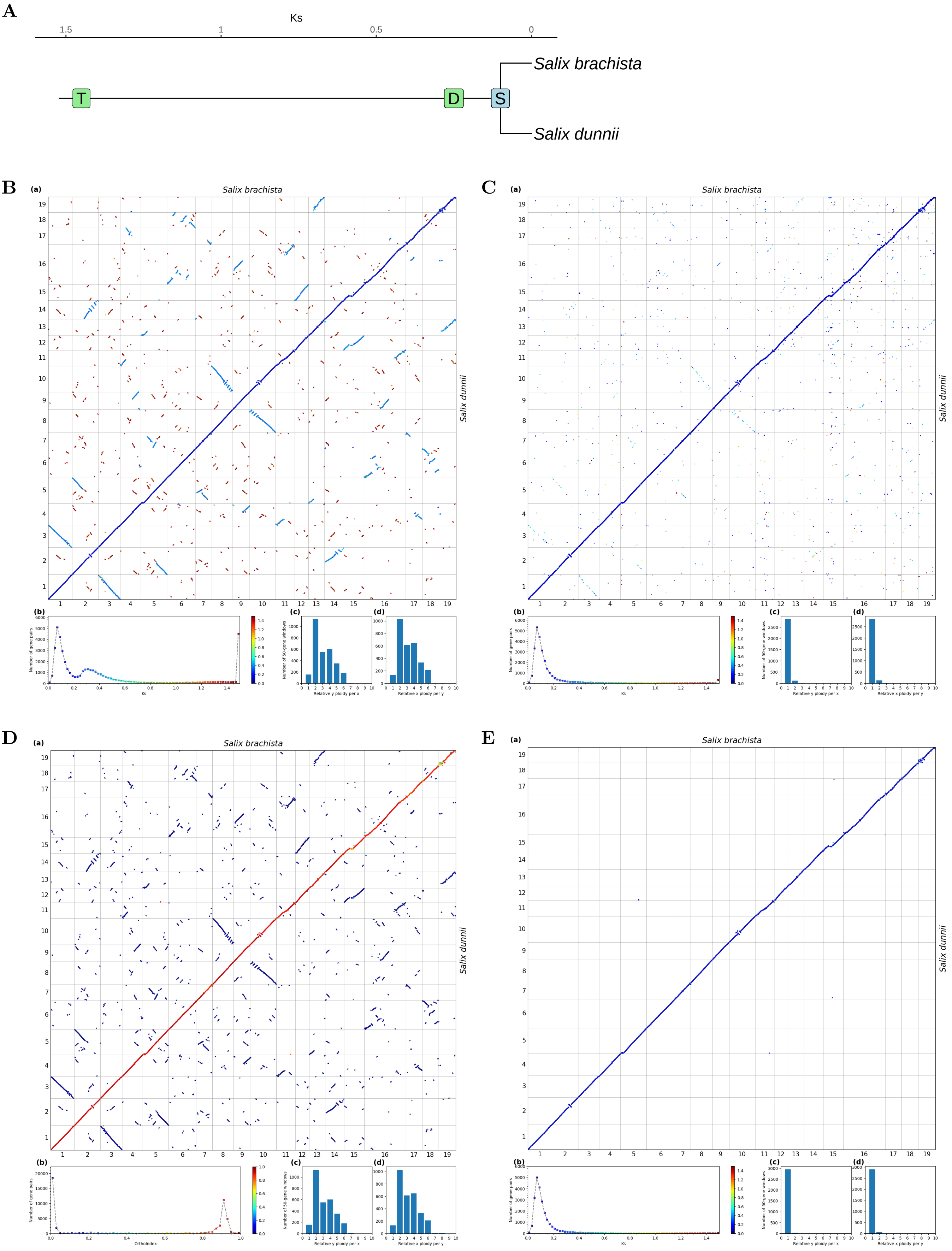

Figure S76. *Orthology Index* in the identification of orthologous synteny in *Salix dunnii* and *Salix brachista*. Refer to **Fig.1** for detailed descriptions.

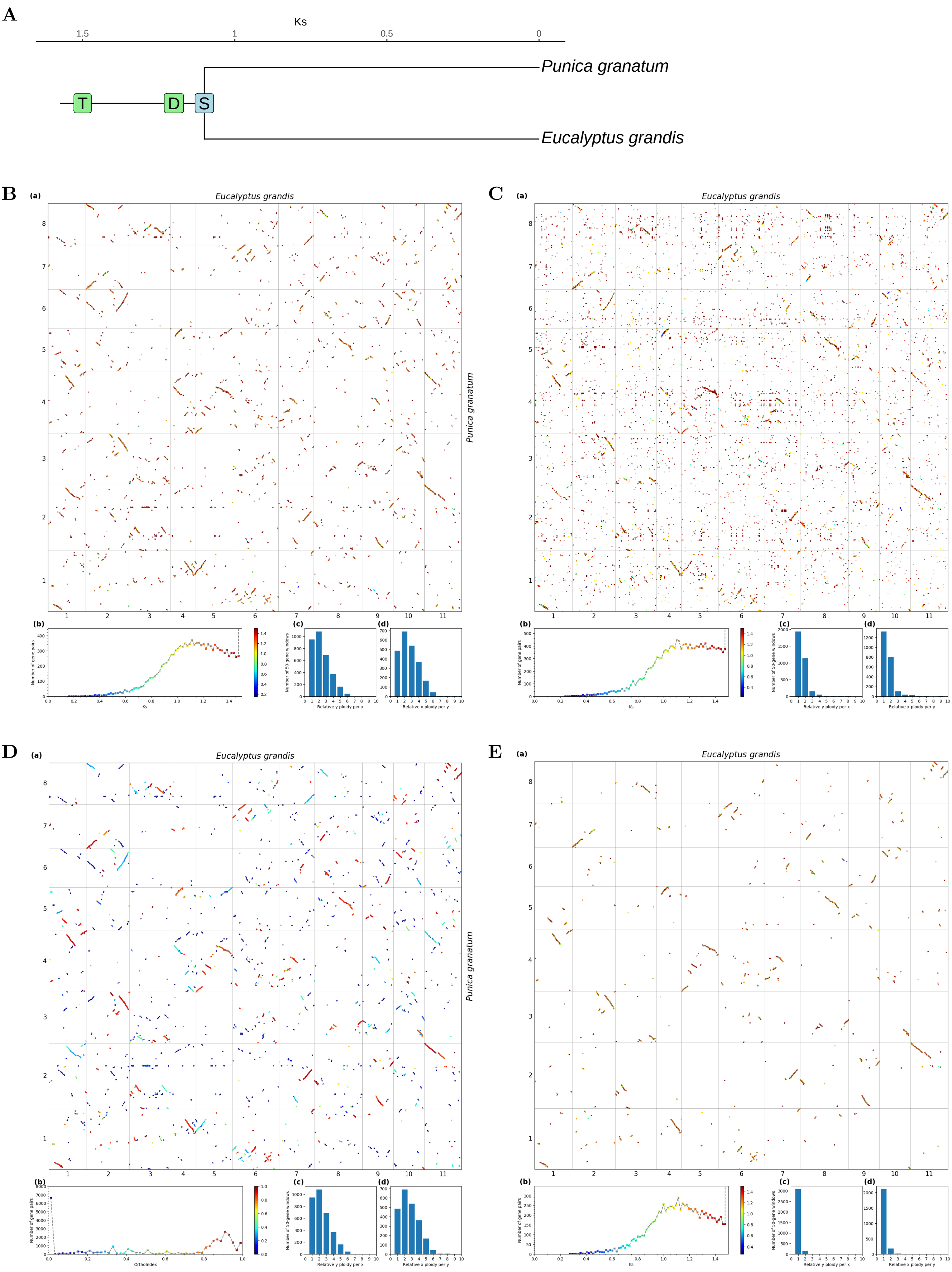

Figure S77. *Orthology Index* in the identification of orthologous synteny in *Eucalyptus grandis* and *Punica granatum*. Refer to **Fig.1** for detailed descriptions.

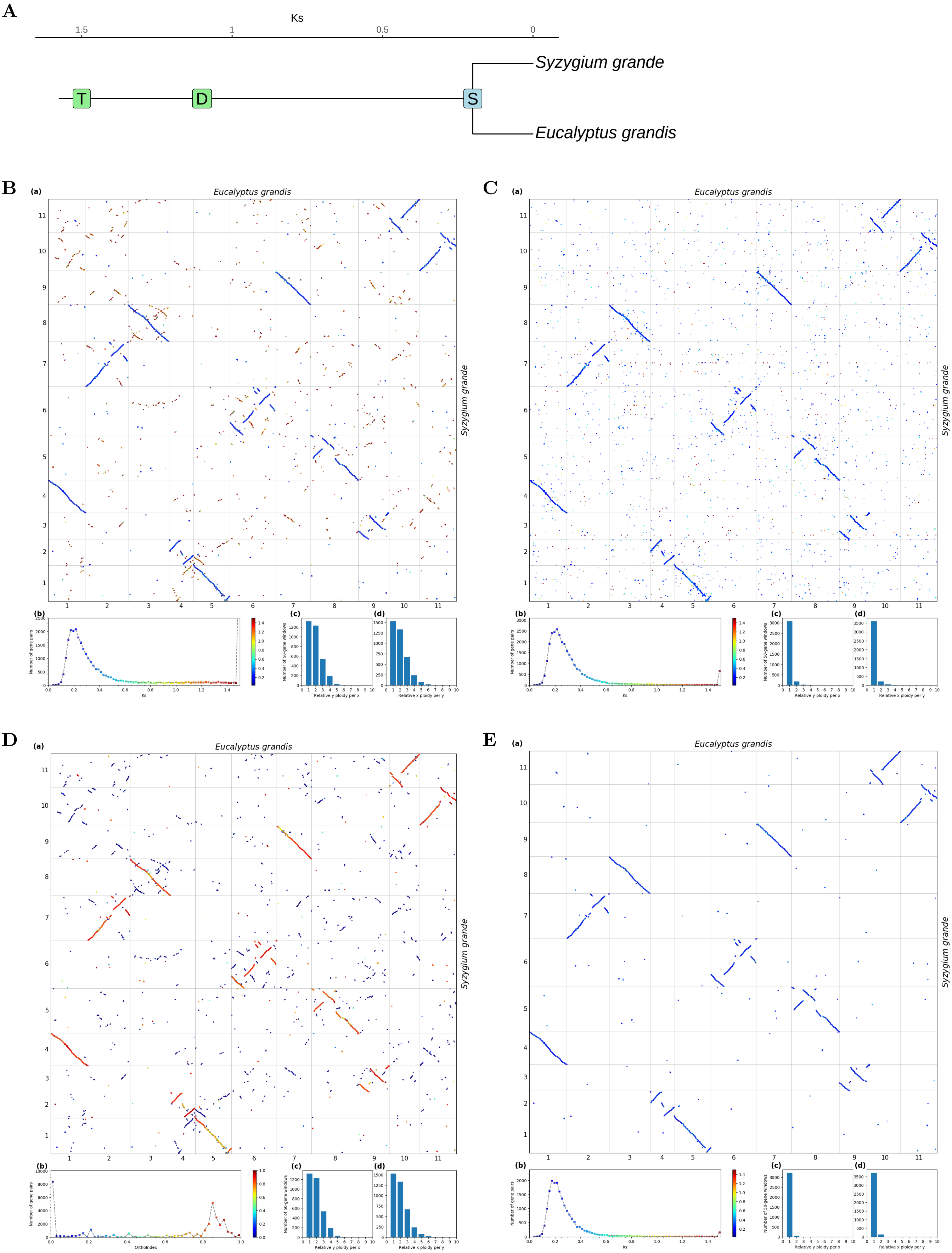

Figure S78. *Orthology Index* in the identification of orthologous synteny in *Eucalyptus grandis* and *Syzygium grande*. Refer to **Fig.1** for detailed descriptions.

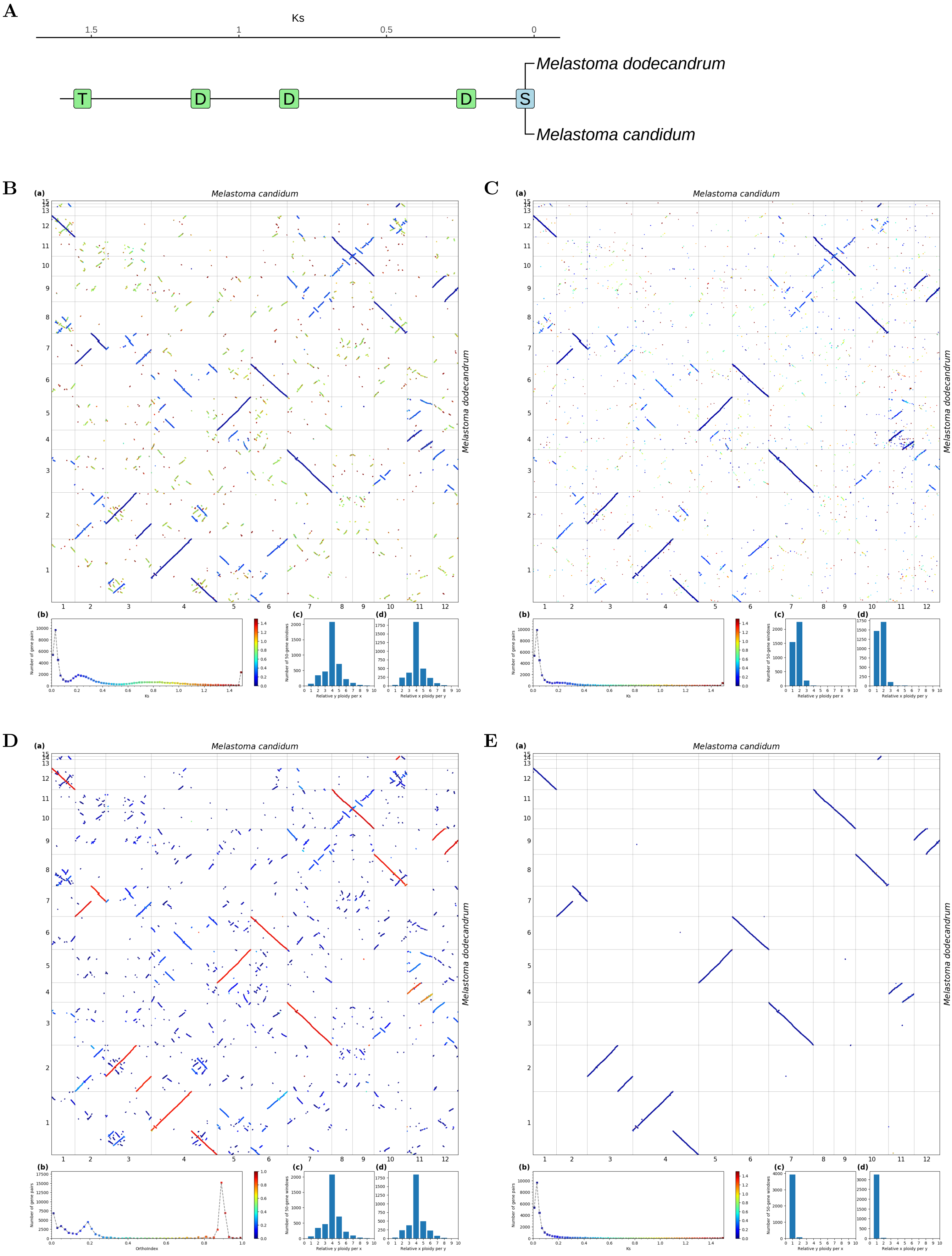

Figure S79. *Orthology Index* in the identification of orthologous synteny in *Melastoma candidum* and *Melastoma dodecandrum*. Refer to **Fig.1** for detailed descriptions.

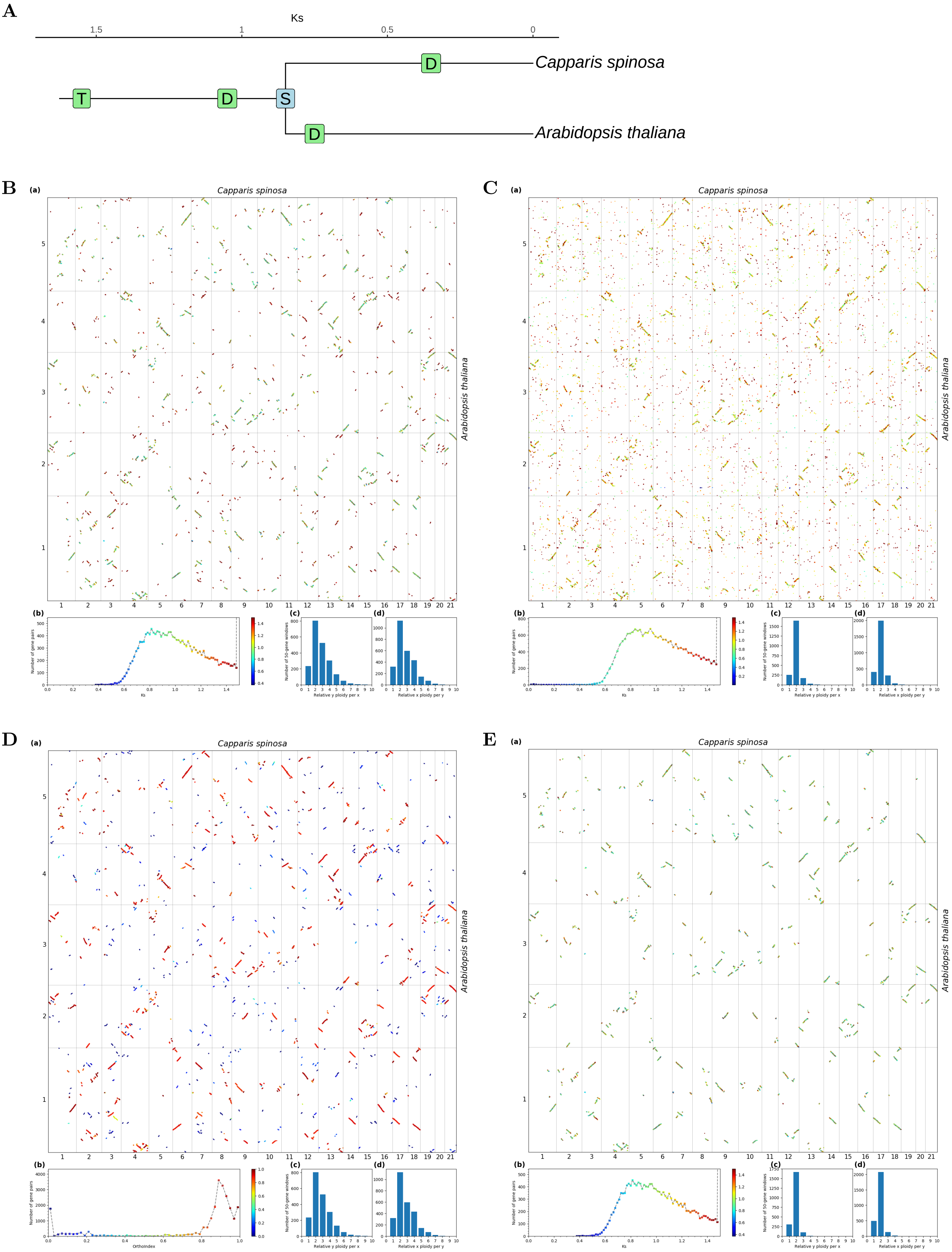

Figure S80. *Orthology Index* in the identification of orthologous synteny in *Arabidopsis thaliana* and *Capparis spinosa*. Refer to **Fig.1** for detailed descriptions.

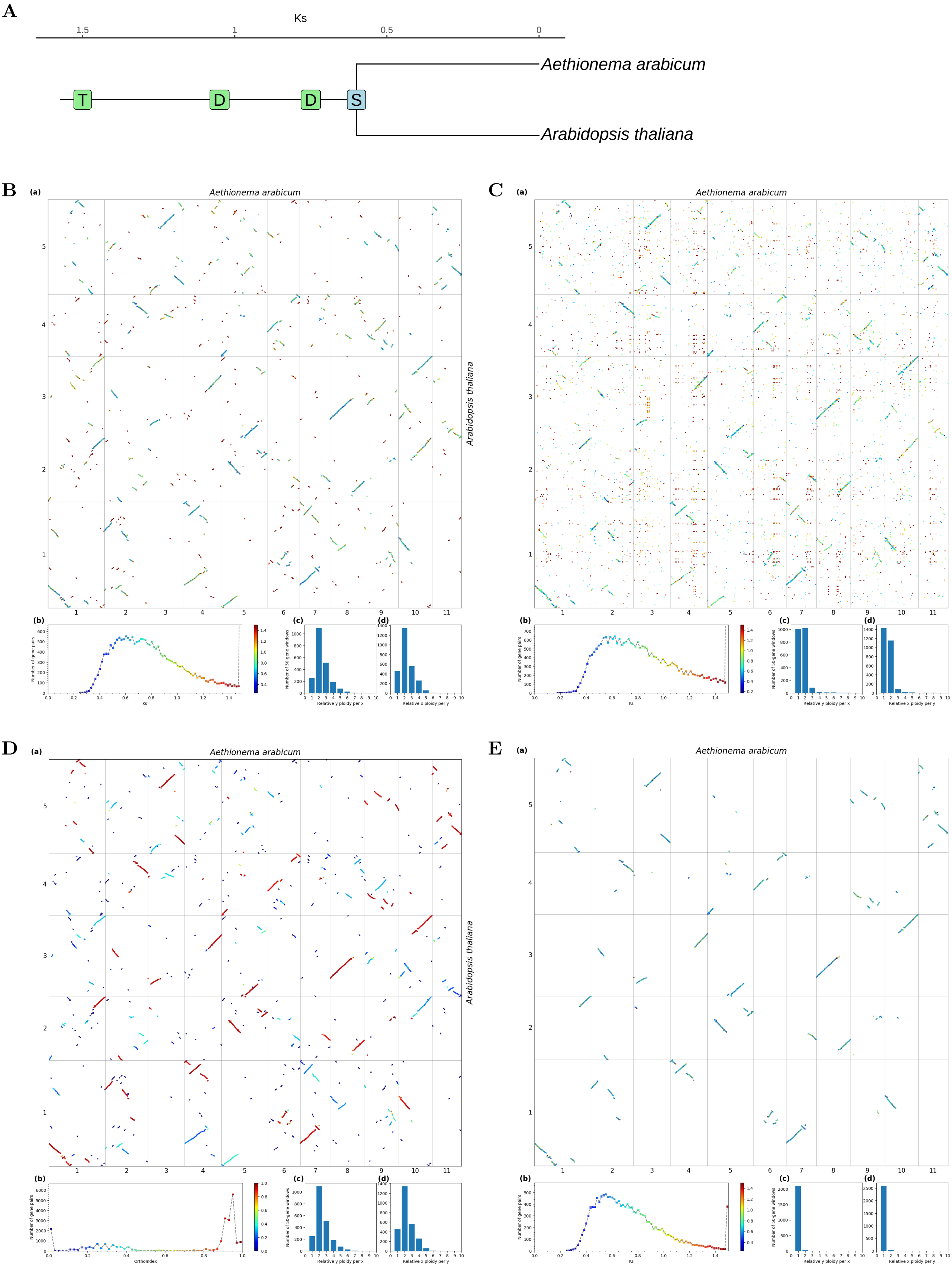

Figure S81. *Orthology Index* in the identification of orthologous synteny in *Arabidopsis thaliana* and *Aethionema arabicum*. Refer to **Fig.1** for detailed descriptions.

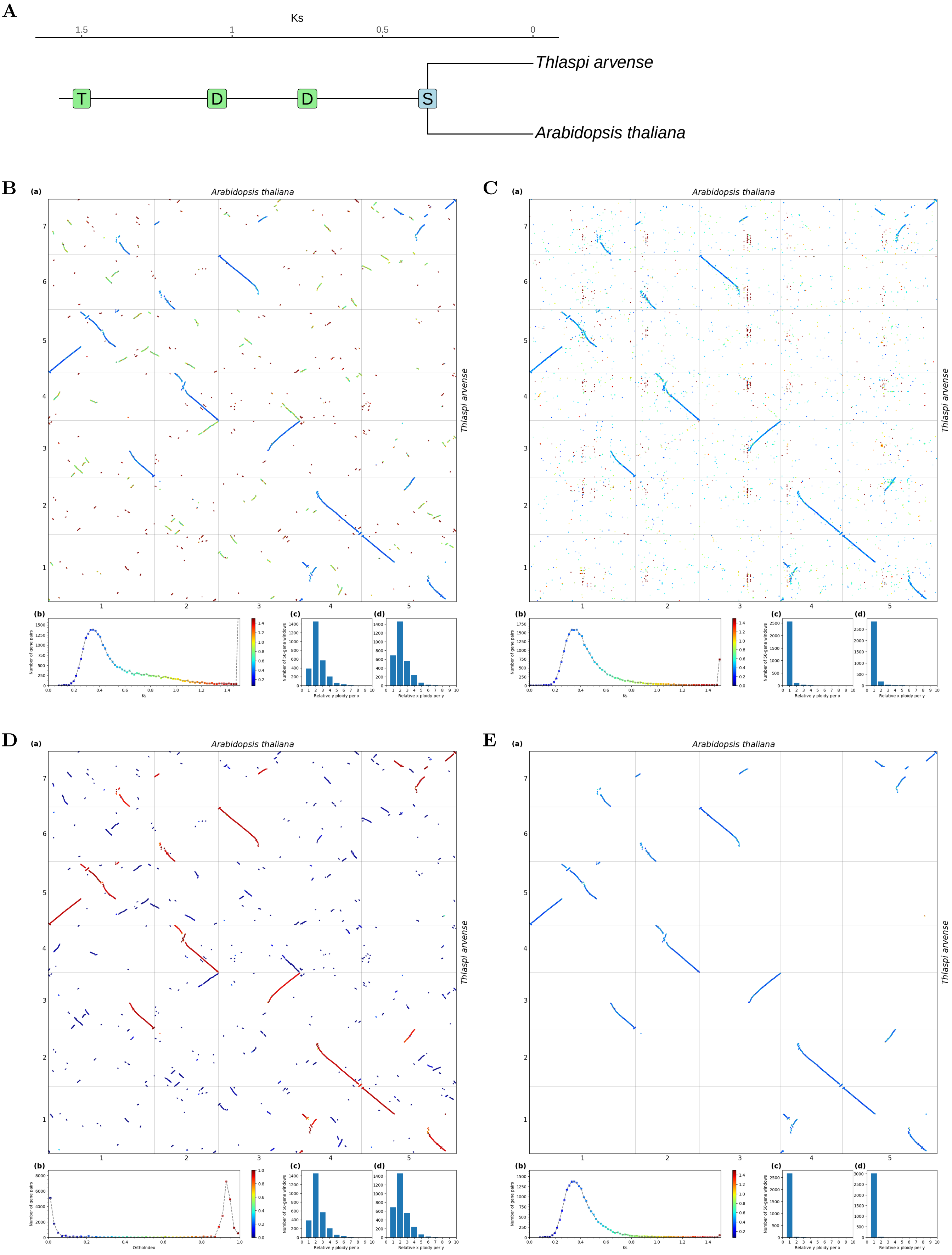

Figure S82. *Orthology Index* in the identification of orthologous synteny in *Arabidopsis thaliana* and *Thlaspi arvense*. Refer to **Fig.1** for detailed descriptions.

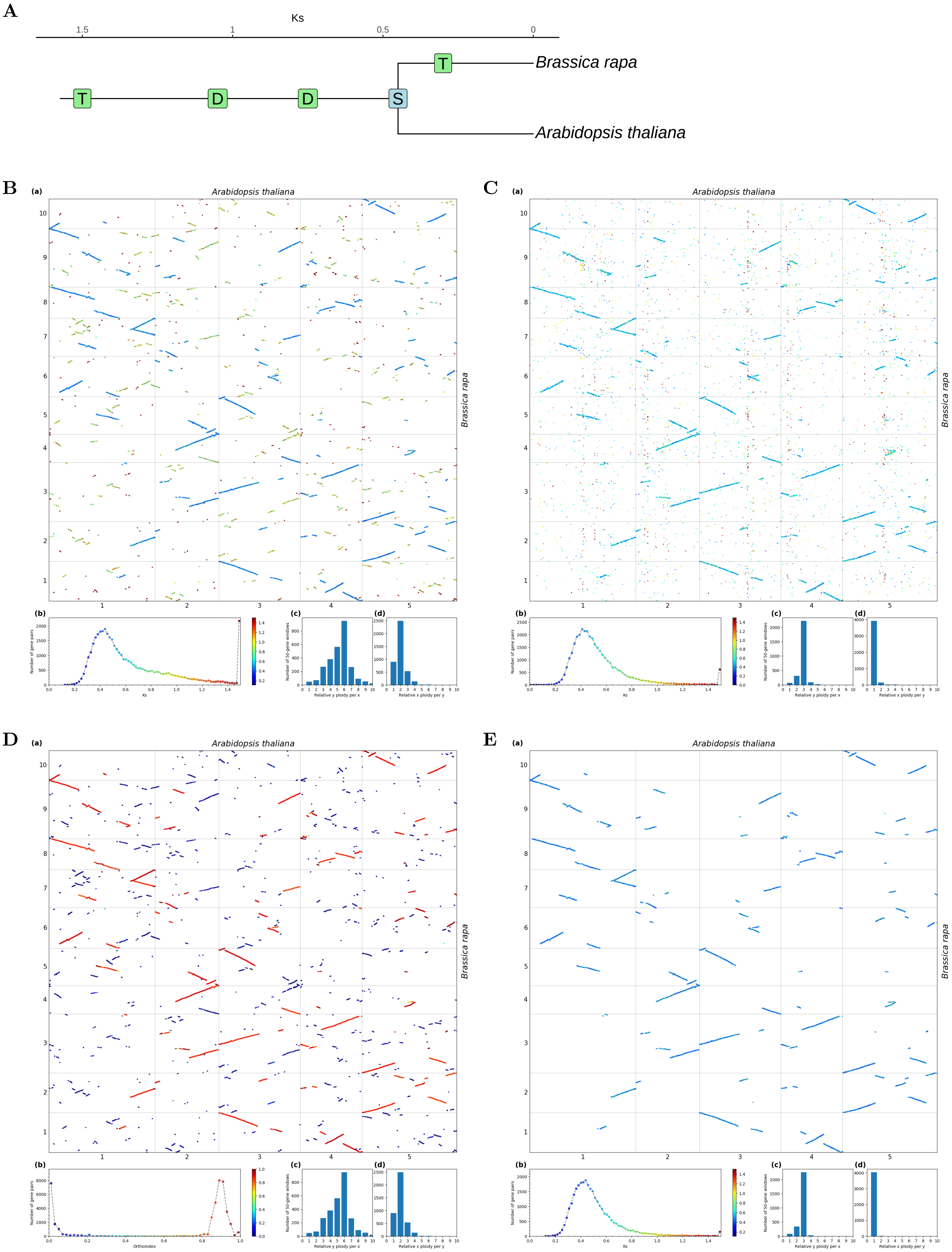

Figure S83. *Orthology Index* in the identification of orthologous synteny in *Arabidopsis thaliana* and *Brassica rapa*. Refer to **Fig.1** for detailed descriptions.

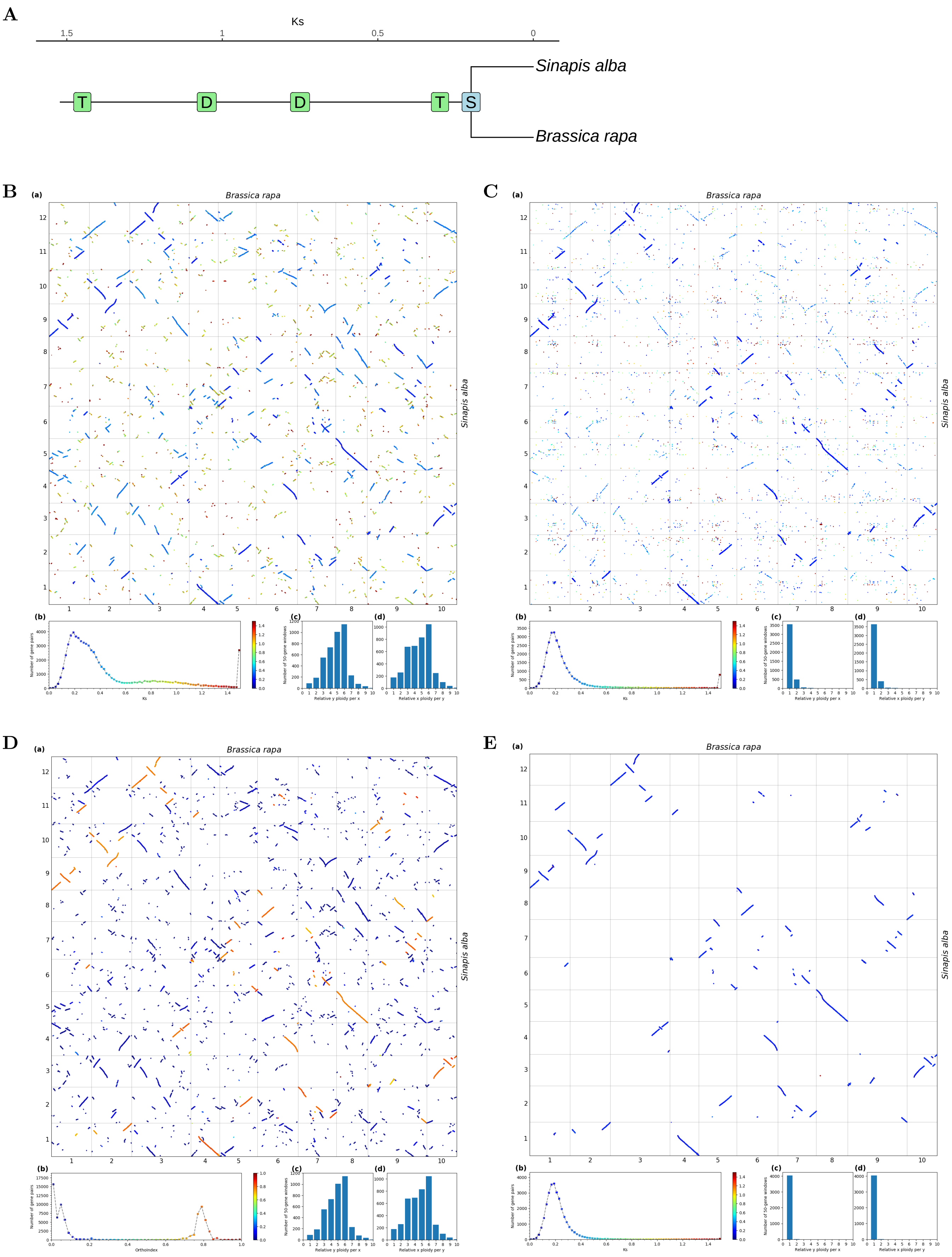

Figure S84. *Orthology Index* in the identification of orthologous synteny in *Brassica rapa* and *Sinapis alba*. Refer to **Fig.1** for detailed descriptions.

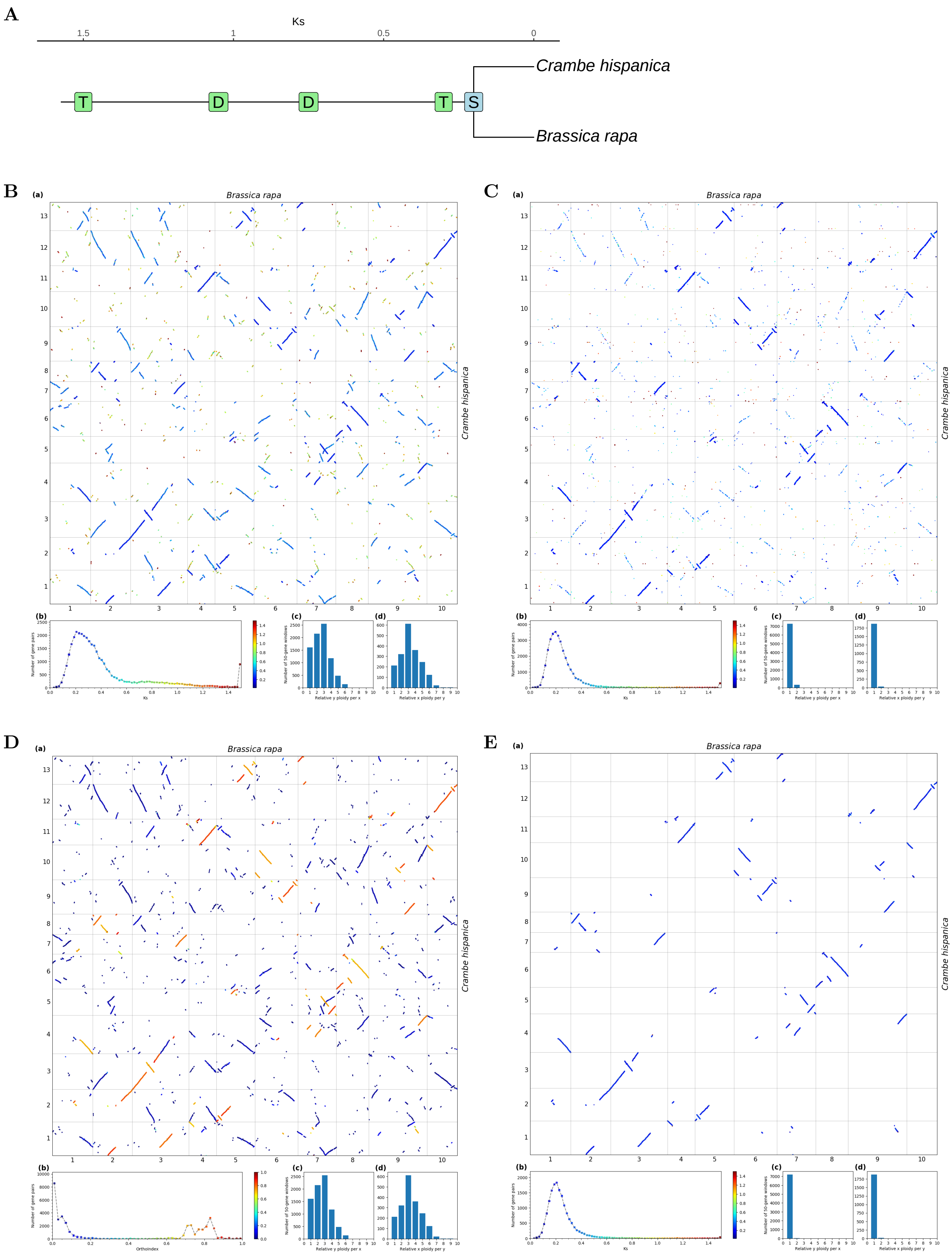

Figure S85. *Orthology Index* in the identification of orthologous synteny in *Brassica rapa* and *Crambe hispanica*. Refer to **Fig.1** for detailed descriptions.

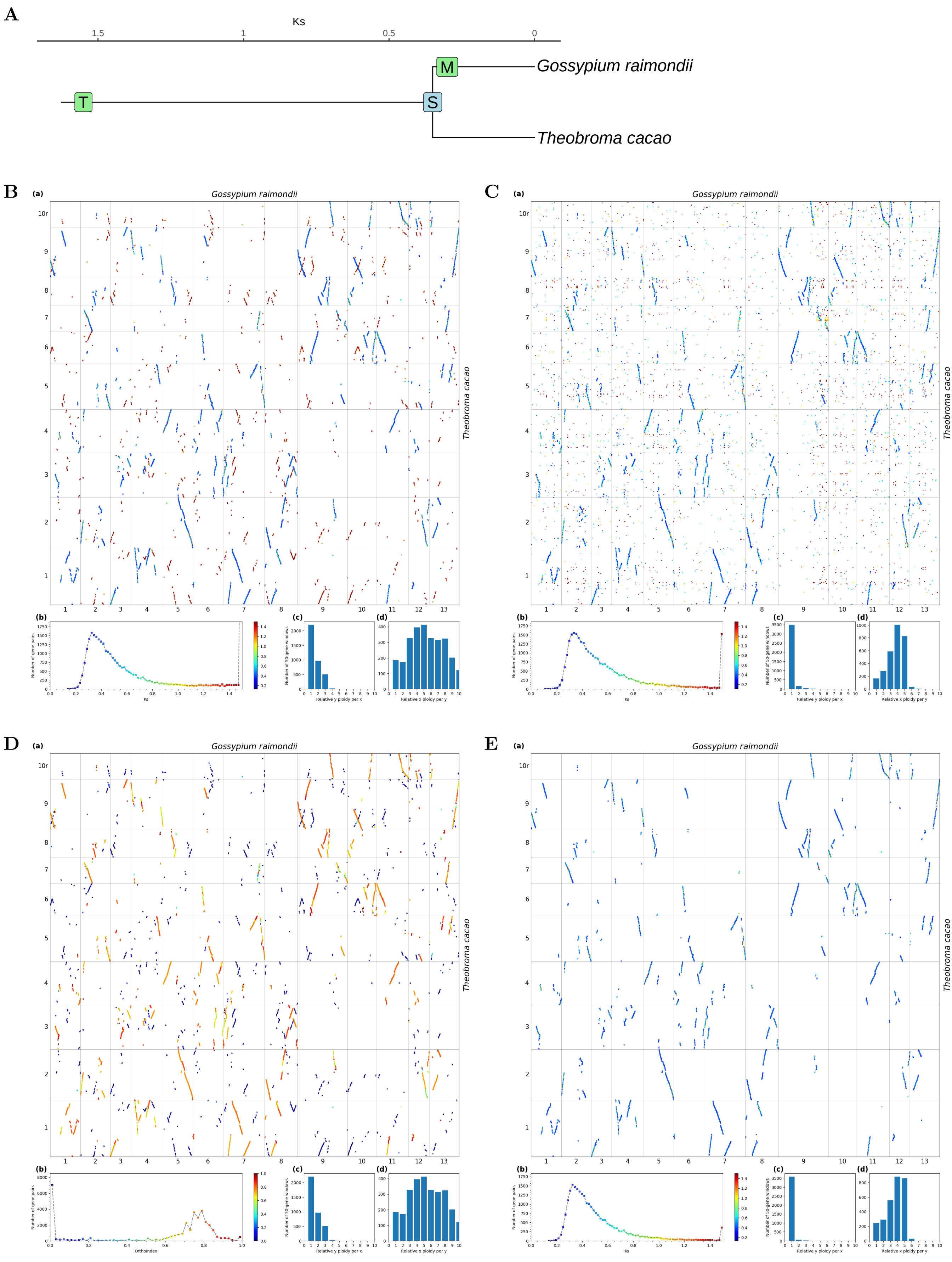

Figure S86. *Orthology Index* in the identification of orthologous synteny in *Theobroma cacao* and *Gossypium raimondii*. Refer to **Fig.1** for detailed descriptions.

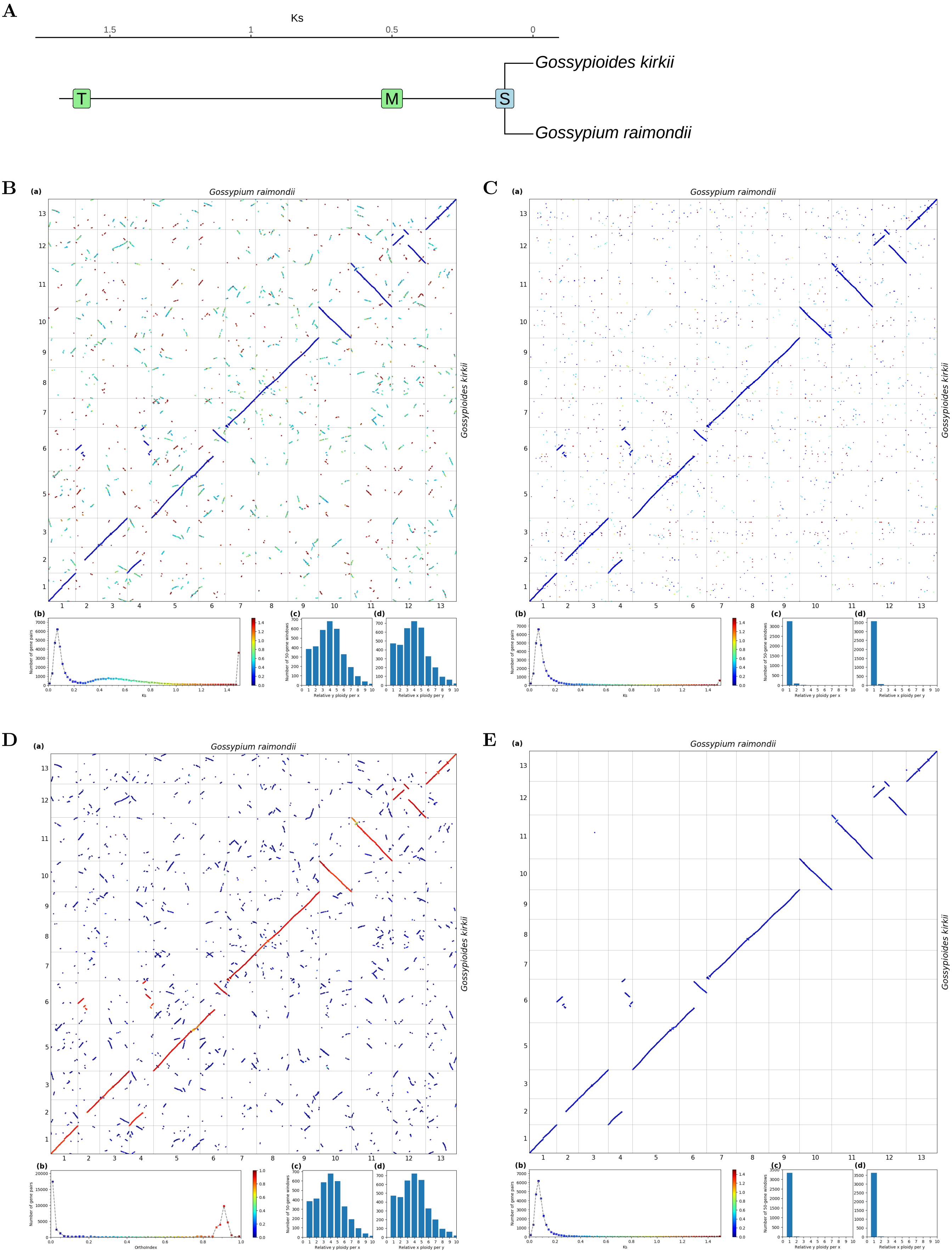

Figure S87. *Orthology Index* in the identification of orthologous synteny in *Gossypium raimondii* and *Gossypioides kirkii*. Refer to **Fig.1** for detailed descriptions.

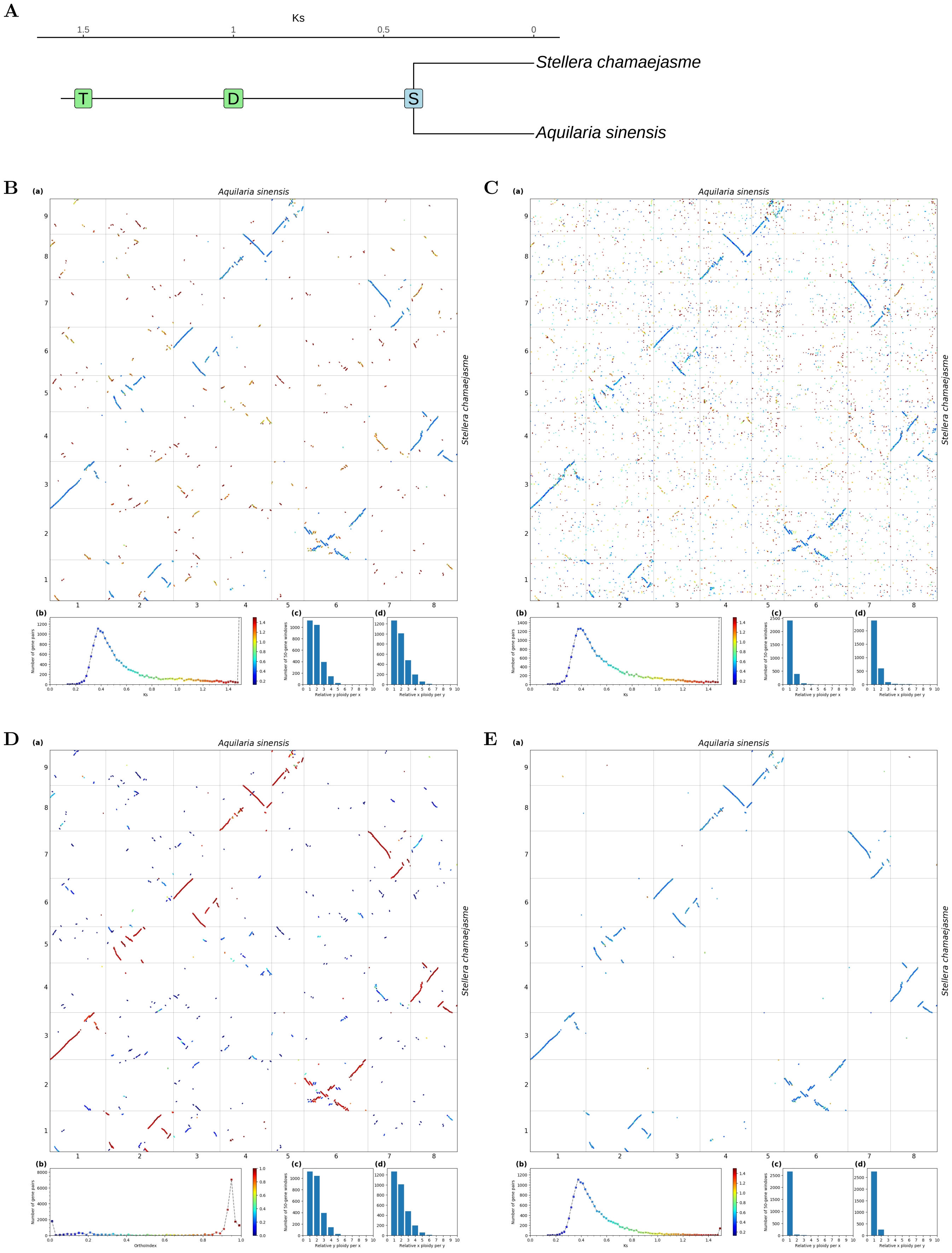

Figure S88. *Orthology Index* in the identification of orthologous synteny in *Aquilaria sinensis* and *Stellera chamaejasme*. Refer to **Fig.1** for detailed descriptions.

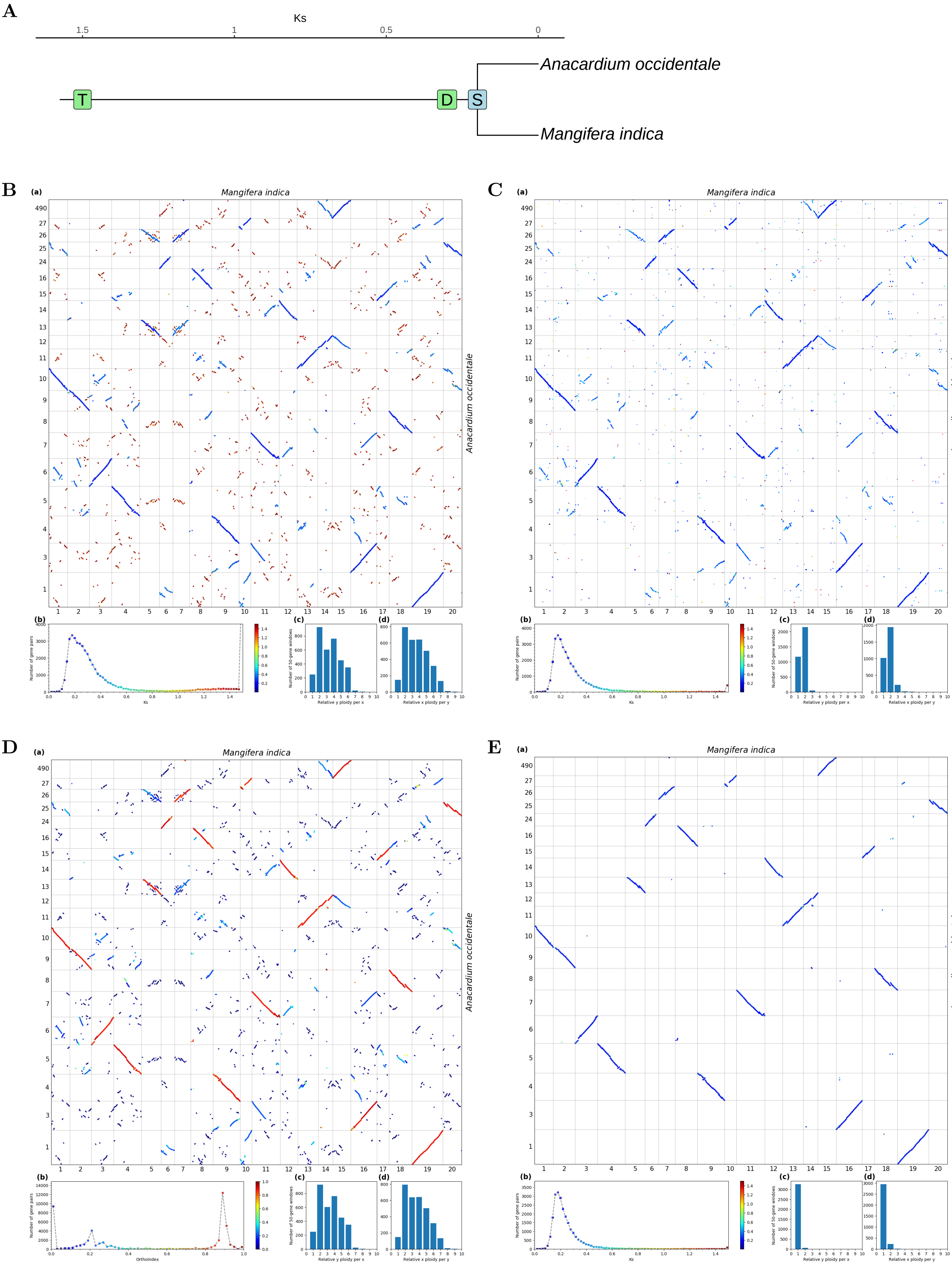

Figure S89. *Orthology Index* in the identification of orthologous synteny in *Mangifera indica* and *Anacardium occidentale*. Refer to **Fig.1** for detailed descriptions.

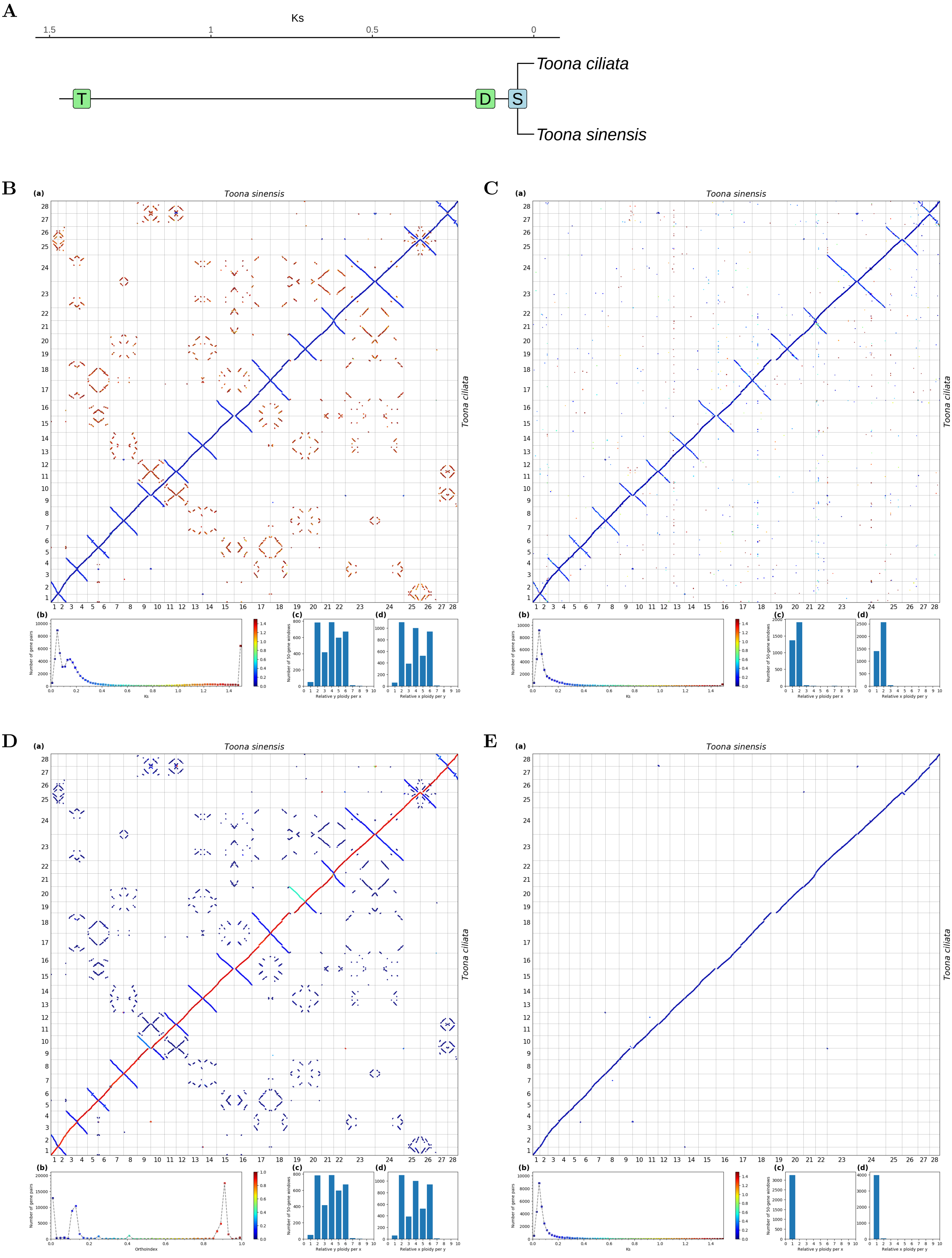

Figure S90. *Orthology Index* in the identification of orthologous synteny in *Toona sinensis* and *Toona ciliata*. Refer to **Fig.1** for detailed descriptions.

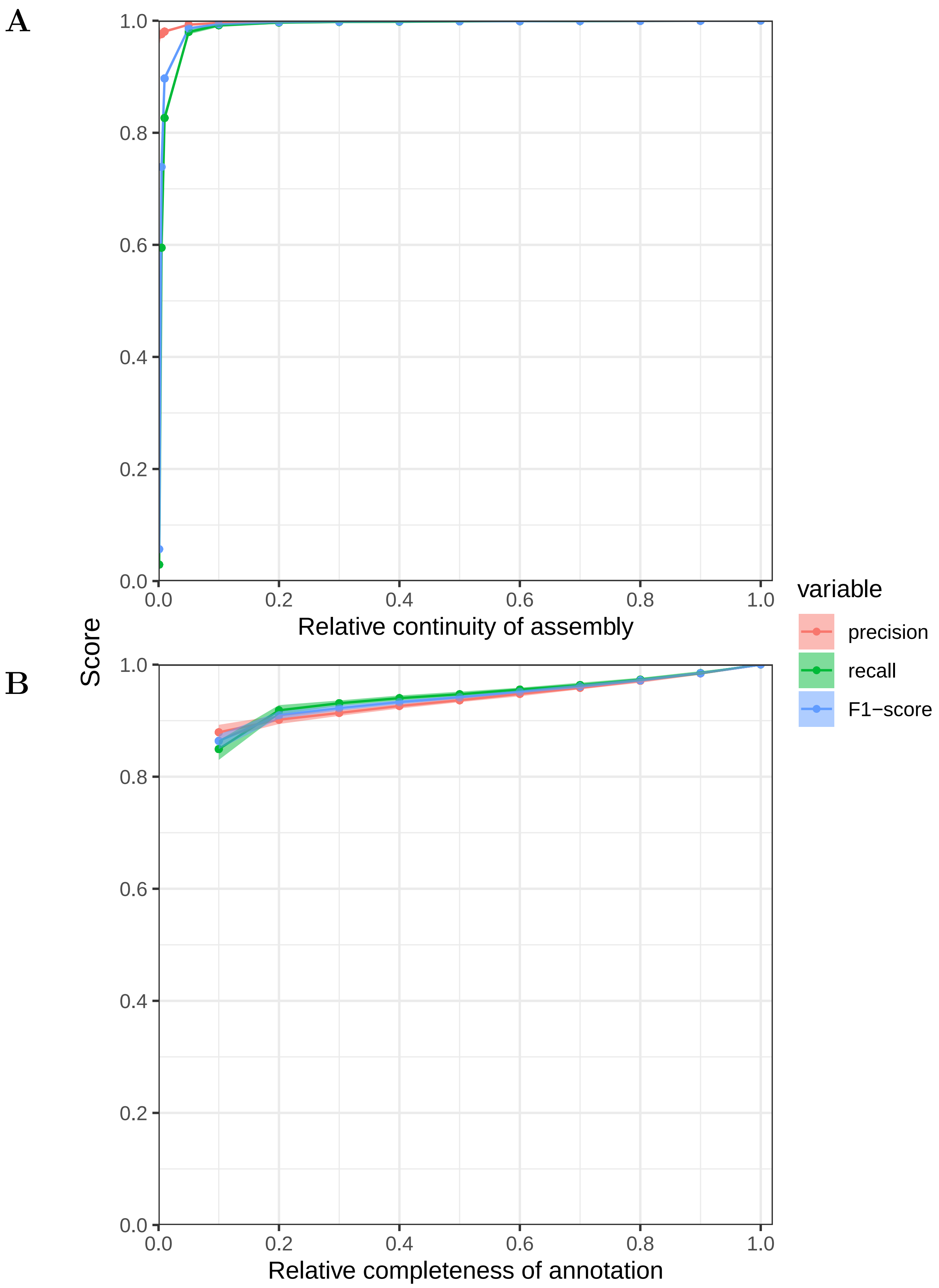

Figure S91. Assessments of the *OI* method agaist genome assembly continuity (A) and annotation completeness (B). The line and point represent the median values, while the shaded area indicates the 95% confidence interval (CI) from 50 repeated simulations.

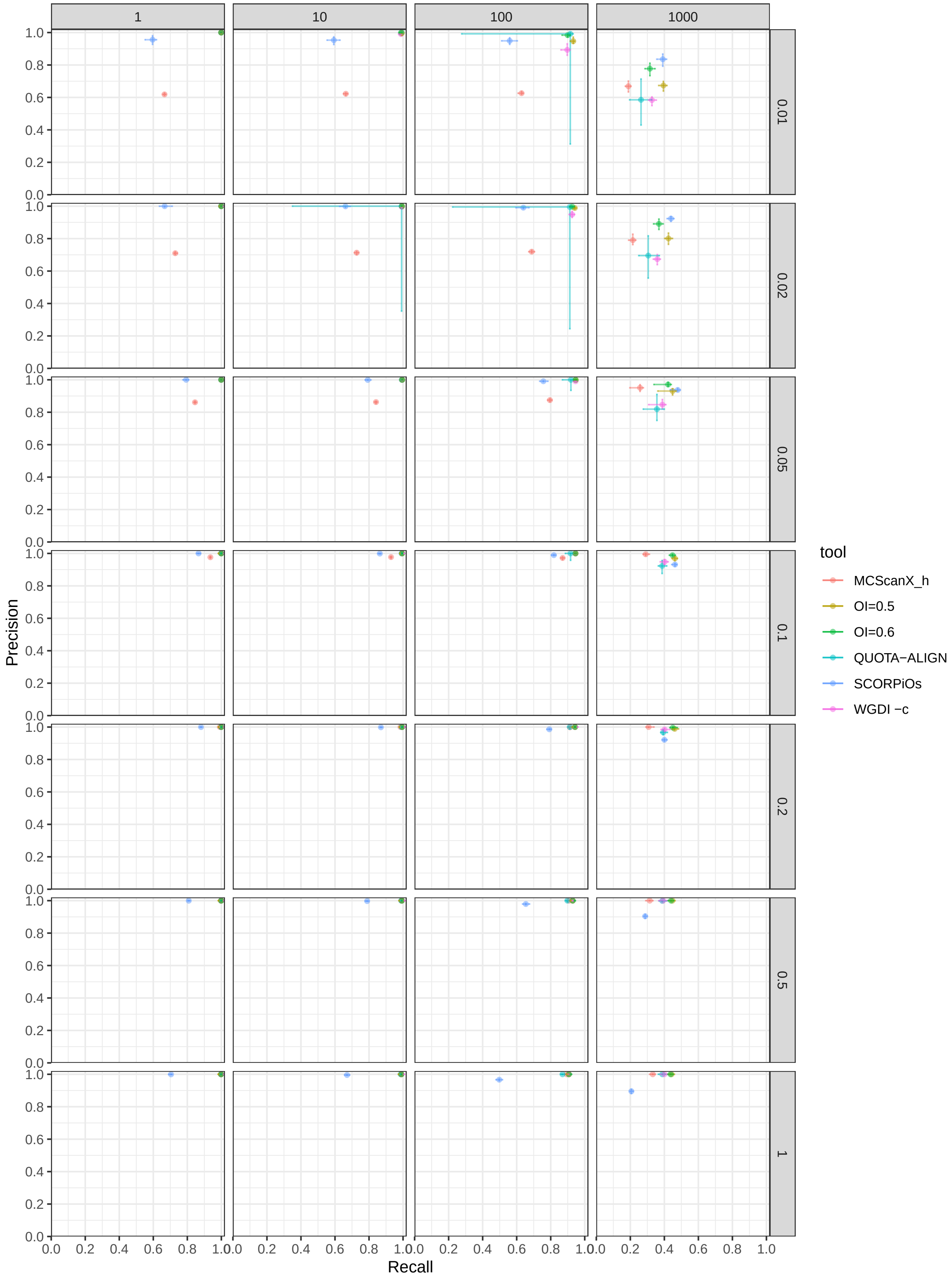

Figure S92. Comparisons of existing tools and OI with cutoffs 0.5 and 0.6, based on the simulated benchmarks with varied levels of ΔT ($\Delta T \epsilon$ {0.01, 0.02, 0.05, 0.1, 0.2, 0.5, 1}) and chromosome evolution ($fold \epsilon$ {1, 10, 100, 1000}). The point represent the median values, and the error bar indicates the 95% CI from 50 repeated simulations.

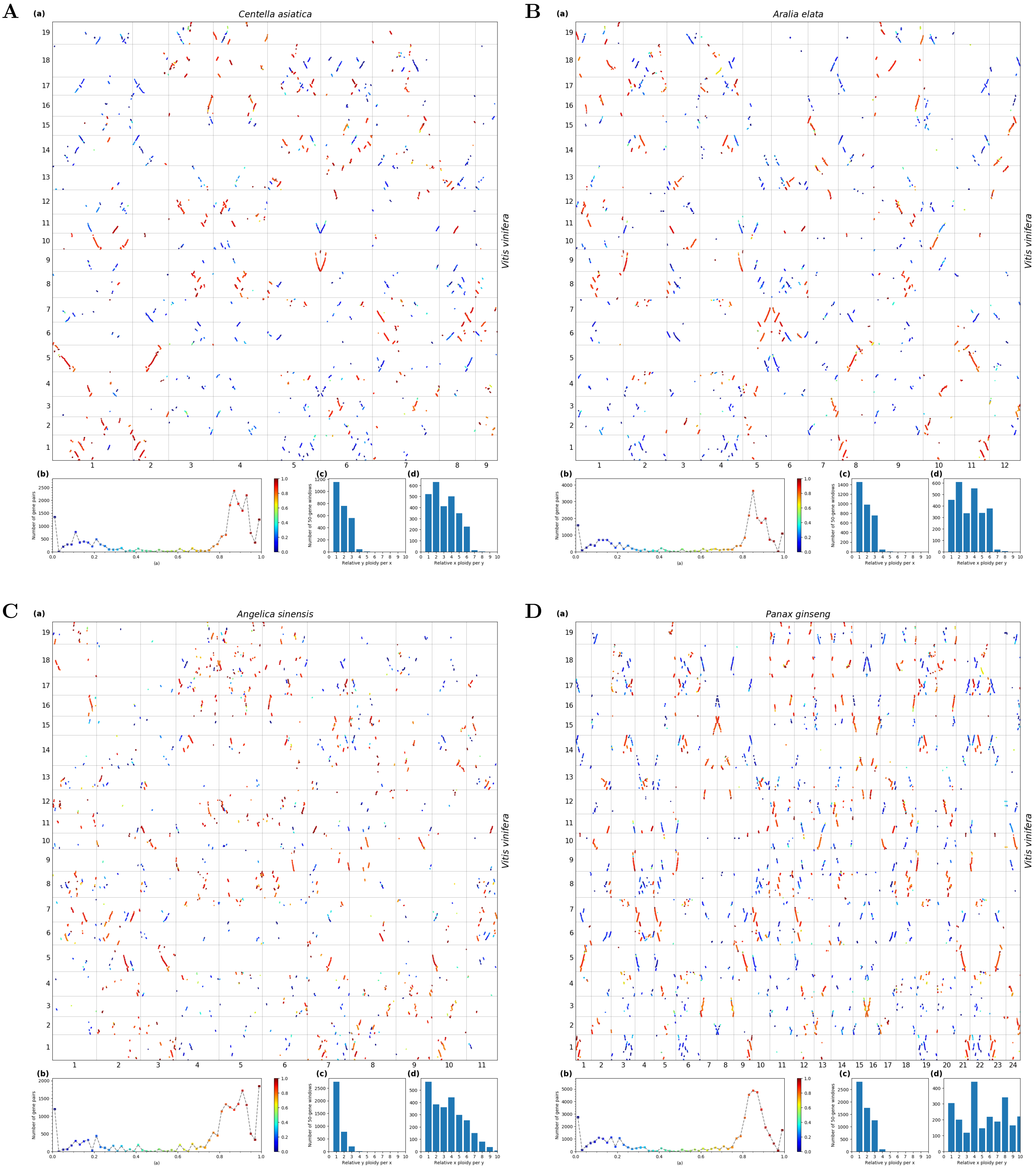

Figure S93. *Orthology Index*-colored dot plots showing orthologous syntenic relationships between *Centella asiatica* : *Vitis vinifera* (2:1 orthologous synteny depth ratio), *Aralia elata* : *Vitis vinifera* (2:1), *Angelica sinensis* : *Vitis vinifera* (4:1), and *Panax ginseng* : *Vitis vinifera* (4:1).

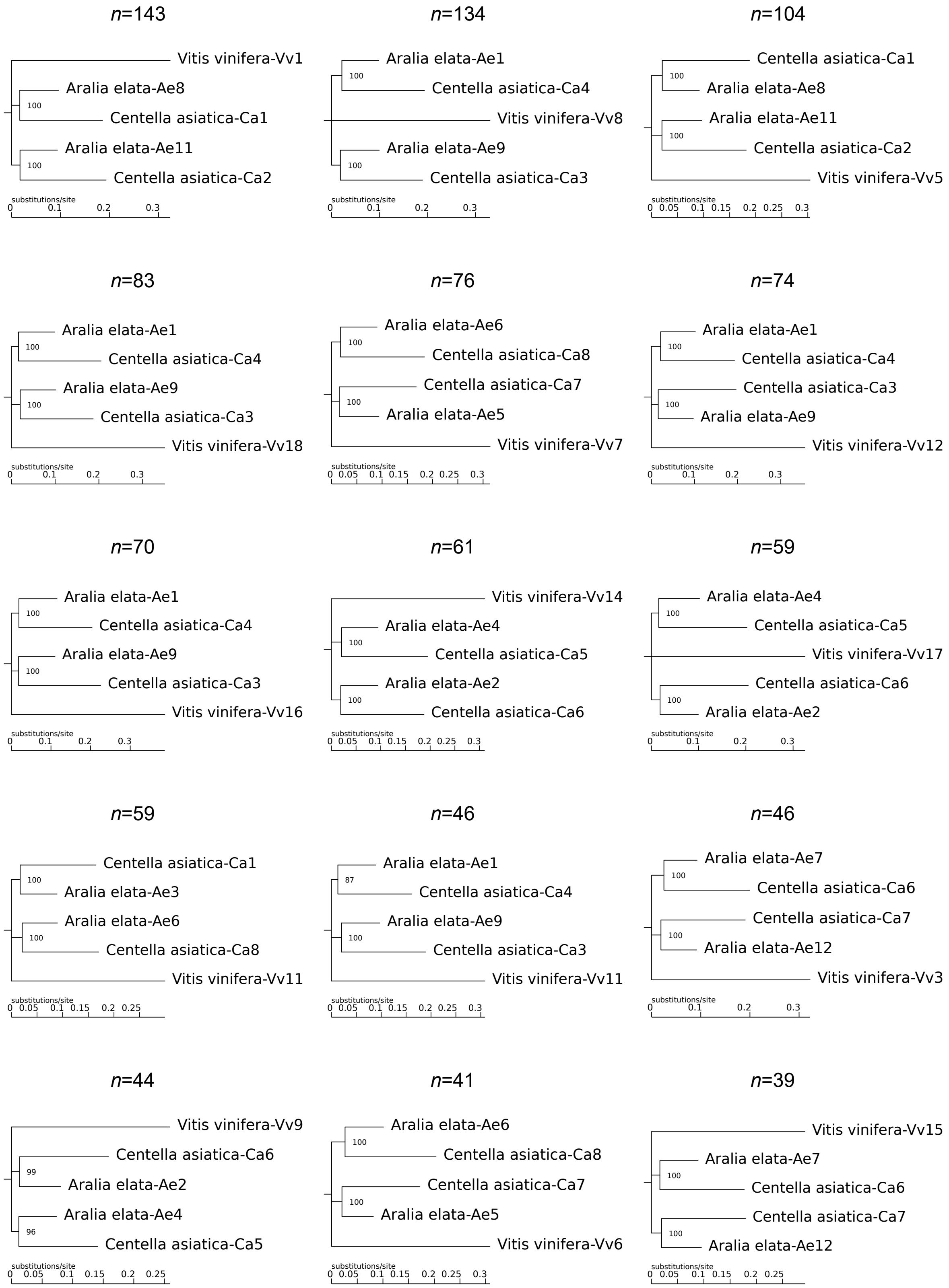

Figure S94. Macro-synteny phylogenies inferred based on concatenated 1:2:2 (*Vitis vinifera* : *Aralia elata* : *Centella asiatica*) orthologous syntenic genes. The leaf labels on the trees are chromosome numbers. n, number of orthologous syntenic genes involved. Numbers at nodes represent the bootstrap values (percentage).

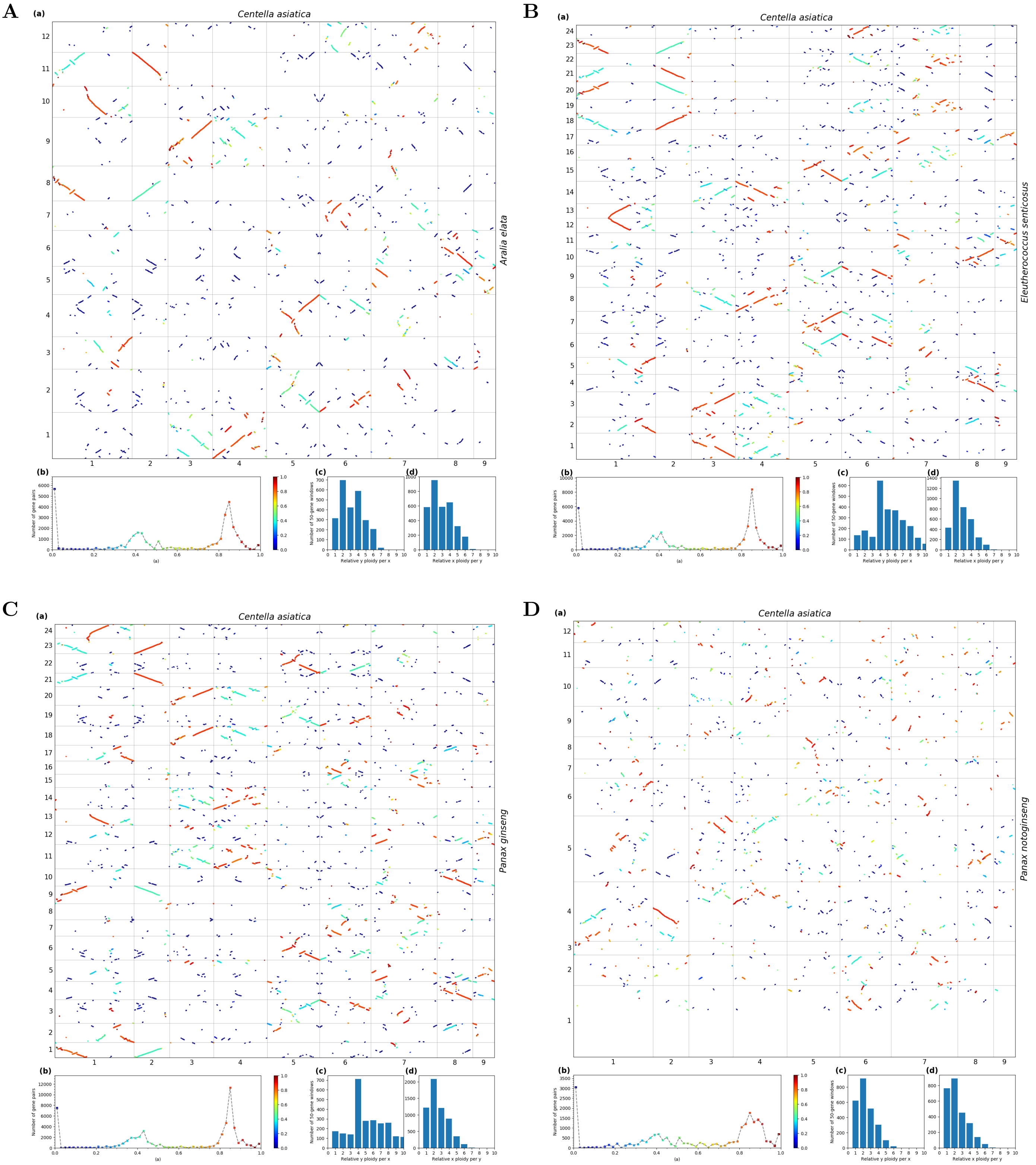

Figure S95. *Orthology Index*-colored dot plots showing orthologous syntenic relationships between *Centella asiatica* and Araliaceae (*Centella asiatica* : *Aralia elata* = 1:1, *Centella asiatica* : *Eleutherococcus senticosus* = 1:2, *Centella asiatica* : *Panax ginseng*=1:2, *Centella asiatica* : *Panax notoginseng*=1:1).

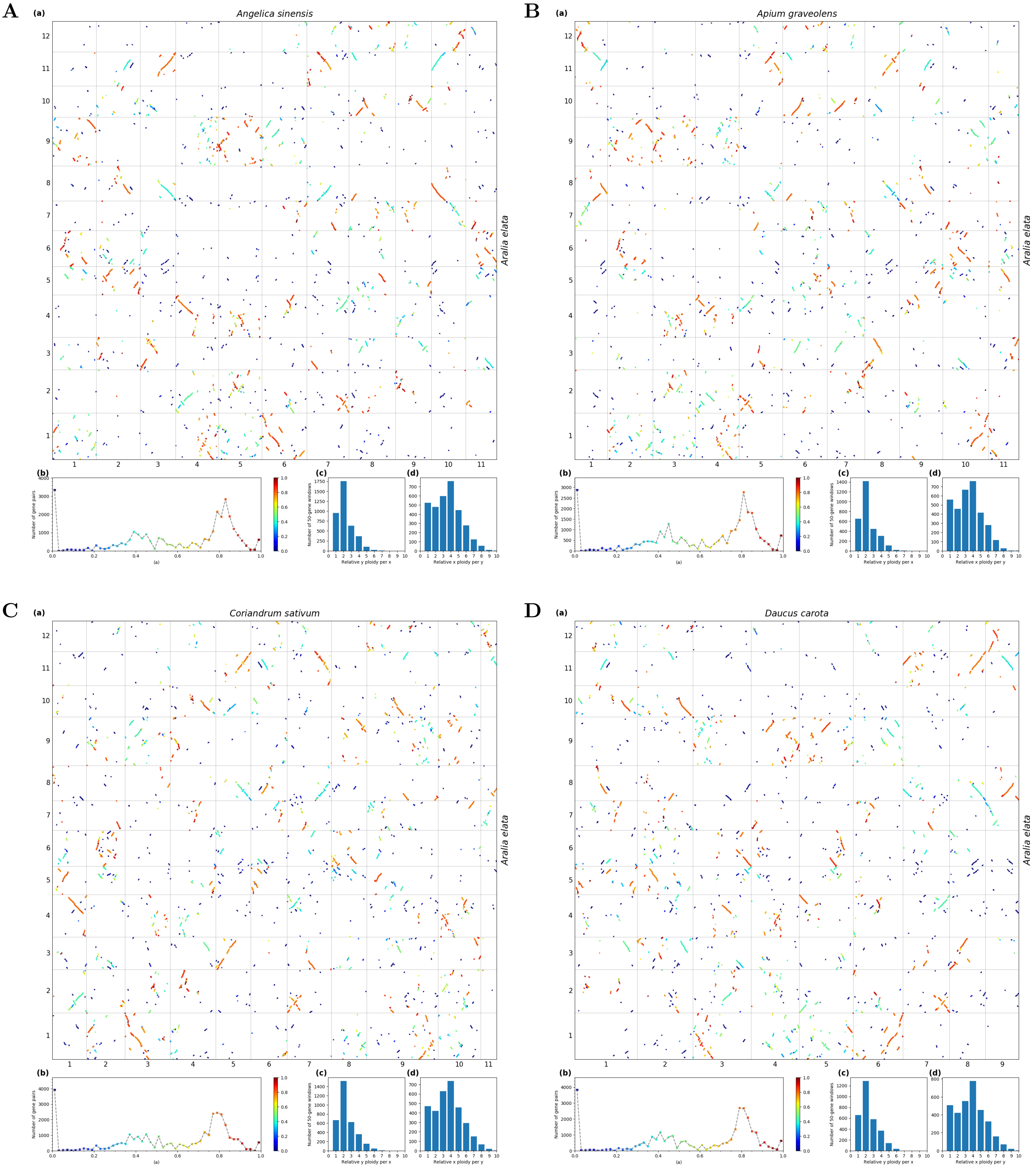

Figure S96. *Orthology Index*-colored dot plots showing orthologous syntenic relationships between *Aralia elata* and Apioideae (all 1:2).

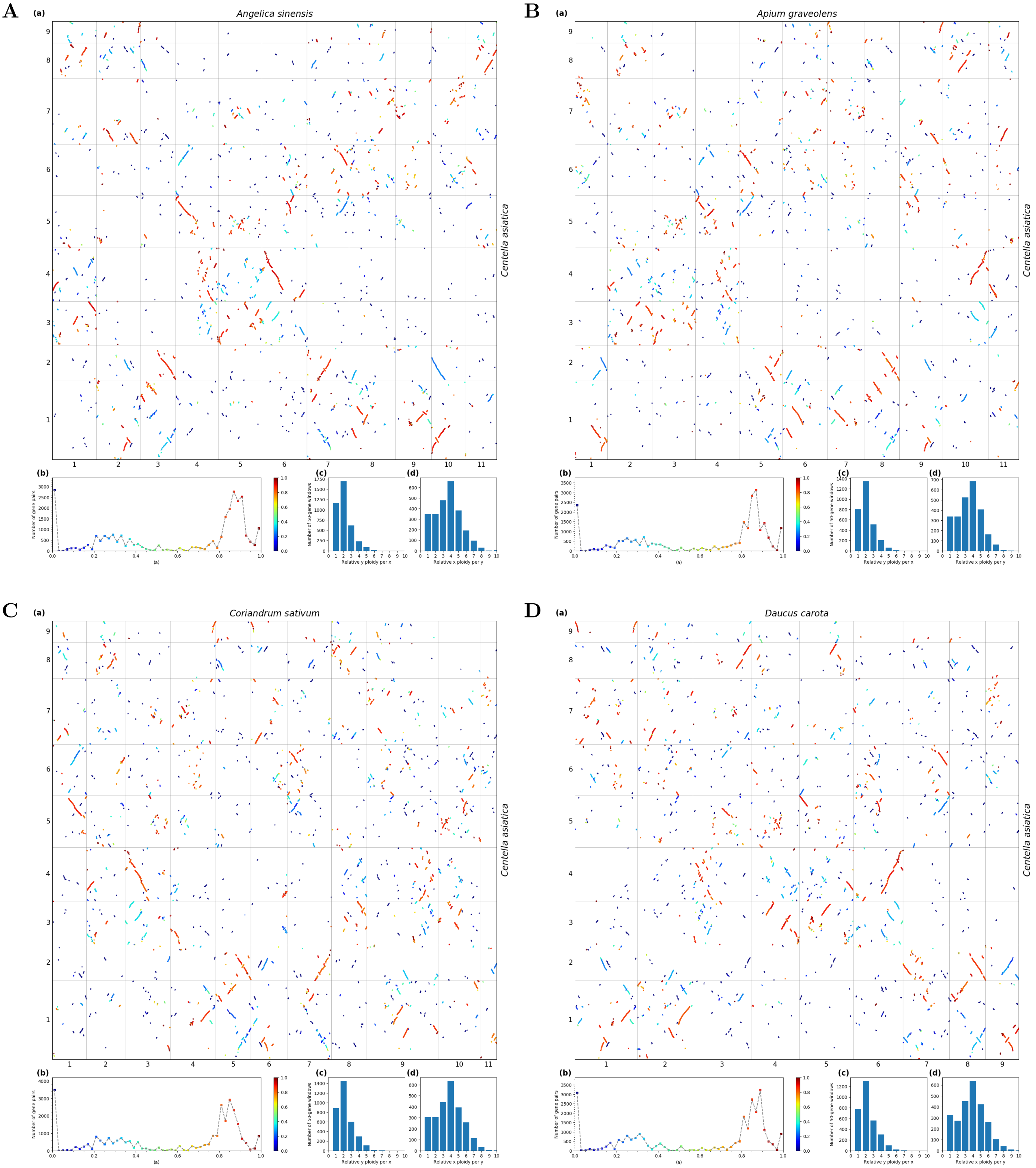

Figure S97. *Orthology Index*-colored dot plots showing orthologous syntenic relationships between *Centella asiatica* and Apioideae (all 1:2).

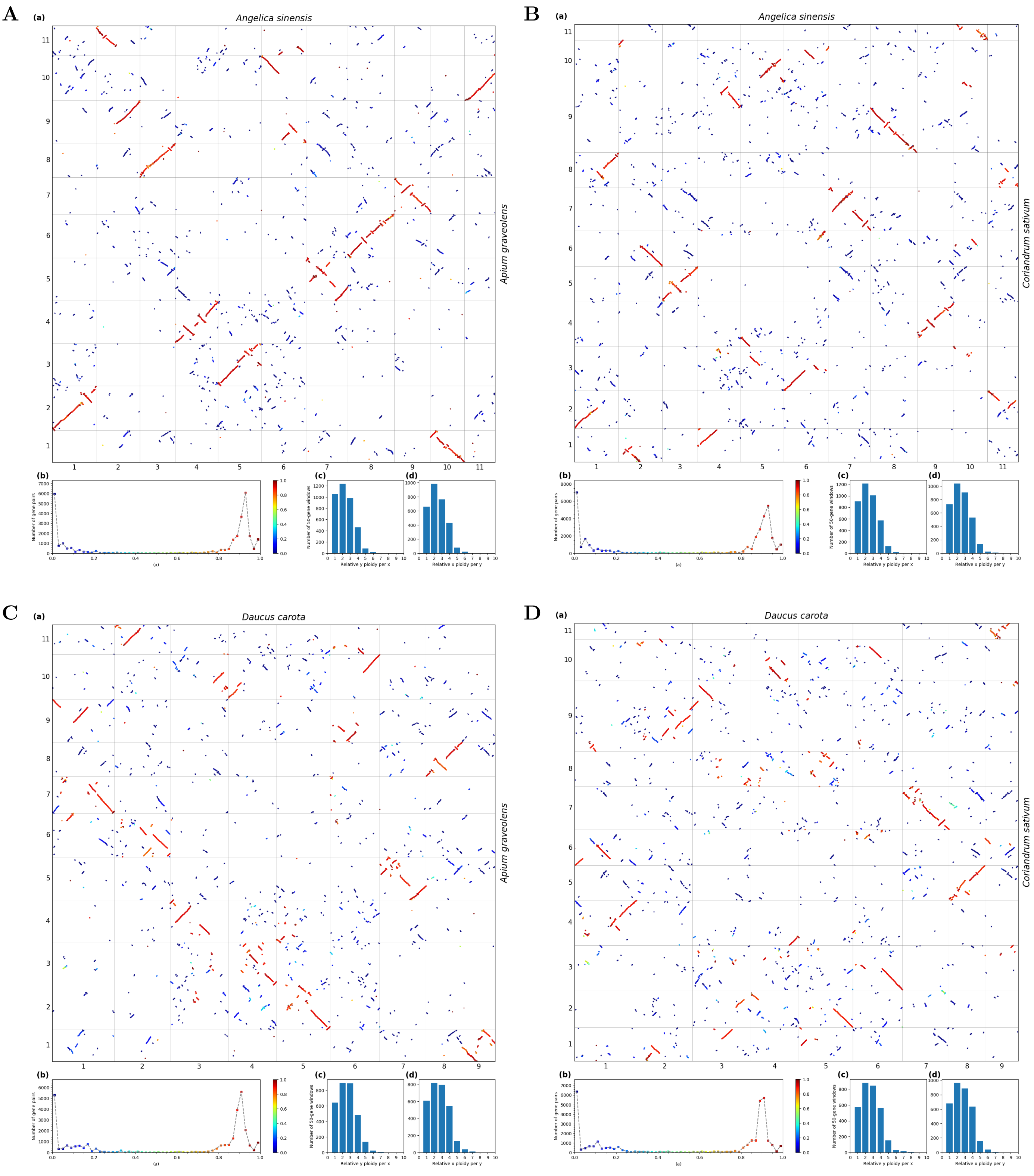

Figure S98. *Orthology Index*-colored dot plots showing orthologous syntenic relationships within Apioideae (all 1:1).

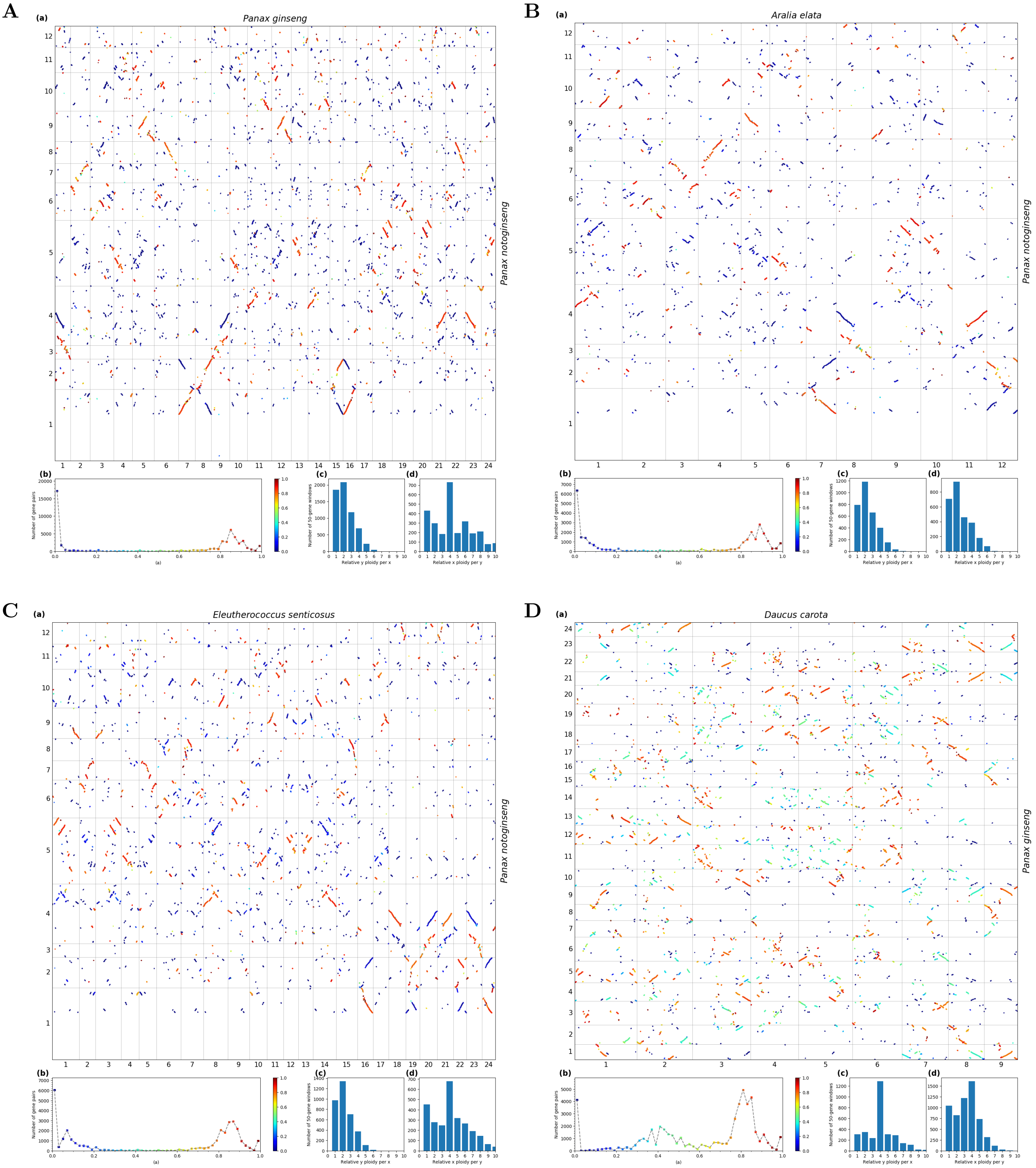

Figure S99. *Orthology Index*-colored dot plots showing orthologous syntenic relationships within Araliaceae (*Panax ginseng* : *Panax notoginseng* = 2:1, *Aralia elata* : *Panax notoginseng* = 1:1, *Eleutherococcus senticosus* : *Panax notoginseng* = 2:1). The other orthologous syntenic depth ratios (*Aralia elata*: *Eleutherococcus senticosus* = 1:2, *Aralia elata*: *Panax ginseng* = 1:2, *Eleutherococcus senticosus*: *Panax ginseng* = 2:2) can be found in Fig. S57-S59.

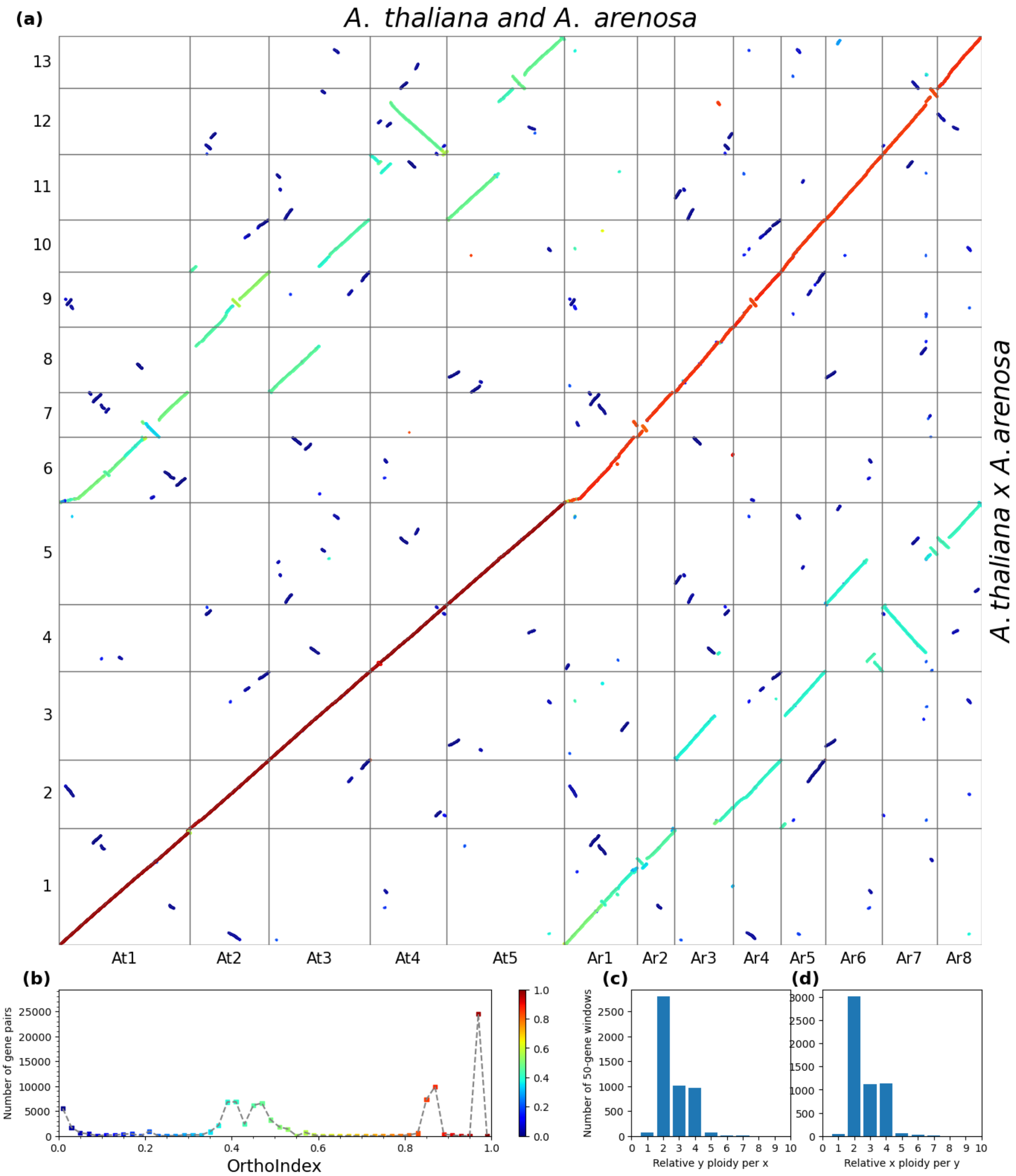

Figure S100. *Orthology Index*-colored dot plots showing orthologous syntenic relationships between *Arabidopsis thaliana* x *A.arenosa* and *A. thaliana* + *A. arenosa*.

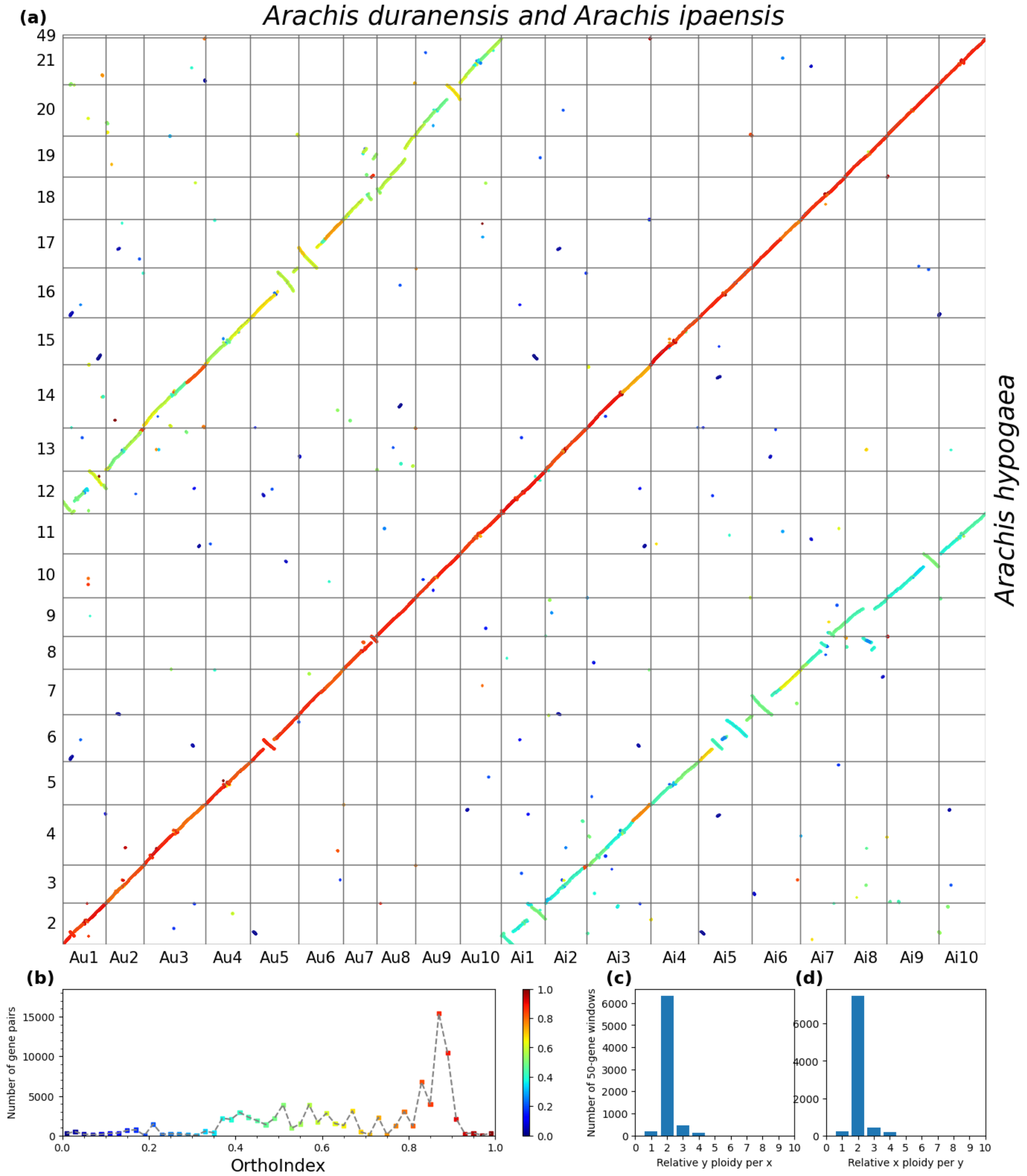

Figure S101. *Orthology Index*-colored dot plots showing orthologous syntenic relationships between *Arachis hypogaea* and *A. duranensis* + *A. ipaensis*.

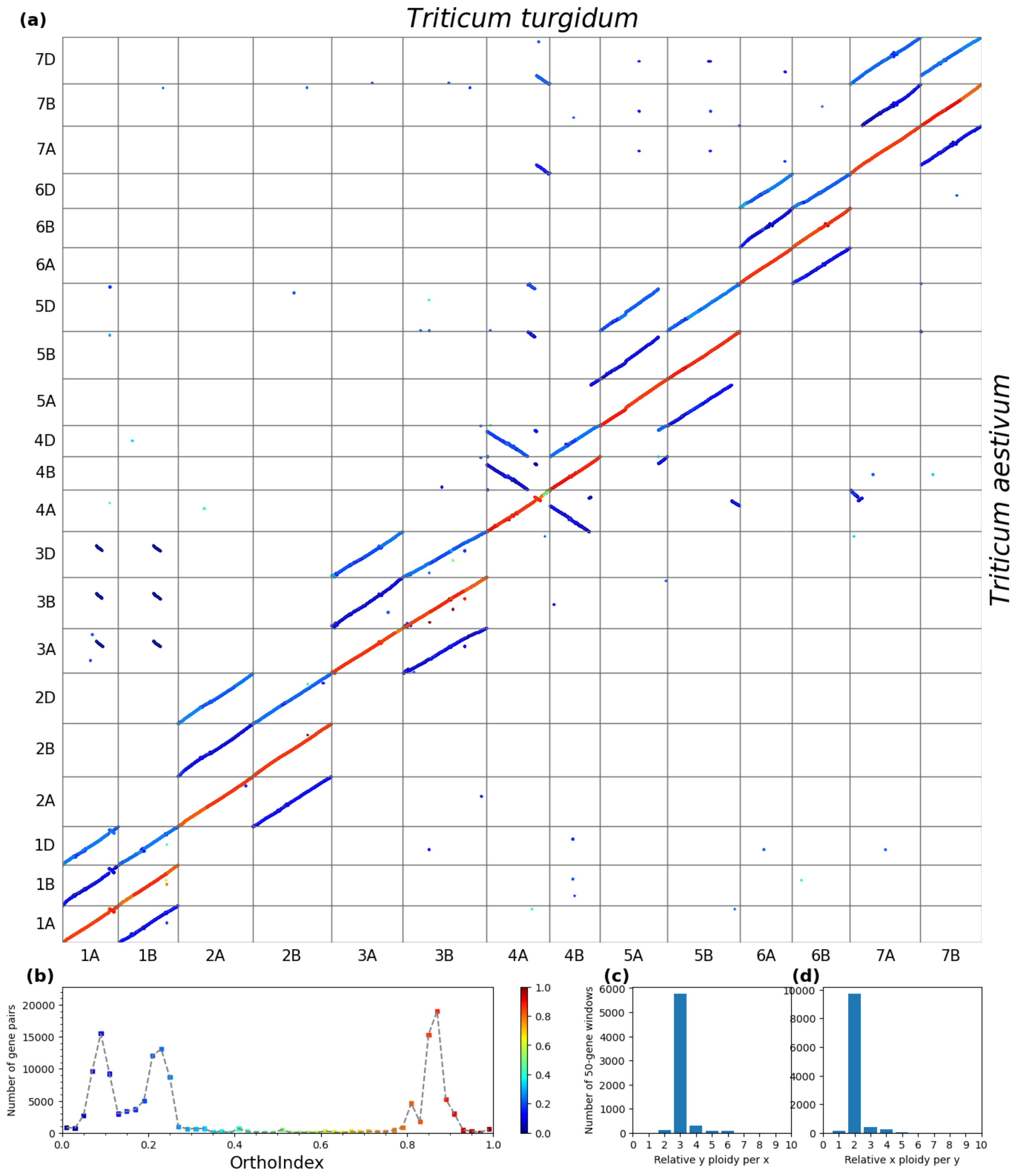

Figure S102. *Orthology Index*-colored dot plots showing orthologous syntenic relationships between *Triticum turgidum* and *T. aestivum.*

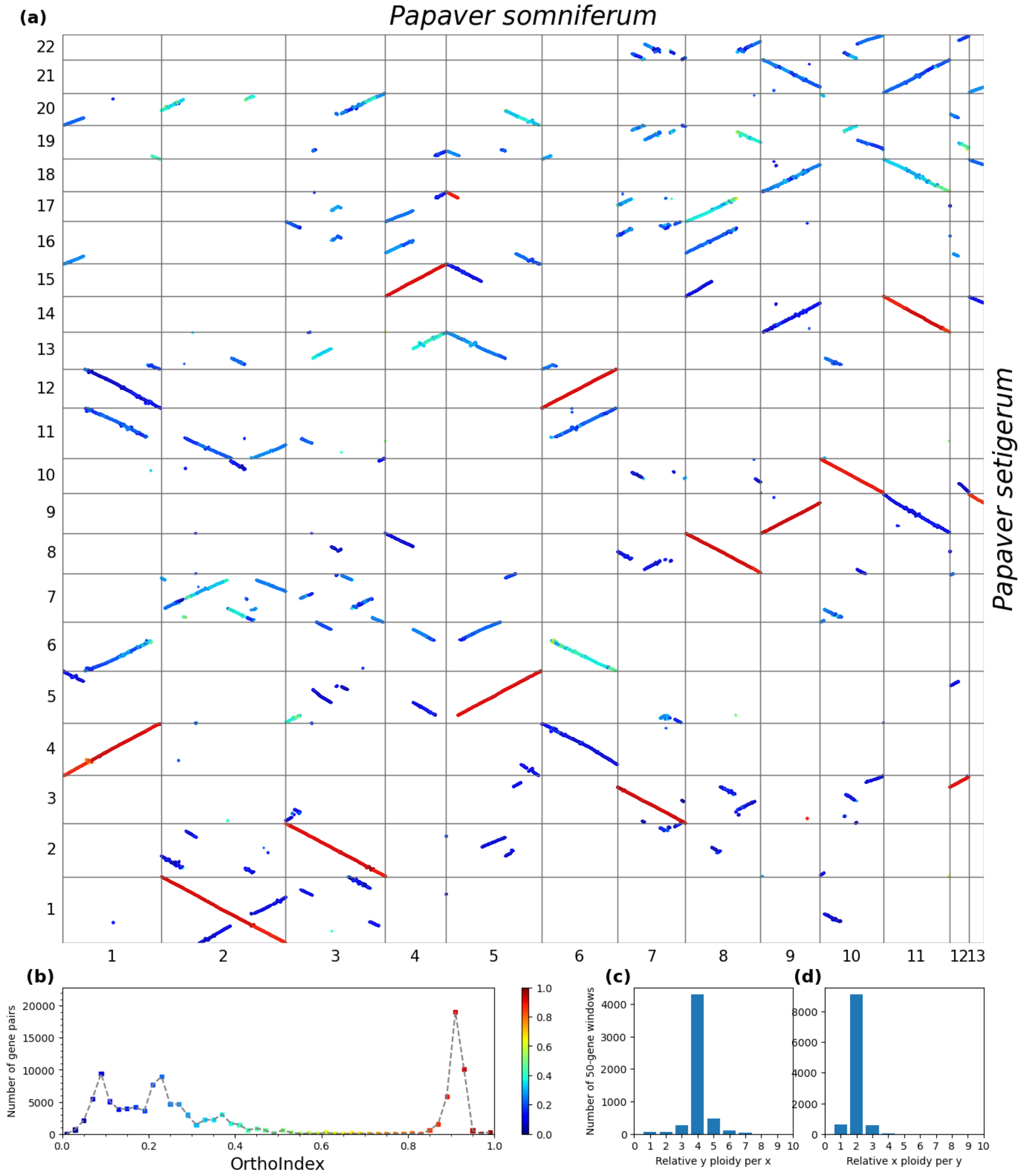

Figure S103. *Orthology Index*-colored dot plots showing orthologous syntenic relationships between *Papaver somniferum* and *P. setigerum*.

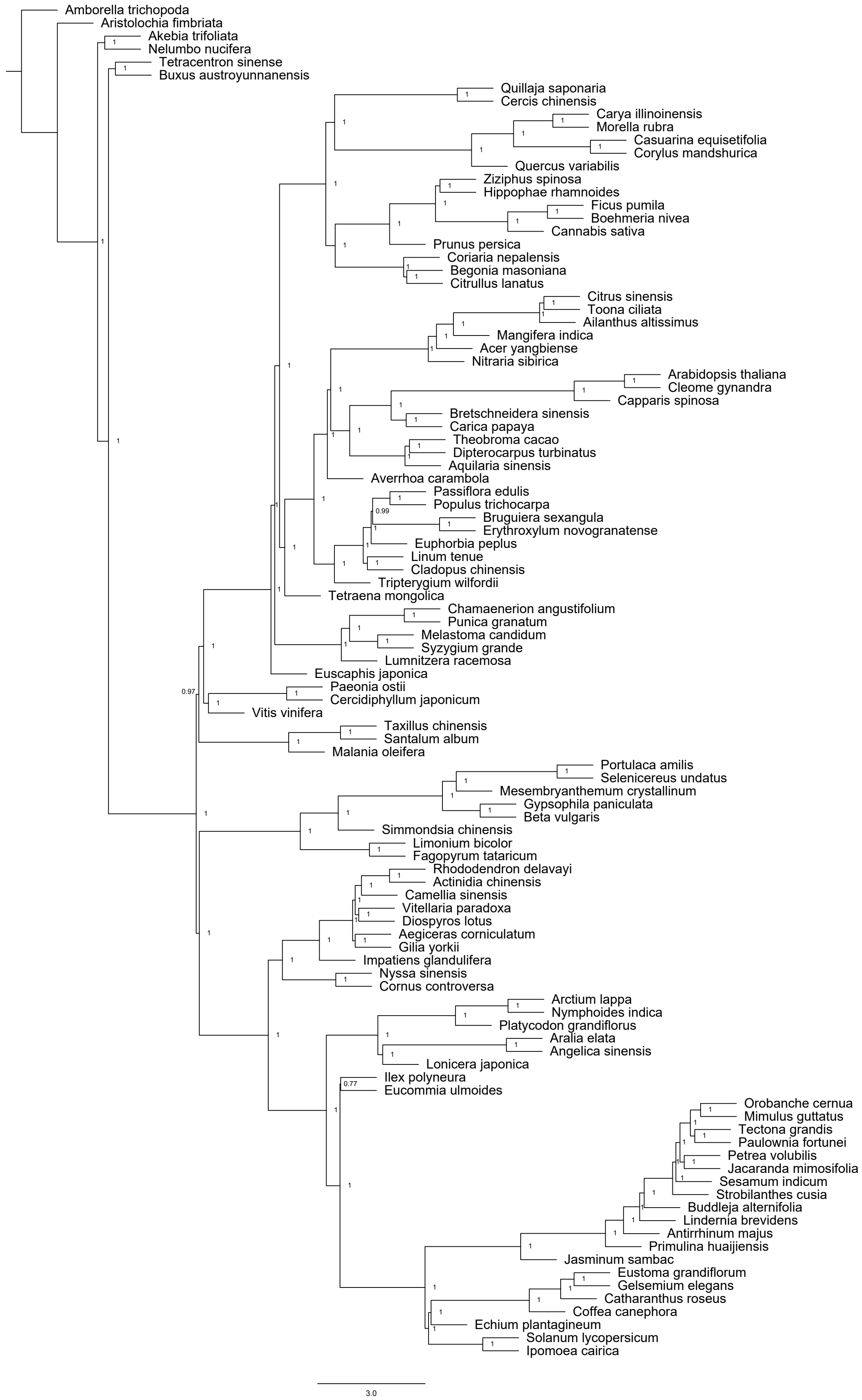

Figure S104. Phylogenetic relationships within the core eudicots based on 12,277 multi-copy SOGs. The numbers at the nodes are posterior probabilities from ASTRAL. Bar, 3.0 coalescent units.

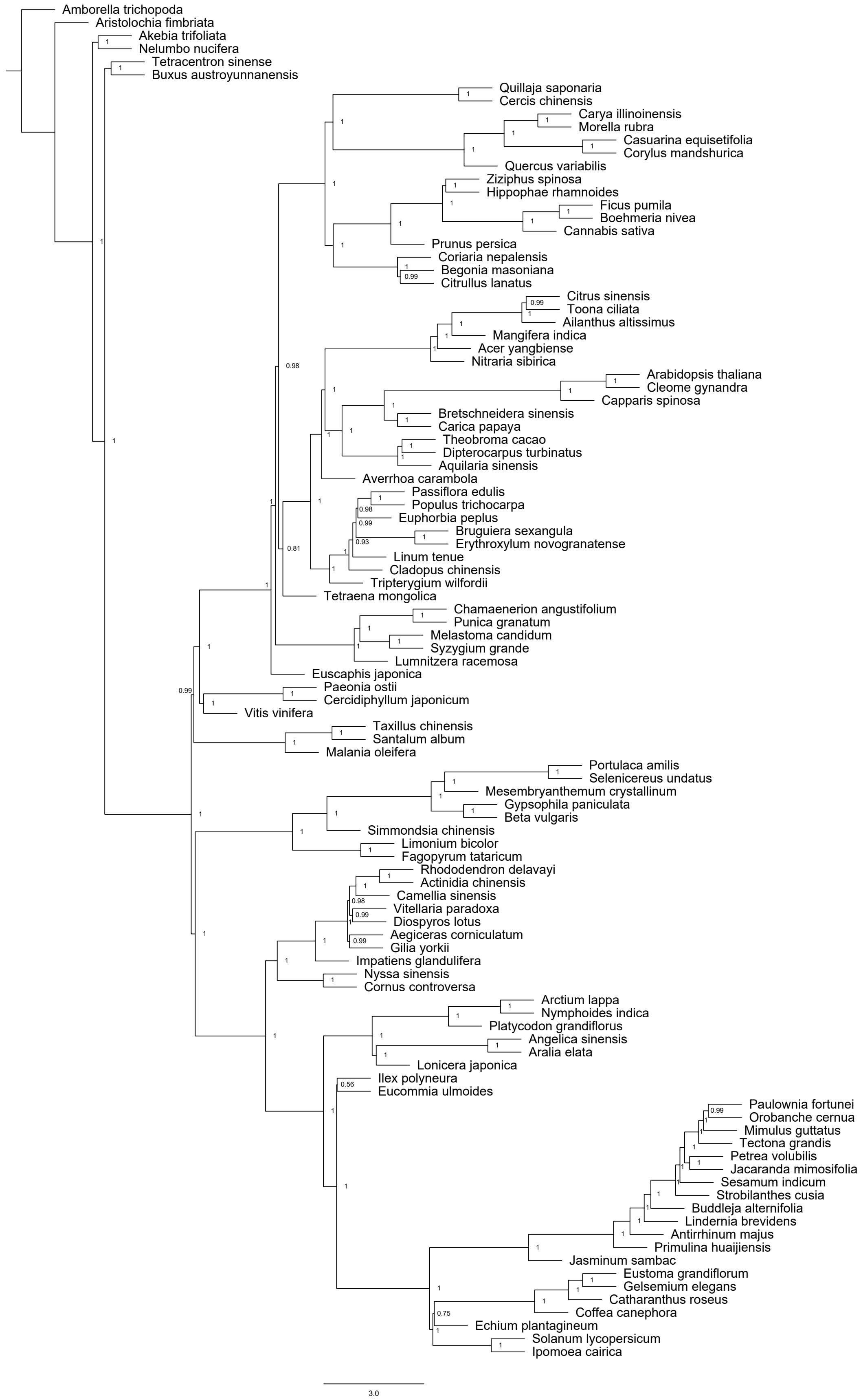

Figure S105. Phylogenetic relationships within the core eudicots based on 5,154 single-copy SOGs. The numbers at the nodes are posterior probabilities from ASTRAL. Bar, 3.0 coalescent units.

Table S1. Cases with shared polyploidization event(s) used in this study.

Orthology	Lineage	Out-paralogy	In-paralogy	Reference
Liriodendron chinense-Cinnamomum kanehirae	Magnoliidae	1 WGD	1 WGD in Cka	(1)
Liriodendron chinense-Magnolia biondii	Magnoliaceae	1 WGD	None	(2)
Cinnamomum kanehirae-Chimonanthus salicifolius	Laurales	1 WGD	1 WGD in Cka<br>1 WGD in Csa	(3)
Cinnamomum kanehirae-Phoebe bournei	Lauraceae	2 WGDs	None	(4)
Protea cynaroides-Telopea speciosissima	Proteaceae	1 WGD	None	(5)
Protea cynaroides-Macadamia integrifolia	Proteaceae	1 WGD	None	(5)
Aquilegia coerulea-Coptis chinensis	Ranunculaceae	1 WGD	None	(6)
Aquilegia coerulea-Papaver somniferum	Ranunculales	1 WGD	1 WGD in Pso	(6)
Corydalis tomentella-Papaver somniferum	Papaveraceae	1 WGD	1 WGD in Pso	(7)
Tetracendron sinense-Trochodendron aralioides	Trochodendraceae	2 WGDs	None	(8)
Vitis vinifera-Theobroma cacao	Pentapetalae	1 WGT	None	(9)
Vitis vinifera-Populus trichocarpa	Pentapetalae	1 WGT	1 WGD in Ptr	(10)
Vitis vinifera-Arabidopsis thaliana	Pentapetalae	1 WGT	2 WGDs in Ath	(10)
Vitis vinifera-Solanum lycopersicum	Pentapetalae	1 WGT	1 WGT in Sly	(11)
Vitis vinifera-Malania oleifera	Pentapetalae	1 WGT	None	(12)
Vitis vinifera-Spinacia oleracea	Pentapetalae	1 WGT	None	(13)
Vitis vinifera-Camellia sinensis	Pentapetalae	1 WGT	1 WGD in Csi	(14)
Vitis vinifera-Coffea canephora	Pentapetalae	1 WGT	None	(15)
Vitis vinifera-Eucommia ulmoides	Pentapetalae	1 WGT	1 WGD in Eul	(16)
Vitis vinifera-Daucus carota	Pentapetalae	1 WGT	2 WGDs in Dca	(17)
Vitis vinifera-Cercidiphyllum japonicum	Pentapetalae	1 WGT	None	(18)
Vitis vinifera-Paeonia ostii	Pentapetalae	1 WGT	None	(19)
Vitis vinifera-Prunus persica	Pentapetalae	1 WGT	None	(20)
Vitis vinifera-Tripterygium wilfordii	Pentapetalae	1 WGT	1 WGT in Twi	(21)
Vitis vinifera-Betula pendula	Pentapetalae	1 WGT	None	(22)
Vitis vinifera-Euscaphis japonica	Pentapetalae	1 WGT	1 WGD in Eja	(23)
Vitis vinifera-Carica papaya	Pentapetalae	1 WGT	None	(24)
Vitis vinifera-Acer yangbiense	Pentapetalae	1 WGT	None	(25)
Populus trichocarpa-Arabidopsis thaliana	Rosidae	1 WGT	1 WGD in Ptr<br>2 WGDs in Ath	(10)
Amaranthus tricolor-Amaranthus hypochondriacus	Amaranthaceae	1 WGT, 1 WGD	None	(26)
Fagopyrum tataricum-Fagopyrum esculentum	Polygonaceae	1 WGT, 2 WGDs	None	(27)
Santalum album-Santalum yasi	Santalaceae	2 WGTs	None	(28)
Rhododendron simsii-Vaccinium corymbosum	Ericaceae	1 WGT, 1 WGD	None	(29)

Rhododendron simsii-Actinidia chinensis	Ericaceae	1 WGT, 1 WGD	1 WGD in Ach	(29)
Rhododendron simsii-Camptotheca acuminata	Ericales-Cornales	1 WGT	1 WGD in Rsi<br>1 WGD in Cac	(29)
Davidia involucrata-Camptotheca acuminata	Nyssaceae	1 WGT, 1 WGD	None	(30)
Paulownia fortunei-Sesamum indicum	Lamiales	1 WGT, 1 WGD	None	(31)
Orobanche cernua-Mimulus guttatus	Lamiales	1 WGT, 1 WGD	None	(32)
Tectona grandis-Jacaranda mimosifolia	Lamiales	1 WGT, 1 WGD	None	(33)
Tectona grandis-Salvia splendens	Lamiaceae	1 WGT, 1 WGD	2 WGDs in Ssp	(33)
Salvia splendens-Salvia hispanica	Lamiaceae	1 WGT, 2 WGDs	1 WGD in Ssp	(34)
Jasminum sambac-Osmanthus fragrans	Oleaceae	2 WGTs	1 WGD in Oeu	(35)
Syringa oblata-Osmanthus fragrans	Oleaceae	2 WGTs, 1 WGD	None	(36)
Coffea canephora-Marsdenia tenacissima	Gentianales	1 WGT	None	(37)
Coffea canephora-Ophiorrhiza pumila	Rubiaceae	1 WGT	None	(38)
Ophiorrhiza pumila-Neolamarckia cadamba	Rubiaceae	1 WGT	1 WGD in Nca	(39)
Solanum lycopersicum-Anisodus acutangulus	Solanaceae	2 WGTs	1 WGD in Aac	(40)
Solanum lycopersicum-Nicotiana tabacum	Solanaceae	2 WGTs	1 WGD in Nta	(41)
Solanum lycopersicum-Lycium barbarum	Solanaceae	2 WGTs	None	(42)
Ipomoea cairica-Cuscuta europaea	Convolvulaceae	2 WGTs	None	(43)
Arctium lappa-Taraxacum mongolicum	Asteraceae	2 WGTs	None	(44)
Mikania micrantha-Helianthus annuus	Asteraceae	2 WGTs, 1 WGD	None	(45)
Mikania micrantha-Stevia rebaudiana	Asteraceae	2 WGTs, 1 WGD	None	(45)
Mikania micrantha-Smallanthus sonchifolius	Asteraceae	2 WGTs, 1 WGD	1 WGD in Sso	(45)
Helianthus annuus-Smallanthus sonchifolius	Asteraceae	2 WGTs, 1 WGD	1 WGD in Sso	(45)
Daucus carota-Angelica sinensis	Apiaceae	1 WGT, 2 WGDs	None	(46)
Aralia elata-Eleutherococcus senticosus	Araliaceae	1 WGT, 1 WGD	1 WGD in Ese	(47)
Aralia elata-Panax ginseng	Araliaceae	1 WGT, 1 WGD	1 WGD in Pgi	(47)
Panax ginseng-Eleutherococcus senticosus	Araliaceae	1 WGT, 1 WGD	1 WGD in Pgi<br>1 WGD in Ese	(47)
Lupinus albus-Lupinus angustifolius	Fabaceae	1 WGT, 1 WGD, 1 WGT	None	(48)
Glycine max-Glycine soja	Fabaceae	1 WGT, 2 WGDs	None	(49)
Glycine max-Arachis hypogaea	Fabaceae	1 WGT, 1 WGD	1 WGD in Ahy<br>1 WGD in Gma	(50)
Arachis hypogaea-Arachis monticola	Fabaceae	1 WGT, 2 WGDs	None	(51)
Dalbergia odorifera-Glycine max	Fabaceae	1 WGT, 1 WGD	1 WGD in Gma	(52)
Gillenia trifoliata-Crataegus pinnatifida	Rosaceae	1 WGT	1 WGD in Cpi	(53)
Crataegus pinnatifida-Malus x domestica	Rosaceae	1 WGT, 1 WGD	None	(53)
Carya illinoinensis-Juglans regia	Juglandaceae	1 WGT, 1 WGD	None	(54)

Citrullus lanatus-Begonia loranthoides	Cucurbitales	1 WGT, 1 WGD	1 WGD in Bma	(55)
Citrullus lanatus-Luffa cylindrica	Cucurbitaceae	1 WGT, 1 WGD	None	(56)
Citrullus lanatus-Cucurbita argyrosperma	Cucurbitaceae	1 WGT, 1 WGD	1 WGD in Car	(55)
Cucurbita argyrosperma-Cucurbita pepo	Cucurbitaceae	1 WGT, 2 WGDs	None	(55)
Begonia loranthoides-Begonia peltatifolia	Begoniaceae	1 WGT, 2 WGDs	None	(55)
Bruguiera parviflora-Ceriops tagal	Rhizophoraceae	1 WGT, 1 WGD	None	(57)
Hevea brasiliensis-Manihot esculenta	Euphorbiaceae	1 WGT, 1 WGD	None	(58)
Populus trichocarpa-Populus euphratica	Salicaceae	1 WGT, 1 WGD	None	(59)
Salix dunnii-Salix brachista	Salicaceae	1 WGT, 1 WGD	None	(59)
Eucalyptus grandis-Punica granatum	Myrtales	1 WGT, 1 WGD	None	(60)
Eucalyptus grandis-Syzygium grande	Myrtaceae	1 WGT, 1 WGD	None	(60)
Melastoma candidum-Melastoma dodecandrum	Melastomataceae	1 WGT, 3 WGDs	None	(61)
Arabidopsis thaliana-Capparis spinosa	Brassicales	1 WGT, 1 WGD	1 WGD in Ath<br>1 WGD in Csp	(62)
Arabidopsis thaliana-Aethionema arabicum	Brassicaceae	1 WGT, 2 WGDs	None	(63)
Arabidopsis thaliana-Thlaspi arvense	Brassicaceae	1 WGT, 2 WGDs	None	(64)
Arabidopsis thaliana-Brassica rapa	Brassicaceae	1 WGT, 2 WGDs	1 WGT in Bra	(65)
Brassica rapa-Sinapis alba	Brassicaceae	1 WGT, 2 WGDs, 1 WGT	None	(66)
Brassica rapa-Crambe hispanica	Brassicaceae	1 WGT, 2 WGDs, 1 WGT	None	(67)
Theobroma cacao-Gossypium raimondii	Malvaceae	1 WGT	1 WGM in Gra	(68)
Gossypium raimondii-Gossypioides kirkii	Malvaceae	1 WGT, 1 WGM	None	(69)
Aquilaria sinensis-Stellera chamaejasme	Thymelaeaceae	1 WGT, 1 WGD	None	(70)
Mangifera indica-Anacardium occidentale	Anacardiaceae	1 WGT, 1 WGD	None	(71)
Toona sinensis-Toona ciliata	Meliaceae	1 WGT, 1 WGD	None	(72)

Note: WGD, whole genome duplication; WGT, whole genome triplication; WGM, whole genome multiplication.

Table S2. Genomic data used in this study (accessed before 2023-06-29).

Species	Order	Family	Source
Amborella trichopoda	Amborellales	Amborellaceae	(73)
Angelica sinensis	Apiales	Apiaceae	(46)
Apium graveolens	Apiales	Apiaceae	(17)
Centella asiatica	Apiales	Apiaceae	(74)
Coriandrum sativum	Apiales	Apiaceae	(75)
Daucus carota	Apiales	Apiaceae	(76)
Aralia elata	Apiales	Araliaceae	(47)
Eleutherococcus senticosus	Apiales	Araliaceae	(77)
Panax ginseng	Apiales	Araliaceae	(78)
Panax notoginseng	Apiales	Araliaceae	(79)
Ilex polyneura	Aquifoliales	Aquifoliaceae	(80)
Arctium lappa	Asterales	Asteraceae	(45)
Helianthus annuus	Asterales	Asteraceae	(81)
Mikania micrantha	Asterales	Asteraceae	(82)
Smallanthus sonchifolius	Asterales	Asteraceae	(45)
Stevia rebaudiana	Asterales	Asteraceae	(83)
Taraxacum mongolicum	Asterales	Asteraceae	(84)
Platycodon grandiflorus	Asterales	Campanulaceae	(85)
Nymphoides indica	Asterales	Menyanthaceae	(86)
Echium plantagineum	Boraginales	Boraginaceae	(87)
Bretschneidera sinensis	Brassicales	Akaniaceae	(88)
Aethionema arabicum	Brassicales	Brassicaceae	(89)
Arabidopsis arenosa	Brassicales	Brassicaceae	Genbank: GCA_905216605
Arabidopsis thaliana	Brassicales	Brassicaceae	(90)
Arabidopsis thaliana x *Arabidopsis arenosa*	Brassicales	Brassicaceae	(91)
Brassica rapa	Brassicales	Brassicaceae	(92)
Crambe hispanica	Brassicales	Brassicaceae	(67)
Sinapis alba	Brassicales	Brassicaceae	(66)
Thlaspi arvense	Brassicales	Brassicaceae	(64)
Capparis spinosa	Brassicales	Capparaceae	(62)
Carica papaya	Brassicales	Caricaceae	(93)
Cleome gynandra	Brassicales	Cleomaceae	(94)
Buxus austroyunnanensis	Buxales	Buxaceae	(95)
Mesembryanthemum crystallinum	Caryophyllales	Aizoaceae	(96)
Amaranthus hypochondriacus	Caryophyllales	Amaranthaceae	(97)
Amaranthus tricolor	Caryophyllales	Amaranthaceae	(26)
Selenicereus undatus	Caryophyllales	Cactaceae	(98)
Gypsophila paniculata	Caryophyllales	Caryophyllaceae	(99)
Beta vulgaris	Caryophyllales	Chenopodiaceae	(100)
Spinacia oleracea	Caryophyllales	Chenopodiaceae	(101)
Limonium bicolor	Caryophyllales	Plumbaginaceae	(102)

Fagopyrum esculentum	Caryophyllales	Polygonaceae	GWH: GWHBJBK00000000
Fagopyrum tataricum	Caryophyllales	Polygonaceae	GWH: GWHBJBL00000000
Portulaca amilis	Caryophyllales	Portulacaceae	(103)
Simmondsia chinensis	Caryophyllales	Simmondsiaceae	(104)
Tripterygium wilfordii	Celastrales	Celastraceae	(21)
Cornus controversa	Cornales	Cornaceae	(105)
Camptotheca acuminata	Cornales	Nyssaceae	(106)
Davidia involucrata	Cornales	Nyssaceae	(30)
Nyssa sinensis	Cornales	Nyssaceae	(107)
Euscaphis japonica	Crossosomatales	Staphyleaceae	(23)
Begonia loranthoides	Cucurbitales	Begoniaceae	(108)
Begonia masoniana	Cucurbitales	Begoniaceae	(108)
Begonia peltatifolia	Cucurbitales	Begoniaceae	(108)
Coriaria nepalensis	Cucurbitales	Coriariaceae	(109)
Citrullus lanatus	Cucurbitales	Cucurbitaceae	(110)
Cucurbita argyrosperma	Cucurbitales	Cucurbitaceae	(111)
Cucurbita pepo	Cucurbitales	Cucurbitaceae	(112)
Luffa cylindrica	Cucurbitales	Cucurbitaceae	(113)
Lonicera japonica	Dipsacales	Caprifoliaceae	(114)
Actinidia chinensis	Ericales	Actinidiaceae	(115)
Impatiens glandulifera	Ericales	Balsaminaceae	Refseq: GCF_ 907164915
Diospyros lotus	Ericales	Ebenaceae	(116)
Rhododendron delavayi	Ericales	Ericaceae	(117)
Rhododendron simsii	Ericales	Ericaceae	(29)
Vaccinium corymbosum	Ericales	Ericaceae	(118)
Gilia yorkii	Ericales	Polemoniaceae	(119)
Aegiceras corniculatum	Ericales	Primulaceae	(120)
Vitellaria paradoxa	Ericales	Sapotaceae	(121)
Camellia sinensis	Ericales	Theaceae	(122)
Arachis duranensis	Fabales	Fabaceae	(123)
Arachis hypogaea	Fabales	Fabaceae	(50)
Arachis ipaensis	Fabales	Fabaceae	(123)
Arachis monticola	Fabales	Fabaceae	(51)
Cercis chinensis	Fabales	Fabaceae	(124)
Dalbergia odorifera	Fabales	Fabaceae	(52)
Glycine max	Fabales	Fabaceae	(125)
Glycine soja	Fabales	Fabaceae	(125)
Lupinus albus	Fabales	Fabaceae	(126)
Lupinus angustifolius	Fabales	Fabaceae	(48)
Quillaja saponaria	Fabales	Quillajaceae	(127)
Betula pendula	Fagales	Betulaceae	(22)
Corylus mandshurica	Fagales	Betulaceae	(128)
Casuarina equisetifolia	Fagales	Casuarinaceae	(129)
Quercus variabilis	Fagales	Fagaceae	(130)

Carya illinoinensis	Fagales	Juglandaceae	(131)
Juglans regia	Fagales	Juglandaceae	(132)
Morella rubra	Fagales	Myricaceae	(133)
Eucommia ulmoides	Garryales	Eucommiaceae	(16)
Catharanthus roseus	Gentianales	Apocynaceae	(134)
Marsdenia tenacissima	Gentianales	Apocynaceae	(37)
Gelsemium elegans	Gentianales	Gelsemiaceae	(135)
Eustoma grandiflorum	Gentianales	Gentianaceae	(136)
Coffea canephora	Gentianales	Rubiaceae	(15)
Neolamarckia cadamba	Gentianales	Rubiaceae	(39)
Ophiorrhiza pumila	Gentianales	Rubiaceae	(38)
Strobilanthes cusia	Lamiales	Acanthaceae	(137)
Jacaranda mimosifolia	Lamiales	Bignoniaceae	(138)
Primulina huaijiensis	Lamiales	Gesneriaceae	(139)
Salvia hispanica	Lamiales	Lamiaceae	(34)
Salvia splendens	Lamiales	Lamiaceae	(33)
Tectona grandis	Lamiales	Lamiaceae	(140)
Lindernia brevidens	Lamiales	Linderniaceae	(141)
Jasminum sambac	Lamiales	Oleaceae	(35)
Osmanthus fragrans	Lamiales	Oleaceae	(142)
Syringa oblata	Lamiales	Oleaceae	(143)
Orobanche cernua	Lamiales	Orobanchaceae	(32)
Paulownia fortunei	Lamiales	Paulowniaceae	(31)
Sesamum indicum	Lamiales	Pedaliaceae	(144)
Mimulus guttatus	Lamiales	Phrymaceae	(145)
Antirrhinum majus	Lamiales	Plantaginaceae	(146)
Buddleja alternifolia	Lamiales	Scrophulariaceae	(147)
Petrea volubilis	Lamiales	Verbenaceae	(148)
Chimonanthus salicifolius	Laurales	Calycanthaceae	(3)
Cinnamomum kanehirae	Laurales	Lauraceae	(149)
Phoebe bournei	Laurales	Lauraceae	(4)
Liriodendron chinense	Magnoliales	Magnoliaceae	(150)
Magnolia biondii	Magnoliales	Magnoliaceae	(2)
Erythroxylum novogranatense	Malpighiales	Erythroxylaceae	(40)
Euphorbia peplus	Malpighiales	Euphorbiaceae	(151)
Hevea brasiliensis	Malpighiales	Euphorbiaceae	(58)
Manihot esculenta	Malpighiales	Euphorbiaceae	(152)
Linum tenue	Malpighiales	Linaceae	(153)
Passiflora edulis	Malpighiales	Passifloraceae	(154)
Cladopus chinensis	Malpighiales	Podostemaceae	(155)
Bruguiera parviflora	Malpighiales	Rhizophoraceae	(57)
Bruguiera sexangula	Malpighiales	Rhizophoraceae	(156)
Ceriops tagal	Malpighiales	Rhizophoraceae	(157)
Populus euphratica	Malpighiales	Salicaceae	(158)

Populus trichocarpa	Malpighiales	Salicaceae	(159)
Salix brachista	Malpighiales	Salicaceae	(160)
Salix dunnii	Malpighiales	Salicaceae	(161)
Dipterocarpus turbinatus	Malvales	Dipterocarpaceae	(162)
Gossypioides kirkii	Malvales	Malvaceae	(163)
Gossypium raimondii	Malvales	Malvaceae	(69)
Theobroma cacao	Malvales	Malvaceae	(164)
Aquilaria sinensis	Malvales	Thymelaeaceae	(165)
Stellera chamaejasme	Malvales	Thymelaeaceae	(70)
Lumnitzera racemosa	Myrtales	Combretaceae	(166)
Punica granatum	Myrtales	Lythraceae	(167)
Melastoma candidum	Myrtales	Melastomataceae	(61)
Melastoma dodecandrum	Myrtales	Melastomataceae	(168)
Eucalyptus grandis	Myrtales	Myrtaceae	(169)
Syzygium grande	Myrtales	Myrtaceae	(60)
Chamaenerion angustifolium	Myrtales	Onagraceae	Genbank: GCA_946814005
Averrhoa carambola	Oxalidales	Oxalidaceae	(170)
Aristolochia fimbriata	Piperales	Aristolochiaceae	(1)
Triticum aestivum	Poales	Poaceae	(171)
Triticum turgidum	Poales	Poaceae	(172)
Nelumbo nucifera	Proteales	Nelumbonaceae	(173)
Macadamia integrifolia	Proteales	Proteaceae	(174)
Protea cynaroides	Proteales	Proteaceae	(5)
Telopea speciosissima	Proteales	Proteaceae	(175)
Akebia trifoliata	Ranunculales	Lardizabalaceae	(176)
Corydalis tomentella	Ranunculales	Papaveraceae	(7)
Papaver setigerum	Ranunculales	Papaveraceae	(177)
Papaver somniferum	Ranunculales	Papaveraceae	(177)
Aquilegia coerulea	Ranunculales	Ranunculaceae	(178)
Coptis chinensis	Ranunculales	Ranunculaceae	(179)
Cannabis sativa	Rosales	Cannabaceae	(180)
Hippophae rhamnoides	Rosales	Elaeagnaceae	(181)
Ficus pumila	Rosales	Moraceae	(182)
Ziziphus spinosa	Rosales	Rhamnaceae	(183)
Crataegus pinnatifida	Rosales	Rosaceae	(53)
Fragaria iinumae	Rosales	Rosaceae	(184)
Fragaria nipponica	Rosales	Rosaceae	(185)
Fragaria vesca	Rosales	Rosaceae	(186)
Fragaria viridis	Rosales	Rosaceae	(187)
Fragaria x ananassa	Rosales	Rosaceae	(188)
Gillenia trifoliata	Rosales	Rosaceae	(189)
Malus x domestica	Rosales	Rosaceae	(190)
Prunus persica	Rosales	Rosaceae	(20)
Boehmeria nivea	Rosales	Urticaceae	(191)

Taxillus chinensis	Santalales	Loranthaceae	(192)
Santalum album	Santalales	Santalaceae	(28)
Santalum yasi	Santalales	Santalaceae	(28)
Malania oleifera	Santalales	Ximeniaceae	(193)
Anacardium occidentale	Sapindales	Anacardiaceae	JGI
Mangifera indica	Sapindales	Anacardiaceae	(194)
Toona ciliata	Sapindales	Meliaceae	(195)
Toona sinensis	Sapindales	Meliaceae	(196)
Nitraria sibirica	Sapindales	Nitrariaceae	(197)
Citrus sinensis	Sapindales	Rutaceae	(198)
Acer yangbiense	Sapindales	Sapindaceae	(25)
Ailanthus altissimus	Sapindales	Simaroubaceae	Genbank:GCA_946807835
Cercidiphyllum japonicum	Saxifragales	Cercidiphyllaceae	(18)
Paeonia ostii	Saxifragales	Paeoniaceae	(19)
Cuscuta europaea	Solanales	Convolvulaceae	(199)
Ipomoea cairica	Solanales	Convolvulaceae	(43)
Anisodus acutangulus	Solanales	Solanaceae	(40)
Lycium barbarum	Solanales	Solanaceae	(200)
Nicotiana tabacum	Solanales	Solanaceae	(41)
Solanum lycopersicum	Solanales	Solanaceae	(201)
Tetracentron sinense	Trochodendrales	Trochodendraceae	(8)
Trochodendron aralioides	Trochodendrales	Trochodendraceae	(202)
Vitis vinifera	Vitales	Vitaceae	(203)
Tetraena mongolica	Zygophyllales	Zygophyllaceae	(204)

Note: Genbank/Refseq: https://www.ncbi.nlm.nih.gov/assembly/; GWH: https://ngdc.cncb.ac.cn/gwh/; JGI: https://phytozome-next.jgi.doe.gov/.

References

1. Qin, L., Hu, Y., Wang, J., Wang, X., Zhao, R., Shan, H., Li, K., Xu, P., Wu, H. and Yan, X. *et al.*. (2021) Insights into angiosperm evolution, floral development and chemical biosynthesis from the *Aristolochia fimbriata* genome. *Nat. Plants*, **7**, 1239–1253.
2. Dong, S., Liu, M., Liu, Y., Chen, F., Yang, T., Chen, L., Zhang, X., Guo, X., Fang, D. and Li, L. *et al.*. (2021) The genome of *Magnolia biondii* Pamp. provides insights into the evolution of Magnoliales and biosynthesis of terpenoids. *Hortic. Res.*, **8**, 38.
3. Lv, Q., Qiu, J., Liu, J., Li, Z., Zhang, W., Wang, Q., Fang, J., Pan, J., Chen, Z. and Cheng, W. *et al.*. (2020) The *Chimonanthus salicifolius* genome provides insight into magnoliid evolution and flavonoid biosynthesis. *Plant J.*, **103**, 1910–1923.
4. Han, X., Zhang, J., Han, S., Chong, S.L., Meng, G., Song, M., Wang, Y., Zhou, S., Liu, C. and Lou, L. *et al.*. (2022) The chromosome-scale genome of *Phoebe bournei* reveals contrasting fates of terpene synthase (TPS)-a and TPS-b subfamilies. *Plant Commun.*, **3**, 100410.
5. Chang, J., Duong, T.A., Schoeman, C., Ma, X., Roodt, D., Barker, N., Li, Z., Van de Peer, Y. and Mizrachi, E. (2023) The genome of the king protea, *Protea cynaroides*. *Plant J.*, **113**, 262–276.
6. Zhang, Y., Ge, W., Teng, J., Yang, Y., Wang, J., Yu, Z., Wang, J., Xiao, Q., Zhao, J. and Shen, S. *et al.*. (2023) Allotetraploidization event of *Coptis chinensis* shared by all Ranunculales. *Hortic. Plant J.*
7. Xu, Z., Li, Z., Ren, F., Gao, R., Wang, Z., Zhang, J., Zhao, T., Ma, X., Pu, X. and Xin, T. *et al.*. (2022) The genome of *Corydalis* reveals the evolution of benzylisoquinoline alkaloid biosynthesis in Ranunculales. *Plant J.*, **111**, 217–230.
8. Liu, P., Zhang, X., Mao, J., Hong, Y., Zhang, R., E, Y., Nie, S., Jia, K., Jiang, C. and He, J. *et al.*. (2020) The *Tetracentron* genome provides insight into the early evolution of eudicots and the formation of vessel elements. *Genome Biol.*, **21**, 291.
9. Argout, X., Salse, J., Aury, J., Guiltinan, M.J., Droc, G., Gouzy, J., Allegre, M., Chaparro, C., Legavre, T. and Maximova, S.N. *et al.*. (2011) The genome of *Theobroma cacao*. *Nat. Genet.*, **43**, 101–108.
10. Tang, H., Bowers, J.E., Wang, X., Ming, R., Alam, M. and Paterson, A.H. (2008) Synteny and collinearity in plant genomes. *Science*, **320**, 486–488.
11. Shusei, S., Satoshi, T., Hideki, H., Erika, A., Kenta, S., Sachiko, I., Takakazu, K., Yasukazu, N., Daisuke, S. and Koh, A. (2012) The tomato genome sequence provides insights into fleshy fruit evolution. *Nature*, **485**, 635–641.
12. Xu, C., Liu, H., Zhou, S., Zhang, D., Zhao, W., Wang, S., Chen, F., Sun, Y., Nie, S. and Jia, K. *et al.*. (2019) Genome sequence of *Malania oleifera*, a tree with great value for nervonic acid production. *Gigascience*, **8**, giy164.
13. Xu, C., Jiao, C., Sun, H., Cai, X., Wang, X., Ge, C., Zheng, Y., Liu, W., Sun, X. and Xu, Y. *et al.*. (2017) Draft genome of spinach and transcriptome diversity of 120 *Spinacia* accessions. *Nat. Commun.*, **8**, 15275.
14. Xia, E., Zhang, H., Sheng, J., Li, K., Zhang, Q., Kim, C., Zhang, Y., Liu, Y., Zhu, T. and Li, W. *et al.*. (2017) The tea tree genome provides insights into tea flavor and independent evolution of caffeine biosynthesis. *Mol. Plant.*, **10**, 866–877.
15. Denoeud, F., Carretero-Paulet, L., Dereeper, A., Droc, G., Guyot, R., Pietrella, M., Zheng, C., Alberti, A., Anthony, F. and Aprea, G. *et al.*. (2014) The coffee genome provides insight into the convergent evolution of caffeine biosynthesis. *Science*, **345**, 1181–1184.

16. Li, Y., Wei, H., Yang, J., Du, K., Li, J., Zhang, Y., Qiu, T., Liu, Z., Ren, Y. and Song, L. *et al.*. (2020) High-quality *de novo* assembly of the *Eucommia ulmoides* haploid genome provides new insights into evolution and rubber biosynthesis. *Hortic. Res.*, **7**, 183.
17. Song, X., Sun, P., Yuan, J., Gong, K., Li, N., Meng, F., Zhang, Z., Li, X., Hu, J. and Wang, J. *et al.*. (2021) The celery genome sequence reveals sequential paleo-polyploidizations, karyotype evolution and resistance gene reduction in apiales. *Plant Biotechnol. J.*, **19**, 731–744.
18. Zhu, S., Chen, J., Zhao, J., Comes, H.P., Li, P., Fu, C., Xie, X., Lu, R., Xu, W. and Feng, Y. *et al.*. (2020) Genomic insights on the contribution of balancing selection and local adaptation to the long-term survival of a widespread living fossil tree, *Cercidiphyllum japonicum*. *New Phytol.*, **228**, 1674–1689.
19. Yuan, J., Jiang, S., Jian, J., Liu, M., Yue, Z., Xu, J., Li, J., Xu, C., Lin, L. and Jing, Y. *et al.*. (2022) Genomic basis of the giga-chromosomes and giga-genome of tree peony *Paeonia ostii*. *Nat. Commun.*, **13**, 7328.
20. The International Peach Genome Initiative. (2013) The high-quality draft genome of peach (*Prunus persica*) identifies unique patterns of genetic diversity, domestication and genome evolution. *Nat. Genet.*, **45**, 487–494.
21. Tu, L., Su, P., Zhang, Z., Gao, L., Wang, J., Hu, T., Zhou, J., Zhang, Y., Zhao, Y. and Liu, Y. *et al.*. (2020) Genome of *Tripterygium wilfordii* and identification of cytochrome P450 involved in triptolide biosynthesis. *Nat. Commun.*, **11**, 971.
22. Salojärvi, J., Smolander, O.P., Nieminen, K., Rajaraman, S., Safronov, O., Safdari, P., Lamminmäki, A., Immanen, J., Lan, T. and Tanskanen, J. (2017) Genome sequencing and population genomic analyses provide insights into the adaptive landscape of silver birch. *Nat. Genet.*, **49**, 904.
23. Sun, W.H., Li, Z., Xiang, S., Ni, L., Zhang, D., Chen, D.Q., Qiu, M.Y., Zhang, Q.G., Xiao, L. and Din, L. *et al.*. (2021) The *Euscaphis japonica* genome and the evolution of malvids. *Plant J.*, **108**, 1382–1399.
24. Ming, R., Hou, S., Feng, Y., Yu, Q., Dionne-Laporte, A., Saw, J.H., Senin, P., Wang, W., Ly, B.V. and Lewis, K.L. *et al.*. (2008) The draft genome of the transgenic tropical fruit tree papaya (*Carica papaya* Linnaeus). *Nature*, **452**, 991–996.
25. Yang, J., Wariss, H.M., Tao, L., Zhang, R., Yun, Q., Hollingsworth, P., Dao, Z., Luo, G., Guo, H. and Ma, Y. *et al.*. (2019) *De novo* genome assembly of the endangered *Acer yangbiense*, a plant species with extremely small populations endemic to Yunnan Province, China. *Gigascience*, **8**, giz85.
26. Wang, H., Xu, D., Wang, S., Wang, A., Lei, L., Jiang, F., Yang, B., Yuan, L., Chen, R. and Zhang, Y. *et al.*. (2023) Chromosome-scale *Amaranthus tricolor* genome provides insights into the evolution of the genus *Amaranthus* and the mechanism of betalain biosynthesis. *DNA Res.*, **30**, dsac50.
27. Lin, H., Yao, Y., Sun, P., Feng, L., Wang, S., Ren, Y., Yu, X., Xi, Z. and Liu, J. (2023) Haplotype-resolved genomes of two buckwheat crops provide insights into their contrasted rutin concentrations and reproductive systems. *BMC Biol.*, **21**, 87.
28. Hong, Z., Peng, D., Tembrock, L.R., Liao, X., Xu, D., Liu, X. and Wu, Z. (2023) Chromosome-level genome assemblies from two sandalwood species provide insights into the evolution of the Santalales. *Commun. Biol.*, **6**, 587.
29. Yang, F., Nie, S., Liu, H., Shi, T., Tian, X., Zhou, S., Bao, Y., Jia, K., Guo, J. and Zhao, W. *et al.*.

(2020) Chromosome-level genome assembly of a parent species of widely cultivated azaleas. *Nat. Commun.*, **11**, 5269.

30. Chen, Y., Ma, T., Zhang, L., Kang, M., Zhang, Z., Zheng, Z., Sun, P., Shrestha, N., Liu, J. and Yang, Y. (2020) Genomic analyses of a "living fossil": The endangered dove-tree. *Mol. Ecol. Resour.*, **20**, 756–769.
31. Cao, Y., Sun, G., Zhai, X., Xu, P., Ma, L., Deng, M., Zhao, Z., Yang, H., Dong, Y. and Shang, Z. *et al.*. (2021) Genomic insights into the fast growth of paulownias and the formation of *Paulownia* witches' broom. *Mol. Plant.*, **14**, 1668–1682.
32. Xu, Y., Zhang, J., Ma, C., Lei, Y., Shen, G., Jin, J., Eaton, D.A.R. and Wu, J. (2022) Comparative genomics of orobanchaceous species with different parasitic lifestyles reveals the origin and stepwise evolution of plant parasitism. *Mol. Plant.*, **15**, 1384–1399.
33. Jia, K., Liu, H., Zhang, R., Xu, J., Zhou, S., Jiao, S., Yan, X., Tian, X., Shi, T. and Luo, H. *et al.*. (2021) Chromosome-scale assembly and evolution of the tetraploid *Salvia splendens* (Lamiaceae) genome. *Hortic. Res.*, **8**, 177.
34. Wang, L., Lee, M., Sun, F., Song, Z., Yang, Z. and Yue, G.H. (2022) A chromosome-level genome assembly of chia provides insights into high omega-3 content and coat color variation of its seeds. *Plant Commun.*, **3**, 100326.
35. Xu, S., Ding, Y., Sun, J., Zhang, Z., Wu, Z., Yang, T., Shen, F. and Xue, G. (2022) A high-quality genome assembly of *Jasminum sambac* provides insight into floral trait formation and Oleaceae genome evolution. *Mol. Ecol. Resour.*, **22**, 724–739.
36. Ma, B., Wu, J., Shi, T., Yang, Y., Wang, W., Zheng, Y., Su, S., Yao, Y., Xue, W. and Porth, I. *et al.*. (2022) Lilac (*Syringa oblata*) genome provides insights into its evolution and molecular mechanism of petal color change. *Commun. Biol.*, **5**, 686.
37. Zhou, Y., Fan, W., Zhang, H., Zhang, J., Zhang, G., Wang, D., Xiang, G., Zhao, C., Li, L. and He, S. *et al.*. (2023) *Marsdenia tenacissima* genome reveals calcium adaptation and tenacissoside biosynthesis. *Plant J.*, **113**, 1146–1159.
38. Rai, A., Hirakawa, H., Nakabayashi, R., Kikuchi, S., Hayashi, K., Rai, M., Tsugawa, H., Nakaya, T., Mori, T. and Nagasaki, H. *et al.*. (2021) Chromosome-level genome assembly of *Ophiorrhiza pumila* reveals the evolution of camptothecin biosynthesis. *Nat. Commun.*, **12**, 405.
39. Zhao, X., Hu, X., OuYang, K., Yang, J., Que, Q., Long, J., Zhang, J., Zhang, T., Wang, X. and Gao, J. *et al.*. (2022) Chromosome-level assembly of the *Neolamarckia cadamba* genome provides insights into the evolution of cadambine biosynthesis. *Plant J.*, **109**, 891–908.
40. Wang, Y., Tain, T., Yu, J., Li, J., Xu, B., Chen, J., D Auria, J.C., Huang, J. and Huang, S. (2023) Genomic and structural basis for evolution of tropane alkaloid biosynthesis. *Proc. Natl. Acad. Sci. U. S. A.*, **120**, e1992519176.
41. Edwards, K.D., Fernandez-Pozo, N., Drake-Stowe, K., Humphry, M., Evans, A.D., Bombarely, A., Allen, F., Hurst, R., White, B. and Kernodle, S.P. *et al.*. (2017) A reference genome for *Nicotiana tabacum* enables map-based cloning of homeologous loci implicated in nitrogen utilization efficiency. *BMC Genomics*, **18**, 448.
42. Cao, Y., Li, Y., Fan, Y., Li, Z., Yoshida, K., Wang, J., Ma, X., Wang, N., Mitsuda, N. and Kotake, T. *et al.*. (2021) Wolfberry genomes and the evolution of *Lycium* (Solanaceae). *Commun. Biol.*, **4**, 671.
43. Jiang, F., Wang, S., Wang, H., Wang, A., Xu, D., Liu, H., Yang, B., Yuan, L., Lei, L. and Chen, R. *et al.*. (2022) A chromosome-level reference genome of a Convolvulaceae species *Ipomoea*

cairica. *G3-Genes Genomes Genet.*, **12**, jkac187.

44. Kong, X., Zhang, Y., Wang, Z., Bao, S., Feng, Y., Wang, J., Yu, Z., Long, F., Xiao, Z. and Hao, Y. *et al.*. (2023) Two-step model of paleohexaploidy, ancestral genome reshuffling and plasticity of heat shock response in Asteraceae. *Hortic. Res.*, **10**, uhad73.
45. Fan, W., Wang, S., Wang, H., Wang, A., Jiang, F., Liu, H., Zhao, H., Xu, D. and Zhang, Y. (2022) The genomes of chicory, endive, great burdock and yacon provide insights into Asteraceae palaeo-polyploidization history and plant inulin production. *Mol. Ecol. Resour.*, **22**, 3124–3140.
46. Han, X., Li, C., Sun, S., Ji, J., Nie, B., Maker, G., Ren, Y. and Wang, L. (2022) The chromosome-level genome of female ginseng (*Angelica sinensis*) provides insights into molecular mechanisms and evolution of coumarin biosynthesis. *Plant J.*, **112**, 1224–1237.
47. Wang, Y., Zhang, H., Ri, H.C., An, Z., Wang, X., Zhou, J., Zheng, D., Wu, H., Wang, P. and Yang, J. *et al.*. (2022) Deletion and tandem duplications of biosynthetic genes drive the diversity of triterpenoids in *Aralia elata*. *Nat. Commun.*, **13**, 2224.
48. Hane, J.K., Ming, Y., Kamphuis, L.G., Nelson, M.N., Garg, G., Atkins, C.A., Bayer, P.E., Bravo, A., Bringans, S. and Cannon, S. *et al.*. (2017) A comprehensive draft genome sequence for lupin (*Lupinus angustifolius*), an emerging health food: insights into plant-microbe interactions and legume evolution. *Plant Biotechnol. J.*, **15**, 318–330.
49. Schmutz, J., Cannon, S.B., Schlueter, J., Ma, J., Mitros, T., Nelson, W., Hyten, D.L., Song, Q., Thelen, J.J. and Cheng, J. *et al.*. (2010) Genome sequence of the palaeopolyploid soybean. *Nature*, **463**, 178–183.
50. Bertioli, D.J., Jenkins, J., Clevenger, J., Dudchenko, O., Gao, D., Seijo, G., Leal-Bertioli, S.C.M., Ren, L., Farmer, A.D. and Pandey, M.K. *et al.*. (2019) The genome sequence of segmental allotetraploid peanut *Arachis hypogaea*. *Nat. Genet.*, **51**, 877–884.
51. Yin, D., Ji, C., Ma, X., Li, H., Zhang, W., Li, S., Liu, F., Zhao, K., Li, F. and Li, K. *et al.*. (2018) Genome of an allotetraploid wild peanut *Arachis monticola*: a *de novo* assembly. *Gigascience*, **7**, giy66.
52. Hong, Z., Li, J., Liu, X., Lian, J., Zhang, N., Yang, Z., Niu, Y., Cui, Z. and Xu, D. (2020) The chromosome-level draft genome of *Dalbergia odorifera*. *Gigascience*, **9**, giaa84.
53. Zhang, T., Qiao, Q., Du, X., Zhang, X., Hou, Y., Wei, X., Sun, C., Zhang, R., Yun, Q. and Crabbe, M.J.C. *et al.*. (2022) Cultivated hawthorn (*Crataegus pinnatifida* var. *major*) genome sheds light on the evolution of Maleae (apple tribe). *J. Integr. Plant Biol.*, **64**, 1487–1501.
54. Xiao, L., Yu, M., Zhang, Y., Hu, J., Zhang, R., Wang, J., Guo, H., Zhang, H., Guo, X. and Deng, T. *et al.*. (2021) Chromosome-scale assembly reveals asymmetric paleo-subgenome evolution and targets for the acceleration of fungal resistance breeding in the nut crop, pecan. *Plant Commun.*, **2**, 100247.
55. Wang, J., Yuan, M., Feng, Y., Zhang, Y., Bao, S., Hao, Y., Ding, Y., Gao, X., Yu, Z. and Xu, Q. *et al.*. (2022) A common whole-genome paleotetraploidization in Cucurbitales. *Plant Physiol.*, **190**, 2430–2448.
56. Wu, H., Zhao, G., Gong, H., Li, J., Luo, C., He, X., Luo, S., Zheng, X., Liu, X. and Guo, J. *et al.*. (2020) A high-quality sponge gourd (*Luffa cylindrica*) genome. *Hortic. Res.*, **7**, 128.
57. Pootakham, W., Sonthirod, C., Naktang, C., Kongkachana, W., Sangsrakru, D., U Thoomporn, S., Maknual, C., Meepol, W., Promchoo, W. and Maprasop, P. *et al.*. (2022) A chromosome-scale reference genome assembly of yellow mangrove (*Bruguiera parviflora*) reveals a whole genome duplication event associated with the Rhizophoraceae lineage. *Mol. Ecol. Resour.*, **22**, 1939–1953.

58. Liu, J., Shi, C., Shi, C., Li, W., Zhang, Q., Zhang, Y., Li, K., Lu, H., Shi, C. and Zhu, S. *et al.*. (2020) The chromosome-based rubber tree genome provides new insights into spurge genome evolution and rubber biosynthesis. *Mol. Plant.*, **13**, 336–350.
59. Dai, X., Hu, Q., Cai, Q., Feng, K., Ye, N., Tuskan, G.A., Milne, R., Chen, Y., Wan, Z. and Wang, Z. (2014) The willow genome and divergent evolution from poplar after the common genome duplication. *Cell Res.*, **24**, 1274.
60. Low, Y.W., Rajaraman, S., Tomlin, C.M., Ahmad, J.A., Ardi, W.H., Armstrong, K., Athen, P., Berhaman, A., Bone, R.E. and Cheek, M. *et al.*. (2022) Genomic insights into rapid speciation within the world's largest tree genus *Syzygiu*m. *Nat. Commun.*, **13**, 5031.
61. Zhong, Y., Wu, W., Sun, C., Zou, P., Liu, Y., Dai, S. and Zhou, R. (2023) Chromosomal-level genome assembly of *Melastoma candidum* provides insights into trichome evolution. *Front. Plant Sci.*, **14**, 1126319.
62. Wang, L., Fan, L., Zhao, Z., Zhang, Z., Jiang, L., Chai, M. and Tian, C. (2022) The *Capparis spinosa* var. *herbacea* genome provides the first genomic instrument for a diversity and evolution study of the Capparaceae family. *Gigascience*, **11**, giac106.
63. Haudry, A., Platts, A.E., Vello, E., Hoen, D.R., Leclercq, M., Williamson, R.J., Forczek, E., Joly-Lopez, Z., Steffen, J.G. and Hazzouri, K.M. *et al.*. (2013) An atlas of over 90,000 conserved noncoding sequences provides insight into crucifer regulatory regions. *Nat. Genet.*, **45**, 891–898.
64. Geng, Y., Guan, Y., Qiong, L., Lu, S., An, M., Crabbe, M.J.C., Qi, J., Zhao, F., Qiao, Q. and Zhang, T. (2021) Genomic analysis of field pennycress (*Thlaspi arvense*) provides insights into mechanisms of adaptation to high elevation. *BMC Biol.*, **19**, 143.
65. Wang, X., Wang, H., Wang, J., Sun, R., Wu, J., Liu, S., Bai, Y., Mun, J., Bancroft, I. and Cheng, F. *et al.*. (2011) The genome of the mesopolyploid crop species *Brassica rapa*. *Nat. Genet.*, **43**, 1035–1039.
66. Yang, T., Cai, B., Jia, Z., Wang, Y., Wang, J., King, G.J., Ge, X. and Li, Z. (2023) *Sinapis* genomes provide insights into whole-genome triplication and divergence patterns within tribe Brassiceae. *Plant J.*, **113**, 246–261.
67. Hao, Y., Mabry, M.E., Edger, P.P., Freeling, M., Zheng, C., Jin, L., VanBuren, R., Colle, M., An, H. and Abrahams, R.S. *et al.*. (2021) The contributions from the progenitor genomes of the mesopolyploid Brassiceae are evolutionarily distinct but functionally compatible. *Genome Res.*, **31**, 799–810.
68. Wang, X., Guo, H., Wang, J., Lei, T., Liu, T., Wang, Z., Li, Y., Lee, T.H., Li, J. and Tang, H. *et al.*. (2016) Comparative genomic de-convolution of the cotton genome revealed a decaploid ancestor and widespread chromosomal fractionation. *New Phytol.*, **209**, 1252–1263.
69. Wang, M., Li, J., Qi, Z., Long, Y., Pei, L., Huang, X., Grover, C.E., Du, X., Xia, C. and Wang, P. *et al.*. (2022) Genomic innovation and regulatory rewiring during evolution of the cotton genus *Gossypium*. *Nat. Genet.*, **54**, 1959–1971.
70. Hu, H., Yang, Y., Li, A., Zheng, Z., Zhang, J. and Liu, J. (2022) Genomic divergence of *Stellera chamaejasme* through local selection across the Qinghai–Tibet plateau and northern China. *Mol. Ecol.*, **31**, 4782–4796.
71. Bai, G., Chen, C., Zhao, C., Zhou, T., Li, D., Zhou, T., Li, W., Lu, Y., Cong, X. and Jia, Y. *et al.*. (2022) The chromosome-level genome for *Toxicodendron vernicifluum* provides crucial insights into Anacardiaceae evolution and urushiol biosynthesis. *iScience*, **25**, 104512.
72. Wang, X., Xiao, Y., He, Z., Li, L., Lv, Y. and Hu, X. (2022) Evolutionary divergence between

Toona ciliata and *Toona sinensis* assayed with their whole genome sequences. *Genes*, **13**, 1799.

73. Käfer, J., Bewick, A., Andres-Robin, A., Lapetoule, G., Harkess, A., Caïus, J., Fogliani, B., Gâteblé, G., Ralph, P. and DePamphilis, C.W. *et al.*. (2021) A derived ZW chromosome system in *Amborella trichopoda*, representing the sister lineage to all other extant flowering plants. *New Phytol.*, **4**, 1636–1642.
74. Pootakham, W., Naktang, C., Kongkachana, W., Sonthirod, C., Yoocha, T., Sangsrakru, D., Jomchai, N., U-thoomporn, S., Romyanon, K. and Toojinda, T. *et al.*. (2021) *De novo* chromosome-level assembly of the *Centella asiatica* genome. *Genomics*, **113**, 2221–2228.
75. Song, X., Wang, J., Li, N., Yu, J., Meng, F., Wei, C., Liu, C., Chen, W., Nie, F. and Zhang, Z. *et al.*. (2019) Deciphering the high quality genome sequence of coriander that causes controversial feelings. *Plant Biotechnol. J.*, 13310.
76. Iorizzo, M., Ellison, S., Senalik, D., Zeng, P., Satapoomin, P., Huang, J., Bowman, M., Iovene, M., Sanseverino, W. and Cavagnaro, P. (2016) A high-quality carrot genome assembly provides new insights into carotenoid accumulation and asterid genome evolution. *Nat. Genet.*, **48**, 657.
77. Yang, Z., Chen, S., Wang, S., Hu, Y., Zhang, G., Dong, Y., Yang, S., Miao, J., Chen, W. and Sheng, J. (2021) Chromosomal-scale genome assembly of *Eleutherococcus senticosus* provides insights into chromosome evolution in Araliaceae. *Mol. Ecol. Resour.*, **21**, 2204–2220.
78. Wang, Z., Wang, X., Lu, T., Li, M., Jiang, P., Zhao, J., Liu, S., Fu, X., Wendel, J.F. and Van de Peer, Y. *et al.*. (2022) Reshuffling of the ancestral core-eudicot genome shaped chromatin topology and epigenetic modification in *Panax*. *Nat. Commun.*, **13**, 1902.
79. Fan, G., Liu, X., Sun, S., Shi, C., Du, X., Han, K., Yang, B., Fu, Y., Liu, M. and Seim, I. *et al.*. (2020) The chromosome level genome and genome-wide association study for the agronomic traits of *Panax notoginseng*. *iScience*, **23**, 101538.
80. Yao, X., Lu, Z., Song, Y., Hu, X. and Corlett, R.T. (2022) A chromosome-scale genome assembly for the holly (*Ilex polyneura*) provides insights into genomic adaptations to elevation in Southwest China. *Hortic. Res.*, **9**, uhab49.
81. Badouin, H., Gouzy, J., Grassa, C.J., Murat, F., Staton, S.E., Cottret, L., Lelandais-Briere, C., Owens, G.L., Carrere, S. and Mayjonade, B. *et al.*. (2017) The sunflower genome provides insights into oil metabolism, flowering and Asterid evolution. *Nature*, **546**, 148–152.
82. Liu, B., Yan, J., Li, W., Yin, L., Li, P., Yu, H., Xing, L., Cai, M., Wang, H. and Zhao, M. *et al.*. (2020) *Mikania micrantha* genome provides insights into the molecular mechanism of rapid growth. *Nat. Commun.*, **11**, 340.
83. Xu, X., Yuan, H., Yu, X., Huang, S., Sun, Y., Zhang, T., Liu, Q., Tong, H., Zhang, Y. and Wang, Y. *et al.*. (2021) The chromosome-level *Stevia* genome provides insights into steviol glycoside biosynthesis. *Hortic. Res.*, **8**, 129.
84. Lin, T., Xu, X., Du, H., Fan, X., Chen, Q., Hai, C., Zhou, Z., Su, X., Kou, L. and Gao, Q. *et al.*. (2022) Extensive sequence divergence between the reference genomes of *Taraxacum kok-saghyz* and *Taraxacum mongolicum*. *Sci. China Life Sci.*, **65**, 515–528.
85. Jia, Y., Chen, S., Chen, W., Zhang, P., Su, Z., Zhang, L., Xu, M. and Guo, L. (2022) A chromosome-level reference genome of Chinese balloon flower (*Platycodon grandiflorus*). *Front. Genet.*, **13**, 869784.
86. Yang, J., Qian, Z., Shi, T., Li, Z. and Chen, J. (2022) Chromosome-level genome assembly of the aquatic plant *Nymphoides indica* reveals transposable element bursts and NBS-LRR gene family expansion shedding light on its invasiveness. *DNA Res.*, **29**, dsac22.

87. Tang, C.Y., Li, S., Wang, Y.T. and Wang, X. (2020) Comparative genome/transcriptome analysis probes Boraginales' phylogenetic position, WGDs in Boraginales, and key enzyme genes in the alkannin/shikonin core pathway. *Mol. Ecol. Resour.*, **20**, 228–241.
88. Zhang, H., Du, X., Dong, C., Zheng, Z., Mu, W., Zhu, M., Yang, Y., Li, X., Hu, H. and Shrestha, N. *et al.*. (2022) Genomes and demographic histories of the endangered *Bretschneidera sinensis* (Akaniaceae). *Gigascience*, **11**, giac50.
89. Fernandez Pozo, N., Metz, T., Chandler, J.O., Gramzow, L., Mérai, Z., Maumus, F., Mittelsten Scheid, O., Theißen, G., Schranz, M.E. and Leubner Metzger, G. *et al.*. (2021) *Aethionema arabicum* genome annotation using PacBio full-length transcripts provides a valuable resource for seed dormancy and Brassicaceae evolution research. *Plant J.*, **106**, 275–293.
90. Cheng, C.Y., Krishnakumar, V., Chan, A.P., Thibaud Nissen, F., Schobel, S. and Town, C.D. (2017) Araport11: a complete reannotation of the *Arabidopsis thaliana* reference genome. *Plant J.*, **89**, 789–804.
91. Jiang, X., Song, Q., Ye, W. and Chen, Z.J. (2021) Concerted genomic and epigenomic changes accompany stabilization of *Arabidopsis* allopolyploids. *Nat. Ecol. Evol.*, **5**, 1382–1393.
92. Zhang, Z., Guo, J., Cai, X., Li, Y., Xi, X., Lin, R., Liang, J., Wang, X. and Wu, J. (2022) Improved reference genome annotation of *Brassica rapa* by Pacific Biosciences RNA sequencing. *Front. Plant Sci.*, **13**, 841618.
93. Yue, J., VanBuren, R., Liu, J., Fang, J., Zhang, X., Liao, Z., Wai, C.M., Xu, X., Chen, S. and Zhang, S. *et al.*. (2022) SunUp and Sunset genomes revealed impact of particle bombardment mediated transformation and domestication history in papaya. *Nat. Genet.*, **54**, 715–724.
94. Hoang, N.V., Sogbohossou, E.O.D., Xiong, W., Simpson, C.J.C., Singh, P., Walden, N., van den Bergh, E., Becker, F.F.M., Li, Z. and Zhu, X. *et al.*. (2023) The *Gynandropsis gynandra* genome provides insights into whole-genome duplications and the evolution of C4 photosynthesis in Cleomaceae. *Plant Cell*, **35**, 1334–1359.
95. Wang, Z., Li, Y., Sun, P., Zhu, M., Wang, D., Lu, Z., Hu, H., Xu, R., Zhang, J. and Ma, J. *et al.*. (2022) A high-quality *Buxus austro-yunnanensis* (Buxales) genome provides new insights into karyotype evolution in early eudicots. *BMC Biol.*, **20**, 216.
96. Shen, S., Li, N., Wang, Y., Zhou, R., Sun, P., Lin, H., Chen, W., Yu, T., Liu, Z. and Wang, Z. *et al.*. (2022) High-quality ice plant reference genome analysis provides insights into genome evolution and allows exploration of genes involved in the transition from C3 to CAM pathways. *Plant Biotechnol. J.*, **20**, 2107–2122.
97. Lightfoot, D.J., Jarvis, D.E., Ramaraj, T., Lee, R., Jellen, E.N. and Maughan, P.J. (2017) Single-molecule sequencing and Hi-C-based proximity-guided assembly of amaranth (*Amaranthus hypochondriacus*) chromosomes provide insights into genome evolution. *BMC Biol.*, **15**, 74.
98. Chen, J., Xie, F., Cui, Y., Chen, C., Lu, W., Hu, X., Hua, Q., Zhao, J., Wu, Z. and Gao, D. *et al.*. (2021) A chromosome-scale genome sequence of pitaya (*Hylocereus undatus*) provides novel insights into the genome evolution and regulation of betalain biosynthesis. *Hortic. Res.*, **8**, 164.
99. Li, F., Gao, Y., Jin, C., Wen, X., Geng, H., Cheng, Y., Qu, H., Liu, X., Feng, S. and Zhang, F. *et al.*. (2022) The chromosome-level genome of *Gypsophila paniculata* reveals the molecular mechanism of floral development and ethylene insensitivity. *Hortic. Res.*, **9**, uhac176.
100. McGrath, J.M., Funk, A., Galewski, P., Ou, S., Townsend, B., Davenport, K., Daligault, H., Johnson, S., Lee, J. and Hastie, A. *et al.*. (2023) A contiguous *de novo* genome assembly of sugar

beet EL10 (*Beta vulgaris* L.). *DNA Res.*, **30**, dsac33.

101. Cai, X., Sun, X., Xu, C., Sun, H., Wang, X., Ge, C., Zhang, Z., Wang, Q., Fei, Z. and Jiao, C. *et al.*. (2021) Genomic analyses provide insights into spinach domestication and the genetic basis of agronomic traits. *Nat. Commun.*, **12**, 7246.
102. Yuan, F., Wang, X., Zhao, B., Xu, X., Shi, M., Leng, B., Dong, X., Lu, C., Feng, Z. and Guo, J. *et al.*. (2022) The genome of the recretohalophyte *Limonium bicolor* provides insights into salt gland development and salinity adaptation during terrestrial evolution. *Mol. Plant.*, **15**, 1024–1044.
103. Gilman, I.S., Moreno-Villena, J.J., Lewis, Z.R., Goolsby, E.W. and Edwards, E.J. (2022) Gene co-expression reveals the modularity and integration of C4 and CAM in *Portulaca*. *Plant Physiol.*, **189**, 735–753.
104. Sturtevant, D., Lu, S., Zhou, Z., Shen, Y., Wang, S., Song, J., Zhong, J., Burks, D.J., Yang, Z. and Yang, Q. *et al.*. (2020) The genome of jojoba (*Simmondsia chinensis*): A taxonomically isolated species that directs wax ester accumulation in its seeds. *Sci. Adv.*, **6**, eaay3240.
105. Dong, C., Wang, S., Zhang, H., Liu, J. and Li, M. (2023) Karyotype evolution of the Asterids insights from the first genome sequences of the family Cornaceae. *DNA Res.*, **30**, dsac51.
106. Kang, M., Fu, R., Zhang, P., Lou, S., Yang, X., Chen, Y., Ma, T., Zhang, Y., Xi, Z. and Liu, J. (2021) A chromosome-level *Camptotheca acuminata* genome assembly provides insights into the evolutionary origin of camptothecin biosynthesis. *Nat. Commun.*, **12**, 3531.
107. Yang, X., Kang, M., Yang, Y., Xiong, H., Wang, M., Zhang, Z., Wang, Z., Wu, H., Ma, T. and Liu, J. *et al.*. (2019) A chromosome-level genome assembly of the Chinese tupelo *Nyssa sinensis*. *Sci. Data*, **6**, 282.
108. Li, L., Chen, X., Fang, D., Dong, S., Guo, X., Li, N., Campos Dominguez, L., Wang, W., Liu, Y. and Lang, X. *et al.*. (2022) Genomes shed light on the evolution of *Begonia*, a mega-diverse genus. *New Phytol.*, **234**, 295–310.
109. Zhao, S., Guo, J., Kong, L., Nie, S., Yan, X., Shi, T., Tian, X., Ma, H., Bao, Y. and Li, Z. *et al.*. (2023) Haplotype-resolved genome assembly of *Coriaria nepalensis* a non-legume nitrogen-fixing shrub. *Sci. Data*, **10**, 259.
110. Deng, Y., Liu, S., Zhang, Y., Tan, J., Li, X., Chu, X., Xu, B., Tian, Y., Sun, Y. and Li, B. *et al.*. (2022) A telomere-to-telomere gap-free reference genome of watermelon and its mutation library provide important resources for gene discovery and breeding. *Mol. Plant.*, **15**, 1268–1284.
111. Barrera-Redondo, J., Sánchez-de La Vega, G., Aguirre-Liguori, J.A., Castellanos-Morales, G., Gutiérrez-Guerrero, Y.T., Aguirre-Dugua, X., Aguirre-Planter, E., Tenaillon, M.I., Lira-Saade, R. and Eguiarte, L.E. (2021) The domestication of *Cucurbita argyrosperma* as revealed by the genome of its wild relative. *Hortic. Res.*, **8**, 109.
112. Montero-Pau, J., Blanca, J., Bombarely, A., Ziarsolo, P., Esteras, C., Marti-Gomez, C., Ferriol, M., Gomez, P., Jamilena, M. and Mueller, L. *et al.*. (2018) *De novo* assembly of the zucchini genome reveals a whole-genome duplication associated with the origin of the *Cucurbita* genus. *Plant Biotechnol. J.*, **16**, 1161–1171.
113. Zhang, T., Ren, X., Zhang, Z., Ming, Y., Yang, Z., Hu, J., Li, S., Wang, Y., Sun, S. and Sun, K. *et al.*. (2020) Long-read sequencing and *de novo* assembly of the *Luffa cylindrica* (L.) Roem. genome. *Mol. Ecol. Resour.*, **20**, 511–519.
114. Pu, X., Li, Z., Tian, Y., Gao, R., Hao, L., Hu, Y., He, C., Sun, W., Xu, M. and Peters, R.J. *et al.*. (2020) The honeysuckle genome provides insight into the molecular mechanism of carotenoid metabolism underlying dynamic flower coloration. *New Phytol.*, **227**, 930–943.

115. Wu, H., Ma, T., Kang, M., Ai, F., Zhang, J., Dong, G. and Liu, J. (2019) A high-quality *Actinidia chinensis* (kiwifruit) genome. *Hortic. Res.*, **6**, 117.
116. Akagi, T., Shirasawa, K., Nagasaki, H., Hirakawa, H., Tao, R., Comai, L. and Henry, I.M. (2020) The persimmon genome reveals clues to the evolution of a lineage-specific sex determination system in plants. *PLoS Genet.*, **16**, e1008566.
117. Wu, X., Zhang, L., Wang, X., Zhang, R., Jin, G., Hu, Y., Yang, H., Wu, Z., Ma, Y. and Zhang, C. *et al.*. (2023) Evolutionary history of two evergreen *Rhododendron* species as revealed by chromosome-level genome assembly. *Front. Plant Sci.*, **14**, 1123707.
118. Colle, M., Leisner, C.P., Wai, C.M., Ou, S., Bird, K.A., Wang, J., Wisecaver, J.H., Yocca, A.E., Alger, E.I. and Tang, H. *et al.*. (2019) Haplotype-phased genome and evolution of phytonutrient pathways of tetraploid blueberry. *Gigascience*, **8**, giz12.
119. Jarvis, D.E., Maughan, P.J., DeTemple, J., Mosquera, V., Li, Z., Barker, M.S., Johnson, L.A. and Whipple, C.J. (2022) Chromosome-scale genome assembly of *Gilia yorkii* enables genetic mapping of floral traits in an interspecies cross. *Genome Biol. Evol.*, **14**, evac17.
120. Ma, D., Guo, Z., Ding, Q., Zhao, Z., Shen, Z., Wei, M., Gao, C., Zhang, L., Li, H. and Zhang, S. *et al.*. (2021) Chromosome-level assembly of the mangrove plant *Aegiceras corniculatum* genome generated through Illumina, PacBio and Hi-C sequencing technologies. *Mol. Ecol. Resour.*, **21**, 1593–1607.
121. Hale, I., Ma, X., Melo, A.T.O., Padi, F.K., Hendre, P.S., Kingan, S.B., Sullivan, S.T., Chen, S., Boffa, J. and Muchugi, A. *et al.*. (2021) Genomic resources to guide improvement of the shea tree. *Front. Plant Sci.*, **12**, 720670.
122. Chen, J., Zheng, C., Ma, J., Jiang, C., Ercisli, S., Yao, M. and Chen, L. (2020) The chromosome-scale genome reveals the evolution and diversification after the recent tetraploidization event in tea plant. *Hortic. Res.*, **7**, 63.
123. Garg, V., Dudchenko, O., Wang, J., Khan, A.W., Gupta, S., Kaur, P., Han, K., Saxena, R.K., Kale, S.M. and Pham, M. *et al.*. (2022) Chromosome-length genome assemblies of six legume species provide insights into genome organization, evolution, and agronomic traits for crop improvement. *J. Adv. Res.*, **42**, 315–329.
124. Li, J., Shen, J., Wang, R., Chen, Y., Zhang, T., Wang, H., Guo, C. and Qi, J. (2023) The nearly complete assembly of the *Cercis chinensis* genome and Fabaceae phylogenomic studies provide insights into new gene evolution. *Plant Commun.*, **4**, 100422.
125. Liu, Y., Du, H., Li, P., Shen, Y., Peng, H., Liu, S., Zhou, G., Zhang, H., Liu, Z. and Shi, M. *et al.*. (2020) Pan-genome of wild and cultivated soybeans. *Cell*, **182**, 162–176.
126. Xu, W., Zhang, Q., Yuan, W., Xu, F., Muhammad Aslam, M., Miao, R., Li, Y., Wang, Q., Li, X. and Zhang, X. *et al.*. (2020) The genome evolution and low-phosphorus adaptation in white lupin. *Nat. Commun.*, **11**, 1069.
127. Reed, J., Orme, A., El-Demerdash, A., Owen, C., Martin, L.B.B., Misra, R.C., Kikuchi, S., Rejzek, M., Martin, A.C. and Harkess, A. *et al.*. (2023) Elucidation of the pathway for biosynthesis of saponin adjuvants from the soapbark tree. *Science*, **379**, 1252–1264.
128. Li, Y., Sun, P., Lu, Z., Chen, J., Wang, Z., Du, X., Zheng, Z., Wu, Y., Hu, H. and Yang, J. *et al.*. (2021) The *Corylus mandshurica* genome provides insights into the evolution of Betulaceae genomes and hazelnut breeding. *Hortic. Res.*, **8**, 54.
129. Zhang, Y., Wei, Y., Meng, J., Wang, Y., Nie, S., Zhang, Z., Wang, H., Yang, Y., Gao, Y. and Wu, J. *et al.*. (2023) Chromosome-scale *de novo* genome assembly and annotation of three representative

Casuarina species: *C. equisetifolia* , *C. glauca*, and *C. cunninghamiana*. *Plant J.*, **114**, 1490–1505.

130. Han, B., Wang, L., Xian, Y., Xie, X., Li, W., Zhao, Y., Zhang, R., Qin, X., Li, D. and Jia, K. (2022) A chromosome-level genome assembly of the Chinese cork oak (*Quercus variabilis*). *Front. Plant Sci.*, **13**, 1001583.
131. Lovell, J.T., Bentley, N.B., Bhattarai, G., Jenkins, J.W., Sreedasyam, A., Alarcon, Y., Bock, C., Boston, L.B., Carlson, J. and Cervantes, K. *et al.*. (2021) Four chromosome scale genomes and a pan-genome annotation to accelerate pecan tree breeding. *Nat. Commun.*, **12**, 4125.
132. Zhang, W., Cao, L., Lin, X., Ding, Y., Liang, Y., Zhang, D., Pang, E., Renner, S.S. and Bai, W. (2022) Dead-end hybridization in walnut trees revealed by large-scale genomic sequence data. *Mol. Biol. Evol.*, **39**, msab308.
133. Jia, H., Jia, H., Cai, Q., Wang, Y., Zhao, H., Yang, W., Wang, G., Li, Y., Zhan, D. and Shen, Y. *et al.*. (2019) The red bayberry genome and genetic basis of sex determination. *Plant Biotechnol. J.*, **17**, 397–409.
134. Li, C., Wood, J.C., Vu, A.H., Hamilton, J.P., Rodriguez Lopez, C.E., Payne, R.M.E., Serna Guerrero, D.A., Gase, K., Yamamoto, K. and Vaillancourt, B. *et al.*. (2023) Single-cell multi-omics in the medicinal plant *Catharanthus roseus*. *Nat. Chem. Biol.*, **19**, 1031–1041.
135. Liu, Y., Tang, Q., Cheng, P., Zhu, M., Zhang, H., Liu, J., Zuo, M., Huang, C., Wu, C. and Sun, Z. *et al.*. (2020) Whole-genome sequencing and analysis of the Chinese herbal plant *Gelsemium elegans*. *Acta Pharm. Sin. B.*, **10**, 374–382.
136. Liang, Y., Li, F., Gao, Q., Jin, C., Dong, L., Wang, Q., Xu, M., Sun, F., Bi, B. and Zhao, P. *et al.*. (2022) The genome of *Eustoma grandiflorum* reveals the whole-genome triplication event contributing to ornamental traits in cultivated lisianthus. *Plant Biotechnol. J.*, **20**, 1856–1858.
137. Xu, W., Zhang, L., Cunningham, A.B., Li, S., Zhuang, H., Wang, Y. and Liu, A. (2020) Blue genome: chromosome-scale genome reveals the evolutionary and molecular basis of indigo biosynthesis in *Strobilanthes cusia*. *Plant J.*, **104**, 864–879.
138. Wang, M., Zhang, L. and Wang, Z. (2021) Chromosomal-level reference genome of the neotropical tree *Jacaranda mimosifolia* D. Don. *Genome Biol. Evol.*, **13**, evab94.
139. Feng, C., Wang, J., Wu, L., Kong, H., Yang, L., Feng, C., Wang, K., Rausher, M. and Kang, M. (2020) The genome of a cave plant, *Primulina huaijiensis*, provides insights into adaptation to limestone karst habitats. *New Phytol.*, **227**, 1249–1263.
140. Zhao, D., Hamilton, J.P., Buell, C.R., Hamberger, B., Johnson, S.R., Bhat, W.W., Godden, G.T., Soltis, P.S., Soltis, D.E. and Kinser, T.J. *et al.*. (2019) A chromosomal-scale genome assembly of *Tectona grandis* reveals the importance of tandem gene duplication and enables discovery of genes in natural product biosynthetic pathways. *Gigascience*, **8**, giz5.
141. VanBuren, R., Man, W.C., Pardo, J., Giarola, V., Ambrosini, S., Song, X. and Bartels, D. (2018) Desiccation tolerance evolved through gene duplication and network rewiring in *Lindernia*. *Plant Cell*, **30**, 2943–2958.
142. Yang, X., Yue, Y., Li, H., Ding, W., Chen, G., Shi, T., Chen, J., Park, M.S., Chen, F. and Wang, L. (2018) The chromosome-level quality genome provides insights into the evolution of the biosynthesis genes for aroma compounds of *Osmanthus fragrans*. *Hortic. Res.*, **5**, 72.
143. Wang, Y., Lu, L., Li, J., Li, H., You, Y., Zang, S., Zhang, Y., Ye, J., Lv, Z. and Zhang, Z. *et al.*. (2022) A chromosome-level genome of S*yringa oblata* provides new insights into chromosome formation in Oleaceae and evolutionary history of lilacs. *Plant J.*, **111**, 836–848.

144. Wang, M., Huang, J., Liu, S., Liu, X., Li, R., Luo, J. and Fu, Z. (2022) Improved assembly and annotation of the sesame genome. *DNA Res.*, **29**, dsac41.
145. Hellsten, U., Wright, K.M., Jenkins, J., Shu, S., Yuan, Y., Wessler, S.R., Schmutz, J., Willis, J.H. and Rokhsar, D.S. (2013) Fine-scale variation in meiotic recombination in *Mimulus* inferred from population shotgun sequencing. *Proc. Natl. Acad. Sci. U. S. A.*, **110**, 19478–19482.
146. Zhu, S., Zhang, Y.E., Copsy, L., Han, Q., Zheng, D., Coen, E. and Xue, Y. (2023) The snapdragon genomes teveal the evolutionary dynamics of the S-Locus supergene. *Mol. Biol. Evol.*, **40**, msad80.
147. Ma, Y.P., Wariss, H.M., Liao, R.L., Zhang, R.G., Yun, Q.Z., Olmstead, R.G., Chau, J.H., Milne, R.I., Van de Peer, Y. and Sun, W.B. (2021) Genome-wide analysis of butterfly bush (*Buddleja alternifolia*) in three uplands provides insights into biogeography, demography and speciation. *New Phytol.*, **232**, 1463–1476.
148. Hamilton, J.P., Vaillancourt, B., Wood, J.C. and Buell, C.R. (2023) Chromosome-scale assembly of the Verbenaceae species Queen's Wreath (*Petrea volubilis* L.). *BMC Genet.*, **24**, 14.
149. Chaw, S., Liu, Y., Wu, Y., Wang, H., Lin, C.I., Wu, C., Ke, H., Chang, L., Hsu, C. and Yang, H. *et al.*. (2019) Stout camphor tree genome fills gaps in understanding of flowering plant genome evolution. *Nat. Plants*, **5**, 63–73.
150. Chen, J., Hao, Z., Guang, X., Zhao, C., Wang, P., Xue, L., Zhu, Q., Yang, L., Sheng, Y. and Zhou, Y. *et al.*. (2019) *Liriodendron* genome sheds light on angiosperm phylogeny and species–pair differentiation. *Nat. Plants*, **5**, 18–25.
151. Johnson, A.R., Yue, Y., Carey, S.B., Park, S.J., Kruse, L.H., Bao, A., Pasha, A., Harkess, A., Provart, N.J. and Moghe, G.D. *et al.*. (2023) Chromosome-level genome assembly of *Euphorbia peplus*, a model system for plant latex, reveals that relative lack of Ty3 transposons contributed to its small genome size. *Genome Biol. Evol.*, **15**, evad18.
152. Bredeson, J.V., Lyons, J.B., Prochnik, S.E., Wu, G.A., Ha, C.M., Edsinger-Gonzales, E., Grimwood, J., Schmutz, J., Rabbi, I.Y. and Egesi, C. *et al.*. (2016) Sequencing wild and cultivated cassava and related species reveals extensive interspecific hybridization and genetic diversity. *Nat. Biotechnol.*, **34**, 562–570.
153. Gutiérrez-Valencia, J., Fracassetti, M., Berdan, E.L., Bunikis, I., Soler, L., Dainat, J., Kutschera, V.E., Losvik, A., Désamoré, A. and Hughes, P.W. *et al.*. (2022) Genomic analyses of the *Linum distyly* supergene reveal convergent evolution at the molecular level. *Curr. Biol.*, **32**, 4360–4371.
154. Xia, Z., Huang, D., Zhang, S., Wang, W., Ma, F., Wu, B., Xu, Y., Xu, B., Chen, D. and Zou, M. *et al.*. (2021) Chromosome-scale genome assembly provides insights into the evolution and flavor synthesis of passion fruit (*Passiflora edulis* Sims). *Hortic. Res.*, **8**, 14.
155. Xue, T., Zheng, X., Chen, D., Liang, L., Chen, N., Huang, Z., Fan, W., Chen, J., Cen, W. and Chen, S. *et al.*. (2020) A high-quality genome provides insights into the new taxonomic status and genomic characteristics of *Cladopus chinensis* (Podostemaceae). *Hortic. Res.*, **7**, 46.
156. Pootakham, W., Naktang, C., Sonthirod, C., Kongkachana, W., Yoocha, T., Jomchai, N., Maknual, C., Chumriang, P., Pravinvongvuthi, T. and Tangphatsornruang, S. (2022) *De novo* reference assembly of the upriver orange mangrove (*Bruguiera sexangula*) genome. *Genome Biol. Evol.*, **14**, evac25.
157. Pootakham, W., Naktang, C., Sonthirod, C., Kongkachana, W., Narong, N., Sangsrakru, D., Maknual, C., Jiumjamrassil, D., Chumriang, P. and Tangphatsornruang, S. (2022) Chromosome-level genome assembly of Indian mangrove (*Ceriops tagal*) revealed a

genome-wide duplication event predating the divergence of Rhizophoraceae mangrove species. *Plant Genome*, **15**, e20217.

158. Zhang, S., Wu, Z., Ma, D., Zhai, J., Han, X., Jiang, Z., Liu, S., Xu, J., Jiao, P. and Li, Z. (2022) Chromosome-scale assemblies of the male and female *Populus euphratica* genomes reveal the molecular basis of sex determination and sexual dimorphism. *Commun. Biol.*, **5**, 1186.
159. Tuskan, G.A., Difazio, S., Jansson, S., Bohlmann, J., Grigoriev, I., Hellsten, U., Putnam, N., Ralph, S., Rombauts, S. and Salamov, A. (2006) The genome of black cottonwood, *Populus trichocarpa* (Torr. & Gray). *Science*, **313**, 1596–1604.
160. Chen, J., Huang, Y., Brachi, B., Yun, Q., Zhang, W., Lu, W., Li, H., Li, W., Sun, X. and Wang, G. *et al.*. (2019) Genome-wide analysis of Cushion willow provides insights into alpine plant divergence in a biodiversity hotspot. *Nat. Commun.*, **10**, 5230.
161. He, L., Jia, K.H., Zhang, R.G., Wang, Y., Shi, T.L., Li, Z.C., Zeng, S.W., Cai, X.J., Wagner, N.D. and Hörandl, E. *et al.*. (2021) Chromosome-scale assembly of the genome of *Salix dunnii* reveals a male-heterogametic sex determination system on chromosome 7. *Mol. Ecol. Resour.*, **21**, 1966–1982.
162. Wang, S., Liang, H., Wang, H., Li, L., Xu, Y., Liu, Y., Liu, M., Wei, J., Ma, T. and Le C *et al.*. (2022) The chromosome-scale genomes of *Dipterocarpus turbinatus* and *Hopea hainanensis* (Dipterocarpaceae) provide insights into fragrant oleoresin biosynthesis and hardwood formation. *Plant Biotechnol. J.*, **20**, 538–553.
163. Udall, J.A., Long, E., Ramaraj, T., Conover, J.L., Yuan, D., Grover, C.E., Gong, L., Arick, N.M.A., Masonbrink, R.E. and Peterson, D.G. *et al.*. (2019) The genome sequence of *Gossypioides kirkii* illustrates a descending dysploidy in plants. *Front. Plant Sci.*, **10**, 1541.
164. Motamayor, J.C., Mockaitis, K., Schmutz, J., Haiminen, N., Livingstone, D.R., Cornejo, O., Findley, S.D., Zheng, P., Utro, F. and Royaert, S. *et al.*. (2013) The genome sequence of the most widely cultivated cacao type and its use to identify candidate genes regulating pod color. *Genome Biol.*, **14**, r53.
165. Ding, X., Mei, W., Lin, Q., Wang, H., Wang, J., Peng, S., Li, H., Zhu, J., Li, W. and Wang, P. *et al.*. (2020) Genome sequence of the agarwood tree *Aquilaria sinensis* (Lour.) Spreng: the first chromosome-level draft genome in the Thymelaeceae family. *Gigascience*, **9**, giaa13.
166. He, Z., Feng, X., Chen, Q., Li, L., Li, S., Han, K., Guo, Z., Wang, J., Liu, M. and Shi, C. *et al.*. (2022) Evolution of coastal forests based on a full set of mangrove genomes. *Nat. Ecol. Evol.*, **6**, 738–749.
167. Luo, X., Li, H., Wu, Z., Yao, W., Zhao, P., Cao, D., Yu, H., Li, K., Poudel, K. and Zhao, D. *et al.*. (2019) The pomegranate (*Punica granatum* L.) draft genome dissects genetic divergence between soft- and hard-seeded cultivars. *Plant Biotechnol. J.*, **18**, 955–968.
168. Hao, Y., Zhou, Y., Chen, B., Chen, G., Wen, Z., Zhang, D., Sun, W., Liu, D., Huang, J. and Chen, J. *et al.*. (2022) The *Melastoma dodecandrum* genome and the evolution of Myrtales. *J. Genet. Genomics*, **49**, 120–131.
169. Myburg, A., Grattapaglia, D., Tuskan, G., Hellsten, U., Hayes, R., Grimwood, J., Jenkins, J., Lindquist, E., Tice, H. and Bauer, D. (2014) The genome of *Eucalyptus grandis*. *Nature*, **510**, 356–362.
170. Wu, S., Sun, W., Xu, Z., Zhai, J., Li, X., Li, C., Zhang, D., Wu, X., Shen, L. and Chen, J. *et al.*. (2020) The genome sequence of star fruit (*Averrhoa carambola*). *Hortic. Res.*, **7**, 95.
171. Zhu, T., Wang, L., Rimbert, H., Rodriguez, J.C., Deal, K.R., De Oliveira, R., Choulet, F.,

Keeble-Gagnère, G., Tibbits, J. and Rogers, J. *et al.*. (2021) Optical maps refine the bread wheat *Triticum aestivum* cv. Chinese Spring genome assembly. *Plant J.*, **107**, 303–314.

172. Maccaferri, M., Harris, N.S., Twardziok, S.O., Pasam, R.K., Gundlach, H., Spannagl, M., Ormanbekova, D., Lux, T., Prade, V.M. and Milner, S.G. *et al.*. (2019) Durum wheat genome highlights past domestication signatures and future improvement targets. *Nat. Genet.*, **51**, 885–895.
173. Li, H., Yang, X., Zhang, Y., Gao, Z., Liang, Y., Chen, J. and Shi, T. (2021) *Nelumbo* genome database, an integrative resource for gene expression and variants of *Nelumbo nucifera*. *Sci. Data*, **8**, 38.
174. Lin, J., Zhang, W., Zhang, X., Ma, X., Zhang, S., Chen, S., Wang, Y., Jia, H., Liao, Z. and Lin, J. *et al.*. (2022) Signatures of selection in recently domesticated macadamia. *Nat. Commun.*, **13**, 242.
175. Chen, S.H., Rossetto, M., van der Merwe, M., Lu Irving, P., Yap, J.Y.S., Sauquet, H., Bourke, G., Amos, T.G., Bragg, J.G. and Edwards, R.J. (2022) Chromosome-level de novo genome assembly of *Telopea speciosissima* (New South Wales waratah) using long-reads, linked-reads and Hi-C. *Mol. Ecol. Resour.*, **22**, 1836–1854.
176. Zhong, S., Li, B., Chen, W., Wang, L., Guan, J., Wang, Q., Yang, Z., Yang, H., Wang, X. and Yu, X. *et al.*. (2022) The chromosome-level genome of *Akebia trifoliata* as an important resource to study plant evolution and environmental adaptation in the Cretaceous. *Plant J.*, **112**, 1316–1330.
177. Yang, X., Gao, S., Guo, L., Wang, B., Jia, Y., Zhou, J., Che, Y., Jia, P., Lin, J. and Xu, T. *et al.*. (2021) Three chromosome-scale *Papaver* genomes reveal punctuated patchwork evolution of the morphinan and noscapine biosynthesis pathway. *Nat. Commun.*, **12**, 6030.
178. Filiault, D.L., Ballerini, E.S., Mandáková, T., Aköz, G., Derieg, N.J., Schmutz, J., Jenkins, J., Grimwood, J., Shu, S. and Hayes, R.D. *et al.*. (2018) The *Aquilegia* genome provides insight into adaptive radiation and reveals an extraordinarily polymorphic chromosome with a unique history. *Elife*, **7**, e36426.
179. Liu, Y., Wang, B., Shu, S., Li, Z., Song, C., Liu, D., Niu, Y., Liu, J., Zhang, J. and Liu, H. *et al.*. (2021) Analysis of the *Coptis chinensis* genome reveals the diversification of protoberberine-type alkaloids. *Nat. Commun.*, **12**, 3276.
180. van Bakel, H., Stout, J.M., Cote, A.G., Tallon, C.M., Sharpe, A.G., Hughes, T.R. and Page, J.E. (2011) The draft genome and transcriptome of *Cannabis sativa*. *Genome Biol.*, **12**, R102.
181. Wu, Z., Chen, H., Pan, Y., Feng, H., Fang, D., Yang, J., Wang, Y., Yang, J., Sahu, S.K. and Liu, J. *et al.*. (2022) Genome of *Hippophae rhamnoides* provides insights into a conserved molecular mechanism in actinorhizal and rhizobial symbioses. *New Phytol.*, **235**, 276–291.
182. Wang, R., Yang, Y., Jing, Y., Segar, S.T., Zhang, Y., Wang, G., Chen, J., Liu, Q., Chen, S. and Chen, Y. *et al.*. (2021) Molecular mechanisms of mutualistic and antagonistic interactions in a plant–pollinator association. *Nat. Ecol. Evol.*, **5**, 974–986.
183. Shen, L., Luo, H., Wang, X., Wang, X., Qiu, X., Liu, H., Zhou, S., Jia, K., Nie, S. and Bao, Y. *et al.*. (2021) Chromosome-scale genome assembly for Chinese sour jujube and insights into its genome evolution and domestication signature. *Front. Plant Sci.*, **12**, 2552.
184. Edger, P.P., McKain, M.R., Yocca, A.E., Knapp, S.J., Qiao, Q. and Zhang, T. (2020) Reply to: Revisiting the origin of octoploid strawberry. *Nat. Genet.*, **52**, 5–7.
185. Hirakawa, H., Shirasawa, K., Kosugi, S., Tashiro, K., Nakayama, S., Yamada, M., Kohara, M., Watanabe, A., Kishida, Y. and Fujishiro, T. *et al.*. (2014) Dissection of the octoploid strawberry genome by deep sequencing of the genomes of *Fragaria* species. *DNA Res.*, **21**, 169–181.

186. Zhou, Y., Xiong, J., Shu, Z., Dong, C., Gu, T., Sun, P., He, S., Jiang, M., Xia, Z. and Xue, J. *et al.*. (2023) The telomere-to-telomere genome of *Fragaria vesca* reveals the genomic evolution of *Fragaria* and the origin of cultivated octoploid strawberry. *Hortic. Res.*, **10**, uhad27.
187. Feng, C., Wang, J., Harris, A.J., Folta, K.M., Zhao, M. and Kang, M. (2021) Tracing the diploid ancestry of the cultivated octoploid strawberry. *Mol. Biol. Evol.*, **38**, 478–485.
188. Fan, Z., Tieman, D.M., Knapp, S.J., Zerbe, P., Famula, R., Barbey, C.R., Folta, K.M., Amadeu, R.R., Lee, M. and Oh, Y. *et al.*. (2022) A multi-omics framework reveals strawberry flavor genes and their regulatory elements. *New Phytol.*, **236**, 1089–1107.
189. Su, W., Jing, Y., Lin, S., Yue, Z., Yang, X., Xu, J., Wu, J., Zhang, Z., Xia, R. and Zhu, J. *et al.*. (2021) Polyploidy underlies co-option and diversification of biosynthetic triterpene pathways in the apple tribe. *Proc. Natl. Acad. Sci. U. S. A.*, **118**, e2101767118.
190. Zhang, L., Hu, J., Han, X., Li, J., Gao, Y., Richards, C.M., Zhang, C., Tian, Y., Liu, G. and Gul, H. *et al.*. (2019) A high-quality apple genome assembly reveals the association of a retrotransposon and red fruit colour. *Nat. Commun.*, **10**, 1494.
191. Wang, Y., Li, F., He, Q., Bao, Z., Zeng, Z., An, D., Zhang, T., Yan, L., Wang, H. and Zhu, S. *et al.*. (2021) Genomic analyses provide comprehensive insights into the domestication of bast fiber crop ramie (*Boehmeria nivea*). *Plant J.*, **107**, 787–800.
192. Fu, J., Wan, L., Song, L., He, L., Jiang, N., Long, H., Huo, J., Ji, X., Hu, F. and Wei, S. *et al.*. (2022) Chromosome-level genome assembly of the hemiparasitic *Taxillus chinensis* (DC.) Danser. *Genome Biol. Evol.*, **14**, evac60.
193. Yang, T., Zhang, R., Tian, X., Yao, G., Shen, Y., Wang, S., Mao, J., Li, G., Liu, A. and Sun, W. *et al.*. (2023) The chromosome-level genome assembly and genes involved in biosynthesis of nervonic acid of *Malania oleifera*. *Sci. Data*, **10**, 298.
194. Wei, L., Xun-Ge, Z., Qun-Jie, Z., Kui, L., Dan, Z., Cong, S. and Li-Zhi, G. (2020) SMRT sequencing generates the chromosome-scale reference genome of tropical fruit mango, *Mangifera indica*. *bioRxiv*, 2020–2022.
195. Wang, X., Xiao, Y., He, Z., Li, L., Song, H.Y., Zhang, J., Cheng, X., Chen, X., Li, P. and Hu, X. (2022) A chromosome-level genome assembly of *Toona ciliata* (Meliaceae). *Genome Biol. Evol.*, **14**, evac121.
196. Ji, Y., Xiu, Z., Chen, C., Wang, Y., Yang, J., Sui, J., Jiang, S., Wang, P., Yue, S. and Zhang, Q. *et al.*. (2021) Long read sequencing of *Toona sinensis* (A. Juss) Roem: A chromosome-level reference genome for the family Meliaceae. *Mol. Ecol. Resour.*, **21**, 1243–1255.
197. Ma, X., Ru, D., Morales-Briones, D.F., Mei, F., Wu, J., Liu, J. and Wu, S. (2023) Genome sequence and salinity adaptation of the desert shrub *Nitraria sibirica* (Nitrariaceae, Sapindales). *DNA Res.*, **30**, dsad11.
198. Wu, B., Yu, Q., Deng, Z., Duan, Y., Luo, F. and Gmitter Jr, F. (2023) A chromosome-level phased genome enabling allele-level studies in sweet orange: a case study on citrus Huanglongbing tolerance. *Hortic. Res.*, **10**, uhac247.
199. Neumann, P., Oliveira, L., Jang, T., Novák, P., Koblížková, A., Schubert, V., Houben, A. and Macas, J. (2023) Disruption of the standard kinetochore in holocentric *Cuscuta* species. *Proc. Natl. Acad. Sci. U. S. A.*, **120**, e1994090176.
200. Wu, Y., Li, D., Hu, Y., Li, H., Ramstein, G.P., Zhou, S., Zhang, X., Bao, Z., Zhang, Y. and Song, B. *et al.*. (2023) Phylogenomic discovery of deleterious mutations facilitates hybrid potato breeding. *Cell*, **186**, 2313–2328.

201. Prashant, S.H., Mirella, F., Henri, V.D.G., Florian, M., Linda, V.B., Elio, S., Jan, V.H., Jan, C., Gabino, S. and Sander, P. *et al.*. (2019) An improved de novo assembly and annotation of the tomato reference genome using single-molecule sequencing, Hi-C proximity ligation and optical maps. *bioRxiv*, 767764.
202. Strijk, J.S., Hinsinger, D.D., Zhang, F. and Cao, K. (2019) *Trochodendron aralioides*, the first chromosome-level draft genome in Trochodendrales and a valuable resource for basal eudicot research. *Gigascience*, **8**, giz136.
203. Jaillon, O., Aury, J.M., Noel, B., Policriti, A., Clepet, C., Casagrande, A., Choisne, N., Aubourg, S., Vitulo, N. and Jubin, C. *et al.*. (2007) The grapevine genome sequence suggests ancestral hexaploidization in major angiosperm phyla. *Nature*, **449**, 463–467.
204. Liu, B., Zhao, X., Wang, Z., Liu, H., Huang, X. and Yang, P. (2023) Chromosome-level genome assembly of the endangered plant *Tetraena mongolica*. *DNA Res.*, **30**, dsad4.
